# Using a combination of short- and long-read sequencing to investigate the diversity in plasmid- and chromosomally encoded extended-spectrum beta-lactamases (ESBLs) in clinical *Shigella* and *Salmonella* isolates in Belgium

**DOI:** 10.1099/mgen.0.000925

**Published:** 2023-01-23

**Authors:** Bas Berbers, Kevin Vanneste, Nancy H. C. J. Roosens, Kathleen Marchal, Pieter-Jan Ceyssens, Sigrid C. J. De Keersmaecker

**Affiliations:** ^1^​ Transversal Activities in Applied Genomics, Sciensano, 1050 Brussels, Belgium; ^2^​ Department of Information Technology, IDLab, Ghent University, IMEC, 9052 Ghent, Belgium; ^3^​ Department of Plant Biotechnology and Bioinformatics, Ghent University, 9052 Ghent, Belgium; ^4^​ Bacterial Diseases, Sciensano, 1050 Brussels, Belgium

**Keywords:** antimicrobial resistance, extended spectrum beta-lactamase, *Shigella*, *Salmonella*, plasmids, whole genome sequencing

## Abstract

For antimicrobial resistance (AMR) surveillance, it is important not only to detect AMR genes, but also to determine their plasmidic or chromosomal location, as this will impact their spread differently. Whole-genome sequencing (WGS) is increasingly used for AMR surveillance. However, determining the genetic context of AMR genes using only short-read sequencing is complicated. The combination with long-read sequencing offers a potential solution, as it allows hybrid assemblies. Nevertheless, its use in surveillance has so far been limited. This study aimed to demonstrate its added value for AMR surveillance based on a case study of extended-spectrum beta-lactamases (ESBLs). ESBL genes have been reported to occur also on plasmids. To gain insight into the diversity and genetic context of ESBL genes detected in clinical isolates received by the Belgian National Reference Center between 2013 and 2018, 100 ESBL-producing *Shigella* and 31 ESBL-producing *Salmonella* were sequenced with MiSeq and a representative selection of 20 *Shigella* and six *Salmonella* isolates additionally with MinION technology, allowing hybrid assembly. The *bla*_CTX-M-15_ gene was found to be responsible for a rapid rise in the ESBL *Shigella* phenotype from 2017. This gene was mostly detected on multi-resistance-carrying IncFII plasmids. Based on clustering, these plasmids were determined to be distinct from the circulating plasmids before 2017. They were spread to different *Shigella* species and within *Shigella sonnei* between multiple genotypes. Another similar IncFII plasmid was detected after 2017 containing *bla*_CTX-M-27_ for which only clonal expansion occurred. Matches of up to 99 % to plasmids of various bacterial hosts from all over the world were found, but global alignments indicated that direct or recent ESBL-plasmid transfers did not occur. It is most likely that travellers introduced these in Belgium and subsequently spread them domestically. However, a clear link to a specific country could not be made. Moreover, integration of *bla*_CTX-M_ in the chromosome of two *Shigella* isolates was determined for the first time, and shown to be related to ISEcp1. In contrast, in *Salmonella *, ESBL genes were only found on plasmids, of which *bla*_CTX-M-55_ and IncHI2 were the most prevalent, respectively. No matching ESBL plasmids or cassettes were detected between clinical *Shigella* and *Salmonella* isolates. The hybrid assembly data allowed us to check the accuracy of plasmid prediction tools. MOB-suite showed the highest accuracy. However, these tools cannot replace the accuracy of long-read and hybrid assemblies. This study illustrates the added value of hybrid assemblies for AMR surveillance and shows that a strategy where even just representative isolates of a collection used for hybrid assemblies could improve international AMR surveillance as it allows plasmid tracking.

## Data Summary

Genome sequences are deposited at the NCBI Sequence Read Archive (SRA) and NCBI GenBank under accession number PRJNA846333.

**Impact Statement**

We show the importance of performing hybrid assemblies in order to achieve full reconstruction of plasmids based on the combination of short- and long-read sequencing to improve tracking of plasmids and AMR genes, whereas short-read assemblies can sometimes result in wrong conclusions about the genetic context of AMR genes. This was applied to investigate the diversity in plasmid- and chromosomally encoded extended-spectrum beta-lactamases (ESBLs) in a collection of clinical *Shigella* and *Salmonella* spp. isolated in Belgium between 2013 and 2018. Most of the ESBL genes were found to be present on plasmids. However, this approach additionally allowed us to detect for the first time the chromosomal integration of ESBL genes in the *Shigella* chromosome. While ESBL genes on plasmids are more easily spread, a chromosomal location represents a more stable situation in terms of AMR gene presence. The strategy used in this study, i.e. selecting a set of representative isolates for hybrid assemblies, combined with short-read sequencing of all isolates, could also be applied in a One-Health context to improve surveillance of circulating plasmids. Understanding how resistance develops and spreads is important to ensure that AMR detection tools remain up-to-date for indispensable surveillance and diagnosis.

## Introduction

Antibiotics remain of the utmost importance for the treatment of bacterial infections. However, due to the (in)appropriate use of antibiotics in humans and animals, the prevalence of bacterial antimicrobial resistance (AMR) has increased dramatically, constituting a major threat to global public health. It has been estimated that in 2050, if no actions are taken, the number of deaths caused by AMR will surpass those caused by cancer [1]. Within the global action plan to combat AMR put forward by the World Health Organization (WHO), understanding how resistance develops and spreads is an important objective, as this knowledge is important to ensure that AMR detection tools remain current for the indispensable surveillance and diagnosis of AMR [2].

Currently, there exist different methods to detect and characterize bacterial AMR [3–5]. The most commonly used detection methods in routine testing determine the phenotypic AMR. However, although being the most informative for therapeutic applications, with these techniques, no information on the nature (i.e. genotype) of the coding AMR genes in the bacterial genome and hence information on the underlying mechanism is obtained. The introduction of molecular/genotypic AMR detection methods (e.g. PCR and qPCR) partly addressed this issue. However, most of these require several sequential analyses to determine the complete AMR profile. Additionally, neither the phenotypic nor molecular-based detection methods allow us to collect information on the location of the AMR genes in the bacterial genome. AMR genes can either be localized on the chromosome or on mobile genetic elements such as plasmids. This has important consequences for understanding the spread of resistance. Plasmids can be exchanged between bacteria and even to bacteria of different species [6, 7]. However, plasmids can be lost by the bacteria when there is no selective pressure due to the energy requirements for maintaining plasmids. By contrast, the presence of AMR genes in chromosomal DNA is more stable than on plasmids and they are not as easily lost [8–11].

Whole genome sequencing (WGS) has been increasingly used as an all-in-one AMR detection/characterization tool. However, one of the most commonly used technologies in routine settings, i.e. based on short-read sequencing such as offered by Illumina MiSeq, does not allow reliable reconstruction of full plasmid sequences. Due to the frequent presence of large repetitive regions in plasmids, which can exceed the length of the sequencing reads, an ambiguity is created regarding where those reads belong in the genome [12]. This kind of ambiguity results in highly fragmented assemblies, where in most cases it is difficult to determine the location of smaller contigs. Newer technologies such as offered by Oxford Nanopore Technologies (ONT) and PacBio sequencing generate long reads, potentially surpassing the repetitive regions. However, this is accompanied with the caveat of a higher error rate compared to short-read technologies. This might impact the accurate detection of specific mutations involved in AMR, especially with insufficient sequencing depth [13]. This has been resolved in some studies by combining both the long and short reads in hybrid assemblies to reliably reconstruct plasmids [14–17]. However, this could prove costly if it were to be implemented for all isolates in routine surveillance of pathogens. Nevertheless, the application of this hybrid assembly approach would offer a powerful tool to National Reference Centres (NRCs) and other organizations/institutes that are in charge of surveillance of specific pathogens, especially if plasmids are involved.

One such example are extended-spectrum beta-lactamase (ESBL)-producing bacteria. ESBLs confer resistance to some of the most commonly prescribed antibiotics in humans, i.e. beta-lactam antibiotics including third-generation cephalosporins (such as ceftriaxone, cefotaxime and ceftazidime) [18]. In addition to ESBL-producing bacteria already being difficult to treat by conferring resistance to many beta-lactam antibiotics, they often harbour resistance genes to several other antibiotics, resulting in multidrug resistance [19]. ESBL-producing *Enterobacteriaceae* include several pathogenic species that cause gastrointestinal diseases (e.g. diarrhoea) such as *Escherichia coli, Salmonella* and *Shigella *. There exist several types of ESBL-encoding genes. In recent years, the *bla*_CTX-M_ genes have become the most dominant type worldwide [20, 21]. There are currently over 170 variants of *bla*_CTX-M_ reported and some are more prevalent than others depending on the geographical area. The most common variants worldwide are *bla*_CTX-M-14_ and *bla*_CTX-M-15_. *bla*_CTX-M_ originated in the chromosome of *Kluyvera* spp. from where it was integrated in a plasmid that spread to other bacterial species such as *E. coli *, *Shigella* and *Salmonella* through horizontal gene transfer [22]. Different plasmids are commonly detected depending on the *bla*_CTX-M_ variant; for example, the incompatibility (Inc) F plasmids are mostly associated with *bla*_CTX-M-15_, IncHI2 plasmids with *bla*_CTX-M-9_ and IncK plasmids with *bla*_CTX-M-14_ [23]. Each Inc plasmid group has some distinct traits associated with them; for example, IncF plasmids are low-copy, conjugative plasmids with sizes from 45 to 200 kb [24]. However, translocation of *the bla*_CTX-M_ gene to the chromosome has also been described in some bacterial species such as *E. coli* and *Salmonella* [25–27], but not yet in *Shigella *. Translocation of *bla*_CTX-M_ from the chromosome to a plasmid or vice versa occurs mostly through transposons, i.e. short sequences of a few kilobases containing functional genes (in this case *bla*_CTX-M_), a transposon gene and flanked by inverted repeats (IR), called the inverted repeat left (IRL) and the inverted repeat right (IRR). Both transposons and plasmids are also referred to as mobile genetic elements (MGEs) and contribute greatly to the worldwide dissemination of AMR genes [28–31]. The transposon gene responsible for most translocations of *bla_CTX-M_* is ISEcp1 [32, 33]. ISEcp1 transposons probably contain an imperfect IRR of 14 bp of which 12 bp is complementary to the IRL [34]. The efficiency of transposition is affected by the amount of mutations (and their locations) in the IRs [35]. It has also been reported that the presence of promoter sequences upstream of the IRR and a short distance between the transposon gene and the *bla*_CTX-M_ gene can lead to higher levels of *bla_CTX-M_* expression [36].

ESBL-producing *E. coli* have been described very extensively in previous studies [37–39]. While *Salmonella* is genetically different, *E. coli* and *Shigella* are very similar to each other (up to 99 % genome sequence identity). The classification between *E. coli* and *Shigella* is maintained because of their different pathogenesis, evolutionary history and distribution in each country [40]. The genus *Shigella* can be further subdivided into *Shigella dysenteriae *, *S. flexneri *, *S. boydii *, and *S. sonnei *. In developed countries, *S. sonnei* is the most commonly observed species, followed by *S. flexneri *, and is considered mostly to be travel-related. *S. flexneri* is more prevalent in developing countries and especially in children (<5 years), but *S. sonnei* is also on the rise in these countries [40]. Virulence of *Shigella* is dependent on the presence of a large ~200 kb virulence plasmid (also called pINV). The pINV is slightly different for each *Shigella* species but they all share a 31 kb core region [41]. *Salmonella* is divided into species, subspecies and serotypes (serovars), of which the subspecies *Salmonella enterica* subsp. *enterica* is mostly encountered in warm-blooded hosts, including humans, and represents 99.5 % of isolated *Salmonella *. Infections occur mostly through contaminated food or drinking water, which is also more common in developing countries due to poorer sanitation [42].

In Belgium, the national surveillance of *Shigella* and *Salmonella* isolated from human cases is performed by the NRC for *Salmonella* and *Shigella* (NRCSS). The NRCSS receives annually around 400 *Shigella* and 2000 *Salmonella* cultures on a voluntary basis from peripheral laboratories. The prevalence of ESBL isolates in *S. sonnei* is relatively high with around 10 % phenotypic resistance against cefotaxime and/or ceftazidime detected in recent years, while for *S. flexneri* and *Salmonella *, ESBL-producing isolates are detected more sporadically. Based on phenotypic and in-house genotyping assays [43], it was not previously possible to elucidate the location and genetic context of the corresponding ESBL genes in these isolates. A previous genomic-based study in Belgium demonstrated that an increase in ciprofloxacin resistance in *S. sonnei* and *S. flexneri* was caused by a combination of domestic circulation and international travel [44]. In contrast to *bla_CTX-M_* genes, ciprofloxacin resistance is mostly linked to chromosomal point mutations and only in a few cases by the mobile *qnr* gene. Moreover, a recent study by the NRCSS showed domestic transfer of multi-drug resistant (MDR) *Shigella* among men who have sex with men (MSM) [45]. Other studies have also described the worrisome trend of increased ESBL-producing *Shigella* in other countries [46–48]. In all of these studies, the ESBL genes were described as being located on plasmids, but this was not always confirmed with long-read sequencing or other techniques.

Therefore, to assess the added value of hybrid assemblies for AMR surveillance, in this study the WGS workflow previously developed [49] was applied to ESBL-producing *Shigella* and *Salmonella* isolated between 2013 and 2018 and received by the Belgian NRCSS. To gain more insight into the diversity of the ESBL genes and their genetic context, a selection of 100 ESBL-positive human *Shigella* and 31 ESBL-positive human *Salmonella* isolates were sequenced with the short-read MiSeq technology, and a subselection was additionally sequenced using the long-read ONT technology. The AMR content, plasmid replicons and transposases were determined for all isolates. Then the ESBL plasmids/chromosomes of the isolates sequenced with both technologies were reconstructed through hybrid assembly and used as references for comparisons with other isolates, and with sequences occurring in international databases. Several approaches were applied to investigate the genetic context of the ESBL genes for all isolates, including using prediction tools. Furthermore, clustering was performed to determine the similarity between all *Shigella* isolates and their plasmids and to evaluate whether these clusters were closely linked to isolation date or travel, in an attempt to explore the possible contribution of hybrid assemblies to the investigation of (international) transmission dynamics of the ESBL plasmids in *Shigella *. These results aim to deliver another proof-of-concept of how hybrid assemblies aid in AMR surveillance.

## Methods

### Bacterial isolates, culturing and DNA extraction

In this study 100 ESBL-positive *Shigella* (i.e. the most recent 100 isolates in the study period 2013–2018 taken in a consecutive, unbiased way) and 31 ESBL-positive *Salmonella* (representative mix of serovars reflecting differences in local epidemiology) isolated from human clinical samples from Belgium (Table S1, available in the online version of this article) were included. For *Shigella* there were 3 isolates in 2013, 1 isolate in 2014, 19 isolates in 2015, 6 isolates in 2016, 28 isolates in 2017 and 43 isolates in 2018. For *Salmonella* there were 13 isolates in 2016, 12 isolates in 2017 and 6 isolates in 2018. Table S1 describes the characteristics and metadata of these isolates. The isolates were cultured on MH medium overnight at 37 °C. The total genomic content (chromosome and plasmid) was extracted from 10 ml of culture using the MagCore Genomic DNA Bacterial Kit according to the manufacturer’s instructions (RBCBioscience).

*Shigella* species were identified using Triple Sugar Iron Agar (TSI; Biotrading) and serotyped by slide agglutination using commercially available monovalent antisera (Denka Seiken). *Salmonella enterica* strains were subjected to serotyping by slide agglutination, according to the Kauffmann-White scheme (Grimond PAD, Weill KX. Antigenic formulae of the *Salmonella* serovars. ninth edition, 2007) [50].

### Antimicrobial susceptibility

Antibiotic susceptibility was assessed using broth microdilution, with the commercially available Sensititre EU Surveillance Salmonella/E. coli EUVSEC Plate (Thermo Fisher Scientific) according to the manufacturer’s instructions.

### Identification of ESBL-positive isolates

A multiplex PCR-based assay for the detection of β-lactamases in Gram-negative bacilli, including ESBLs (CTX-M families and subtypes, ESBL and non-ESBL SHV- and TEM-likes, OXA-1/2/7-likes, PER, VEB, GES), plasmid-mediated cephalosporinases (CMY, MOX, FOX, ACC, DHA, MIR/ACT) and carbapenemases (OXA-48, NDM, KPC, VIM, IMP), was performed as previously described [43].

### Whole genome sequencing

WGS was done with similar methods as in Berbers *et al*. [49]. Briefly, short-read sequencing libraries were prepared for all isolates included in the study with an Illumina Nextera XT DNA Library Preparation Kit and sequenced on an Illumina MiSeq instrument with a 250 bp paired-end protocol (MiSeq v3 chemistry) according to the manufacturer’s instructions. Trimming of the short reads was performed with Trimmomatic (version 0.32) [51]. First, the Illuminaclip option was used to remove the Nextera adapter sequences. Then, a sliding window approach of four bases and trimming when the Phred score dropped below 30 was employed. Lastly, the leading and trailing bases of a read were removed when the Phred score dropped below 3. All reads that were smaller than 50 bp were removed.

For 20 *Shigella* and six *Salmonella* isolates, the MinION long-read sequencing libraries were prepared by using the 1D ligation sequencing kit (SQK-LSK109; Oxford Nanopore) according to the manufacturer’s protocol for genomic DNA with barcoding with 12 isolates per flowcell (EXP-NBD104; Oxford Nanopore). From each isolate, 1 µg of DNA was used at the start of the protocol. The optional step of shearing the DNA to 8 kb fragments with Covaris G tubes was not performed. The sequencing was carried out on a R9.4.1 flow cell (Oxford Nanopore) and sequenced for 72 h.

Basecalling and demultiplexing of the Nanopore sequences was performed with Guppy (3.2.4). Then, all Nanopore reads with an average quality score <7 or a length <1000 bp were removed with NanoFilt [52]. For the output of the sequencing runs and the theoretical coverage of each sample, see Table S2. The statistics of the Illumina reads were determined with FastQC [53], and those of the Nanopore reads were determined with NanoStat [52]. Raw sequencing data and the *de novo* assemblies were submitted to the NCBI Sequence Read Archive (SRA) [54] and NCBI GenBank [55] under accession numbers PRJNA846333.

### Bioinformatic analysis

*De novo* short-read assemblies were done with SPAdes (v3.13.0) at default parameters, while *de novo* hybrid assemblies were done with Unicycler (version v0.4.8) at default parameters. To check for misassemblies, we used read mappings for specific regions of interest such as the chromosomal integrations of *bla*_CTX-M_. Reconstructed plasmids were retrieved from the hybrid assemblies. The reconstructed plasmids were identified based on if it was a circular contig and comparisons to databases such as NCBI nt, ResFinder (version 4.1) and PlasmidFinder (version 2.1). ResFinder (version 4.1) was used to determine genes responsible for the genotypic AMR resistance. PlasmidFinder (version 2.1) was used for the detection of plasmid replicons. ISfinder [56] was used to detect transposase genes. These three databases were queried using the web-tools which are based on blast, applied to the assemblies. Mlplasmid (v2.1.0) [57], Plasflow (version 1.1) [58] and MOB-suite (version 3.0.3) [59], and blast with the NCBI database were used to predict the origin (chromosome or plasmid) of short-read contigs. For Mlplasmid the option *E. coli* was used on the *Shigella* isolates due to the high similarity of these species, while with Plasflow the option *Enterobacteriaceae* was used for all isolates. For local alignments blast (v2.11.0) [60] was used, while for global alignments MauveProgressive (v 2.4.0) [61] was used. Genomes were annotated with Prokka (v1.14) [62], and these annotations were also used to determine the ORFs of genes. The visualization of these annotations was done with BRIG (v0.95) [63]. The sequence type (ST) was determined by multi-locus sequence typing MLST using blast on assemblies with the Warwick and classic scheme from Enterobase [64] for *Shigella* and *Salmonella *. The genotype of *Shigella* was determined with Mykrobe [65] and the output was then parsed with the sonneityping script (https://github.com/katholt/sonneityping).

### Phylogenomic trees

For the MLST tree the plasmid scheme from pubMLST [66] was used with the whole genome short-read assemblies as input. The MLST profiles of the isolates were then processed by creating an allele matrix by combining the typing output across all isolates, retaining only perfect hits (i.e. 100 % covered and 100 % nucleotide identity). A distance matrix was then constructed through pair-wise comparison of all isolates, with the distance defined as the number of different allele identifiers divided by the number of shared allele identifiers. The phylogeny was then reconstructed using the neighbour-joining method of the TreeConstruction Biopython module [67, 68].

To generate a plasmid alignment phylogenetic tree, assemblies which only or mostly consisted of the plasmid sequences of all 100 *Shigella* isolates were needed. For this all short reads were mapped with BBmap [69] (global aligner, with slow option enabled) to only the reconstructed *Shigella* ESBL plasmids from the hybrid assemblies. Due to the selection process based on short-read data, these reconstructed plasmids were representative of the collection of isolates used in this study. Therefore the mapped reads should contain most of the ESBL plasmid sequences of the collection. The mapped MiSeq reads were extracted with BBmap and then used for a *de novo* SPAdes [70] assembly. Subsequently, the resulting assemblies were aligned with MauveProgressive [61], which also generates a neighbour-joining tree based on these alignments in newick format.

Visualization of the phylogenetic trees was done with GrapeTree [71] and the visualization of the annotated phylogenetic tree was done with Interactive Tree Of Life (iTOL) [72].

## Results and discussion

### Selection of ESBL-producing *Shigella* and *Salmonella* isolates

Between 2013 and 2018, the Belgian NRCSS received 2315 *Shigella* isolates. With antibiotic susceptibility tests, it was determined that 113 isolates (~5 %) displayed reduced susceptibility to indicator cephalosporins [minimum inhibitory concentration (MIC) ≥4 µg ml^−1^]. Most of the isolates were identified as *S. sonnei *, while five were *S. flexneri* and two were *S. boydii *. The NRCSS noted a sudden increase in the prevalence of ESBL-positive human *Shigella* isolates in Belgium from 2017 onwards. Moreover, in-house genotyping assays [43] indicated that a shift in ESBL genes occurred, with the *bla_CTX-M-15_* gene becoming the most prevalent in the complete collection after 2017 (Fig S1). Therefore, from the 100 isolates that were included in this study (the most recent 100 of 2013–2018 consecutively taken), the highest proportion dates from after 2017.

In contrast, in our study period we observed only sporadic cases of ESBL producing *Salmonella* spp. In the timeframe covering the period of the rise in ESBL resistance seen in *Shigella *, 94 phenotypic ESBL-positive clinical *Salmonella enterica* subsp. *enterica* isolates were received by the NRCSS (out of a total of 6258 *Salmonella* isolates). On average 1.5 % of *Salmonella* isolates annually received during this period were resistant to indicator cephalosporins. As such, in contrast to *Shigella *, no rise in ESBL-resistance was observed. Therefore, a representative mix of ESBL-producing serovars (reflecting differences in local epidemiology) were selected for this study, i.e. Newport (*n*=2), Typhimurium (*n*=11), Monophasic (*n*=5), Infantis (*n*=7), Agona (*n*=1), Enteritidis (*n*=2), Rissen (*n*=1), Dublin (*n*=1) and Typhi (*n*=1).

### AMR and plasmid profile based on short-read sequencing

**Fig. 1.**
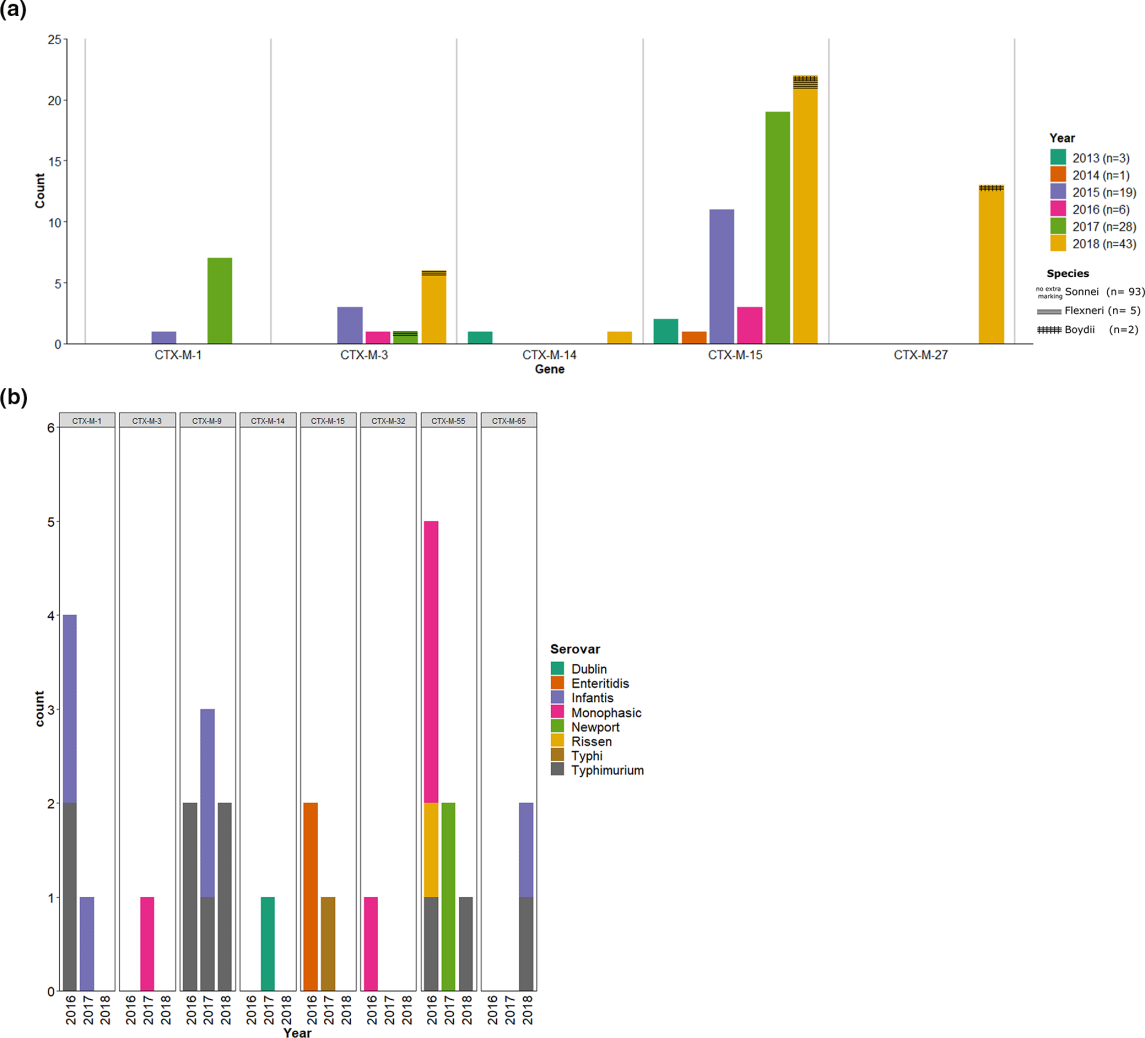
ESBL genes determined from short-read assemblies of 100 *Shigella* (**a**) and 31 *Salmonella* (**b**) isolates included in this study.

Short-read sequencing was performed on the selected isolates (Table S1). The resulting assemblies were highly fragmented with an average of 689 contigs per *Shigella* isolate and 194 contigs per *Salmonella* isolate (Table S2). Most *Shigella* isolates were of ST 152 (based on MLST). However, up to 12 different genotype profiles (based on the scheme from Mykrobe [65]) were detected (Table S1). The *Salmonella* isolates had more sequence diversity, because 10 different STs were detected (a different ST per serovar and the more prevalent serovars contained multiple STs) (Table S1). Due to the many short contigs, it proved difficult to indicate with certainty whether the ESBL genes were located on the chromosome or on a plasmid. Nevertheless, the AMR profile (Fig. 1, Tables S3 and S4) and presence of plasmid replicons (Tables S5 and S6) could be determined for each isolate.

It could be confirmed that the rapid rise in ESBL-producing *Shigella* in 2017 was mostly caused by *bla*_CTX-M-15_ (Fig. 1a). In 2018, emergence of the *bla*_CTX-M-27_ gene could be detected, which was only observed with WGS due to the detection limitations of the in-house developed multiplex assay (Fig. S1). This showcases the additional benefit that with the use of WGS all known AMR genes and variants can be detected. The Col156 (93/100 isolates), Col(BS512) (92/100 isolates) and IncFII (78/100 isolates) plasmid replicons were highly prevalent in the analysed *Shigella* isolates (Table S5). However, it should be noted that the Col156 replicon has often been detected on small, high-copy number plasmids but also on the *S. sonnei* chromosome [73], and therefore its detection does not necessarily indicate the presence of a plasmid. Furthermore, most isolates contained multiple plasmid replicons, which could suggest the presence of multiple plasmids per isolate, although these replicons could also be integrated in the chromosome. Based on the isolation year and the detected plasmid replicons, there was a slight rise in plasmid replicon IncFII in 2017 and in 2018 40/43 isolates contained IncFII (Table S5). In 47/100 cases, the ESBL gene was found to be located on the same contig as the plasmid replicon (Table S5). In an attempt to further investigate the location of the ESBL genes using the short-read assemblies, several analyses were performed. The contigs containing an ESBL gene were compared to the NCBI nt database to determine if top hits were of chromosomal or plasmid origin. Moreover, a tool (MOB-suite) was used that automates this process by clustering sequences to a curated plasmid database, thereby determining whether it is likely to be a plasmid or a chromosomal sequence [59]. This tool also predicts the transferability/mobility of plasmids, where possible. Additionally, machine learning tools (mlplasmids and plasflow) were applied [57, 58]. These analyses predicted that most ESBL genes in the investigated *Shigella* isolates were localized on a plasmid (average of 73 % with all four methods, Table S7).

In contrast to the *Shigella* isolates, no apparent rise in specific ESBL genes could be detected in any year for the analysed *Salmonella* isolates even when this was determined per serovar (Fig. 1b). Also unlike in *Shigella *, rather than the *bla_CTX-M-15_* gene (3/31), the *bla_CTX-M-55_* (one SNP different from *bla_CTX-M-15_*) gene (8/31) was the most prevalent. Nor did the plasmid replicons have any similarity to what was detected in *Shigella *, because the IncHI2 plasmid replicon (10/31) was most prevalent in *Salmonella* (Table S6). Previous studies have shown a wide range of possible plasmid replicons in *Salmonella* in other countries, such as IncHI2, IncA/C, IncL/M, IncI1, IncF, IncK and IncN plasmid replicons, with IncA/I occurring both in humans and in animals [74–76]. Prediction tools showed that in *Salmonella* probably all ESBL genes were localized on plasmids, but only for isolate i104 was the ESBL gene on the same contig as the plasmid replicon.

### Characteristics of reconstructed ESBL plasmids of *Shigella* and *Salmonella*

To verify the assumptions on the ESBL gene locations and to fully reconstruct the plasmids, additional long-read sequencing was necessary. Twenty *Shigella* and six *Salmonella* isolates were selected based on a combination of isolation year (one from 2013, one from 2014, four from 2015, four from 2016, ten from 2017 and six from 2018), unique AMR genes or plasmid replicons, probable location of the ESBL gene (chromosome or plasmid) or whether the ESBL contigs contained high similarity (95–100 % identity and coverage) using blast to other isolates from the collection (possibly indicating a similar/common ESBL plasmid). Moreover, five *Shigella* isolates were selected solely for the presence of *bla*_CTX-M-15_ as this was the gene found to have increased in prevalence in 2017 (see Table S1 for the reason for inclusion for long-read sequencing per selected isolate).

Hybrid assemblies of the 20 *Shigella* isolates resulted in successfully reconstructed chromosomes and ESBL plasmids (Table 1) for 19/20 isolates. One *Shigella* ESBL plasmid was still fragmented into multiple contigs (i27). The fragmented ESBL plasmid was omitted from subsequent analyses.

**Table 1.** Description of the reconstructed ESBL plasmids from *Shigella* isolates

Isolate	Code isolate	Code plasmid	Year	Size of plasmid (kb)	ESBL gene	Other AMR genes†	Plasmid replicon	Transposon gene 5′ of ESBL gene	Transposon gene 3′ of ESBL gene
S13BD04362	i3	p3	2013	98	*bla*_CTX-M-14_	–	IncI1-I(Gamma)	–	**IS1380 family transposase ISEcp1**
S14BD05406	i4	p4	2014	94	*bla*_CTX-M-15_	*aac(3)-Iid, bla*_TEM-1B_ *,*	IncI1-I(Gamma)	IS6 family transposase IS26	**IS6 family transposase IS26**
S15BD02407	i5	p5	2015	96	*bla*_CTX-M-15_	*qnrS1*	IncB/O/K/Z	Tn3 family transposase Tn2, **IS3 family transposase ISEc36**	–
S15BD06969	i9	p9	2015	112	*bla*_CTX-M-1_	*aadA5, bla*_TEM-1B_ *, sul2, dfrA17*	IncI1-I(Gamma)	**IS5 family transposase ISKpn26**	–
S15BD07413	i12	p12	2015	90	*bla*_CTX-M-15_	*bla*_TEM-1B_	IncI1-I(Gamma)	Tn3 family transposase Tn2	**IS1380 family transposase ISEcp1**
S15BD09164	i20	p20	2015	86	*bla*_CTX-M-55_	–	IncI1-I(Gamma)	**IS1380 family transposase ISEcp1**	–
S17BD00672*	i31	p31	2017	86	*bla*_CTX-M-3_	–	IncI1-I(Gamma)	–	–
S17BD04134	i34	p34	2017	65	*bla*_CTX-M-15_	–	IncFII	Tn3 family transposase Tn2	**IS6 family transposase IS15**
S17BD05200	i36	–	2017	Chromosomal	*bla*_CTX-M-15_	*aac(3)-Iid, aadA2, bla*_TEM-1B_ *, mdf(A), mph(A), sul1, dfrA12, dfrA1*	Col156	ISNCY family transposase ISSen7	**IS1380 family transposase ISEcp1**
S17BD05916	i38	p38	2017	111	*bla*_CTX-M-15_	–	IncFIB	–	**IS1380 family transposase ISEcp1**
S17BD05944	i39	p39	2017	92	*bla*_CTX-M-15_	*bla*_TEM-1B_	IncB/O/K/Z	Tn3 family transposase Tn2	**IS1380 family transposase ISEcp1**
S17BD06357	i41	–	2017	Chromosomal	*bla*_CTX-M-15_	*mdf(A), dfrA1*	Col156	Tn3 family transposase Tn2	**IS1380 family transposase ISEcp1**
S17BD08179	i54	p54	2017	89	*bla*_CTX-M-15_	*mph(A), qnrS1, dfrA12*	IncFII	Tn3 family transposase Tn2	**IS6 family transposase IS15**
S17BD08233	i55	p55	2017	108	*bla*_CTX-M-1_	*sul2, tet(A*)	IncI1-I(Gamma)	–	**IS1380 family transposase ISEcp1**
S18BD01106	i59	p59	2017	89	*bla*_CTX-M-15_	*aadA5, mph(A), sul1, qnrS1, dfrA17*	IncFII	Tn3 family transposase Tn2	**IS6 family transposase IS15**
S18BD03178	i67	p67	2018	78	*bla*_CTX-M-27_	*erm(B), mph(A*)	IncFII	IS6 family transposase IS26	**IS6 family transposase IS15**
S18BD03411	i69	p69	2018	111	*bla*_CTX-M-14_	*aac(3)-Iid, aadA5, mph(A), sul1, dfrA17*	IncB/O/K/Z	–	**IS1380 family transposase ISEcp1**
S18BD04295	i73	p73	2018	101	*bla*_CTX-M-15_	*qnrS1*	IncB/O/K/Z	Tn3 family transposase Tn2, **IS3 family transposase ISEc36**	–
S18BD05612	i83	p83	2018	78	*bla*_CTX-M-15_	*qnrS1*	IncFII	Tn3 family transposase Tn2	**IS6 family transposase IS15**

In bold are the transposases that are probably responsible for the integration of the ESBL gene in the plasmid or chromosome based on the directionality and proximity to the ESBL gene of the transposases.

*All isolates are *Shigella sonnei* except for the one with an asterisk, which is *Shigella flexneri*.

†Other AMR genes on the same plasmid as the ESBL gene; except for isolates i36 and i41 (chromosomal ESBL gene), this column shows the other AMR genes on the chromosome.

From the hybrid assemblies it was determined that in 17/19 *Shigella* and 6/6 *Salmonella* isolates, the ESBL gene was located on a plasmid. Similarly as was determined with PlasmidFinder (Tables S5 and S6), 24/25 of these isolates (all except i127) contained 1–10 plasmids (average=5.2) besides the ESBL plasmid. In 9/19 of the isolates, the *Shigella* virulence plasmid pINV could be detected but none of them contained an ESBL gene. The non-ESBL plasmids also contained other AMR genes such as *aac(3)-Iid, bla*_TEM-1B_ *, qnrS1, aadA2, aadA5, sul1, sul2, dfrA1, dfrA12, dfrA17, tet(A), mdf(A), mph(A*) and *erm(B*). However, we mainly focused on the ESBL genes given the aim of the study. In Tables 1 and 2, a summary is shown of the plasmid size, ESBL gene on every plasmid, other AMR genes, the plasmid replicon and transposase genes located in proximity (~1–300 bp) to the ESBL gene, for *Shigella* and *Salmonella *, respectively.

**Table 2.** Description of the reconstructed ESBL plasmids from *Salmonella* isolates

Isolate	Code isolate	Code plasmid	Year isolate	Size of plasmid (kb)	ESBL gene	Other AMR genes	Plasmid replicon	Transposon gene 5′ side of ESBL gene	Transposon gene 3′ side of ESBL gene
S16BD03602	i105	p105	2016	52	*bla*_CTX-M-15_	–	IncFII	–	**IS1380 family transposase ISEcp1**
S16BD04351	i106	p106	2016	124	*bla*_CTX-M-15_	*blaTEM-1B, blaOXA-1, mph(A*)	IncFIA	IS6 family transposase IS26	**IS6 family transposase IS26**
S16BD07964	i114	p114	2016	128	*bla*_CTX-M-32_	*tet(B), sul2, aph(3'')-Ib, aph(3'')-Ib, aph(6)-Id*	IncFIA	**IS1380 family transposase ISEcp1** IS5 family transposase ISKpn26	IS66 family transposase ISEc43
S17BD06931	i123	p123	2017	287	*bla*_CTX-M-9_	*mcr-9, blaTEM-1B, sul1, floR, ant(2'')-Ia, aadA2b, aph(6)-Id, aph(3'')-Ib, tet(A), dfrA16, qnrA1*	IncHI2	**IS6 family transposase IS26**	Tn3 family transposase IS3000
S18BD02037	i127	p127	2018	318	*bla*_CTX-M-65_	*tet(A), floR, sul1, dfrA14, aadA1, aph(4)-Ia, aph(3')-Ia, aac(3)-IV*	IncFIB(pN55391)	**IS6 family transposase IS15**	IS5 family transposase IS903
S18BD07928	i131	p131	2018	281	*bla*_CTX-M-9_	*mcr-9, aadA2b, ant(2'')-Ia, dfrA16, sul1, tet(A), tet(A), catA1*	IncHI2	**Tn3 family transposase IS3000**	IS6 family transposase IS26

In bold are the transposases that are probably responsible for the integration of the ESBL gene in the plasmid or chromosome based on the directionality and proximity to the ESBL gene of the transposases.

**Fig. 2.**
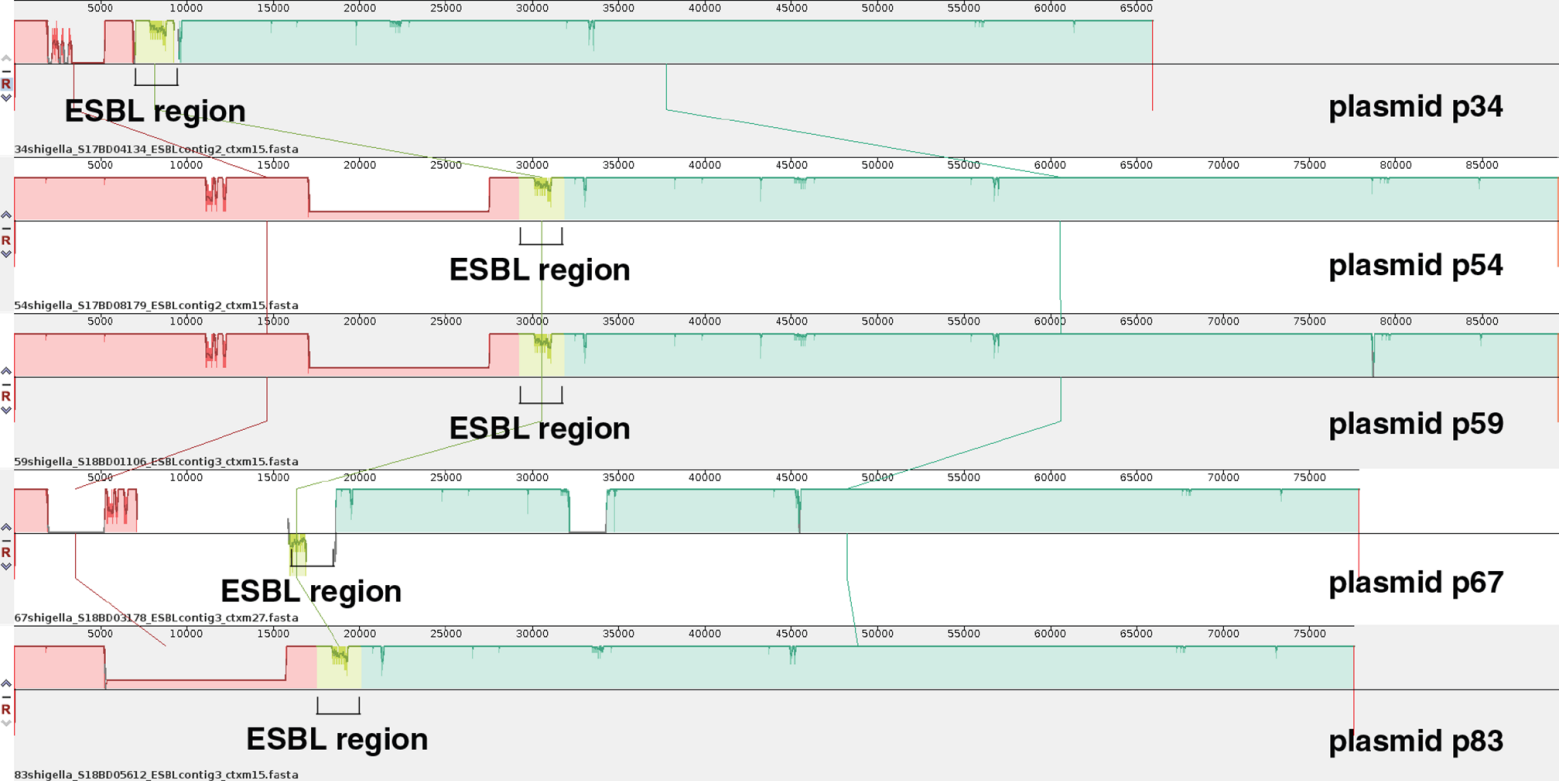
ProgressiveMauve alignment of the IncFII plasmids reconstructed with hybrid assemblies. The colours indicate locally collinear blocks (LCBs), which are homologous regions without rearrangements between two or more sequences. The p-numbers are the ESBL plasmids of the corresponding i-number isolates.

The size of the *Shigella* ESBL plasmids ranged from 65 to 112 kb. Between reconstructed plasmids with the same plasmid replicon there was a high similarity (average of 77 % coverage and 98 % identity) over the entire structure. The plasmids with the IncFII replicon showed higher similarity amongst each other compared to the other plasmid replicons (Figs 2 and S2 a, b). Isolates i54 and i59 with an IncFII replicon even had almost identical ESBL plasmids (90 bp difference, 100 % identity and 99 % coverage), while others had a similar plasmid backbone (average of 85 % coverage and 99 % identity). The plasmids with the IncFII replicon (in 78/100 isolates) were also the plasmids that often contained the *bla*_CTX-M-15_ genes (49/78 isolates). However, there were also some relevant differences between the plasmids such as p67 containing a different *bla_CTX-M_* variant and *erm(B*) gene, while both p54 and p59 harboured *sul1* and *dfrA17* AMR genes in a unique region. Within *Enterobacteriaceae *, IncFII has been reported multiple times to be associated with *bla_CTX-M-15_* in other countries [77, 78].

In *Salmonella* the ESBL plasmids ranged from 52 to 318 kb. Of the six reconstructed *Salmonella* ESBL plasmids, two plasmid replicons were unique (i.e. IncFII and IncFIB replicon) and of plasmids with replicons IncFIA and IncHI2 there were two of each present. The ESBL plasmids with unique replicons (p105 and p127) contained considerable variance compared to each other (only a 263 bp match, and ~0 % coverage). The two IncFIA ESBL plasmids displayed a low level of similarity in their alignments (55 % coverage and 99 % identity). The two IncHI2 ESBL plasmids seemed to have similar size and genetic content, but upon closer inspection only the backbone was identical (Fig. S2c, d).

We found that the *Shigella* isolates i4, i5, i9, i12, i39, i54, i55, i59, i67, i69, i73 and i83 contain ESBL plasmids with MDR conferring resistance to antibiotics in the aminoglycoside, beta-lactam, macrolide, quinolone, sulphonamide, tetracycline and trimethoprim classes. The plasmid-mediated *qnrS* gene, encoding ciprofloxacin resistance (quinolone class), has been found to be often co-associated with genes coding for ESBLs [79]. In isolates i5, i54, i73 and i83 the *qnrS* gene occurred on the same plasmid as the *bla_CTX-M_* gene. Ciprofloxacin resistance is not only limited to the mobile *qnrS* gene. In Belgium in similar years as for this study, chromosomal mutations in the *Shigella gyrA* and *parC* have been reported to contribute to rising ciprofloxacin resistance [44]. In the ESBL-positive *Shigella* isolates of this study, 51/100 had a point mutation in *gyrA* and/or *parC*, with *gyrA* p.S83L and p.D87G and *parC* p.S80I being the most common mutations (Table S9). Worldwide up to half of *Shigella* strains are MDR [80], which can greatly complicate treatment in vulnerable patients. When multiple AMR genes are located on a single plasmid, antibiotic stewardship will become even more difficult as reducing usage of one type of antibiotic class is not sufficient to reduce resistance towards all antibiotics for which these AMR genes confer resistance and are located on the same plasmid, i.e. selective pressure causes the plasmid to remain in the bacterial population.

Similarly, all *Salmonella* ESBL plasmids except for the smaller 52 kb ESBL plasmid (p105) contained multi-resistance to antibiotics of several classes (Table 2). Of concern are the multi-resistant plasmids of ~300 kb (p123, p127 and p131). Two of them (p123 and p131) even contained intact *mcr-9* genes, which confer resistance to the last resort antibiotic colistin. However, broth microdilution tests (0.5–8 µg ml^−1^) determined that these *mcr-9* genes did not confer phenotypic resistance to colistin (data not shown). Moreover, colistin is never used in clinical treatment of *Shigella* or *Salmonella *, so the presence of this gene is less worrisome. Even so, the presence of the *mcr-9* genes is still relevant for their potential spread to other pathogens that are treated with colistin [81].

**Fig. 3.**
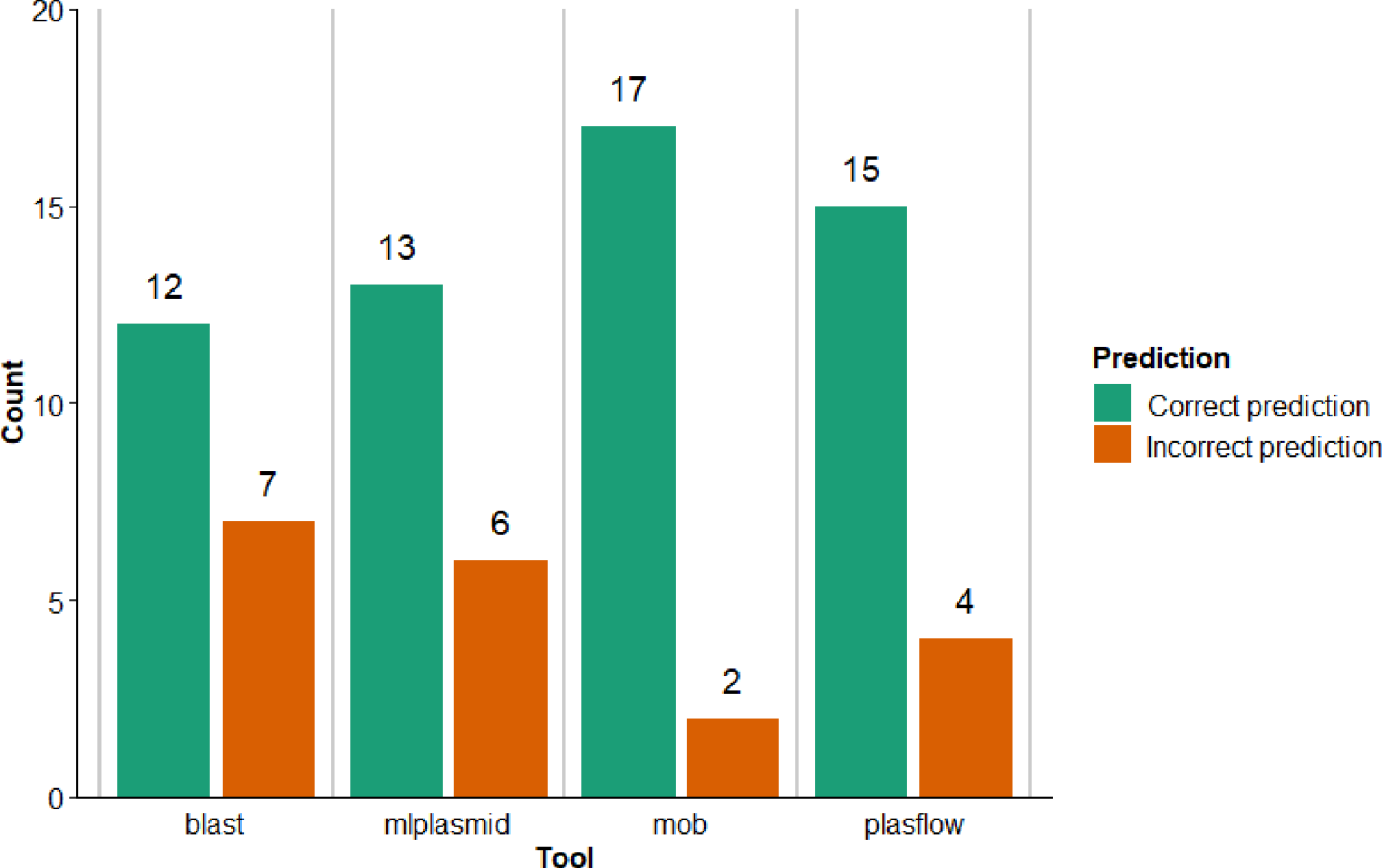
Accuracy of prediction by blast using the NCBI nt database, Mlplasmid, MOB-suite and plasflow on the *Shigella* hybrid assemblies. A prediction was considered correct when Mlplasmid and plasflow indicated with at least 70 % certainty that the ESBL contig was part of the correct structure as confirmed by the hybrid assemblies, for blast if >70 % of hits out of 100 matched it was considered a correct prediction and mob-suite did not give certainty values so the output was used as such.

Most ESBL genes in *Shigella* have the IS1380 family transposase ISEcp1 and/or Tn3 family transposase Tn2 in close proximity, with ISEcp1 probably being responsible for the ESBL gene integration in this plasmid due to the orientation of the transposase gene and short distance (~50 bp) to the *bla_CTX-M_* genes [34]. Plasmids p12, p34 and p39 (Figs 3 and S3) have a high density of transposase genes surrounding the ESBL gene. These closely located transposase genes could indicate multiple transposition events in this location, which might indicate that these isolates or the previous plasmid’s host had to adapt to changing environments as transposases have a defensive function for bacteria [82]. Although the smaller sample size impedes making a general conclusion, in *Salmonella* there were several transposases only detected once, but the IS1380 family transposase ISEcp1 and/or Tn3 family transposase Tn2 in these isolates were also the most prominent. In the short-read assemblies, there were 42/100 *Shigella* and 15/31 *Salmonella* isolates where the ESBL contig did not contain a transposase (Table S8). By manually comparing the short-read assemblies and comparing them to the hybrid assemblies, it could be determined that in some cases, absence of a transposase gene on the ESBL contig was due to the very fragmented short-read assemblies.

### Evaluation of plasmid prediction tools

**Fig. 4.**
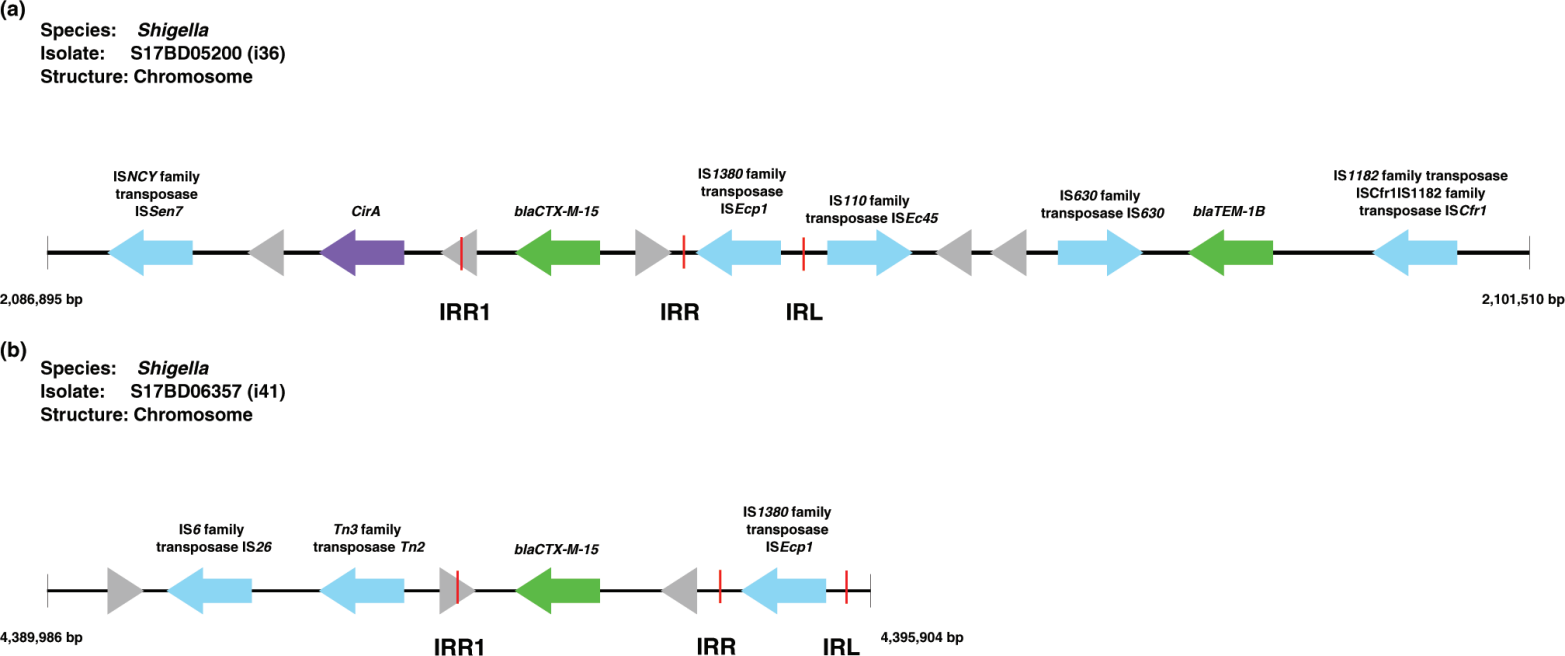
Context of the ESBL genes (green), transposases (blue), hypothetical genes (grey) and other genes (purple) for chromosomal integration of ESBL genes in *Shigella* S17BD05200 (**i36**) and S17BD06357 (**i41**). The direction of the arrow displays the orientation of the genes. For i41 the closest gene is *yhbX* (not shown). Red bars indicate the locations of the inverted repeat right (IRR) and inverted repeat left (IRL).

By having for some isolates both short-read assemblies and hybrid assemblies, it gave an opportunity to check how accurate the applied plasmid prediction tools were in determining the correct location of ESBL genes in *Enterobacteriaceae* (chromosome or plasmid). For this we used the short-read assemblies of *Shigella *, because more of them were used for hybrid assemblies. MOB-suite was the most accurate with 17 correctly and two incorrectly predicted locations of the ESBL gene (Fig. 4). These two incorrect predictions indicated a chromosome where it should have been a plasmid (isolate i3 with a contig of 48 kb and i67 with a contig of 2.5 kb). These two plasmid locations were correctly predicted by Plasflow, but this latter tool incorrectly predicted the location of the two ESBL genes we observed to be integrated in the chromosome (i36 with a contig of 17 kb and i41 with a contig of 32 kb). Mlplasmid’s performance was similar to blast, but Mlplasmid is optimized/trained with *E. coli* isolates and while these are very similar (up to 99 %) in sequence to *Shigella *, this could explain the lower performance. Moreover, with all methods there were more false or unclassified/uncertain predictions for isolates with very short ESBL contigs (<3 kb in size). Surprisingly, for a few longer contigs (>10 kb) there were also conflicting results between the prediction tools (Table S7). Therefore, plasmid prediction tools can be useful to quickly determine whether a gene is probably located on a plasmid, but ultimately they are limited by their training datasets and quality or length of the analysed sequence. Nevertheless, in the 100 *Shigella* isolates all prediction tools predicted that the ESBL genes were mainly located on a plasmid (Table S7). This seems to be in accordance with previous studies where up to 90 % accuracy in predicting plasmids by such tools was shown [83]. This high concordance for *Shigella* is of particular interest, as Plasflow and Mlplasmid were trained with *E. coli* data.

### Integration of ESBL genes in the chromosome of *Shigella*

In two *S. sonnei* isolates (i36 and i41, Fig. 4), using the hybrid assemblies, it was possible to detect an integration of the ESBL gene (*bla*_CTX-M-15_) in the chromosome of ~2700 and ~2400 bp in size, near the *cirA* and *yhbX* genes respectively. This ESBL gene integration is a novel finding in *S. sonnei *. The ESBL integration (including surrounding regions as depicted in Fig. 4) in *Shigella* i41 has up to 93 % similarity to the *Klebsiella* and *E. coli* chromosome and plasmids, while for i36 a similar integration (100 % similarity with ESBL gene and flanking regions, accession number CP050202.1) was described before in an *E. coli* chromosome [84].

Both integrations were associated with the ISEcp1 transposase, but they are both integrated in a different region of the *Shigella* chromosome.

When the integrations were aligned to each other and to the ISFinder database (Fig. S4) [56], it was possible to determine that in i36 the transposon including the IRL, IRR and coding sequence (CDS) of ISEcp1 is conserved perfectly (1656 bp), while i41 has a homologous match for 1193/1656 bp with mismatches/mutations in the 5′ region including the IRL. Downstream of the IRR, the two insertions are similar with only 2 bp mismatch in the IRR1 between each other, but neither isolate has an IRR1 that perfectly matches the 14 bp sequence described in the databases. Further transposition events are more likely to occur for i36 than for i41, because of the better conservation of the IRL. While transposons are able to contain multiple genes, based on the alignments it seems that here they both only harboured *bla*_CTX-M-15_ [85].

ISEcp1 transposases are most commonly associated with ESBL genes in *Enterobacteriaceae* [34]. To our knowledge, the complete sequence of an ESBL integration had not been described before in *Shigella *. In previous studies, conjugation experiments have determined that an ESBL gene could have been chromosomally located in *Shigella *, but it was not certain which ESBL gene was detected and where it was integrated in the chromosome [22, 86, 87]. This type of integration, in contrast to a plasmidic location, could lead to more stable expression of ESBL genes (depending on the conservation of the IRL/IRR) in *Shigella* as the gene is less likely to be lost even when there is less antibiotic selection pressure [8–10]. Therefore, the discovery of chromosomal integrations is important for the further surveillance of *Shigella *, as this chromosomal context enables their vertical, stable transmission in clinically important lineages, which should be further monitored.

We did not find confirmation of similar integrations in the chromosome in other *Shigella* isolates included in this study based on the mapping of sequencing data. Indeed, for 47/100 analysed *Shigella* isolates in this study, it is likely that the ESBL gene is on a plasmid, because the plasmid replicon is on the same contig as the ESBL gene. This was increased to 95/100 isolates with ESBL genes on a plasmid and 2/100 ESBL genes on the chromosome if we take into consideration the hybrid assemblies and mapping results (>97 % mapping to reconstructed plasmids, Table S10). For the three remaining isolates (i1, i32 and i100), the location of the ESBL gene is less clear. For two of those (i1 and i100), the ESBL genes were predicted to be located on a plasmid and the ESBL gene of i32 on a chromosome (with the exception of Plasflow that predicted the ESBL gene of i32 to be on a plasmid). As shown above there is a possibility of a false prediction with these tools, but not knowing the exact location of the ESBL gene in these isolates did not impact answering our research questions. For *Salmonella* there was no indication in any of the 31 isolates that the ESBL gene was located on the chromosome.

### Comparison of reconstructed ESBL plasmids to international databases

By comparing the sequences of the reconstructed plasmids to those present in the international NCBI nt database (summarized in Tables 3 and 4), we could illustrate some take-home messages related to surveillance of plasmids in public health. For example, this comparison allows us to determine whether the reconstructed plasmids had been detected before in Belgium or in any other country. For the *Shigella* isolates, some plasmids had very high sequence similarity to entries of the international database, such as plasmid p3 that showed 99 % similarity and coverage to a plasmid detected in Japan (MK764028.2) [88]. Also for p69 and p83, this high sequence similarity with previously described plasmids was found. Similarly, for the *Salmonella* reconstructed ESBL plasmids, there was up to 99 % coverage found in international sequences (Table 4). In contrast to all other reconstructed *Salmonella* plasmids, p106 had a chromosomal sequence as the best match in the NCBI nt database. However, the second to tenth best matching sequences were all plasmids (Table S32) just as for the other isolates (Tables S14–S36). Therefore this chromosomal sequence (CP050205.1) probably contains an integrated plasmid sequence or was falsely categorized as chromosomal, illustrating the potential issue of (miss)classification in public databases.

**Table 3.** Comparison of all reconstructed ESBL plasmids from *Shigella* to their most similar plasmid sequences from the NCBI nt database

Query	Travel	Highest scoring match	Accession no. highest scoring match	Isolation date highest scoring match	Country of origin	Query coverage (%)	Identity (%)	Genetic difference (based on global alignment)
p3	–	* Escherichia coli* strain 80DF1 plasmid p80DF1, complete sequence	MK764028.2	2016	Japan	99.00	99.95	No rearrangements and only differences at SNP level in genes encoding hypothetical proteins
p4	–	* Salmonella enterica* subsp. *enterica* serovar Typhi strain 311189_268103 plasmid pk88, complete sequence	CP029903.1	2007	Brazil	88.00	98.50	Query and subject both missing several 1–2 kb regions. Subject also missing the ***aac(3)-Iid*** gene
p5	–	* Escherichia coli* plasmid pWP8-S18-ESBL-07_2 DNA, complete genome, strain: WP8-S18-ESBL-07	AP022263.1	2018	Japan	95.00	100.00	Subject is missing a 5 kb region
p9	–	* Escherichia coli* plasmid pCOV10 clone COV10_c1	MG648909.1	2017	France	82.00	100.00	Subject is missing a 3 kb region
p12	–	* Salmonella enterica* subsp. *enterica* serovar Typhi strain 311189_268103 plasmid pk88	CP029903.1	2007	Brazil	98.00	99.98	Subject is missing a 1.5 kb region
p20	Thailand	* Klebsiella pneumoniae* strain 628 plasmid p628-CTXM, complete sequence	KP987217.1	2015	China	97.00	99.79	Subject is missing a 2 kb and 1 kb region
p31	–	* Shigella sonnei* strain 19.0822.3296 plasmid p19-0822-3296, complete sequence	CP049184.1	2019	Switzerland	98.00	99.98	Query is missing a 1.2 kb and 1 kb region, while subject is missing a 1.4 kb region
p34	Iran	* Escherichia coli* plasmid p37, complete sequence	MT077885.1	2020	USA	94.00	99.97	Query is missing several regions containing ***ermB***, while subject is missing a 2 kb region. Due to point mutations ***blaCTX-M-27*** instead of ***blaCTXM-15*** is detected in the subject
p38	–	* Escherichia coli* strain 4/0 plasmid p4_0.2, complete sequence	CP023851.1	2009	Sweden	90.00	98.82	Multiple insertions of 1–2 kb in both query and subject containing ***blaCTX-M-15***
p39	–	* Escherichia coli* strain SCEC020007 plasmid pBOKZ_020007, complete sequence	CP025625.1	2016	China	80.00	98.12	Subject is missing a 10 kb region that contains ***blaCTX-M-15*** and ***blaTEM-1B***
p54	–	* Shigella sonnei* strain 19.0821.3486 plasmid p19-0821-3486, complete sequence	CP049186.1	2019	Switzerland (travel Egypt)	88.00	99.99	Query is missing a 1 kb and 2.5 kb region, while the subject is missing a 10 kb region containing ***aadA5**, **dfrA17, mph(A)*** and ***sul1***
p55	–	* Escherichia coli* plasmid pCOV33 clone COV33_c1	MG649046.1	2017	France	98.00	99.99	Query is missing a 1 kb region, while the subject is missing a 2 kb region. The *blaCTX-M* gene is in reverse complement
p59	–	* Shigella sonnei* strain 19.0821.3486 plasmid p19-0821-3486, complete sequence	CP049186.1	2019	Switzerland (travel Egypt)	88.00	99.99	Query is missing a 1 kb and 2.5 kb region, while the subject is missing a 10 kb region containing ***aadA5**, **dfrA17, mph(A)*** and ***sul1***
p67	–	* Shigella sonnei* strain 19.0821.3486 plasmid p19-0821-3486, complete sequence	CP049168.1	2019	Switzerland (travel Egypt)	89.00	99.99	Query is missing a 0.6 kb region, while the subject is missing a 10 kb region containing ***mph(A)** and **ermB***. Due to point mutations ***blaCTX-M-27*** instead of ***blaCTXM-15*** is detected in the subject
p69	–	* Escherichia coli* strain VREC-hospital6489704 genome assembly, plasmid: 1	LR595877.1	2019	UK	100.00	99.96	Almost identical, only differences at SNP level in genes encoding hypothetical proteins and rearrangement in a small region containing genes encoding hypothetical proteins
p73	–	* Escherichia coli* plasmid pWP8-S18-ESBL-07_2 DNA, complete genome, strain: WP8-S18-ESBL-07	AP022263.1	2018	Japan	90.00	100.00	Subject is missing a 5 kb, 3 kb and another 3 kb region
p83	Egypt	* Shigella sonnei* strain 19.0821.3486 plasmid p19-0821-3486, complete sequence	CP049186.1	2019	Switzerland (travel Egypt)	100.00	99.93	Query is missing a 5 kb region

Query = sequence used as input/reference in blast, the p-numbers are the ESBL plasmids of the corresponding i-number isolates. Travel = reference isolate is from patient with recent visit to the indicated country. A dash indicates that no travel information was available for that patient. Highest scoring match = sequence from NCBI nt database with highest alignment score to the query. Country of origin = country where highest scoring match was isolated. Query coverage (%) = percentage of reference sequence that is covered by highest scoring match. Identity (%) = sequence similarity between query and highest scoring match.

**Table 4.** Comparison of all reconstructed ESBL plasmids from *Salmonella* to the most similar plasmid sequences from the NCBI nt database

Query	Travel	Highest scoring match	Accession no. highest scoring match	Isolation date highest scoring match	Country of origin	Query coverage (%)	Identity (%)	Genetic difference (based on global alignment)
p105	–	* Escherichia coli* strain PK9 plasmid pHNPK9-FOS, complete sequence	MT074415.1	2020	China	92.00	99.37	The query is missing two 1 kb and a 4 kb region containing ***fosA10***, while the subject is missing a 3 kb and 1 kb region containing ***blaCTX-M-15***
p106	–	* Escherichia coli* strain RH-048-CS chromosome	CP050205.1	2017	Bangladesh	86.00	99.01	Rearrangements and differences on SNP level
p114	–	* Escherichia col* i strain ATCC 700415 plasmid unnamed, complete sequence	CP022610.1	1992	USA	96.00	99.87	The query is missing a 14 kb and 1 kb region, while the subject is missing a region containing ***blaCTX-M-32***
p123	–	* Citrobacter freundii* strain 154 plasmid p154_1, complete sequence	CP038654.1	2014	Spain	95.00	99.99	The query is missing a 11 kb and 18 kb region containing ***sul1, aadA1, VIM1,*** and ***QnrA1***, while the subject is missing a 8 kb and 9 kb region containing ***neo, folP*** and ***tetA***
p127	–	* Salmonella enterica* subsp. *enterica* serovar Infantis strain 114 061 plasmid p114061, complete sequence	CP070303.1	2015	UK	100.00	99.99	The query is missing a 1 kb and 4 kb region containing ***FosA3***
p131	–	* Enterobacter hormaechei* strain EGYMCRVIM plasmid pMS-37a, complete sequence	CP053191.1	2017	Egypt	95.00	99.99	The query is missing a 1 kb, 6 kb and another 6 kb region containing ***ant1***, ***folP*** and ***aacA4***, while the subject is missing a 9 kb, 4 kb, 8 kb and 3 kb region containing ***blaCTX-M-9, folP, ant1*** *, **cat**, **folP*** and ***aadB***

Query = sequence used as reference/input in blast, the p-numbers are the ESBL plasmids of the corresponding i-number isolates. Travel = reference isolate is from patient with recent visit to the country. A dash indicates that no travel information was available for that patient. Highest scoring match = sequence from NCBI nt database with highest alignment score to the query. Country of origin = country where highest scoring match was isolated. Query coverage (%) = percentage of reference sequence that is covered by highest scoring match. Identity (%) = sequence similarity between query and highest scoring match.

While most entries in international databases contain metadata about the country and date of isolation, there is usually not much other metadata except when it is linked to a more in-depth study. For example, by searching for the study where plasmid p19-0821-3486 (CP049186.1) was described [47], there was additional metadata available that that the patient had also travelled to Egypt just as for the matching *Shigella* plasmid sequence (p54) in our study. This demonstrated the combined power of WGS and epidemiological data in understanding the sources and reservoirs of AMR. Therefore, a comprehensive database that tracks occurrences of plasmids and their metadata in order to determine the worldwide dissemination could be useful for (inter)national surveillance of AMR. While there already exist such initiatives that incorporate and curate the data that NCBI provides as for PLSDB [89], these still lack detailed metadata for many entries.

Interestingly, through a visualization of global alignments (Fig. S7), it became apparent that some sequences (from the NCBI nt database) had additional rearrangements or insertions that increased their size compared to the *Shigella* and *Salmonella* plasmid sequences from our study, but this was impossible to determine with only the blast output. For example, CP049186.1 had 100 % coverage and 99.93 % sequence similarity to the *Shigella* ESBL plasmid p83, but CP049186.1 had an additional transposon of around 5 kb (consisting of chaperonin, recombinase and hypothetical genes) inserted. This occurred in both *Shigella* and *Salmonella* isolates that were blasted to databases. Therefore, with the annotation and global alignment it was determined that in some isolates these differences in sequence even contained AMR genes of clinical significance, but in other isolates the differences were only in repetitive regions or hypothetical genes (Table 3).

Alternatively, a possible reason for the differences between plasmids of other studies is that many research institutions use different DNA extraction, sequencing protocols and bioinformatics approaches, which can all slightly change the final *de novo* assembly of the ESBL plasmids [90–92]. In an ideal situation, raw sequencing reads would be publicly available for all assembled sequences to be able to compare plasmids with the same assembly protocols. However, many reconstructed plasmids in this study contained deletions and insertions compared to the international isolates, which are probably not all caused by the choice of methodology. Therefore, this also illustrates the need for accurate assemblies (as obtained with hybrid assemblies), because rearrangements, duplications and deletions (especially in repetitive regions) are otherwise difficult to determine.

Some of the ESBL plasmids (Tables 3 and 4) to which similarity was found in the international database, originated from other species than the host bacterium such as *Salmonella* and *E. coli* for the *Shigella* isolates, which indicates that there was probably cross-species exchange of MGEs or plasmids. Other studies have also described this type of exchange between species, especially within the family *Enterobacteriaceae* [93, 94]. Therefore, the sequence data from *Salmonella* and *Shigella* obtained in this study were further compared to determine if this kind of exchange of ESBL gene fragments or plasmids occurred between the two clinical species. By mapping short sequencing reads from all 31 *Salmonella* isolates to the reconstructed ESBL plasmid from *Shigella* and vice versa, no perfect matches (100 % coverage and identity) between the ESBL plasmids of *Shigella* and *Salmonella* were detected (Tables S11–S13). However, some *Salmonella* ESBL plasmids (p113, p120 and p128) could have a common origin as they have up to 98 % similarity to *Shigella* ESBL plasmids (Table S11), but global alignments show structural rearrangements which makes recent exchange of these plasmids unlikely. A fragment of ~2.3 kb that includes the ESBL (*bla*_CTX-M-15_) gene and the transposase genes matched between the *Salmonella* plasmid p105 (2016), the *Shigella* plasmid p39 (2017) and the chromosome of *Shigella* isolate i41 (2017). This finding could point to a similar origin that spread to multiple species and even to the chromosome. However, it is unlikely that this exchange of a 2.3 kb region led to the rise in phenotypical ESBL detection in *Shigella,* because it was not prominently present in the isolates causative of the rise in ESBL in *Shigella *. Nevertheless, additional studies including more isolates, and also from other econiches, would be needed to confirm this.

### Similarity among all *Shigella* isolates including their ESBL plasmids

**Fig. 5.**
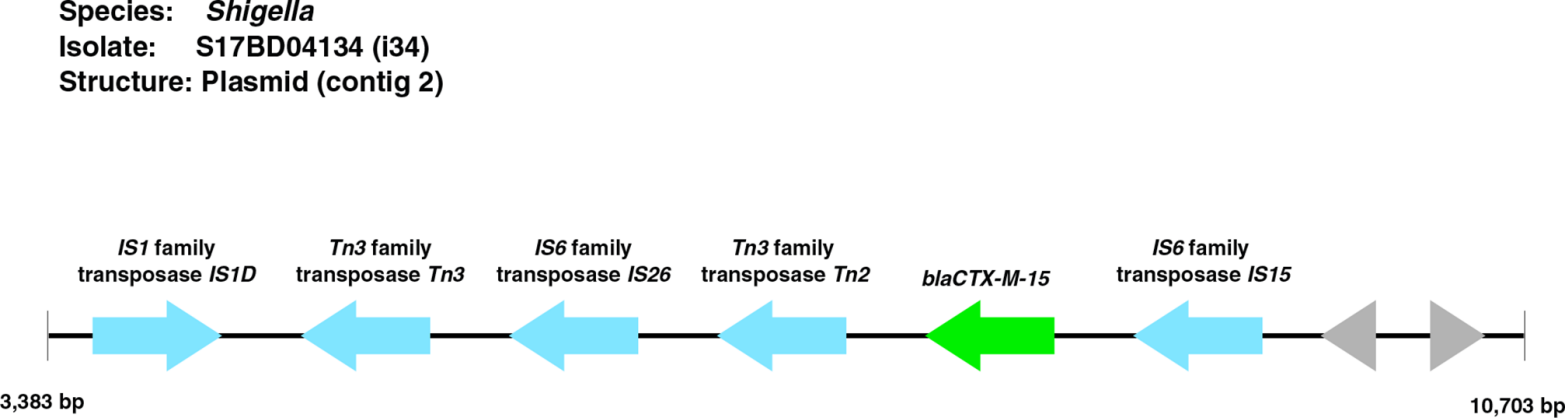
Genetic context of the ESBL gene in isolate S17BD04134 (**i34**). Each arrow represents a gene and the direction of the arrow displays the orientation of the genes. Blue=transposase, green=antimicrobial resistance gene, grey=hypothetical gene.

As elaborated upon above, in *Shigella *, a high similarity between IncFII reconstructed ESBL plasmids compared to the other plasmid replicons was found (Fig. 5). However, these hybrid assemblies were only performed on a representative selection of the ESBL-producing *Shigella* sampled in Belgium between 2013 and 2018 (for the IncFII replicon 5/78 isolates). Sequencing each isolate with short- and long-read sequencing technology is expensive and, depending on the sequence diversity within a collection, it may even be unnecessary. Therefore, an alternative approach was followed to investigate the similarity between all isolates and their plasmids included in this study.

**Fig. 6.**
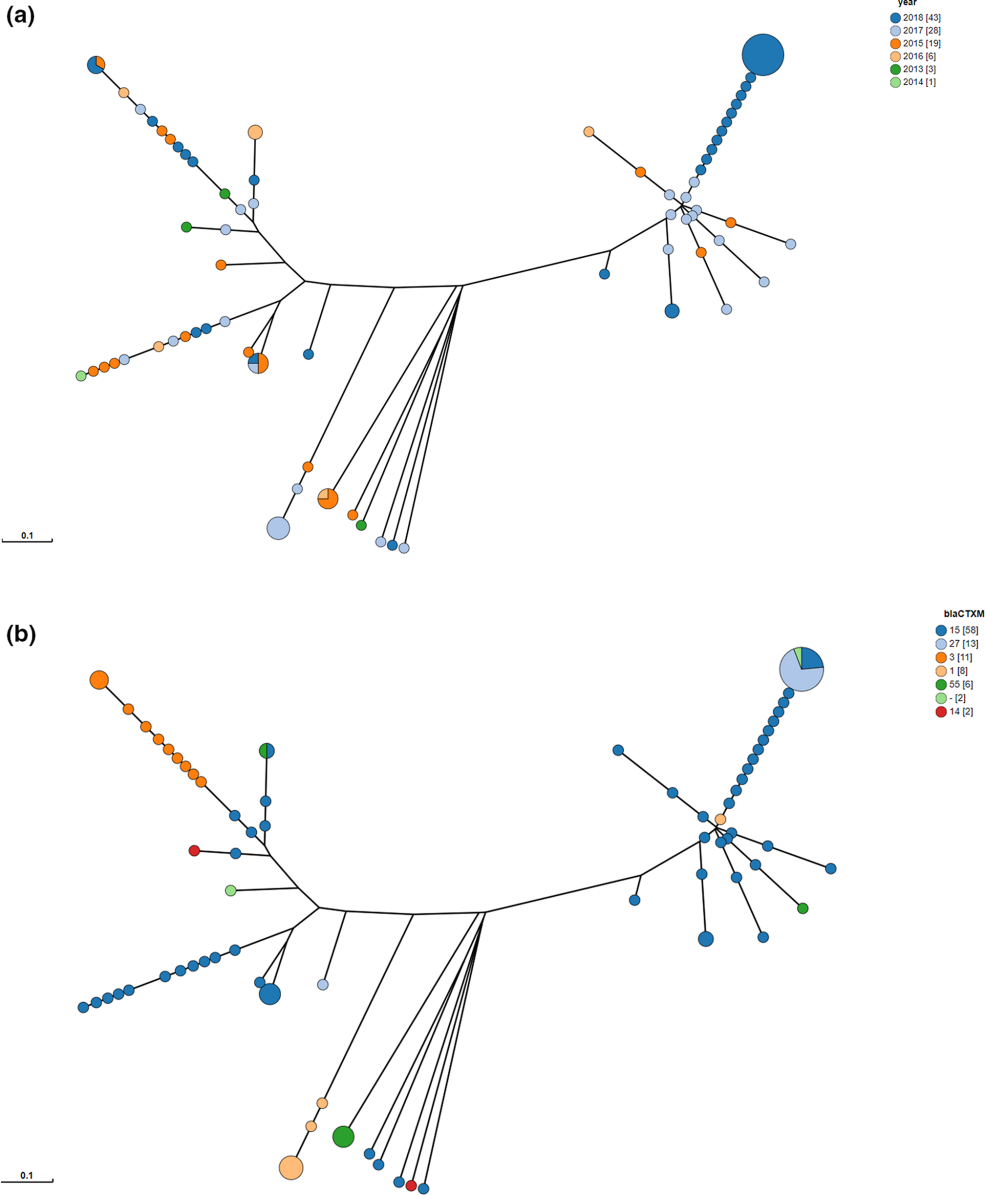
Neighbour-joining tree based on a Plasmid pubMLST analysis on the short-read assemblies of *Shigella* isolates indicating that *bla_CTX-M-15_* and *bla_CTX-M-27_* isolates harboured different plasmids after 2017 compared to previous years. Each node represents at least one of the 100 *Shigella* isolates, where a bigger node represents multiple isolates. The distance between nodes corresponds to the number of different allele identifiers divided by the number of shared allele identifiers. Colours indicate the isolation year of isolates (**a**) or the *bla_CTX-M_* gene variant (**b**).

**Fig. 7.**
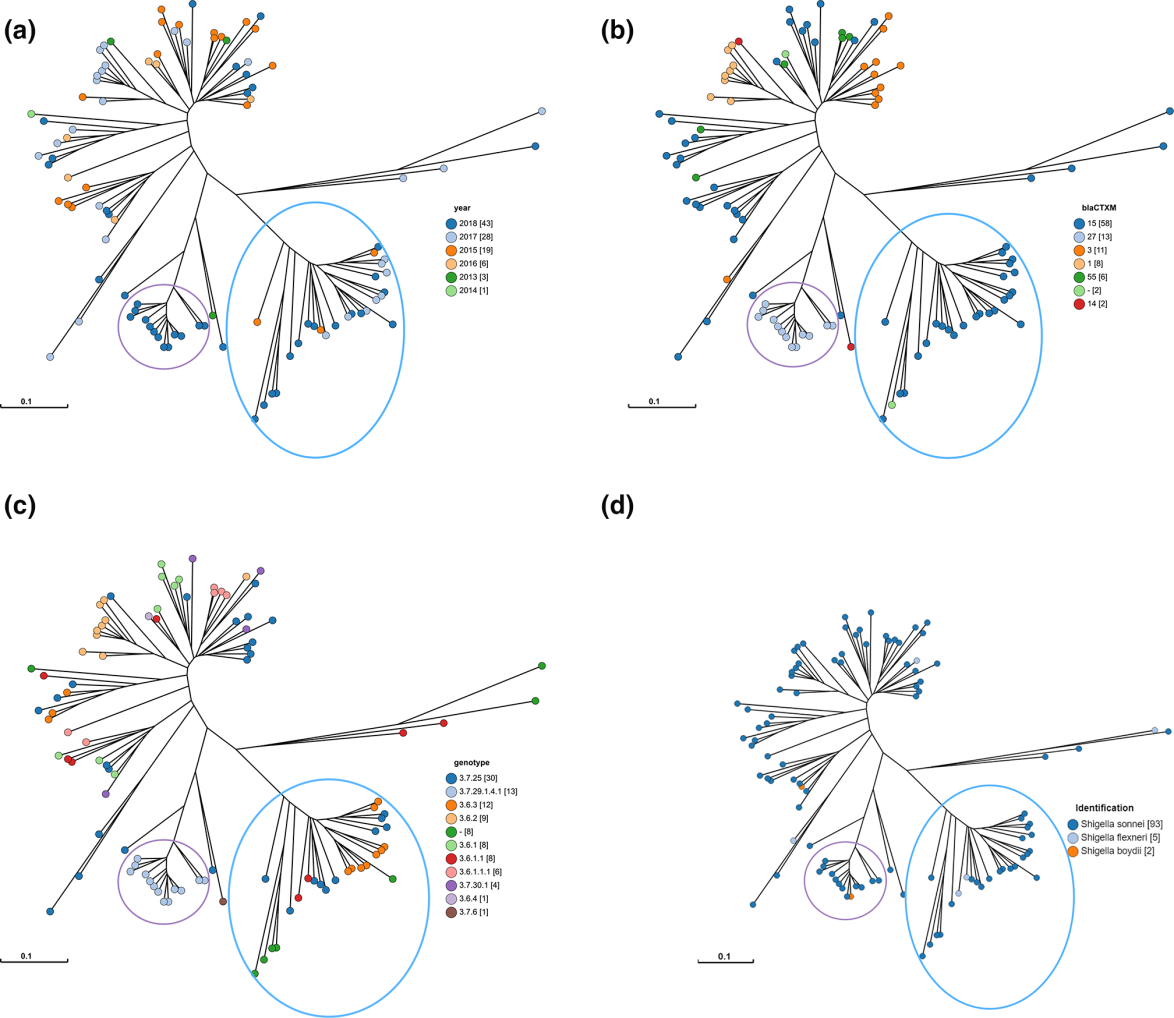
Clonal and interspecies transmission within *bla*_CTX-M-15_ and *bla*_CTX-M-27_ clusters in *Shigella *. Neighbour-joining tree based on ESBL plasmid alignments with filtered short-read assemblies showing more precise clustering on ESBL plasmid diversity that also separates *bla*_CTX-M-15_ and *bla*_CTX-M-27_ isolates. Each node represents at least one of the 100 *Shigella* isolates, where a bigger node represents multiple isolates. The distance between nodes is based on the average nucleotides aligned between genome pairs and then normalized to a value between 0 and 1. Colours indicate the isolation year of isolates (**a**) or the *bla_CTX-M_* gene variant (**b**) or the genotype (**c**). or the species (**d**). The clusters of *bla*_CTX-M-15_ and *bla*_CTX-M-27_ isolates are indicated with a blue and purple circle, respectively.

Phylogenomic trees based on short-read sequencing data were generated to determine whether the similarity between the *Shigella* isolates, including their plasmid content, could be linked to the year of isolation or recent travel. Moreover, by clustering the isolates using different methods we aimed to examine whether the rapid rise of ESBL in 2017 was caused by a common plasmid or several unique plasmids. Different methods were used to generate phylogenomic trees. However, some algorithms require sequences with high similarity and while there are some ESBL plasmids that show high similarity (e.g. IncFII plasmids), there are other ESBL plasmids that have rearrangements, insertions/deletions of genes, point mutations and different plasmid replicons. Thus, the differences in these plasmids made them unsuitable for methods such as SNP phylogenies as one reference sequence is needed for all isolates. A core gene MLST tree could be made (Fig. S5), but this depicted mostly the chromosomal differences between isolates and no conclusions on the plasmid content. The most useful trees were generated with the plasmid MLST scheme from pubMLST (Fig. 6) and a neighbour-joining tree made with Mauve Progressive (global alignments) (Fig. 7).

The Plasmid pubMLST contains sequences of plasmid replicons and other common plasmid genes. Therefore, clustering based on pubMLST (Fig. 6) shows several clusters of isolates with a similar plasmid profile. When the clusters are annotated by year of isolation (Fig. 6a) and ESBL gene variant (Fig. 6b), a cluster with isolates from 2017 and 2018 that mainly contains *bla*_CTX-M-15_ and bla_CTX-M-27_ could be detected. This correlates with the rapid increase in the ESBL phenotype that was seen in *Shigella* in these years. Furthermore, these isolates are on the opposite part of the tree compared to *bla*_CTX-M-15_-positive isolates from before 2017. These observations indicate that these 2017–2018 isolates contained a different plasmid profile than the isolates of previous years.

Moreover, when a clustering method is used that is based on alignment of the ESBL plasmid sequences and that is not restricted by a database such as with the plasmid pubMLST scheme (Fig. 6), it is possible to determine how similar these ESBL plasmids are (Fig. 7). Similarly to the plasmid pubMLST tree (Fig. 6), the isolates from 2017 and 2018 containing the *bla*_CTX-M-15_ and *bla*_CTX-M-27_ genes clustered separately from the other isolates (Fig. 7). However, based on the plasmid alignment clustering (Fig. 7), it could be noted that the isolates containing *bla*_CTX-M-15_ and *bla*_CTX-M-27_ also clustered separately from each other, indicating that the plasmids have a slightly different overall structure, even though both contain the IncFII plasmid replicon. This separate clustering cannot be clarified by the different *bla*_CTX-M_ variants, because *bla*_CTX-M-15_ and *bla*_CTX-M-27_ genes have 75.3 % sequence similarity and 94 % coverage (accession numbers: AY044436 and AY156923). Interestingly, the isolates of the *bla*_CTX-M-27_ cluster all had the same genotype profile while there was a mix of three genotype profiles in the *bla*_CTX-M-15_ cluster (exchange of plasmids) (Fig. 7c). Moreover, in both clusters the most predominant species was *S. sonnei *, but in the *bla*_CTX-M-27_ cluster there was a single *S. boydii* isolate and in the *bla*_CTX-M-15_ cluster there were two *S. flexneri* isolates present (Fig. 7d). This indicates that there may have been mostly clonal expansion within the *bla*_CTX-M-27_ cluster and ESBL plasmid exchange between genotypes and species in the *bla*_CTX-M-15_ cluster.

To determine the exact similarity of these ESBL plasmids within the clusters, the sequencing reads were filtered, assemble, and mapped to the reconstructed plasmids (Fig. S6 and Table S10). The *bla*_CTX-M-15_ and *bla*_CTX-M-27_ clusters contain many highly similar or identical plasmids compared to the reconstructed IncFII plasmids p34, p54, p59, p67 and p83, which are all isolated after 2017 (Fig. S6 and Table S10). This confirms that there was a shift in the sequence of the ESBL plasmids after 2017 that was probably caused by the introduction of a new plasmid. Moreover, between the *bla*_CTX-M-15_ and *bla*_CTX-M-27_ clusters there is also high similarity, which indicates similar backbones in these plasmids and this caused them to cluster together in Fig. 6.

To determine a possible origin of the ESBL plasmid in *bla*_CTX-M-15_, the metadata were further analysed. Travel data were only available for 16/100 *Shigella* isolates (Table S1). The majority of these known travellers went to Egypt (7/16) of which three travellers were in the *bla*_CTX-M-15_ cluster and two travellers in the *bla*_CTX-M 27_ cluster. Moreover, Iran and Georgia were each destinations where one traveller from the *bla*_CTX-M-15_ cluster went to. For the *bla*_CTX-M-27_ cluster the only other destination was Switzerland.

Even though Egypt has a history of *bla*_CTX-M-15_ and other ESBL variants in *Enterobacteriaceae* [95, 96] these isolates were only detected in 2018. However, at the beginning of the *bla*_CTX-M-15_ cluster there was a traveller returning from Iran in 2017. Although only five out of 26 patients of the *bla*_CTX-M-15_ cluster reported recent travel, this indicates that after introduction of the ESBL plasmid there was also domestic transfer or that not all travel was reported by the patients. Moreover, other studies *regarding Shigella* surveillance in non-endemic regions have shown that travellers contributed to rising levels of ESBLs [47, 97, 98].

For the *Salmonella* ESBL plasmids, it was clear by direct comparisons between the reconstructed plasmids that there was not much similarity among all the different serovars. When filtering, assembling and mapping the sequencing reads of all *Salmonella* isolates to the reconstructed *Salmonella* plasmid mapping (Table S12), the highest similarity was detected to the reconstructed plasmids p123 (2017, Infantis), p127 (2018, Infantis) and p131 (2018, Typhimurium), with for p131 100 % similarity to reads of i111 (2016), i118 (2017) and i129 (2018) (Table S12). No phylogenetic analysis for the *Salmonella* isolates was performed, because there was less similarity among these plasmids, the sample size was smaller and there was no rise in the ESBL phenotype.

## Conclusion

In the current study, the diversity and genetic context of ESBL genes in clinical *Shigella* and *Salmonella* spp. isolates received by the NRCSS in the period 2013–2018 was investigated. We applied a previously described WGS workflow [49] to these isolates, as with only phenotypical and PCR-based results from routine surveillance, it had not been possible previously to determine the genetic context of these ESBL genes. This strategy consisted of short-read sequencing performed on all isolates and, based on the resulting data, a selection of representative isolates was made for long-read sequencing. With hybrid sequencing, the ESBL plasmids were then reconstructed, which were used as references for mapping of the short-read data and other analyses, to obtain insight into the ESBL diversity and genetic context. While plasmid prediction tools (based on machine learning) are a promising alternative, there are still some incorrect predictions which necessitate the use of long-read sequencing.

A rise as of 2017 in ESBL-positive *Shigella* isolates could be explained by an increase of *bla*_CTX-M-15_ and an introduction of *bla*_CTX-M-27_ as from 2018. These genes were mostly present on multi-resistance-carrying IncFII plasmids. Based on our clustering and similarity analyses, it is likely that international travel introduced IncFII plasmids containing *bla*_CTX-M-15_ in Belgium in 2017 that caused the rapid rise in the *Shigella* ESBL phenotype. Although there were several travel events in the cluster of the responsible plasmids, not much travel information was available, which may indicate that there was also domestic transfer of these plasmids. For this *bla_CTX-M-15_* cluster, exchange of similar IncFII plasmids between different *S. sonnei* genotypes and even interspecies exchange between *S. sonnei* and *S. flexneri* was observed. The *bla*_CTX-M-27_ cluster, observed from 2018, showed clonal expansion of one specific genotype containing a similar ESBL-positive IncFII plasmid as that occurring in the *bla_CTX-M-15_* cluster. Interestingly, our approach allowed us to determine the chromosomal integration of ESBL genes into the *Shigella* isolates. This has not previously been reported.

In contrast, for *Salmonella *, all ESBL genes were located on plasmids. Although based on limited results during our study period, the local epidemiology seemed to be dominated by the IncHI2 plasmid replicon. Among the *Salmonella* ESBL plasmids there was high diversity, but due to the low number of ESBL-positive *Salmonella* isolates detected each year, it remained difficult to determine their source. Even though the *Salmonella* ESBL plasmids showed high similarity to the *Shigella* ESBL plasmids, there were no perfect matches between them and the most prevalent ESBL gene and plasmid replicons were different between the collections, which makes it unlikely that recent exchange of a plasmid occurred between the two species.

The strategy described in this study could also be applied to other bacterial collections or routine surveillance. Moreover, it should be possible to detect whether there is exchange of MGEs between bacteria of different sources/econiches (One-Health) or between different species. Thus it would be especially interesting to understand AMR gene dynamics between different environments and perform proper risk assessment, in order to make informed policy decisions. Currently, hybrid assemblies are still necessary to reconstruct plasmids with their accurate genetic content, including AMR genes, and size. The long-read sequencing experiments in this study have been performed with R9.4.1 MinION flowcells. However, recently Nanopore also released the R10 flowcells which promise higher accuracy [99]. Nevertheless, it seems likely that an even higher accuracy and reduction in cost is needed to be able to use only long-read sequencing for surveillance.

WGS is a very powerful tool, allowing the retrieval of considerable information from bacterial isolates and plasmids. However, if the metadata are lacking, it will make links between different isolates and plasmids less strong. Also, more detailed metadata could improve our knowledge on what drives certain plasmids to disseminate rapidly and what could be done to reduce their prevalence. Not only are the metadata of importance, but so too which data are available in databases. While some databases provide raw sequencing reads and assemblies, others provide only assemblies. To determine if differences between sequences (assemblies) are not caused by differences in the bioinformatic methods, it is important to have the raw sequencing reads available. Therefore, more databases that include raw data, assemblies and curation would support surveillance and tracking of problematic plasmids.

In conclusion, this study delivered another proof-of-concept of how the use of state-of-the art AMR detection tools based on short- and long-read WGS offers a strategy to understand how resistance develops and spreads, thereby contributing to the objectives put forward by the WHO to combat AMR resistance. Moreover, currently, standard application of WGS data for epidemiological surveillance, such as core gene MLST, is typically restricted to variation within core chromosomal loci, meaning that plasmids and other MGEs are mostly overlooked. However, most resistance markers are encoded on plasmids, which frequently recombine. Therefore, to improve understanding and to prevent the transmission of AMR, future surveillance should include plasmid sequencing, for which we have proposed a strategy.

## Supplementary Data

Supplementary material 1
