# Supplementary material for: Using a combination of short- and long-read sequencing to investigate the diversity in plasmid- and chromosomally encoded extended-spectrum beta-lactamases (ESBLs) in clinical Shigella and Salmonella isolates in Belgium
Source: Microb Genom. 2023 Jan 23;9(1):mgen000925. doi: 10.1099/mgen.0.000925 (PMC9973847; doi:10.1099/mgen.0.000925)
Supplement: Supplementary material 1 [file mgen-9-925-s001.pdf]

Using a combination of short and long read sequencing to investigate the diversity in plasmid- and chromosomally encoded extended-spectrum-beta-lactamases (ESBL) in clinical Shigella and Salmonella isolates in Belgium

Supplementary results

Table of content

Table/figure number	Table/figure title	page number
Table S1	Description and metadata of the clinical *Shigella* and *Salmonella* isolates used in this study	3
Table S2	average values of short read assembly parameters of the 100 *Shigella* and 31 *Salmonella* isolates	11
Table S3	Antimicrobial resistance genes detected with ResFinder in all short-read assemblies of the *Shigella* isolates used in this study	12
Table S4	Antimicrobial resistance genes detected with ResFinder in all short-read assemblies of the *Salmonella* isolates used in this study	45
Table S5	PlasmidFinder output of all *Shigella* isolates used in this study	55
Table S6	PlasmidFinder output of all *Salmonella* isolates used in this study	69
Table S7	prediction with BLAST, mlplasmid, plasflow and mobsuite on location (chromosome or plasmid) of each ESBL containing short-read contig of *Shigella*	72
Figure S1	inhouse genotyping assays to determine *CTX-M* gene variants in *Shigella*.	76
Figure S2	global alignment between all reconstructed IncI1-I(Gamma) (A.), IncB/O/K/Z (B.), IncFIA (C.) and IncHI2 (D.) plasmids. The colours indicate locally collinear blocks (LCBs), which are homologous regions without rearrangements between two or more sequences.	79

Figure S3	Context of the ESBL genes (green), transposases (blue), hypothetical genes (grey) and other genes (purple) around the ESBL gene of *Shigella* i3 (A), i4 (B), i5 (C), i9 (D), i12 (E), i20 (F), i31(G), i38 (H), i39 (I), i54 (J), i55 (K), i59 (L), i67 (M), i69 (N), i73 (O) and i83 (P) The direction of the arrow shows displays the orientation of the genes.	87
Table S8	transposases detected in short read assemblies	88
Table S9	*Shigella* pointfinder output	96
Figure S4	alignments between chromosomal ESBL integrations in i36 and i41 (A.), between i36 and the ISEcp1 sequence (B.) and between i41 and the ISEcp1 sequence (C.)	111
Table S10	mapping of *Shigella* miseq reads to reconstructed *Shigella* plasmids	112
Table S11	mapping of *Salmonella* miseq reads to reconstructed *Shigella* plasmids	115
Table S12	mapping of *Salmonella* miseq reads to reconstructed *Salmonella* plasmids	116
Table S13	mapping of *Shigella* miseq reads to reconstructed *Salmonella* plasmids	118
Figure S5	cgMLST tree of all *Shigella* isolates and coloured by year of isolation (A.) and *blaCTX-M* variant (B.)	123
Figure S6	High similarity of ESBL plasmids in $bla_{CTX\text{-}M\text{-}15}$ and $bla_{CTX\text{-}M\text{-}27}$ clusters in *Shigella*	124
Figure S7	Global alignments between p3 (A.), p4 (B.), p5 (C.) p9 (D.), p12 (E.), p20 (F.), p31 (G.), p34 (H.), p38 (I.), p39 (J.), p54 (K.), p55 (L.), p59 (M.), p67 (N.), p69 (O.), p73 (P.), p83 (Q.), p105 (R.), p106 (S.), p114 (T.), p123 (U.), p127 (V.), p131 (W.) and their top blast hits The colours indicate locally collinear blocks (LCBs), which are homologous	136

	regions without rearrangements between two or more sequences.	
Table S14	blast hits 2nd to 10th for p3	136
Table S15	blast hits 2nd to 10th for p4	137
Table S16	blast hits 2nd to 10th for p5	137
Table S17	blast hits 2nd to 10th for p9	138
Table S18	blast hits 2nd to 10th for p12	138
Table S19	blast hits 2nd to 10th for p20	139
Table S20	blast hits 2nd to 10th for p31	139
Table S21	blast hits 2nd to 10th for p34	140
Table S22	blast hits 2nd to 10th for p38	140
Table S23	blast hits 2nd to 10th for p39	141
Table S24	blast hits 2nd to 10th for p54	141
Table S25	blast hits 2nd to 10th for p55	142
Table S26	blast hits 2nd to 10th for p59	142
Table S27	blast hits 2nd to 10th for p67	143
Table S28	blast hits 2nd to 10th for p69	143
Table S29	blast hits 2nd to 10th for p73	143
Table S30	blast hits 2nd to 10th for p83	144
Table S31	blast hits 2nd to 10th for p105	144
Table S32	blast hits 2nd to 10th for p106	145
Table S33	blast hits 2nd to 10th for p114	145
Table S34	blast hits 2nd to 10th for p123	146
Table S35	blast hits 2nd to 10th for p127	146
Table S36	blast hits 2nd to 10th for p131	147

Table S1: Description and metadata of the clinical *Shigella* and *Salmonella* isolates used in this study

full name isolate	Code isolate	Code ESBL plasmid	BioSample accession	year	ST	*blaCTXM*	*blaTEM*	*blaOXA*	Patient Sex	Patient Age	Recently Stayed Abroad	Travel to	Source	Species	Genotype	Serotype	reason for inclusion long read sequencing
S13BD00549	i1	p1	SAMN28885442	2013	152	15	-	-	Male	53	Y	Lao People's Democratic Republic	faeces	*Shigella sonnei*	3.6.1.1.1	-	-
S13BD02710	i2	p2	SAMN28885443	2013	152	15	1B	-	Female	34	N	-	faeces	*Shigella sonnei*	3.7.25	-	-
S13BD04362	i3	p3	SAMN28885444	2013	152	14	-	-	Male	42	N	-	faeces	*Shigella sonnei*	3.7.25	-	contains *blaCTXM-14* and col(MG828)
S14BD05406	i4	p4	SAMN28885445	2014	245	15	1B	-	Female	50	N	-	faeces	*Shigella sonnei*	-	-	contains *blaCTXM-15* + uncertain whether ESBL gene was on chromosome or plasmid with short read data
S15BD02407	i5	p5	SAMN28885446	2015	152	15	-	-	unknown	12	N	-	faeces	*Shigella sonnei*	3.6.1	-	contains blaCTXM-15 + contains IncB/O/K/Z_4 plasmid replicon
S15BD03051	i6	p6	SAMN28885447	2015	152	3	-	-	Male	21	N	-	faeces	*Shigella sonnei*	3.7.25	-	-
S15BD06181	i7	p7	SAMN28885448	2015	152	3	-	-	Male	21	N	-	faeces	*Shigella sonnei*	3.7.25	-	-
S15BD06648	i8	p8	SAMN28885449	2015	152	3	-	-	Female	9	N	-	faeces	*Shigella sonnei*	3.7.25	-	-
S15BD06969	i9	p9	SAMN28885450	2015	152	1	-	-	Female	58	N	-	other	*Shigella sonnei*	3.6.2	-	contains *blaCTXM-1* and similar plasmid profile to other travelers to Thailand
S15BD07164	i10	p10	SAMN28885451	2015	152	15	-	-	Female	2	N	-	faeces	*Shigella sonnei*	3.7.25	-	-
S15BD07250	i11	p11	SAMN28885452	2015	152	15	-	-	Male	0	Y	Georgia	faeces	*Shigella sonnei*	3.7.25	-	-
S15BD07413	i12	p12	SAMN28885453	2015	152	15	1B	-	Female	69	N	-	faeces	*Shigella sonnei*	3.6.2	-	contains *blaCTXM-15* and on same short read contig *blaTEM* + high BLAST similarity to many other likely plasmid contigs of *Shigella* isolates
S15BD08049	i13	p13	SAMN28885454	2015	152	15	-	-	Male	60	N	-	faeces	*Shigella sonnei*	3.6.3	-	-
S15BD08205	i14	p14	SAMN28885455	2015	152	-	-	-	Female	52	N	-	faeces	*Shigella sonnei*	3.6.1	-	-
S15BD08240	i15	p15	SAMN28885456	2015	152	15	-	-	Female	52	N	-	faeces	*Shigella sonnei*	3.6.1.1	-	-
S15BD08296	i16	p16	SAMN28885457	2015	152	15	-	-	Female	3	N	-	faeces	*Shigella sonnei*	3.6.1.1	-	-
S15BD08759	i17	p17	SAMN28885458	2015	152	15	1B	-	Male	3	N	-	faeces	*Shigella sonnei*	3.6.1	-	-

S15BD08835	i18	p18	SAMN28885459	2015	152	15	1B	-	Male	45	Y	Thailand	faeces	*Shigella sonnei*	3.6.1	-	-
S15BD09162	i19	p19	SAMN28885460	2015	152	55	-	-	Female	12	Y	Thailand	faeces	*Shigella sonnei*	3.6.1.1.1	-	-
S15BD09164	i20	p20	SAMN28885461	2015	152	55	-	-	Male	45	Y	Thailand	faeces	*Shigella sonnei*	3.6.1.1.1	-	contains *blaCTXM-55* + travel to Thailand with similar plasmid profile to other travelers
S15BD09165	i21	p21	SAMN28885462	2015	152	55	-	-	Female	11	N	-	faeces	*Shigella sonnei*	3.6.1.1.1	-	-
S15BD09964	i22	p22	SAMN28885463	2015	152	15	1B	-	Male	69	N	-	faeces	*Shigella sonnei*	3.6.1	-	-
S15BD10177	i23	p23	SAMN28885464	2015	152	15	-	-	Male	72	N	-	other	*Shigella sonnei*	3.6.1.1.1	-	-
S16BD00902	i24	p24	SAMN28885465	2016	152	3	-	-	Female	2	N	-	faeces	*Shigella sonnei*	3.7.25	-	-
S16BD01990	i25	p25	SAMN28885466	2016	152	15	-	-	Female	68	N	-	faeces	*Shigella sonnei*	3.6.1	-	-
S16BD04164	i26	p26	SAMN28885467	2016	152	15	-	-	Female	28	Y	Cambodia	faeces	*Shigella sonnei*	3.6.3	-	-
S16BD05101	i27	p27	SAMN28885468	2016	152	55	-	-	Female	23	N	-	faeces	*Shigella sonnei*	3.6.1.1.1	-	contains *blaCTXM-55* + contains incX4 plasmid replicon that is unique in collection + high BLAST similarity to many other likely plasmid contigs of *Shigella* isolates
S16BD05219	i28	p28	SAMN28885469	2016	152	55	-	-	unknown	30	N	-	unknown	*Shigella sonnei*	3.6.1.1	-	-
S16BD05540	i29	p29	SAMN28885470	2016	1503	15	-	-	Male	72	N	-	faeces	*Shigella sonnei*	3.6.4	-	-
S17BD00112	i30	p30	SAMN28885471	2017	152	15	1B	-	Male	45	N	-	Autre - Andere	*Shigella sonnei*	3.7.25	-	-
S17BD00672	i31	p31	SAMN28885472	2017	152	3	-	-	Female	65	N	-	faeces	*Shigella flexneri*	3.7.25	6	contains *blaCTXM-3* + plasmid replicon ColpVC
S17BD03431	i32	p32	SAMN28885473	2017	145	15	-	-	Female	65	N	-	faeces	*Shigella sonnei*	-	-	-
S17BD03508	i33	p33	SAMN28885474	2017	152	15	1B	-	Male	46	N	-	faeces	*Shigella sonnei*	3.7.25	-	-
S17BD04134	i34	p34	SAMN28885475	2017	152	15	-	-	Male	4	Y	Iran, Islamic Republic of	faeces	*Shigella sonnei*	3.7.25	-	contains *blaCTXM-15* and IncFII on same contig, which was only visible in this isolate with short read data
S17BD04542	i35	p35	SAMN28885476	2017	152	15	-	-	Female	25	Y	India	faeces	*Shigella sonnei*	3.7.30.1	-	-
S17BD05200	i36	p36	SAMN28885477	2017	152	15	1B	-	Male	35	N	-	faeces	*Shigella sonnei*	3.6.1.1	-	based on short read data it was likely that ESBL gene was located on chromosome
S17BD05456	i37	p37	SAMN28885478	2017	152	15	-	-	Male	12	N	-	faeces	*Shigella sonnei*	3.7.25	-	-
S17BD05916	i38	p38	SAMN28885479	2017	152	15	-	-	unknown	-	N	-	faeces	*Shigella sonnei*	3.7.25	-	contains *blaCTXM-15*, but the plasmid contig showed low similarity to other *blaCTXM-15 Shigella* isolates

S17BD05944	i39	p39	SAMN28885480	2017	152	15	1B	-	Male	2	N	-	faeces	*Shigella sonnei*	3.7.25	-	unique plasmid replicon
S17BD06235	i40	p40	SAMN28885481	2017	152	15	187	-	Female	19	N	-	faeces	*Shigella sonnei*	3.6.1	-	-
S17BD06357	i41	p41	SAMN28885482	2017	152	15	-	-	Female	49	N	-	faeces	*Shigella sonnei*	3.6.1.1	-	contains *blaCTXM-15*, but the plasmid contig showed low similarity to other *blaCTXM-15 Shigella* isolates
S17BD06649	i42	p42	SAMN28885483	2017	152	15	-	-	Female	30	N	-	faeces	*Shigella sonnei*	3.7.25	-	-
S17BD06975	i43	p43	SAMN28885484	2017	152	15	1B	-	Female	6	N	-	faeces	*Shigella sonnei*	3.6.1	-	-
S17BD07077	i44	p44	SAMN28885485	2017	152	15	-	-	Male	40	N	-	faeces	*Shigella sonnei*	3.7.25	-	-
S17BD07216	i45	p45	SAMN28885486	2017	152	1	-	-	Female	57	N	-	faeces	*Shigella sonnei*	3.6.2	-	-
S17BD07293	i46	p46	SAMN28885487	2017	152	15	-	-	Female	6	N	-	faeces	*Shigella sonnei*	3.7.25	-	-
S17BD07332	i47	p47	SAMN28885488	2017	152	1	-	-	Male	4	N	-	faeces	*Shigella sonnei*	3.6.2	-	-
S17BD07399	i48	p48	SAMN28885489	2017	152	1	-	-	Male	9	N	-	faeces	*Shigella sonnei*	3.6.2	-	-
S17BD07692	i49	p49	SAMN28885490	2017	152	15	-	-	unknown	3	N	-	faeces	*Shigella sonnei*	3.6.3	-	-
S17BD07935	i50	p50	SAMN28885491	2017	152	15	-	-	unknown	52	N	-	faeces	*Shigella sonnei*	3.6.3	-	-
S17BD07949	i51	p51	SAMN28885492	2017	152	55	-	-	unknown	34	N	-	faeces	*Shigella sonnei*	3.7.25	-	-
S17BD08123	i52	p52	SAMN28885493	2017	152	1	-	-	Male	2	N	-	faeces	*Shigella sonnei*	3.6.2	-	-
S17BD08170	i53	p53	SAMN28885494	2017	152	1	-	-	Female	28	N	-	faeces	*Shigella sonnei*	3.6.2	-	-
S17BD08179	i54	p54	SAMN28885495	2017	152	15	-	-	Female	4	N	-	faeces	*Shigella sonnei*	3.6.3	-	plasmid prediction tools showed that ESBl gene was likely on plasmid but analysis with BLAST was uncertain
S17BD08233	i55	p55	SAMN28885496	2017	152	1	-	-	Female	37	N	-	faeces	*Shigella sonnei*	3.6.2	-	contains *blaCTXM-1* and shows similiarity to other *blaCTXM-1* isolates
S17BD08293	i56	p56	SAMN28885497	2017	152	1	-	-	Female	4	N	-	faeces	*Shigella sonnei*	3.6.2	-	-
S17BD08948	i57	p57	SAMN28885498	2017	152	15	-	-	Male	10	N	-	faeces	*Shigella sonnei*	3.6.3	-	-
S18BD01024	i58	p58	SAMN28885499	2018	152	15	-	-	Female	38	N	-	faeces	*Shigella sonnei*	3.6.3	-	-
S18BD01106	i59	p59	SAMN28885500	2018	152	15	-	-	Male	14	N	-	faeces	*Shigella sonnei*	3.6.3	-	similarity to other incl1 Shigella plasmids and 1 Salmonella plasmid
S18BD02025	i60	p60	SAMN28885501	2018	152	15	-	-	Male	5	N	-	faeces	*Shigella sonnei*	3.6.3	-	-

S18BD02468	i61	p61	SAMN28885502	2018	-	15	-	-	Male	2	N	-	faeces	*Shigella sonnei*	-	-	-
S18BD02580	i62	p62	SAMN28885503	2018	152	3	-	-	Female	29	N	-	faeces	*Shigella sonnei*	3.7.25	-	-
S18BD02594	i63	p63	SAMN28885504	2018	152	15	1B	-	Female	40	N	-	faeces	*Shigella flexneri*	3.6.1.1	2a	-
S18BD02657	i64	p64	SAMN28885505	2018	152	15	-	-	Male	26	N	-	faeces	*Shigella sonnei*	3.6.3	-	-
S18BD02761	i65	p65	SAMN28885506	2018	152	15	-	-	Male	45	N	-	faeces	*Shigella sonnei*	3.6.3	-	-
S18BD02818	i66	p66	SAMN28885507	2018	245	15	-	-	Female	6	N	-	faeces	*Shigella sonnei*	-	-	-
S18BD03178	i67	p67	SAMN28885508	2018	152	27	-	-	Male	24	N	-	faeces	*Shigella sonnei*	3.7.29.1.4.1	-	contains *blaCTXM* -27 + 19kb alignment with short read data to many other *blaCTXM*-27 containing isolates
S18BD03186	i68	p68	SAMN28885509	2018	152	27	-	-	Male	30	N	-	faeces	*Shigella sonnei*	3.7.29.1.4.1	-	-
S18BD03411	i69	p69	SAMN28885510	2018	152	14	-	-	Male	35	N	-	faeces	*Shigella sonnei*	3.7.6	-	contains *blaCTXM* -14 and incB/O/K/Z on same contig, which was only visible in this isolate with short read data
S18BD03575	i70	p70	SAMN28885511	2018	152	27	-	-	Female	63	Y	Egypt	faeces	*Shigella sonnei*	3.7.29.1.4.1	-	-
S18BD03841	i71	p71	SAMN28885512	2018	152	27	-	-	Male	41	N	-	faeces	*Shigella sonnei*	3.7.29.1.4.1	-	-
S18BD04258	i72	p72	SAMN28885513	2018	152	27	-	-	Male	58	N	-	faeces	*Shigella sonnei*	3.7.29.1.4.1	-	-
S18BD04295	i73	p73	SAMN28885514	2018	152	15	-	-	Male	71	N	-	faeces	*Shigella sonnei*	3.7.25	-	contains Col(KPHS6) which was unique in the collection.
S18BD04337	i74	p74	SAMN28885515	2018	152	27	-	-	Male	21	N	-	faeces	*Shigella sonnei*	3.7.29.1.4.1	-	-
S18BD04382	i75	p75	SAMN28885516	2018	152	27	-	-	Female	42	Y	Switzerland	faeces	*Shigella sonnei*	3.7.29.1.4.1	-	-
S18BD04853	i76	p76	SAMN28885517	2018	152	27	-	-	Male	21	N	-	faeces	*Shigella sonnei*	3.7.29.1.4.1	-	-
S18BD04903	i77	p77	SAMN28885518	2018	152	27	-	-	Male	19	N	-	faeces	*Shigella sonnei*	3.7.29.1.4.1	-	-
S18BD04953	i78	p78	SAMN28885519	2018	152	27	-	-	Male	52	N	-	faeces	*Shigella sonnei*	3.7.29.1.4.1	-	-
S18BD05188	i79	p79	SAMN28885520	2018	152	27	-	-	Female	66	N	-	faeces	*Shigella sonnei*	3.7.29.1.4.1	-	-
S18BD05398	i80	p80	SAMN28885521	2018	152	27	-	-	Male	33	Y	Egypt	faeces	*Shigella sonnei*	3.7.29.1.4.1	-	-
S18BD05404	i81	p81	SAMN28885522	2018	152	15	-	-	Female	5	Y	Egypt	faeces	*Shigella flexneri*	3.6.1.1	4	-
S18BD05550	i82	p82	SAMN28885523	2018	152	3	-	-	Female	32	Y	Egypt	faeces	*Shigella sonnei*	3.7.25	-	-

S18BD05612	i83	p83	SAMN28885524	2018	152	15	-	-	Male	14	Y	Egypt	faeces	*Shigella sonnei*	3.7.25	-	travel to Egypt + similarity to other travelers based on short read data
S18BD05858	i84	p84	SAMN28885525	2018	245	15	-	1	Male	16	Y	Egypt	faeces	*Shigella sonnei*	-	-	-
S18BD05859	i85	p85	SAMN28885526	2018	152	15	-	-	Female	24	N	-	faeces	*Shigella sonnei*	3.7.25	-	-
S18BD05904	i86	p86	SAMN28885527	2018	152	15	-	-	Male	47	N	-	faeces	*Shigella boydii*	3.7.25	-	-
S18BD05921	i87	p87	SAMN28885528	2018	152	15	-	-	Male	48	N	-	faeces	*Shigella sonnei*	3.7.25	-	-
S18BD06091	i88	p88	SAMN28885529	2018	152	15	1B	-	Male	11	N	-	faeces	*Shigella sonnei*	3.7.30.1	-	-
S18BD06159	i89	p89	SAMN28885530	2018	148	15	-	1	Male	11	N	-	faeces	*Shigella sonnei*	-	-	-
S18BD06265	i90	p90	SAMN28885531	2018	152	15	-	-	Male	24	N	-	faeces	*Shigella sonnei*	3.6.3	-	-
S18BD06401	i91	p91	SAMN28885532	2018	152	3	-	-	Female	46	Y	Egypt	faeces	*Shigella sonnei*	3.7.25	-	-
S18BD06486	i92	p92	SAMN28885533	2018	152	3	-	-	Male	2	N	-	faeces	*Shigella flexneri*	3.7.25	4	-
S18BD06501	i93	p93	SAMN28885534	2018	152	15	-	-	Female	1	N	-	faeces	*Shigella sonnei*	3.7.25	-	-
S18BD06724	i94	p94	SAMN28885535	2018	152	15	-	-	Female	53	N	-	faeces	*Shigella sonnei*	3.7.25	-	-
S18BD07214	i95	p95	SAMN28885536	2018	8878	-	1B	-	Male	62	N	-	faeces	*Shigella sonnei*	-	-	-
S18BD07247	i96	p96	SAMN28885537	2018	152	3	-	-	Male	60	N	-	faeces	*Shigella sonnei*	3.7.30.1	-	-
S18BD07270	i97	p97	SAMN28885538	2018	152	3	-	-	Female	42	N	-	faeces	*Shigella sonnei*	3.7.30.1	-	-
S18BD07286	i98	p98	SAMN28885539	2018	152	27	-	-	Male	3	N	-	faeces	*Shigella boydii*	3.7.29.1.4.1	2	-
S18BD09269	i99	p99	SAMN28885540	2018	152	15	1B	-	Female	29	N	-	faeces	*Shigella sonnei*	3.6.1.1	-	-
S18BD09328	i100	p100	SAMN28885541	2018	145	15	1B	-	Male	42	N	-	faeces	*Shigella flexneri*	-	2a	-
S16BD00370	i101	p101	SAMN28885542	2016	34	55	-	-	unknown	3	N	-	faeces	monophasic *Salmonella enterica* serovar 4,[5],12:I:−	-	O:4 (B)	-
S16BD01842	i102	p102	SAMN28885543	2016	469	55	1B	-	Male	2	Y	Thailand	blood	*Salmonella enterica* serovar Rissen	-	O:7 (C1)	-
S16BD02923	i103	p103	SAMN28885544	2016	32	1	-	-	Male	34	N	-	faeces	*Salmonella* Infantis	-	O:7 (C1)	-

S16BD03139	i104	p104	SAMN28885545	2016	34	1	1B	-	Female	3	N	-	faeces	*Salmonella enterica* serovar Typhimurium	-	O:4 (B)	-
S16BD03602	i105	p105	SAMN28885546	2016	11	15	-	-	unknown	1	N	-	faeces	*Salmonella enterica* serotype Enteritidis	-	O:9 (D1)	contains *blaCTXM-15* + only *Salmonella* isolate with incFII
S16BD04351	i106	p106	SAMN28885547	2016	-	15	1B	1	Female	68	N	-	faeces	*Salmonella enterica* serotype Enteritidis	-	O:9 (D1)	contains *blaCTXM-15* and blaOXA + 2 unique plasmid replicons
S16BD04541	i107	p107	SAMN28885548	2016	32	1	-	-	Male	25	N	-	faeces	*Salmonella* Infantis	-	O:7 (C1)	-
S16BD04562	i108	p108	SAMN28885549	2016	34	55	1B	-	Male	4	N	-	faeces	monophasic *Salmonella enterica* serovar 4,[5],12:I:−	-	O:4 (B)	-
S16BD05311	i109	p109	SAMN28885550	2016	34	55	1B	-	Male	69	N	-	faeces	*Salmonella enterica* serovar Typhimurium	-	O:4 (B)	-
S16BD05388	i110	p110	SAMN28885551	2016	34	55	1B	-	Male	13	N	-	faeces	monophasic *Salmonella enterica* serovar 4,[5],12:I:−	-	O:4 (B)	-
S16BD05938	i111	p111	SAMN28885552	2016	19	9	-	-	Male	3	N	-	faeces	*Salmonella enterica* serovar Typhimurium	-	O:4 (B)	-
S16BD06375	i112	p112	SAMN28885553	2016	19	9	-	-	Female	17	N	-	faeces	*Salmonella enterica* serovar Typhimurium	-	O:4 (B)	-
S16BD06461	i113	p113	SAMN28885554	2016	19	1	-	-	Male	83	N	-	faeces	*Salmonella enterica* serovar Typhimurium	-	O:4 (B)	-

S16BD07964	i114	p114	SAMN28885555	2017	34	32	1B	-	Female	88	N	-	Urine	monophasic *Salmonella enterica* serovar 4,[5],12:I:−	-	O:4 (B)	contains *blaCTXM-32* + similarity of plasmid contig to that of Shigella isolate
S17BD00109	i115	p115	SAMN28885556	2017	4157	55	1B	-	Female	41	N	-	faeces	*Salmonella enterica* serovar Newport	-	O:8 (C2-C3)	-
S17BD00327	i116	p116	SAMN28885557	2017	4157	55	1B	-	Female	41	N	-	faeces	*Salmonella enterica* serovar Newport	-	O:8 (C2-C3)	-
S17BD03474	i117	p117	SAMN28885558	2017	1	15	1B	-	unknown	-	Y	Pakistan	blood	*Salmonella* Typhi	-	O:9 (D1)	-
S17BD04225	i118	p118	SAMN28885559	2017	19	9	-	-	Female	3	N	-	faeces	*Salmonella enterica* serovar Typhimurium variant O:5-	-	O:4 (B)	-
S17BD04479	i119	p119	SAMN28885560	2017	19	14	-	-	Male	87	N	-	blood	*Salmonella enterica* subspecies *enterica serovar* Dublin	-	O:9 (D1)	-
S17BD04785	i120	p120	SAMN28885561	2017	19	3	-	-	Female	4	N	-	faeces	monophasic *Salmonella enterica* serovar 4,[5],12:I:−	-	O:4 (B)	-
S17BD06407	i121	p121	SAMN28885562	2017	19	9	1B	-	Female	1	N	-	faeces	*Salmonella* Infantis	-	O:7 (C1)	-
S17BD06808	i122	p122	SAMN28885563	2017	26	-	1B	-	Male	53	N	-	faeces	*Salmonella enterica* serovar Agona	-	O:4 (B)	-
S17BD06931	i123	p123	SAMN28885564	2017	34	9	1B	-	Female	69	N	-	faeces	*Salmonella* Infantis	-	O:7 (C1)	contains *blaCTXM-9* and plasmid replicon p0111
S17BD07444	i124	p124	SAMN28885565	2017	32	1	-	-	Female	26	N	-	faeces	*Salmonella* Infantis	-	O:7 (C1)	-
S17BD08371	i125	p125	SAMN28885566	2017	13	-	1B	-	Female	33	N	-	faeces	*Salmonella* Infantis	-	O:7 (C1)	-
S18BD00539	i126	p126	SAMN28885567	2018	32	-	52C	-	Male	9	N	-	faeces	*Salmonella enterica*	-	O:4 (B)	-

														serovar Typhimurium			
S18BD02037	i127	p127	SAMN28885568	2018	32	65	-	-	Male	48	-	-	faeces	*Salmonella* Infantis	-	O:7 (C1)	contains no plasmid replicons, but *blaCTXM-65* was predicted to be on a plasmid
S18BD03032	i128	p128	SAMN28885569	2018	32	55	-	-	unknown	0	N	-	faeces	*Salmonella enterica* serovar Typhimurium	-	O:4 (B)	-
S18BD06426	i129	p129	SAMN28885570	2018	19	9	-	-	Male	2	N	-	faeces	*Salmonella enterica* serovar Typhimurium variant O:5-	-	O:4 (B)	-
S18BD06686	i130	p130	SAMN28885571	2018	32	65	-	-	Male	3	N	-	faeces	*Salmonella enterica* serovar Typhimurium	-	O:4 (B)	-
S18BD07928	i131	p131	SAMN28885572	2018	19	9			Male	23	N	-	faeces	*Salmonella enterica* serovar Typhimurium	-	O:4 (B)	contains *blaCTXM-9* + only Salmonella isolate with incl2 plasmid replicon

Genotype, sequence type, presence of genes and plasmid replicons were determined with WGS, while serotype was determined with phenotypical methods.

Table S2: average values of short read assembly parameters of the 100 *Shigella* and 31 *Salmonella* isolates

parameter	*Shigella*	*Salmonella*
number of scaffolds	688.8	194
Size (in Mb)	4.7	5.05
N50	61	5.45
L50 (in Kb)	24.4	301.04

N90	224	20.77
L90 (in Kb)	4.7	66.94
max contig length (in Kb)	91.4	891.75
number of contigs >50 Kb	12.9	20.06
GC%	0.507	0.520

Table S3: Antimicrobial resistance genes detected with ResFinder in all short-read assemblies of the *Shigella* isolates used in this study

Isolate	Year	Resistance gene	Identity (%)	Query/Template Length	Contig	Position in contig	Antibiotic class
i1	2013	*aph(3'')-Ib_5*	99.88	804/804	170	3614..4417	aminoglycoside
i1	2013	*aph(6)-Id_1*	100	837/837	170	2778..3614	aminoglycoside
i1	2013	*blaCTX-M-15_1*	100	876/876	3	1541..2416	beta-lactam
i1	2013	*dfrA1_8*	99.79	474/474	161	6438..6911	trimethoprim
i1	2013	*mdf(A)_1*	98.46	1233/1233	143	6783..8010	macrolide
i1	2013	*sul2_2*	100	816/816	170	4478..5293	sulphonamide
i1	2013	*tet(A)_6*	100	1275/1275	170	243..1517	tetracycline
i2	2013	*aadA1_4*	100	789/789	37	29373..30161	aminoglycoside
i2	2013	*blaCTX-M-15_1*	100	876/876	1	79403..80278	beta-lactam
i2	2013	*blaTEM-1B_1*	100	861/861	1	84060..84920	beta-lactam
i2	2013	*dfrA1_8*	100	474/474	37	30838..31311	trimethoprim
i2	2013	*mdf(A)_1*	98.46	1233/1233	30	2966..4193	macrolide
i3	2013	*aadA1_4*	100	789/789	195	2984..3772	aminoglycoside
i3	2013	*blaCTX-M-137_1*	92.58	876/876	6	22562..23437	beta-lactam
i3	2013	*blaCTX-M-14_1*	100	876/876	6	22562..23437	beta-lactam

i3	2013	*dfrA1_8*	100	474/474	195	4449..4922	trimethoprim
i3	2013	*mdf(A)_1*	98.46	1233/1233	49	2964..4191	macrolide
i4	2014	*aac(3)-IId_1*	99.88	861/861	159	282..1142	aminoglycoside
i4	2014	*aph(3'')-Ib_5*	100	804/804	196	2359..3162	aminoglycoside
i4	2014	*aph(6)-Id_4*	100	831/831	196	3168..3998	aminoglycoside
i4	2014	*blaCTX-M-15_1*	100	876/876	261	323..1198	beta-lactam
i4	2014	*blaTEM-1B_1*	100	861/861	281	193..1053	beta-lactam
i4	2014	*dfrA8_1*	99.8	510/510	269	753..1262	trimethoprim
i4	2014	*mdf(A)_1*	98.22	1233/1233	38	32672..33898	macrolide
i4	2014	*sul2_2*	100	816/816	196	1483..2298	sulphonamide
i5	2015	*aph(3'')-Ib_5*	99.88	804/804	163	6241..7044	aminoglycoside
i5	2015	*aph(6)-Id_1*	100	837/837	163	5405..6241	aminoglycoside
i5	2015	*blaCTX-M-15_1*	100	876/876	1	83110..83985	beta-lactam
i5	2015	*dfrA1_8*	99.79	474/474	40	2387..2860	trimethoprim
i5	2015	*mdf(A)_1*	98.46	1233/1233	135	2965..4192	macrolide
i5	2015	*qnrS1_1*	100	657/657	1	88626..89282	quinolone
i5	2015	*sul2_2*	100	816/816	163	7105..7920	sulphonamide
i5	2015	*tet(A)_6*	100	1275/1275	163	2870..4144	tetracycline
i6	2015	*aadA1_4*	100	789/789	34	29374..30162	aminoglycoside
i6	2015	*blaCTX-M-3_1*	100	876/876	61	2465..3340	beta-lactam
i6	2015	*dfrA1_8*	100	474/474	34	30839..31312	trimethoprim
i6	2015	*mdf(A)_1*	98.46	1233/1233	50	21240..22467	macrolide
i7	2015	*aadA1_4*	100	789/789	34	29373..30161	aminoglycoside
i7	2015	*aph(3'')-Ib_5*	100	804/804	162	14..817	aminoglycoside
i7	2015	*aph(6)-Id_1*	100	837/837	162	817..1653	aminoglycoside
i7	2015	*blaCTX-M-3_1*	100	876/876	1	19494..20369	beta-lactam
i7	2015	*dfrA1_8*	100	474/474	34	30838..31311	trimethoprim

i7	2015	*mdf(A)_1*	98.46	1233/1233	27	2966..4193	macrolide
i7	2015	*sul2_2*	100	816/816	162	7539..8354	sulphonamide
i7	2015	*tet(A)_6*	100	1275/1275	162	2914..4188	tetracycline
i8	2015	*aadA1_4*	100	789/789	35	3538..4326	aminoglycoside
i8	2015	*aph(3'')-Ib_5*	100	804/804	165	5676..6479	aminoglycoside
i8	2015	*aph(6)-Id_1*	100	837/837	165	4840..5676	aminoglycoside
i8	2015	*blaCTX-M-3_1*	100	876/876	52	20053..20928	beta-lactam
i8	2015	*dfrA1_8*	100	474/474	35	2388..2861	trimethoprim
i8	2015	*mdf(A)_1*	98.46	1233/1233	27	33353..34580	macrolide
i8	2015	*sul2_2*	100	816/816	165	6540..7355	sulphonamide
i8	2015	*tet(A)_6*	100	1275/1275	165	2305..3579	tetracycline
i9	2015	*aadA5_1*	100	789/789	1	87568..88356	aminoglycoside
i9	2015	*blaCTX-M-1_1*	100	876/876	1	1450..2325	beta-lactam
i9	2015	*dfrA17_1*	100	474/474	1	88487..88960	trimethoprim
i9	2015	*dfrA1_8*	100	474/474	39	29944..30417	trimethoprim
i9	2015	*mdf(A)_1*	98.46	1233/1233	137	6783..8010	macrolide
i9	2015	*sul2_2*	100	816/816	1	84555..85370	sulphonamide
i10	2015	*aadA1_4*	100	789/789	30	29373..30161	aminoglycoside
i10	2015	*aph(3'')-Ib_5*	100	804/804	169	6154..6957	aminoglycoside
i10	2015	*aph(6)-Id_1*	100	837/837	169	5318..6154	aminoglycoside
i10	2015	*blaCTX-M-15_1*	100	876/876	23	34745..35620	beta-lactam
i10	2015	*dfrA1_8*	100	474/474	30	30838..31311	trimethoprim
i10	2015	*mdf(A)_1*	98.46	1233/1233	53	2964..4191	macrolide
i10	2015	*sul2_2*	100	816/816	169	7018..7833	sulphonamide
i10	2015	*tet(A)_6*	100	1275/1275	169	2783..4057	tetracycline
i11	2015	*aadA1_4*	100	789/789	50	3539..4327	aminoglycoside
i11	2015	*aph(3'')-Ib_5*	100	804/804	161	1527..2330	aminoglycoside

i11	2015	*aph(6)-Id_1*	100	837/837	161	691..1527	aminoglycoside
i11	2015	*blaCTX-M-15_1*	100	876/876	24	34736..35611	beta-lactam
i11	2015	*dfrA1_8*	100	474/474	50	2389..2862	trimethoprim
i11	2015	*mdf(A)_1*	98.46	1233/1233	48	2966..4193	macrolide
i11	2015	*sul2_2*	100	816/816	161	2391..3206	sulphonamide
i11	2015	*tet(A)_6*	100	1275/1275	161	6557..7831	tetracycline
i12	2015	*aph(3'')-Ib_5*	99.88	804/804	162	6240..7043	aminoglycoside
i12	2015	*aph(6)-Id_1*	99.88	837/837	162	5404..6240	aminoglycoside
i12	2015	*blaCTX-M-15_1*	100	876/876	1	4981..5856	beta-lactam
i12	2015	*blaTEM-1B_1*	99.88	861/861	1	1299..2159	beta-lactam
i12	2015	*dfrA1_8*	100	474/474	205	2384..2857	trimethoprim
i12	2015	*mdf(A)_1*	98.38	1233/1233	136	6783..8010	macrolide
i12	2015	*sul2_2*	100	816/816	162	7104..7919	sulphonamide
i12	2015	*tet(A)_6*	100	1275/1275	162	2869..4143	tetracycline
i13	2015	*aph(3'')-Ib_5*	99.88	804/804	167	194..997	aminoglycoside
i13	2015	*aph(6)-Id_4*	99.87	770/831	167	7737..8506	aminoglycoside
i13	2015	*blaCTX-M-15_1*	100	876/876	285	1737..2612	beta-lactam
i13	2015	*dfrA1_8*	100	474/474	187	4653..5126	trimethoprim
i13	2015	*mdf(A)_1*	98.46	1233/1233	140	6783..8010	macrolide
i13	2015	*qnrS1_1*	100	657/657	105	2131..2787	quinolone
i13	2015	*sul2_2*	100	816/816	167	1058..1873	sulphonamide
i13	2015	*tet(A)_6*	100	1275/1275	167	5202..6476	tetracycline
i14	2015	*aph(3'')-Ib_5*	99.88	804/804	158	5528..6331	aminoglycoside
i14	2015	*aph(6)-Id_1*	100	837/837	158	4692..5528	aminoglycoside
i14	2015	*blaCMY-2_1*	100	1146/1146	3	30771..31916	beta-lactam
i14	2015	*dfrA1_8*	99.79	474/474	39	2387..2860	trimethoprim
i14	2015	*mdf(A)_1*	98.46	1233/1233	133	2965..4192	macrolide

i14	2015	*sul2_2*	100	816/816	158	6392..7207	sulphonamide
i14	2015	*tet(A)_6*	100	1275/1275	158	2157..3431	tetracycline
i15	2015	*blaCTX-M-15_1*	100	876/876	1	35491..36366	beta-lactam
i15	2015	*dfrA1_8*	99.79	474/474	31	29986..30459	trimethoprim
i15	2015	*mdf(A)_1*	98.46	1233/1233	136	6783..8010	macrolide
i15	2015	*qnrS1_1*	100	657/657	1	41007..41663	quinolone
i16	2015	*blaCTX-M-15_1*	100	876/876	1	56314..57189	beta-lactam
i16	2015	*dfrA1_8*	99.79	474/474	36	2388..2861	trimethoprim
i16	2015	*mdf(A)_1*	98.46	1233/1233	133	6783..8010	macrolide
i16	2015	*qnrS1_1*	100	657/657	1	51017..51673	quinolone
i17	2015	*aac(3)-IId_1*	99.88	861/861	170	117..977	aminoglycoside
i17	2015	*aph(3'')-Ib_5*	99.88	804/804	164	1055..1858	aminoglycoside
i17	2015	*aph(6)-Id_1*	100	837/837	164	219..1055	aminoglycoside
i17	2015	*blaCTX-M-15_1*	100	876/876	188	323..1198	beta-lactam
i17	2015	*blaTEM-1B_1*	100	861/861	170	5949..6809	beta-lactam
i17	2015	*dfrA1_8*	99.79	474/474	36	29986..30459	trimethoprim
i17	2015	*mdf(A)_1*	98.46	1233/1233	131	6783..8010	macrolide
i17	2015	*sul2_2*	100	816/816	164	1919..2734	sulphonamide
i17	2015	*tet(A)_6*	100	1275/1275	164	6085..7359	tetracycline
i18	2015	*aac(3)-IId_1*	99.88	861/861	1	67806..68666	aminoglycoside
i18	2015	*aph(3'')-Ib_5*	99.88	804/804	162	871..1674	aminoglycoside
i18	2015	*aph(6)-Id_1*	100	837/837	162	35..871	aminoglycoside
i18	2015	*blaCTX-M-15_1*	100	876/876	1	69851..70726	beta-lactam
i18	2015	*blaTEM-1B_1*	100	861/861	1	61974..62834	beta-lactam
i18	2015	*dfrA1_8*	99.79	474/474	37	2387..2860	trimethoprim
i18	2015	*mdf(A)_1*	98.46	1233/1233	136	6783..8010	macrolide
i18	2015	*sul2_2*	100	816/816	162	1735..2550	sulphonamide

i18	2015	*tet(A)_6*	100	1275/1275	162	5901..7175	tetracycline
i19	2015	*aph(3'')-Ib_5*	99.88	804/804	163	3442..4245	aminoglycoside
i19	2015	*aph(6)-Id_1*	100	837/837	163	4245..5081	aminoglycoside
i19	2015	*blaCTX-M-55_1*	100	876/876	1	63061..63936	beta-lactam
i19	2015	*dfrA1_8*	99.79	474/474	157	2384..2857	trimethoprim
i19	2015	*mdf(A)_1*	98.46	1233/1233	134	2965..4192	macrolide
i19	2015	*sul2_2*	100	816/816	163	2566..3381	sulphonamide
i19	2015	*tet(A)_6*	100	1275/1275	163	6342..7616	tetracycline
i20	2015	*aph(3'')-Ib_5*	99.88	804/804	160	3928..4731	aminoglycoside
i20	2015	*aph(6)-Id_1*	100	837/837	160	3092..3928	aminoglycoside
i20	2015	*blaCTX-M-55_1*	100	876/876	2	21497..22372	beta-lactam
i20	2015	*dfrA1_8*	99.79	474/474	155	6090..6563	trimethoprim
i20	2015	*mdf(A)_1*	98.46	1233/1233	134	2965..4192	macrolide
i20	2015	*sul2_2*	100	816/816	160	4792..5607	sulphonamide
i20	2015	*tet(A)_6*	100	1275/1275	160	557..1831	tetracycline
i21	2015	*aph(3'')-Ib_5*	99.88	804/804	160	5846..6649	aminoglycoside
i21	2015	*aph(6)-Id_1*	100	837/837	160	5010..5846	aminoglycoside
i21	2015	*blaCTX-M-55_1*	100	876/876	2	62444..63319	beta-lactam
i21	2015	*dfrA1_8*	99.79	474/474	154	2384..2857	trimethoprim
i21	2015	*mdf(A)_1*	98.46	1233/1233	133	2965..4192	macrolide
i21	2015	*sul2_2*	100	816/816	160	6710..7525	sulphonamide
i21	2015	*tet(A)_6*	100	1275/1275	160	2475..3749	tetracycline
i22	2015	*aac(3)-IId_1*	99.88	861/861	1	24791..25651	aminoglycoside
i22	2015	*aph(3'')-Ib_5*	99.88	804/804	161	6199..7002	aminoglycoside
i22	2015	*aph(6)-Id_1*	100	837/837	161	7002..7838	aminoglycoside
i22	2015	*blaCTX-M-15_1*	100	876/876	1	22731..23606	beta-lactam
i22	2015	*blaTEM-1B_1*	100	861/861	1	30623..31483	beta-lactam

i22	2015	*dfrA1_8*	99.79	474/474	39	2387..2860	trimethoprim
i22	2015	*mdf(A)_1*	98.46	1233/1233	135	2965..4192	macrolide
i22	2015	*sul2_2*	100	816/816	161	5323..6138	sulphonamide
i22	2015	*tet(A)_6*	100	1275/1275	161	698..1972	tetracycline
i23	2015	*aph(3'')-Ib_5*	99.88	804/804	164	1410..2213	aminoglycoside
i23	2015	*aph(6)-Id_1*	100	837/837	164	2213..3049	aminoglycoside
i23	2015	*blaCTX-M-15_1*	100	876/876	1	40388..41263	beta-lactam
i23	2015	*dfrA1_8*	99.79	474/474	158	6438..6911	trimethoprim
i23	2015	*mdf(A)_1*	98.46	1233/1233	134	6783..8010	macrolide
i23	2015	*qnrS1_1*	100	657/657	1	35091..35747	quinolone
i23	2015	*sul2_2*	100	816/816	164	534..1349	sulphonamide
i23	2015	*tet(A)_6*	100	1275/1275	164	4310..5584	tetracycline
i24	2016	*aadA1_4*	100	789/789	38	3538..4326	aminoglycoside
i24	2016	*aph(3'')-Ib_5*	100	804/804	159	6381..7184	aminoglycoside
i24	2016	*aph(6)-Id_1*	100	837/837	159	5545..6381	aminoglycoside
i24	2016	*blaCTX-M-3_1*	100	876/876	1	18812..19687	beta-lactam
i24	2016	*dfrA1_8*	100	474/474	38	2388..2861	trimethoprim
i24	2016	*mdf(A)_1*	98.46	1233/1233	30	2966..4193	macrolide
i24	2016	*sul2_2*	100	816/816	159	7245..8060	sulphonamide
i24	2016	*tet(A)_6*	100	1275/1275	159	3010..4284	tetracycline
i25	2016	*aph(3'')-Ib_5*	99.88	804/804	157	5117..5920	aminoglycoside
i25	2016	*aph(6)-Id_1*	100	837/837	157	4281..5117	aminoglycoside
i25	2016	*blaCTX-M-15_1*	100	876/876	1	9024..9899	beta-lactam
i25	2016	*dfrA1_8*	99.79	474/474	40	2387..2860	trimethoprim
i25	2016	*mdf(A)_1*	98.46	1233/1233	133	2965..4192	macrolide
i25	2016	*qnrS1_1*	100	657/657	1	3727..4383	quinolone
i25	2016	*sul2_2*	100	816/816	157	5981..6796	sulphonamide

i25	2016	*tet(A)_6*	100	1275/1275	157	1746..3020	tetracycline
i26	2016	*aph(3'')-Ib_5*	99.88	804/804	166	2540..3343	aminoglycoside
i26	2016	*aph(6)-Id_1*	100	837/837	166	3343..4179	aminoglycoside
i26	2016	*blaCTX-M-15_1*	100	876/876	268	1737..2612	beta-lactam
i26	2016	*dfrA1_8*	100	474/474	182	4653..5126	trimethoprim
i26	2016	*mdf(A)_1*	98.46	1233/1233	140	6783..8010	macrolide
i26	2016	*qnrS1_1*	100	657/657	116	11822..12478	quinolone
i26	2016	*sul2_2*	100	816/816	166	1664..2479	sulphonamide
i26	2016	*tet(A)_6*	100	1275/1275	166	5440..6714	tetracycline
i27	2016	*aph(3'')-Ib_5*	99.88	804/804	165	6359..7162	aminoglycoside
i27	2016	*aph(6)-Id_1*	100	837/837	165	5523..6359	aminoglycoside
i27	2016	*blaCTX-M-55_1*	100	876/876	64	21605..22480	beta-lactam
i27	2016	*dfrA1_8*	99.79	474/474	160	6090..6563	trimethoprim
i27	2016	*mdf(A)_1*	98.46	1233/1233	138	6783..8010	macrolide
i27	2016	*sul2_2*	100	816/816	165	7223..8038	sulphonamide
i27	2016	*tet(A)_6*	100	1275/1275	165	2988..4262	tetracycline
i28	2016	*aph(3'')-Ib_5*	99.88	804/804	160	7706..8509	aminoglycoside
i28	2016	*aph(6)-Id_1*	100	837/837	160	108..944	aminoglycoside
i28	2016	*blaCTX-M-55_1*	100	876/876	2	61776..62651	beta-lactam
i28	2016	*dfrA1_8*	99.79	474/474	39	29986..30459	trimethoprim
i28	2016	*mdf(A)_1*	98.46	1233/1233	134	6783..8010	macrolide
i28	2016	*sul2_2*	100	816/816	160	6830..7645	sulphonamide
i28	2016	*tet(A)_6*	100	1275/1275	160	2205..3479	tetracycline
i29	2016	*aph(3'')-Ib_5*	99.88	804/804	163	5930..6733	aminoglycoside
i29	2016	*aph(6)-Id_1*	100	837/837	163	5094..5930	aminoglycoside
i29	2016	*blaCTX-M-15_1*	100	876/876	13	6069..6944	beta-lactam
i29	2016	*dfrA1_8*	100	474/474	40	29987..30460	trimethoprim

i29	2016	*mdf(A)_1*	98.46	1233/1233	137	6783..8010	macrolide
i29	2016	*sul2_2*	100	816/816	163	6794..7609	sulphonamide
i29	2016	*tet(A)_6*	100	1275/1275	163	2559..3833	tetracycline
i30	2017	*aac(3)-IId_1*	99.88	861/861	1	68725..69585	aminoglycoside
i30	2017	*aadA1_4*	100	789/789	38	3538..4326	aminoglycoside
i30	2017	*aph(3'')-Ib_5*	100	804/804	161	6381..7184	aminoglycoside
i30	2017	*aph(6)-Id_1*	100	837/837	161	5545..6381	aminoglycoside
i30	2017	*blaCTX-M-15_1*	100	876/876	1	70755..71630	beta-lactam
i30	2017	*blaTEM-1B_1*	100	861/861	1	62893..63753	beta-lactam
i30	2017	*dfrA1_8*	100	474/474	38	2388..2861	trimethoprim
i30	2017	*mdf(A)_1*	98.46	1233/1233	30	2966..4193	macrolide
i30	2017	*sul2_2*	100	816/816	161	7245..8060	sulphonamide
i30	2017	*tet(A)_6*	100	1275/1275	161	3010..4284	tetracycline
i31	2017	*aadA1_4*	100	789/789	38	29373..30161	aminoglycoside
i31	2017	*aph(3'')-Ib_5*	100	804/804	164	6413..7216	aminoglycoside
i31	2017	*aph(6)-Id_1*	99.16	837/837	164	5584..6413	aminoglycoside
i31	2017	*blaCTX-M-3_1*	100	876/876	1	65172..66047	beta-lactam
i31	2017	*dfrA1_8*	100	474/474	38	30838..31311	trimethoprim
i31	2017	*mdf(A)_1*	98.46	1233/1233	30	33353..34580	macrolide
i31	2017	*sul2_2*	100	816/816	164	7277..8092	sulphonamide
i31	2017	*tet(A)_6*	100	1275/1275	164	3049..4323	tetracycline
i32	2017	*aadA1_4*	100	789/789	179	3589..4377	aminoglycoside
i32	2017	*blaCTX-M-15_1*	100	876/876	79	16604..17479	beta-lactam
i32	2017	*dfrA1_8*	100	474/474	179	5054..5527	trimethoprim
i32	2017	*mdf(A)_1*	98.46	1233/1233	165	3479..4709	macrolide
i32	2017	*tet(B)_2*	100	1206/1206	203	2508..3713	tetracycline
i33	2017	*aac(3)-IId_1*	99.88	861/861	90	16021..16881	aminoglycoside

i33	2017	*aadA1_4*	100	789/789	37	3538..4326	aminoglycoside
i33	2017	*blaCTX-M-15_1*	100	876/876	326	196..1071	beta-lactam
i33	2017	*blaTEM-1B_1*	100	861/861	90	10189..11049	beta-lactam
i33	2017	*dfrA1_8*	100	474/474	37	2388..2861	trimethoprim
i33	2017	*mdf(A)_1*	98.46	1233/1233	30	33353..34580	macrolide
i33	2017	*qnrS1_1*	100	657/657	112	1547..2203	quinolone
i34	2017	*aadA1_4*	100	789/789	37	3538..4326	aminoglycoside
i34	2017	*aph(3'')-Ib_5*	100	804/804	159	320..1123	aminoglycoside
i34	2017	*aph(6)-Id_4*	100	644/831	159	7885..8528	aminoglycoside
i34	2017	*blaCTX-M-15_1*	100	876/876	11	21816..22691	beta-lactam
i34	2017	*dfrA1_8*	100	474/474	37	2388..2861	trimethoprim
i34	2017	*mdf(A)_1*	98.46	1233/1233	30	2966..4193	macrolide
i34	2017	*sul2_2*	100	816/816	159	1184..1999	sulphonamide
i34	2017	*tet(A)_6*	100	1275/1275	159	5350..6624	tetracycline
i35	2017	*aadA1_4*	100	789/789	37	3538..4326	aminoglycoside
i35	2017	*aph(3'')-Ib_5*	100	804/804	163	412..1215	aminoglycoside
i35	2017	*aph(6)-Id_1*	100	837/837	163	1215..2051	aminoglycoside
i35	2017	*blaCTX-M-15_1*	100	876/876	1	82976..83851	beta-lactam
i35	2017	*dfrA1_8*	100	474/474	37	2388..2861	trimethoprim
i35	2017	*mdf(A)_1*	98.46	1233/1233	30	2966..4193	macrolide
i35	2017	*qnrS1_1*	100	657/657	1	88492..89148	quinolone
i35	2017	*sul2_13*	100	592/816	163	7926..8517	sulphonamide
i35	2017	*sul2_19*	100	592/816	163	7926..8517	sulphonamide
i35	2017	*sul2_2*	100	592/816	163	7926..8517	sulphonamide
i35	2017	*tet(A)_6*	100	1275/1275	163	3312..4586	tetracycline
i36	2017	*aac(3)-IId_1*	99.88	861/861	178	6028..6888	aminoglycoside
i36	2017	*aadA2_2*	100	792/792	144	1410..2201	aminoglycoside

i36	2017	*aph(3'')-Ib_5*	99.88	804/804	161	4696..5499	aminoglycoside
i36	2017	*aph(6)-Id_1*	100	837/837	161	3860..4696	aminoglycoside
i36	2017	*blaCTX-M-15_1*	100	876/876	88	5270..6145	beta-lactam
i36	2017	*blaTEM-1B_1*	100	861/861	178	196..1056	beta-lactam
i36	2017	*dfrA12_8*	100	498/498	144	505..1002	trimethoprim
i36	2017	*dfrA1_8*	99.79	474/474	38	2387..2860	trimethoprim
i36	2017	*mdf(A)_1*	98.46	1233/1233	132	6783..8010	macrolide
i36	2017	*mph(A)_1*	100	906/906	144	8856..9761	macrolide
i36	2017	*sul1_2*	100	840/840	144	2706..3545	sulphonamide
i36	2017	*sul2_2*	100	816/816	161	5560..6375	sulphonamide
i36	2017	*tet(A)_6*	100	1275/1275	161	1325..2599	tetracycline
i37	2017	*aadA1_4*	100	789/789	37	3538..4326	aminoglycoside
i37	2017	*aph(3'')-Ib_5*	100	804/804	165	4542..5345	aminoglycoside
i37	2017	*aph(6)-Id_1*	100	837/837	165	5345..6181	aminoglycoside
i37	2017	*blaCTX-M-15_1*	100	876/876	272	347..1222	beta-lactam
i37	2017	*dfrA1_8*	100	474/474	37	2388..2861	trimethoprim
i37	2017	*mdf(A)_1*	98.46	1233/1233	29	33353..34580	macrolide
i37	2017	*qnrS1_1*	100	657/657	113	11633..12289	quinolone
i37	2017	*sul2_2*	100	816/816	165	3666..4481	sulphonamide
i37	2017	*tet(A)_6*	100	1076/1275	165	7442..8517	tetracycline
i38	2017	*aadA1_4*	100	789/789	37	29373..30161	aminoglycoside
i38	2017	*aph(3'')-Ib_5*	100	804/804	158	1160..1963	aminoglycoside
i38	2017	*aph(6)-Id_1*	100	837/837	158	1963..2799	aminoglycoside
i38	2017	*blaCTX-M-15_1*	100	876/876	1	50259..51134	beta-lactam
i38	2017	*dfrA1_8*	100	474/474	37	30838..31311	trimethoprim
i38	2017	*mdf(A)_1*	98.46	1233/1233	30	2966..4193	macrolide
i38	2017	*sul2_2*	100	816/816	158	284..1099	sulphonamide

i38	2017	*tet(A)_6*	100	1275/1275	158	4060..5334	tetracycline
i39	2017	*aadA1_4*	100	789/789	38	3538..4326	aminoglycoside
i39	2017	*blaCTX-M-15_1*	100	876/876	2	66475..67350	beta-lactam
i39	2017	*blaTEM-1B_1*	100	861/861	2	62210..63070	beta-lactam
i39	2017	*dfrA1_8*	100	474/474	38	2388..2861	trimethoprim
i39	2017	*mdf(A)_1*	98.46	1233/1233	31	2966..4193	macrolide
i40	2017	*aadA1_5*	100	792/792	325	134..925	aminoglycoside
i40	2017	*aph(3'')-Ib_5*	99.88	804/804	120	510..1313	aminoglycoside
i40	2017	*aph(6)-Id_1*	100	837/837	120	1313..2149	aminoglycoside
i40	2017	*blaCTX-M-15_1*	100	876/876	1	81835..82710	beta-lactam
i40	2017	*blaTEM-187_1*	98.92	553/858	408	1..553	beta-lactam
i40	2017	*blaTEM-205_1*	98.92	553/858	408	1..553	beta-lactam
i40	2017	*blaTEM-21_1*	98.92	553/858	408	1..553	beta-lactam
i40	2017	*blaTEM-22_1*	98.92	553/858	408	1..553	beta-lactam
i40	2017	*dfrA1_10*	99.79	474/474	423	22..495	trimethoprim
i40	2017	*dfrA1_8*	99.79	474/474	423	22..495	trimethoprim
i40	2017	*dfrA1_9*	99.79	474/474	423	22..495	trimethoprim
i40	2017	*mdf(A)_1*	98.46	1233/1233	136	6783..8010	macrolide
i40	2017	*qnrS1_1*	100	657/657	1	87351..88007	quinolone
i40	2017	*sul2_2*	100	816/816	120	8035..8850	sulphonamide
i40	2017	*tet(A)_6*	100	1275/1275	120	3410..4684	tetracycline
i41	2017	*aph(3'')-Ib_5*	99.88	804/804	165	2969..3772	aminoglycoside
i41	2017	*aph(6)-Id_1*	100	837/837	165	3772..4608	aminoglycoside
i41	2017	*blaCTX-M-15_1*	100	876/876	39	1368..2243	beta-lactam
i41	2017	*dfrA1_8*	99.79	474/474	38	2387..2860	trimethoprim
i41	2017	*mdf(A)_1*	98.46	1233/1233	136	6783..8010	macrolide
i41	2017	*sul2_2*	100	816/816	165	2093..2908	sulphonamide

i41	2017	*tet(A)_6*	100	1275/1275	165	5869..7143	tetracycline
i42	2017	*aadA1_4*	100	789/789	37	3538..4326	aminoglycoside
i42	2017	*aph(3'')-Ib_5*	100	804/804	162	2421..3224	aminoglycoside
i42	2017	*aph(6)-Id_1*	100	837/837	162	3224..4060	aminoglycoside
i42	2017	*blaCTX-M-15_1*	100	876/876	274	347..1222	beta-lactam
i42	2017	*dfrA1_8*	100	474/474	37	2388..2861	trimethoprim
i42	2017	*mdf(A)_1*	98.46	1233/1233	29	2966..4193	macrolide
i42	2017	*qnrS1_1*	100	657/657	112	11822..12478	quinolone
i42	2017	*sul2_2*	100	816/816	162	1545..2360	sulphonamide
i42	2017	*tet(A)_6*	100	1275/1275	162	5321..6595	tetracycline
i43	2017	*aac(3)-IId_1*	99.88	861/861	21	15935..16795	aminoglycoside
i43	2017	*aph(3'')-Ib_5*	99.88	804/804	164	1278..2081	aminoglycoside
i43	2017	*aph(6)-Id_1*	100	837/837	164	2081..2917	aminoglycoside
i43	2017	*blaCTX-M-15_1*	100	876/876	21	17980..18855	beta-lactam
i43	2017	*blaTEM-1B_1*	100	861/861	21	10103..10963	beta-lactam
i43	2017	*dfrA1_8*	99.79	474/474	39	2387..2860	trimethoprim
i43	2017	*mdf(A)_1*	98.46	1233/1233	137	6783..8010	macrolide
i43	2017	*sul2_2*	100	816/816	164	402..1217	sulphonamide
i43	2017	*tet(A)_6*	100	1275/1275	164	4178..5452	tetracycline
i44	2017	*aadA1_4*	100	601/789	410	11..611	aminoglycoside
i44	2017	*aph(3'')-Ib_5*	100	804/804	100	12384..13187	aminoglycoside
i44	2017	*aph(6)-Id_1*	100	837/837	100	13187..14023	aminoglycoside
i44	2017	*blaCTX-M-15_1*	100	876/876	282	1737..2612	beta-lactam
i44	2017	*dfrA1_10*	99.79	474/474	415	22..495	trimethoprim
i44	2017	*dfrA1_8*	99.79	474/474	415	22..495	trimethoprim
i44	2017	*dfrA1_9*	99.79	474/474	415	22..495	trimethoprim
i44	2017	*mdf(A)_1*	98.46	1233/1233	30	33353..34580	macrolide

i44	2017	*qnrS1_1*	100	657/657	114	1547..2203	quinolone
i44	2017	*sul1_39*	100	811/828	334	515..1325	sulphonamide
i44	2017	*sul2_2*	100	816/816	100	11508..12323	sulphonamide
i44	2017	*tet(A)_6*	100	1275/1275	100	6894..8168	tetracycline
i45	2017	*aph(3'')-Ib_5*	99.88	804/804	232	156..959	aminoglycoside
i45	2017	*aph(6)-Id_1*	100	809/837	232	959..1767	aminoglycoside
i45	2017	*blaCTX-M-1_1*	100	876/876	24	23912..24787	beta-lactam
i45	2017	*dfrA1_8*	100	474/474	39	29965..30438	trimethoprim
i45	2017	*mdf(A)_1*	98.46	1233/1233	137	2965..4192	macrolide
i45	2017	*sul2_2*	100	816/816	344	97..912	sulphonamide
i45	2017	*tet(A)_6*	100	1127/1275	281	877..2003	tetracycline
i46	2017	*aadA1_4*	100	789/789	37	3538..4326	aminoglycoside
i46	2017	*aph(3'')-Ib_5*	100	804/804	163	6083..6886	aminoglycoside
i46	2017	*aph(6)-Id_1*	100	837/837	163	5247..6083	aminoglycoside
i46	2017	*blaCTX-M-15_1*	100	876/876	276	347..1222	beta-lactam
i46	2017	*dfrA1_8*	100	474/474	37	2388..2861	trimethoprim
i46	2017	*mdf(A)_1*	98.46	1233/1233	29	2966..4193	macrolide
i46	2017	*qnrS1_1*	100	657/657	112	1547..2203	quinolone
i46	2017	*sul2_2*	100	816/816	163	6947..7762	sulphonamide
i46	2017	*tet(A)_6*	100	1275/1275	163	2712..3986	tetracycline
i47	2017	*aph(3'')-Ib_5*	99.88	804/804	231	3062..3865	aminoglycoside
i47	2017	*aph(6)-Id_1*	100	809/837	231	2254..3062	aminoglycoside
i47	2017	*blaCTX-M-1_1*	100	876/876	1	23412..24287	beta-lactam
i47	2017	*dfrA1_8*	100	474/474	38	29965..30438	trimethoprim
i47	2017	*mdf(A)_1*	98.46	1233/1233	135	2965..4192	macrolide
i47	2017	*sul2_2*	100	816/816	339	33..848	sulphonamide
i47	2017	*tet(A)_6*	100	1127/1275	279	877..2003	tetracycline

i48	2017	*aph(3'')-Ib_5*	99.88	804/804	234	3062..3865	aminoglycoside
i48	2017	*aph(6)-Id_1*	100	809/837	234	2254..3062	aminoglycoside
i48	2017	*blaCTX-M-1_1*	100	876/876	1	23912..24787	beta-lactam
i48	2017	*dfrA1_8*	100	474/474	37	2388..2861	trimethoprim
i48	2017	*mdf(A)_1*	98.46	1233/1233	135	6783..8010	macrolide
i48	2017	*sul2_2*	100	816/816	344	97..912	sulphonamide
i48	2017	*tet(A)_6*	100	1127/1275	281	877..2003	tetracycline
i49	2017	*aadA5_1*	100	789/789	145	7799..8587	aminoglycoside
i49	2017	*aph(3'')-Ib_5*	99.88	804/804	163	5250..6053	aminoglycoside
i49	2017	*aph(6)-Id_1*	100	837/837	163	4414..5250	aminoglycoside
i49	2017	*blaCTX-M-15_1*	100	876/876	238	2826..3701	beta-lactam
i49	2017	*dfrA17_1*	100	474/474	145	8718..9191	trimethoprim
i49	2017	*dfrA1_8*	100	474/474	180	2387..2860	trimethoprim
i49	2017	*mdf(A)_1*	98.46	1233/1233	138	6783..8010	macrolide
i49	2017	*mph(A)_1*	100	906/906	145	197..1102	macrolide
i49	2017	*qnrS1_1*	100	657/657	132	9076..9732	quinolone
i49	2017	*sul1_2*	100	840/840	145	6413..7252	sulphonamide
i49	2017	*sul2_2*	100	816/816	163	6114..6929	sulphonamide
i49	2017	*tet(A)_6*	100	1275/1275	163	1879..3153	tetracycline
i50	2017	*aadA5_1*	100	789/789	149	1520..2308	aminoglycoside
i50	2017	*aph(3'')-Ib_5*	99.88	804/804	168	6160..6963	aminoglycoside
i50	2017	*aph(6)-Id_1*	100	837/837	168	5324..6160	aminoglycoside
i50	2017	*blaCTX-M-15_1*	100	876/876	251	264..1139	beta-lactam
i50	2017	*dfrA17_1*	100	474/474	149	916..1389	trimethoprim
i50	2017	*dfrA1_8*	100	474/474	184	4653..5126	trimethoprim
i50	2017	*mdf(A)_1*	98.46	1233/1233	141	6783..8010	macrolide
i50	2017	*mph(A)_1*	100	906/906	149	9005..9910	macrolide

i50	2017	*qnrS1_1*	100	657/657	135	1547..2203	quinolone
i50	2017	*sul1_2*	100	840/840	149	2855..3694	sulphonamide
i50	2017	*sul2_2*	100	816/816	168	7024..7839	sulphonamide
i50	2017	*tet(A)_6*	100	1275/1275	168	2789..4063	tetracycline
i51	2017	*aadA1_4*	100	789/789	37	29373..30161	aminoglycoside
i51	2017	*aph(3'')-Ib_5*	100	804/804	163	4063..4866	aminoglycoside
i51	2017	*aph(6)-Id_1*	100	837/837	163	3227..4063	aminoglycoside
i51	2017	*blaCTX-M-55_1*	100	876/876	91	7773..8648	beta-lactam
i51	2017	*dfrA1_8*	100	474/474	37	30838..31311	trimethoprim
i51	2017	*mdf(A)_1*	98.46	1233/1233	31	33219..34446	macrolide
i51	2017	*mph(A)_1*	100	906/906	91	226..1131	macrolide
i51	2017	*sul2_2*	100	816/816	163	4927..5742	sulphonamide
i51	2017	*tet(A)_6*	100	1275/1275	163	692..1966	tetracycline
i52	2017	*aph(3'')-Ib_5*	99.88	804/804	309	156..959	aminoglycoside
i52	2017	*aph(6)-Id_1*	100	809/837	309	959..1767	aminoglycoside
i52	2017	*blaCTX-M-1_1*	100	876/876	5	2376..3251	beta-lactam
i52	2017	*dfrA1_8*	100	474/474	40	29965..30438	trimethoprim
i52	2017	*mdf(A)_1*	98.46	1233/1233	136	6783..8010	macrolide
i52	2017	*sul2_2*	100	816/816	377	97..912	sulphonamide
i52	2017	*tet(A)_6*	100	1127/1275	303	1..1127	tetracycline
i53	2017	*aph(3'')-Ib_5*	99.88	804/804	234	3062..3865	aminoglycoside
i53	2017	*aph(6)-Id_1*	100	809/837	234	2254..3062	aminoglycoside
i53	2017	*blaCTX-M-1_1*	100	876/876	5	2428..3303	beta-lactam
i53	2017	*dfrA1_8*	100	474/474	38	2387..2860	trimethoprim
i53	2017	*mdf(A)_1*	98.46	1233/1233	135	2965..4192	macrolide
i53	2017	*sul2_2*	100	816/816	345	97..912	sulphonamide
i53	2017	*tet(A)_6*	100	1127/1275	283	877..2003	tetracycline

i54	2017	*aadA5_1*	100	789/789	144	1520..2308	aminoglycoside
i54	2017	*aph(3'')-Ib_5*	99.88	804/804	164	151..954	aminoglycoside
i54	2017	*aph(6)-Id_4*	99.88	813/831	164	7705..8517	aminoglycoside
i54	2017	*blaCTX-M-15_1*	100	876/876	237	264..1139	beta-lactam
i54	2017	*dfrA17_1*	100	474/474	144	916..1389	trimethoprim
i54	2017	*dfrA1_8*	100	474/474	179	2387..2860	trimethoprim
i54	2017	*mdf(A)_1*	98.46	1233/1233	137	2965..4192	macrolide
i54	2017	*mph(A)_1*	100	906/906	144	9005..9910	macrolide
i54	2017	*qnrS1_1*	100	657/657	131	1547..2203	quinolone
i54	2017	*sul1_2*	100	840/840	144	2855..3694	sulphonamide
i54	2017	*sul2_2*	100	816/816	164	1015..1830	sulphonamide
i54	2017	*tet(A)_6*	100	1275/1275	164	5170..6444	tetracycline
i55	2017	*aph(3'')-Ib_5*	99.88	804/804	234	3062..3865	aminoglycoside
i55	2017	*aph(6)-Id_1*	100	809/837	234	2254..3062	aminoglycoside
i55	2017	*blaCTX-M-1_1*	100	876/876	11	28957..29832	beta-lactam
i55	2017	*dfrA1_8*	100	474/474	39	2388..2861	trimethoprim
i55	2017	*mdf(A)_1*	98.46	1233/1233	137	2965..4192	macrolide
i55	2017	*sul2_2*	100	816/816	341	97..912	sulphonamide
i55	2017	*tet(A)_6*	100	1127/1275	280	1..1127	tetracycline
i56	2017	*aph(3'')-Ib_5*	99.88	804/804	232	3062..3865	aminoglycoside
i56	2017	*aph(6)-Id_1*	100	809/837	232	2254..3062	aminoglycoside
i56	2017	*blaCTX-M-1_1*	100	876/876	237	1927..2802	beta-lactam
i56	2017	*dfrA1_8*	100	474/474	38	29965..30438	trimethoprim
i56	2017	*mdf(A)_1*	98.46	1233/1233	136	6783..8010	macrolide
i56	2017	*sul2_2*	100	816/816	346	97..912	sulphonamide
i56	2017	*tet(A)_6*	100	1127/1275	280	1..1127	tetracycline
i57	2017	*aadA5_1*	100	789/789	147	7799..8587	aminoglycoside

i57	2017	*aph(3'')-Ib_5*	99.88	804/804	166	7458..8261	aminoglycoside
i57	2017	*aph(6)-Id_1*	100	837/837	166	6622..7458	aminoglycoside
i57	2017	*blaCTX-M-15_1*	100	876/876	248	2826..3701	beta-lactam
i57	2017	*dfrA17_1*	100	474/474	147	8718..9191	trimethoprim
i57	2017	*dfrA1_8*	100	474/474	185	2385..2858	trimethoprim
i57	2017	*mdf(A)_1*	98.46	1233/1233	139	2965..4192	macrolide
i57	2017	*mph(A)_1*	100	906/906	147	197..1102	macrolide
i57	2017	*qnrS1_1*	100	657/657	133	1547..2203	quinolone
i57	2017	*sul1_2*	100	840/840	147	6413..7252	sulphonamide
i57	2017	*sul2_2*	100	747/816	166	1..747	sulphonamide
i57	2017	*tet(A)_6*	100	1275/1275	166	4087..5361	tetracycline
i58	2018	*aadA5_1*	100	789/789	145	1520..2308	aminoglycoside
i58	2018	*aph(3'')-Ib_5*	99.87	787/804	163	1..787	aminoglycoside
i58	2018	*aph(6)-Id_1*	100	837/837	163	787..1623	aminoglycoside
i58	2018	*blaCTX-M-15_1*	100	876/876	236	2826..3701	beta-lactam
i58	2018	*dfrA17_1*	100	474/474	145	916..1389	trimethoprim
i58	2018	*dfrA1_8*	100	474/474	180	2387..2860	trimethoprim
i58	2018	*mdf(A)_1*	98.46	1233/1233	138	2965..4192	macrolide
i58	2018	*mph(A)_1*	100	906/906	145	9005..9910	macrolide
i58	2018	*qnrS1_1*	100	657/657	132	1547..2203	quinolone
i58	2018	*sul1_2*	100	840/840	145	2855..3694	sulphonamide
i58	2018	*sul2_2*	100	816/816	163	7498..8313	sulphonamide
i58	2018	*tet(A)_6*	100	1275/1275	163	2884..4158	tetracycline
i59	2018	*aadA5_1*	100	789/789	150	7799..8587	aminoglycoside
i59	2018	*aph(3'')-Ib_5*	99.88	804/804	168	5250..6053	aminoglycoside
i59	2018	*aph(6)-Id_1*	100	837/837	168	4414..5250	aminoglycoside
i59	2018	*blaCTX-M-15_1*	100	876/876	270	264..1139	beta-lactam

i59	2018	*dfrA17_1*	100	474/474	150	8718..9191	trimethoprim
i59	2018	*dfrA1_8*	100	474/474	189	4653..5126	trimethoprim
i59	2018	*mdf(A)_1*	98.46	1233/1233	140	6783..8010	macrolide
i59	2018	*mph(A)_1*	100	906/906	150	197..1102	macrolide
i59	2018	*qnrS1_1*	100	657/657	134	1547..2203	quinolone
i59	2018	*sul1_2*	100	840/840	150	6413..7252	sulphonamide
i59	2018	*sul2_2*	100	816/816	168	6114..6929	sulphonamide
i59	2018	*tet(A)_6*	100	1275/1275	168	1879..3153	tetracycline
i60	2018	*aadA5_1*	100	789/789	148	1520..2308	aminoglycoside
i60	2018	*aph(3'')-Ib_5*	99.88	804/804	168	2460..3263	aminoglycoside
i60	2018	*aph(6)-Id_1*	100	837/837	168	3263..4099	aminoglycoside
i60	2018	*blaCTX-M-15_1*	100	876/876	243	264..1139	beta-lactam
i60	2018	*dfrA17_1*	100	474/474	148	916..1389	trimethoprim
i60	2018	*dfrA1_8*	100	474/474	183	2385..2858	trimethoprim
i60	2018	*mdf(A)_1*	98.46	1233/1233	139	6783..8010	macrolide
i60	2018	*mph(A)_1*	100	906/906	148	9005..9910	macrolide
i60	2018	*qnrS1_1*	100	657/657	133	1547..2203	quinolone
i60	2018	*sul1_2*	100	840/840	148	2855..3694	sulphonamide
i60	2018	*sul2_2*	100	816/816	168	1584..2399	sulphonamide
i60	2018	*tet(A)_6*	100	1275/1275	168	5360..6634	tetracycline
i61	2018	*aadA5_1*	100	789/789	40	7799..8587	aminoglycoside
i61	2018	*aph(3'')-Ib_5*	99.88	804/804	72	1608..2411	aminoglycoside
i61	2018	*aph(6)-Id_1*	100	837/837	72	2411..3247	aminoglycoside
i61	2018	*blaCTX-M-15_1*	100	876/876	354	2826..3701	beta-lactam
i61	2018	*dfrA17_1*	100	474/474	40	8718..9191	trimethoprim
i61	2018	*dfrA1_8*	100	474/474	236	2162..2635	trimethoprim
i61	2018	*mph(A)_1*	100	906/906	40	197..1102	macrolide

i61	2018	*qnrS1_1*	100	657/657	26	9076..9732	quinolone
i61	2018	*sul1_2*	100	840/840	40	6413..7252	sulphonamide
i61	2018	*sul2_2*	100	816/816	72	732..1547	sulphonamide
i61	2018	*tet(A)_6*	100	1275/1275	72	4508..5782	tetracycline
i62	2018	*aadA1_4*	100	789/789	38	3538..4326	aminoglycoside
i62	2018	*aph(3'')-Ib_5*	100	804/804	162	974..1777	aminoglycoside
i62	2018	*aph(6)-Id_1*	100	837/837	162	138..974	aminoglycoside
i62	2018	*blaCTX-M-3_1*	100	876/876	1	18938..19813	beta-lactam
i62	2018	*dfrA1_8*	100	474/474	38	2388..2861	trimethoprim
i62	2018	*mdf(A)_1*	98.46	1233/1233	30	2966..4193	macrolide
i62	2018	*sul2_2*	100	816/816	162	1838..2653	sulphonamide
i62	2018	*tet(A)_6*	100	1275/1275	162	6004..7278	tetracycline
i63	2018	*blaCTX-M-15_1*	100	876/876	146	270..1145	beta-lactam
i63	2018	*blaTEM-1B_1*	100	861/861	292	291..1151	beta-lactam
i63	2018	*dfrA1_8*	99.79	474/474	36	2387..2860	trimethoprim
i63	2018	*mdf(A)_1*	98.46	1233/1233	136	6783..8010	macrolide
i63	2018	*mph(A)_1*	100	906/906	220	196..1101	macrolide
i63	2018	*qnrS1_1*	100	657/657	146	5786..6442	quinolone
i64	2018	*aph(3'')-Ib_5*	99.88	804/804	165	5535..6338	aminoglycoside
i64	2018	*aph(6)-Id_1*	100	837/837	165	4699..5535	aminoglycoside
i64	2018	*blaCTX-M-15_1*	100	876/876	245	264..1139	beta-lactam
i64	2018	*dfrA1_8*	100	474/474	184	2387..2860	trimethoprim
i64	2018	*mdf(A)_1*	98.46	1233/1233	139	6783..8010	macrolide
i64	2018	*mph(A)_1*	100	906/906	260	197..1102	macrolide
i64	2018	*qnrS1_1*	100	657/657	114	1641..2297	quinolone
i64	2018	*sul2_2*	100	816/816	165	6399..7214	sulphonamide
i64	2018	*tet(A)_6*	100	1275/1275	165	2164..3438	tetracycline

i65	2018	*aadA5_1*	100	789/789	147	7799..8587	aminoglycoside
i65	2018	*aph(3'')-Ib_5*	99.88	804/804	165	4088..4891	aminoglycoside
i65	2018	*aph(6)-Id_1*	100	837/837	165	3252..4088	aminoglycoside
i65	2018	*blaCTX-M-15_1*	100	876/876	238	2826..3701	beta-lactam
i65	2018	*dfrA17_1*	100	474/474	147	8718..9191	trimethoprim
i65	2018	*dfrA1_8*	100	474/474	181	4653..5126	trimethoprim
i65	2018	*mdf(A)_1*	98.46	1233/1233	139	6783..8010	macrolide
i65	2018	*mph(A)_1*	100	906/906	147	197..1102	macrolide
i65	2018	*qnrS1_1*	100	657/657	133	9076..9732	quinolone
i65	2018	*sul1_2*	100	840/840	147	6413..7252	sulphonamide
i65	2018	*sul2_2*	100	816/816	165	4952..5767	sulphonamide
i65	2018	*tet(A)_6*	100	1275/1275	165	717..1991	tetracycline
i66	2018	*blaCTX-M-15_1*	100	876/876	201	347..1222	beta-lactam
i66	2018	*mdf(A)_1*	98.22	1233/1233	36	2157..3383	macrolide
i66	2018	*qnrS1_1*	100	657/657	169	1547..2203	quinolone
i67	2018	*aadA1_4*	100	789/789	37	29373..30161	aminoglycoside
i67	2018	*aph(3'')-Ib_5*	100	804/804	4	52003..52806	aminoglycoside
i67	2018	*aph(6)-Id_1*	100	837/837	4	52806..53642	aminoglycoside
i67	2018	*blaCTX-M-137_1*	92.69	876/876	281	872..1747	beta-lactam
i67	2018	*blaCTX-M-27_1*	100	876/876	281	872..1747	beta-lactam
i67	2018	*dfrA1_8*	100	474/474	37	30838..31311	trimethoprim
i67	2018	*erm(B)_1*	99.86	738/738	214	3538..4275	macrolide
i67	2018	*mdf(A)_1*	98.46	1233/1233	31	2966..4193	macrolide
i67	2018	*mph(A)_1*	100	903/906	227	3290..4192	macrolide
i67	2018	*sul2_2*	100	816/816	4	51127..51942	sulphonamide
i67	2018	*tet(A)_4*	100	1200/1200	84	13662..14861	tetracycline
i68	2018	*aadA1_4*	100	789/789	36	3539..4327	aminoglycoside

i68	2018	*aph(3'')-Ib_5*	100	804/804	4	51584..52387	aminoglycoside
i68	2018	*aph(6)-Id_1*	100	837/837	4	52387..53223	aminoglycoside
i68	2018	*blaCTX-M-137_1*	92.69	876/876	296	872..1747	beta-lactam
i68	2018	*blaCTX-M-27_1*	100	876/876	296	872..1747	beta-lactam
i68	2018	*dfrA1_8*	100	474/474	36	2389..2862	trimethoprim
i68	2018	*erm(B)_1*	99.86	738/738	217	420..1157	macrolide
i68	2018	*mdf(A)_1*	98.46	1233/1233	30	33096..34323	macrolide
i68	2018	*mph(A)_1*	100	903/906	229	3290..4192	macrolide
i68	2018	*sul2_2*	100	816/816	4	50708..51523	sulphonamide
i68	2018	*tet(A)_4*	100	1200/1200	84	4236..5435	tetracycline
i69	2018	*aac(3)-IId_1*	99.88	861/861	204	2757..3617	aminoglycoside
i69	2018	*aadA1_4*	100	789/789	38	3538..4326	aminoglycoside
i69	2018	*aadA5_1*	99.87	789/789	192	2572..3360	aminoglycoside
i69	2018	*blaCTX-M-137_1*	92.58	876/876	1	39851..40726	beta-lactam
i69	2018	*blaCTX-M-14_1*	100	876/876	1	39851..40726	beta-lactam
i69	2018	*dfrA17_1*	100	474/474	192	3491..3964	trimethoprim
i69	2018	*dfrA1_8*	100	474/474	38	2388..2861	trimethoprim
i69	2018	*mdf(A)_1*	98.46	1233/1233	31	2966..4193	macrolide
i69	2018	*mph(A)_1*	100	906/906	244	2385..3290	macrolide
i69	2018	*sul1_2*	100	840/840	192	1186..2025	sulphonamide
i70	2018	*aadA1_4*	100	789/789	37	3538..4326	aminoglycoside
i70	2018	*aph(3'')-Ib_5*	100	804/804	4	20230..21033	aminoglycoside
i70	2018	*aph(6)-Id_1*	100	837/837	4	19394..20230	aminoglycoside
i70	2018	*blaCTX-M-137_1*	92.69	876/876	281	708..1583	beta-lactam
i70	2018	*blaCTX-M-27_1*	100	876/876	281	708..1583	beta-lactam
i70	2018	*dfrA1_8*	100	474/474	37	2388..2861	trimethoprim
i70	2018	*erm(B)_1*	99.86	738/738	214	3538..4275	macrolide

i70	2018	*mdf(A)_1*	98.46	1233/1233	31	2966..4193	macrolide
i70	2018	*mph(A)_1*	100	903/906	227	196..1098	macrolide
i70	2018	*sul2_2*	100	816/816	4	21094..21909	sulphonamide
i70	2018	*tet(A)_4*	100	1200/1200	84	4236..5435	tetracycline
i71	2018	*aadA1_4*	100	789/789	36	3539..4327	aminoglycoside
i71	2018	*aph(3'')-Ib_5*	100	804/804	4	52003..52806	aminoglycoside
i71	2018	*aph(6)-Id_1*	100	837/837	4	52806..53642	aminoglycoside
i71	2018	*blaCTX-M-137_1*	92.69	876/876	278	872..1747	beta-lactam
i71	2018	*blaCTX-M-27_1*	100	876/876	278	872..1747	beta-lactam
i71	2018	*dfrA17_1*	100	288/474	436	131..418	trimethoprim
i71	2018	*dfrA17_10*	100	288/474	436	131..418	trimethoprim
i71	2018	*dfrA17_7*	100	288/474	436	131..418	trimethoprim
i71	2018	*dfrA1_8*	100	474/474	36	2389..2862	trimethoprim
i71	2018	*erm(B)_1*	99.86	738/738	214	420..1157	macrolide
i71	2018	*mdf(A)_1*	98.46	1233/1233	30	33096..34323	macrolide
i71	2018	*mph(A)_1*	100	903/906	225	3290..4192	macrolide
i71	2018	*sul2_2*	100	816/816	4	51127..51942	sulphonamide
i71	2018	*tet(A)_4*	100	1200/1200	83	4236..5435	tetracycline
i72	2018	*aadA1_4*	100	789/789	38	3538..4326	aminoglycoside
i72	2018	*aph(3'')-Ib_5*	100	804/804	4	20230..21033	aminoglycoside
i72	2018	*aph(6)-Id_1*	100	837/837	4	19394..20230	aminoglycoside
i72	2018	*blaCTX-M-137_1*	92.69	876/876	291	708..1583	beta-lactam
i72	2018	*blaCTX-M-27_1*	100	876/876	291	708..1583	beta-lactam
i72	2018	*dfrA1_8*	100	474/474	38	2388..2861	trimethoprim
i72	2018	*erm(B)_1*	99.86	738/738	211	3538..4275	macrolide
i72	2018	*mdf(A)_1*	98.46	1233/1233	32	33096..34323	macrolide
i72	2018	*mph(A)_1*	100	903/906	225	3292..4194	macrolide

i72	2018	*sul2_2*	100	816/816	4	21094..21909	sulphonamide
i72	2018	*tet(A)_4*	100	1200/1200	81	13662..14861	tetracycline
i73	2018	*aadA1_4*	100	789/789	38	29373..30161	aminoglycoside
i73	2018	*aph(3'')-Ib_5*	100	788/804	162	7718..8505	aminoglycoside
i73	2018	*aph(6)-Id_1*	100	837/837	162	143..979	aminoglycoside
i73	2018	*blaCTX-M-15_1*	100	876/876	2	88106..88981	beta-lactam
i73	2018	*dfrA1_8*	100	474/474	38	30838..31311	trimethoprim
i73	2018	*mdf(A)_1*	98.46	1233/1233	31	33353..34580	macrolide
i73	2018	*qnrS1_1*	100	657/657	2	93622..94278	quinolone
i73	2018	*sul2_2*	100	816/816	162	6842..7657	sulphonamide
i73	2018	*tet(A)_6*	100	1275/1275	162	2240..3514	tetracycline
i74	2018	*aadA1_4*	100	789/789	37	29373..30161	aminoglycoside
i74	2018	*aph(3'')-Ib_5*	100	804/804	4	51584..52387	aminoglycoside
i74	2018	*aph(6)-Id_1*	100	837/837	4	52387..53223	aminoglycoside
i74	2018	*blaCTX-M-137_1*	92.69	876/876	279	708..1583	beta-lactam
i74	2018	*blaCTX-M-27_1*	100	876/876	279	708..1583	beta-lactam
i74	2018	*dfrA1_8*	100	474/474	37	30838..31311	trimethoprim
i74	2018	*erm(B)_1*	99.86	738/738	215	3538..4275	macrolide
i74	2018	*mdf(A)_1*	98.46	1233/1233	31	2966..4193	macrolide
i74	2018	*mph(A)_1*	100	903/906	226	3292..4194	macrolide
i74	2018	*sul2_2*	100	816/816	4	50708..51523	sulphonamide
i74	2018	*tet(A)_4*	100	1200/1200	82	4236..5435	tetracycline
i75	2018	*aadA1_4*	100	789/789	36	29373..30161	aminoglycoside
i75	2018	*aph(3'')-Ib_5*	100	804/804	45	20230..21033	aminoglycoside
i75	2018	*aph(6)-Id_1*	100	837/837	45	19394..20230	aminoglycoside
i75	2018	*blaCTX-M-137_1*	92.69	876/876	299	872..1747	beta-lactam
i75	2018	*blaCTX-M-27_1*	100	876/876	299	872..1747	beta-lactam

i75	2018	*dfrA1_8*	100	474/474	36	30838..31311	trimethoprim
i75	2018	*erm(B)_1*	99.86	738/738	221	420..1157	macrolide
i75	2018	*mdf(A)_1*	98.46	1233/1233	29	33096..34323	macrolide
i75	2018	*mph(A)_1*	100	903/906	236	3292..4194	macrolide
i75	2018	*sul2_2*	100	816/816	45	21094..21909	sulphonamide
i75	2018	*tet(A)_4*	100	1200/1200	85	4236..5435	tetracycline
i76	2018	*aadA1_4*	100	789/789	36	3539..4327	aminoglycoside
i76	2018	*aph(3'')-Ib_5*	100	804/804	4	52174..52977	aminoglycoside
i76	2018	*aph(6)-Id_1*	100	837/837	4	52977..53813	aminoglycoside
i76	2018	*blaCTX-M-137_1*	92.69	876/876	279	872..1747	beta-lactam
i76	2018	*blaCTX-M-27_1*	100	876/876	279	872..1747	beta-lactam
i76	2018	*dfrA1_8*	100	474/474	36	2389..2862	trimethoprim
i76	2018	*erm(B)_1*	99.86	738/738	213	3538..4275	macrolide
i76	2018	*mdf(A)_1*	98.46	1233/1233	30	33096..34323	macrolide
i76	2018	*mph(A)_1*	100	903/906	225	3292..4194	macrolide
i76	2018	*sul2_2*	100	816/816	4	51298..52113	sulphonamide
i76	2018	*tet(A)_4*	100	1200/1200	84	4236..5435	tetracycline
i77	2018	*aadA1_4*	100	789/789	38	29373..30161	aminoglycoside
i77	2018	*aph(3'')-Ib_5*	100	804/804	45	20230..21033	aminoglycoside
i77	2018	*aph(6)-Id_1*	100	837/837	45	19394..20230	aminoglycoside
i77	2018	*blaCTX-M-137_1*	92.69	876/876	303	708..1583	beta-lactam
i77	2018	*blaCTX-M-27_1*	100	876/876	303	708..1583	beta-lactam
i77	2018	*dfrA1_8*	100	474/474	38	30838..31311	trimethoprim
i77	2018	*erm(B)_1*	99.86	738/738	228	420..1157	macrolide
i77	2018	*mdf(A)_1*	98.46	1233/1233	46	24904..26131	macrolide
i77	2018	*mph(A)_1*	100	903/906	242	196..1098	macrolide
i77	2018	*sul2_2*	100	816/816	45	21094..21909	sulphonamide

i77	2018	*tet(A)_4*	100	1200/1200	86	13662..14861	tetracycline
i78	2018	*aadA1_4*	100	789/789	36	3539..4327	aminoglycoside
i78	2018	*aph(3'')-Ib_5*	100	804/804	44	20230..21033	aminoglycoside
i78	2018	*aph(6)-Id_1*	100	837/837	44	19394..20230	aminoglycoside
i78	2018	*blaCTX-M-137_1*	92.69	876/876	301	872..1747	beta-lactam
i78	2018	*blaCTX-M-27_1*	100	876/876	301	872..1747	beta-lactam
i78	2018	*dfrA1_8*	100	474/474	36	2389..2862	trimethoprim
i78	2018	*erm(B)_1*	99.86	738/738	225	3538..4275	macrolide
i78	2018	*mdf(A)_1*	98.46	1233/1233	30	33096..34323	macrolide
i78	2018	*mph(A)_1*	100	903/906	240	197..1099	macrolide
i78	2018	*sul2_2*	100	816/816	44	21094..21909	sulphonamide
i78	2018	*tet(A)_4*	100	1200/1200	83	14107..15306	tetracycline
i79	2018	*aadA1_4*	100	789/789	37	29373..30161	aminoglycoside
i79	2018	*aph(3'')-Ib_5*	100	804/804	44	20230..21033	aminoglycoside
i79	2018	*aph(6)-Id_1*	100	837/837	44	19394..20230	aminoglycoside
i79	2018	*blaCTX-M-137_1*	92.69	876/876	301	872..1747	beta-lactam
i79	2018	*blaCTX-M-27_1*	100	876/876	301	872..1747	beta-lactam
i79	2018	*dfrA1_8*	100	474/474	37	30838..31311	trimethoprim
i79	2018	*erm(B)_1*	99.86	738/738	224	3538..4275	macrolide
i79	2018	*mdf(A)_1*	98.46	1233/1233	31	33096..34323	macrolide
i79	2018	*mph(A)_1*	100	903/906	239	197..1099	macrolide
i79	2018	*sul2_2*	100	816/816	44	21094..21909	sulphonamide
i79	2018	*tet(A)_4*	100	1200/1200	85	13662..14861	tetracycline
i80	2018	*aadA1_4*	100	789/789	38	29373..30161	aminoglycoside
i80	2018	*aph(3'')-Ib_5*	100	804/804	4	20230..21033	aminoglycoside
i80	2018	*aph(6)-Id_1*	100	837/837	4	19394..20230	aminoglycoside
i80	2018	*blaCTX-M-137_1*	92.69	876/876	277	708..1583	beta-lactam

i80	2018	*blaCTX-M-27_1*	100	876/876	277	708..1583	beta-lactam
i80	2018	*dfrA1_8*	100	474/474	38	30838..31311	trimethoprim
i80	2018	*erm(B)_1*	99.86	738/738	212	420..1157	macrolide
i80	2018	*mdf(A)_1*	98.46	1233/1233	31	2966..4193	macrolide
i80	2018	*mph(A)_1*	100	903/906	224	196..1098	macrolide
i80	2018	*sul2_2*	100	816/816	4	21094..21909	sulphonamide
i80	2018	*tet(A)_4*	100	1200/1200	84	13662..14861	tetracycline
i81	2018	*aph(3'')-Ib_5*	99.88	804/804	158	5588..6391	aminoglycoside
i81	2018	*aph(6)-Id_1*	100	837/837	158	4752..5588	aminoglycoside
i81	2018	*blaCTX-M-15_1*	100	876/876	5	42624..43499	beta-lactam
i81	2018	*dfrA1_8*	99.79	474/474	39	29986..30459	trimethoprim
i81	2018	*mdf(A)_1*	98.46	1233/1233	131	2965..4192	macrolide
i81	2018	*qnrS1_1*	100	657/657	5	37327..37983	quinolone
i81	2018	*sul2_2*	100	816/816	158	6452..7267	sulphonamide
i81	2018	*tet(A)_6*	100	1275/1275	158	2217..3491	tetracycline
i82	2018	*aadA1_4*	100	789/789	38	3538..4326	aminoglycoside
i82	2018	*aph(3'')-Ib_5*	100	804/804	162	7629..8432	aminoglycoside
i82	2018	*aph(6)-Id_1*	100	837/837	162	31..867	aminoglycoside
i82	2018	*blaCTX-M-3_1*	100	876/876	1	18831..19706	beta-lactam
i82	2018	*dfrA1_8*	100	474/474	38	2388..2861	trimethoprim
i82	2018	*mdf(A)_1*	98.46	1233/1233	30	2966..4193	macrolide
i82	2018	*sul2_2*	100	816/816	162	6753..7568	sulphonamide
i82	2018	*tet(A)_6*	100	1275/1275	162	2128..3402	tetracycline
i83	2018	*aadA1_4*	100	789/789	37	3898..4686	aminoglycoside
i83	2018	*aph(3'')-Ib_5*	100	804/804	165	4187..4990	aminoglycoside
i83	2018	*aph(6)-Id_1*	100	837/837	165	3351..4187	aminoglycoside
i83	2018	*blaCTX-M-15_1*	100	876/876	107	262..1137	beta-lactam

i83	2018	*dfrA1_8*	100	474/474	37	2748..3221	trimethoprim
i83	2018	*mdf(A)_1*	98.46	1233/1233	30	2966..4193	macrolide
i83	2018	*qnrS1_1*	100	657/657	107	5778..6434	quinolone
i83	2018	*sul2_2*	100	816/816	165	5051..5866	sulphonamide
i83	2018	*tet(A)_6*	100	1275/1275	165	816..2090	tetracycline
i84	2018	*aadA1_3*	99.75	792/792	236	207..998	aminoglycoside
i84	2018	*aadA1_5*	99.75	792/792	236	207..998	aminoglycoside
i84	2018	*aadA5_1*	100	789/789	134	979..1767	aminoglycoside
i84	2018	*blaCTX-M-15_1*	100	876/876	202	264..1139	beta-lactam
i84	2018	*blaOXA-1_1*	100	831/831	236	1111..1941	beta-lactam
i84	2018	*catA1_1*	99.85	660/660	165	5363..6022	phenicol
i84	2018	*dfrA17_1*	100	474/474	134	375..848	trimethoprim
i84	2018	*mdf(A)_1*	98.22	1233/1233	37	2157..3383	macrolide
i84	2018	*mph(A)_1*	100	906/906	134	8464..9369	macrolide
i84	2018	*qnrS1_1*	100	657/657	102	1547..2203	quinolone
i84	2018	*sul1_2*	100	840/840	134	2314..3153	sulphonamide
i84	2018	*tet(B)_2*	100	1206/1206	189	1176..2381	tetracycline
i85	2018	*aadA1_4*	100	789/789	37	29373..30161	aminoglycoside
i85	2018	*aph(3'')-Ib_5*	100	720/804	165	1..720	aminoglycoside
i85	2018	*aph(6)-Id_1*	100	837/837	165	7471..8307	aminoglycoside
i85	2018	*blaCTX-M-15_1*	100	876/876	268	347..1222	beta-lactam
i85	2018	*dfrA1_8*	100	474/474	37	30838..31311	trimethoprim
i85	2018	*mdf(A)_1*	98.46	1233/1233	44	2966..4193	macrolide
i85	2018	*qnrS1_1*	100	657/657	112	1547..2203	quinolone
i85	2018	*sul2_2*	100	816/816	165	781..1596	sulphonamide
i85	2018	*tet(A)_6*	100	1275/1275	165	4936..6210	tetracycline
i86	2018	*aadA1_4*	100	789/789	37	29373..30161	aminoglycoside

i86	2018	*aph(3'')-Ib_5*	100	804/804	161	5547..6350	aminoglycoside
i86	2018	*aph(6)-Id_1*	100	837/837	161	6350..7186	aminoglycoside
i86	2018	*blaCTX-M-15_1*	100	876/876	3	87963..88838	beta-lactam
i86	2018	*dfrA1_8*	100	474/474	37	30838..31311	trimethoprim
i86	2018	*mdf(A)_1*	98.46	1233/1233	30	33353..34580	macrolide
i86	2018	*qnrS1_1*	100	657/657	3	93479..94135	quinolone
i86	2018	*sul2_2*	100	816/816	161	4671..5486	sulphonamide
i86	2018	*tet(A)_6*	100	1275/1275	161	68..1342	tetracycline
i87	2018	*aadA1_4*	100	789/789	37	29373..30161	aminoglycoside
i87	2018	*aph(3'')-Ib_5*	100	804/804	164	1769..2572	aminoglycoside
i87	2018	*aph(6)-Id_1*	100	837/837	164	2572..3408	aminoglycoside
i87	2018	*blaCTX-M-15_1*	100	876/876	238	2826..3701	beta-lactam
i87	2018	*dfrA1_8*	100	474/474	37	30838..31311	trimethoprim
i87	2018	*mdf(A)_1*	98.46	1233/1233	31	33219..34446	macrolide
i87	2018	*qnrS1_1*	100	657/657	143	8008..8664	quinolone
i87	2018	*sul2_2*	100	816/816	164	893..1708	sulphonamide
i87	2018	*tet(A)_6*	100	1275/1275	164	4669..5943	tetracycline
i88	2018	*aac(3)-IId_1*	99.88	861/861	1	12906..13766	aminoglycoside
i88	2018	*aadA1_4*	100	789/789	40	3538..4326	aminoglycoside
i88	2018	*blaCTX-M-15_1*	100	876/876	1	10846..11721	beta-lactam
i88	2018	*blaTEM-1B_1*	100	861/861	1	18738..19598	beta-lactam
i88	2018	*dfrA1_8*	100	474/474	40	2388..2861	trimethoprim
i88	2018	*mdf(A)_1*	98.46	1233/1233	32	2966..4193	macrolide
i89	2018	*aadA1_3*	99.75	792/792	331	1370..2161	aminoglycoside
i89	2018	*aadA1_5*	99.75	792/792	331	1370..2161	aminoglycoside
i89	2018	*aadA5_1*	100	789/789	149	979..1767	aminoglycoside
i89	2018	*aph(3'')-Ib_5*	100	804/804	211	1196..1999	aminoglycoside

i89	2018	*aph(6)-Id_1*	99.88	837/837	211	1999..2835	aminoglycoside
i89	2018	*blaCTX-M-15_1*	100	876/876	278	264..1139	beta-lactam
i89	2018	*blaOXA-1_1*	100	831/831	331	427..1257	beta-lactam
i89	2018	*catA1_1*	99.85	660/660	210	5363..6022	phenicol
i89	2018	*dfrA17_1*	100	474/474	149	375..848	trimethoprim
i89	2018	*dfrA1_8*	100	474/474	27	29991..30464	trimethoprim
i89	2018	*mdf(A)_1*	98.38	1233/1233	89	9066..10295	macrolide
i89	2018	*mph(A)_1*	100	906/906	149	8464..9369	macrolide
i89	2018	*qnrS1_1*	100	657/657	208	4241..4897	quinolone
i89	2018	*sul1_2*	100	840/840	149	2314..3153	sulphonamide
i89	2018	*sul2_2*	100	816/816	211	320..1135	sulphonamide
i89	2018	*tet(B)_2*	100	1206/1206	225	2508..3713	tetracycline
i90	2018	*aadA5_1*	100	789/789	150	1520..2308	aminoglycoside
i90	2018	*aph(6)-Id_1*	100	837/837	168	453..1289	aminoglycoside
i90	2018	*blaCTX-M-15_1*	100	876/876	251	2826..3701	beta-lactam
i90	2018	*dfrA17_1*	100	474/474	150	916..1389	trimethoprim
i90	2018	*dfrA1_8*	100	474/474	188	4590..5063	trimethoprim
i90	2018	*mdf(A)_1*	98.46	1233/1233	141	2965..4192	macrolide
i90	2018	*mph(A)_1*	100	906/906	150	9005..9910	macrolide
i90	2018	*qnrS1_1*	100	657/657	135	9076..9732	quinolone
i90	2018	*sul1_2*	100	840/840	150	2855..3694	sulphonamide
i90	2018	*sul2_2*	100	816/816	168	7175..7990	sulphonamide
i90	2018	*tet(A)_6*	100	1275/1275	168	2550..3824	tetracycline
i91	2018	*aadA1_4*	100	789/789	36	3538..4326	aminoglycoside
i91	2018	*aph(3'')-Ib_5*	100	804/804	161	1921..2724	aminoglycoside
i91	2018	*aph(6)-Id_1*	100	837/837	161	2724..3560	aminoglycoside
i91	2018	*blaCTX-M-3_1*	100	876/876	1	71302..72177	beta-lactam

i91	2018	*dfrA1_8*	100	474/474	36	2388..2861	trimethoprim
i91	2018	*mdf(A)_1*	98.46	1233/1233	29	33353..34580	macrolide
i91	2018	*sul2_2*	100	816/816	161	1045..1860	sulphonamide
i91	2018	*tet(A)_6*	100	1275/1275	161	4821..6095	tetracycline
i92	2018	*aadA1_4*	100	789/789	37	3538..4326	aminoglycoside
i92	2018	*aph(3'')-Ib_5*	100	804/804	161	5461..6264	aminoglycoside
i92	2018	*aph(6)-Id_1*	100	837/837	161	4625..5461	aminoglycoside
i92	2018	*blaCTX-M-3_1*	100	876/876	2	20022..20897	beta-lactam
i92	2018	*dfrA1_8*	100	474/474	37	2388..2861	trimethoprim
i92	2018	*mdf(A)_1*	98.46	1233/1233	30	2966..4193	macrolide
i92	2018	*sul2_2*	100	816/816	161	6325..7140	sulphonamide
i92	2018	*tet(A)_6*	100	1275/1275	161	2090..3364	tetracycline
i93	2018	*aadA1_4*	100	789/789	37	3538..4326	aminoglycoside
i93	2018	*aph(3'')-Ib_5*	100	804/804	159	3369..4172	aminoglycoside
i93	2018	*aph(6)-Id_1*	100	837/837	159	4172..5008	aminoglycoside
i93	2018	*blaCTX-M-15_1*	100	876/876	10	34773..35648	beta-lactam
i93	2018	*dfrA1_8*	100	474/474	37	2388..2861	trimethoprim
i93	2018	*mdf(A)_1*	98.46	1233/1233	31	33219..34446	macrolide
i93	2018	*sul2_2*	100	816/816	159	2493..3308	sulphonamide
i93	2018	*tet(A)_6*	100	1275/1275	159	6269..7543	tetracycline
i94	2018	*aadA1_4*	100	789/789	36	29373..30161	aminoglycoside
i94	2018	*aph(3'')-Ib_5*	100	787/804	163	1..787	aminoglycoside
i94	2018	*aph(6)-Id_1*	100	837/837	163	787..1623	aminoglycoside
i94	2018	*blaCTX-M-15_1*	100	876/876	260	347..1222	beta-lactam
i94	2018	*dfrA1_8*	100	474/474	36	30838..31311	trimethoprim
i94	2018	*mdf(A)_1*	98.46	1233/1233	29	2966..4193	macrolide
i94	2018	*qnrS1_1*	100	657/657	113	11822..12478	quinolone

i94	2018	*sul2_2*	100	816/816	163	7498..8313	sulphonamide
i94	2018	*tet(A)_6*	100	1275/1275	163	2884..4158	tetracycline
i95	2018	*aph(3'')-Ib_5*	100	804/804	92	4623..5426	aminoglycoside
i95	2018	*aph(6)-Id_1*	100	837/837	92	5426..6262	aminoglycoside
i95	2018	*blaDHA-1_1*	100	1140/1140	98	5248..6387	beta-lactam
i95	2018	*blaTEM-1B_1*	100	861/861	92	10759..11619	beta-lactam
i95	2018	*dfrA14_1*	100	474/474	342	144..617	trimethoprim
i95	2018	*dfrA17_1*	100	474/474	282	588..1061	trimethoprim
i95	2018	*mdf(A)_1*	98.22	1233/1233	34	2157..3383	macrolide
i95	2018	*mph(A)_1*	100	906/906	98	14114..15019	macrolide
i95	2018	*qnrB4_1*	100	645/645	98	480..1124	quinolone
i95	2018	*sul1_2*	100	840/840	98	7964..8803	sulphonamide
i95	2018	*sul2_2*	100	816/816	92	3747..4562	sulphonamide
i95	2018	*tet(A)_3*	99.75	1200/1200	92	8252..9451	tetracycline
i96	2018	*aadA1_4*	100	789/789	35	3538..4326	aminoglycoside
i96	2018	*blaCTX-M-3_1*	100	876/876	2	66443..67318	beta-lactam
i96	2018	*dfrA1_8*	100	474/474	35	2388..2861	trimethoprim
i96	2018	*mdf(A)_1*	98.46	1233/1233	28	2966..4193	macrolide
i97	2018	*aadA1_4*	100	789/789	34	3538..4326	aminoglycoside
i97	2018	*blaCTX-M-3_1*	100	876/876	57	18939..19814	beta-lactam
i97	2018	*dfrA1_8*	100	474/474	34	2388..2861	trimethoprim
i97	2018	*mdf(A)_1*	98.46	1233/1233	28	33353..34580	macrolide
i98	2018	*aadA1_4*	100	789/789	37	29373..30161	aminoglycoside
i98	2018	*aph(3'')-Ib_5*	100	804/804	4	20230..21033	aminoglycoside
i98	2018	*aph(6)-Id_1*	100	837/837	4	19394..20230	aminoglycoside
i98	2018	*blaCTX-M-137_1*	92.69	876/876	279	708..1583	beta-lactam
i98	2018	*blaCTX-M-27_1*	100	876/876	279	708..1583	beta-lactam

i98	2018	*dfrA1_8*	100	474/474	37	30838..31311	trimethoprim
i98	2018	*erm(B)_1*	99.86	738/738	215	420..1157	macrolide
i98	2018	*mdf(A)_1*	98.46	1233/1233	31	2966..4193	macrolide
i98	2018	*mph(A)_1*	100	903/906	226	3292..4194	macrolide
i98	2018	*sul2_2*	100	816/816	4	21094..21909	sulphonamide
i98	2018	*tet(A)_4*	100	1200/1200	83	13662..14861	tetracycline
i99	2018	*aph(3'')-Ib_2*	99.83	570/804	162	7959..8528	aminoglycoside
i99	2018	*aph(3'')-Ib_3*	99.83	570/804	162	7959..8528	aminoglycoside
i99	2018	*aph(3'')-Ib_5*	99.83	570/804	162	7959..8528	aminoglycoside
i99	2018	*aph(6)-Id_1*	100	837/837	162	7123..7959	aminoglycoside
i99	2018	*blaCTX-M-15_1*	99.89	876/876	141	264..1139	beta-lactam
i99	2018	*blaTEM-1B_1*	100	861/861	21	5267..6127	beta-lactam
i99	2018	*dfrA1_8*	99.79	474/474	39	2387..2860	trimethoprim
i99	2018	*erm(B)_1*	99.86	738/738	228	420..1157	macrolide
i99	2018	*mdf(A)_1*	98.46	1233/1233	134	6783..8010	macrolide
i99	2018	*mph(A)_1*	100	903/906	21	235..1137	macrolide
i99	2018	*qnrS1_1*	100	657/657	141	5780..6436	quinolone
i99	2018	*sul2_2*	100	816/816	162	422..1237	sulphonamide
i99	2018	*tet(A)_6*	100	1275/1275	162	4588..5862	tetracycline
i100	2018	*aadA1_4*	100	789/789	137	3536..4324	aminoglycoside
i100	2018	*aph(3'')-Ib_5*	100	804/804	64	4623..5426	aminoglycoside
i100	2018	*aph(6)-Id_1*	100	837/837	64	5426..6262	aminoglycoside
i100	2018	*blaCTX-M-15_1*	100	876/876	64	15733..16608	beta-lactam
i100	2018	*blaTEM-1B_1*	100	861/861	64	19430..20290	beta-lactam
i100	2018	*dfrA14_1*	100	474/474	286	311..784	trimethoprim
i100	2018	*dfrA1_8*	100	474/474	137	2386..2859	trimethoprim
i100	2018	*mdf(A)_1*	98.54	1233/1233	157	3478..4708	macrolide

i100	2018	*qnrS1_1*	100	657/657	64	10436..11092	quinolone
i100	2018	*sul2_2*	100	816/816	64	3747..4562	sulphonamide
i100	2018	*tet(A)_4*	100	1200/1200	7	2768..3967	tetracycline

Table S4: Antimicrobial resistance genes detected with ResFinder in all short-read assemblies of the *Salmonella* isolates used in this study

**Isolate**	**Year**	**Resistance gene**	**Identity (%)**	**Query/Template Length**	**Contig**	**Position in contig**	**Antibiotic class**
i101	2016	*aac(3)-IId_1*	99.88	861/861	44	366..1226	aminoglycoside
i101	2016	*aac(6')-Iaa_1*	100	438/438	1	145460..145897	aminoglycoside
i101	2016	*aadA1_3*	100	792/792	27	4620..5411	aminoglycoside
i101	2016	*aph(3')-Ia_3*	99.88	816/816	51	1203..2018	aminoglycoside
i101	2016	*aph(6)-Id_4*	99.86	741/831	51	127..867	aminoglycoside
i101	2016	*blaCTX-M-55_1*	100	876/876	36	6059..6934	beta-lactam
i101	2016	*cmlA1_1*	99.92	1260/1260	27	5504..6763	phenicol
i101	2016	*dfrA12_8*	99.8	498/498	27	8224..8721	trimethoprim
i101	2016	*qnrS1_1*	100	657/657	36	762..1418	quinolone
i101	2016	*sul3_2*	100	792/792	27	2147..2938	sulphonamide
i101	2016	*tet(A)_4*	100	1200/1200	27	12556..13755	tetracycline
i101	2016	*tet(B)_2*	100	1206/1206	33	4910..6115	tetracycline
i102	2016	*aac(3)-IId_1*	99.88	861/861	50	772..1632	aminoglycoside
i102	2016	*aac(6')-Iaa_1*	97.03	438/438	3	110698..111135	aminoglycoside
i102	2016	*aph(3'')-Ib_5*	100	804/804	30	24052..24855	aminoglycoside
i102	2016	*aph(6)-Id_1*	100	837/837	30	24855..25691	aminoglycoside
i102	2016	*blaCTX-M-55_1*	100	876/876	51	322..1197	beta-lactam
i102	2016	*blaTEM-1B_1*	100	861/861	52	651..1511	beta-lactam
i102	2016	*catA2_1*	96.11	642/642	61	155..796	phenicol
i102	2016	*oqxA_1*	100	1176/1176	37	383..1558	quinolone

i102	2016	*oqxB_1*	99.97	3153/3153	37	1582..4734	quinolone
i102	2016	*qnrS1_1*	100	657/657	44	761..1417	quinolone
i102	2016	*sul2_2*	100	816/816	30	23176..23991	sulphonamide
i102	2016	*tet(A)_6*	100	1247/1275	47	981..2227	tetracycline
i103	2016	*aac(6')-Iaa_1*	97.49	438/438	1	574093..574530	aminoglycoside
i103	2016	*aph(3')-Ia_7*	100	816/816	45	220..1035	aminoglycoside
i103	2016	*blaCTX-M-1_1*	100	876/876	36	3584..4459	beta-lactam
i103	2016	*dfrA14_4*	100	474/474	35	2371..2844	trimethoprim
i103	2016	*dfrA1_10*	99.79	474/474	15	99516..99989	trimethoprim
i103	2016	*dfrA1_8*	99.79	474/474	15	99516..99989	trimethoprim
i103	2016	*dfrA1_9*	99.79	474/474	15	99516..99989	trimethoprim
i103	2016	*sul1_2*	100	840/840	15	98144..98983	sulphonamide
i103	2016	*tet(A)_4*	100	1200/1200	15	87303..88502	tetracycline
i104	2016	*aac(6')-Iaa_1*	100	438/438	1	569447..569884	aminoglycoside
i104	2016	*aadA1_3*	100	792/792	19	63533..64324	aminoglycoside
i104	2016	*blaCTX-M-1_1*	100	876/876	27	25139..26014	beta-lactam
i104	2016	*blaTEM-1B_1*	100	861/861	19	54969..55829	beta-lactam
i104	2016	*cmlA1_1*	99.84	1260/1260	19	62181..63440	phenicol
i104	2016	*dfrA12_8*	100	498/498	19	60223..60720	trimethoprim
i104	2016	*mef(B)_1*	100	1230/1230	19	68558..69787	macrolide
i104	2016	*sul3_2*	100	792/792	19	66006..66797	sulphonamide
i105	2016	*aac(6')-Iaa_1*	96.35	438/438	1	815223..815660	aminoglycoside
i105	2016	*blaCTX-M-15_1*	100	876/876	17	521..1396	beta-lactam
i106	2016	*blaCTX-M-15_1*	100	876/876	36	1507..2382	beta-lactam
i106	2016	*blaOXA-1_1*	100	602/831	36	514..1115	beta-lactam
i106	2016	*blaTEM-1B_1*	100	861/861	11	178502..179362	beta-lactam
i106	2016	*mdf(A)_1*	98.38	1233/1233	19	32587..33819	macrolide
i106	2016	*mph(A)_1*	100	906/906	48	2048..2953	macrolide

i107	2016	*aac(6')-Iaa_1*	97.49	438/438	1	574093..574530	aminoglycoside
i107	2016	*aph(3')-Ia_7*	100	816/816	36	214..1029	aminoglycoside
i107	2016	*blaCTX-M-1_1*	100	876/876	30	3584..4459	beta-lactam
i107	2016	*dfrA14_4*	100	474/474	28	3249..3722	trimethoprim
i107	2016	*dfrA1_10*	99.79	474/474	15	99516..99989	trimethoprim
i107	2016	*dfrA1_8*	99.79	474/474	15	99516..99989	trimethoprim
i107	2016	*dfrA1_9*	99.79	474/474	15	99516..99989	trimethoprim
i107	2016	*sul1_2*	100	840/840	15	98144..98983	sulphonamide
i107	2016	*tet(A)_4*	100	1200/1200	15	87303..88502	tetracycline
i108	2016	*aac(6')-Iaa_1*	100	438/438	1	569447..569884	aminoglycoside
i108	2016	*aph(3'')-Ib_5*	100	804/804	37	2865..3668	aminoglycoside
i108	2016	*aph(6)-Id_1*	100	837/837	37	3668..4504	aminoglycoside
i108	2016	*blaCTX-M-55_1*	100	876/876	39	671..1546	beta-lactam
i108	2016	*blaTEM-1B_1*	100	861/861	46	660..1520	beta-lactam
i108	2016	*qnrS1_1*	100	657/657	42	1547..2203	quinolone
i108	2016	*sul2_3*	100	816/816	37	1989..2804	sulphonamide
i108	2016	*tet(B)_2*	100	1206/1206	30	10268..11473	tetracycline
i109	2016	*aac(6')-Iaa_1*	100	438/438	1	569447..569884	aminoglycoside
i109	2016	*aph(3'')-Ib_5*	100	804/804	37	972..1775	aminoglycoside
i109	2016	*aph(6)-Id_1*	100	837/837	37	136..972	aminoglycoside
i109	2016	*blaCTX-M-55_1*	100	876/876	39	671..1546	beta-lactam
i109	2016	*blaTEM-1B_1*	100	861/861	46	660..1520	beta-lactam
i109	2016	*qnrS1_1*	100	657/657	41	761..1417	quinolone
i109	2016	*sul2_3*	100	816/816	37	1836..2651	sulphonamide
i109	2016	*tet(B)_2*	100	1206/1206	31	10268..11473	tetracycline
i110	2016	*aac(6')-Iaa_1*	100	438/438	1	569447..569884	aminoglycoside
i110	2016	*aph(3'')-Ib_5*	100	804/804	33	972..1775	aminoglycoside
i110	2016	*aph(6)-Id_1*	100	837/837	33	136..972	aminoglycoside

i110	2016	*blaCTX-M-55_1*	100	876/876	35	671..1546	beta-lactam
i110	2016	*blaTEM-1B_1*	100	861/861	42	660..1520	beta-lactam
i110	2016	*qnrS1_1*	100	657/657	39	761..1417	quinolone
i110	2016	*sul2_3*	100	816/816	33	1836..2651	sulphonamide
i110	2016	*tet(B)_2*	100	1206/1206	27	10268..11473	tetracycline
i111	2016	*aac(6')-Iaa_1*	100	438/438	2	337959..338396	aminoglycoside
i111	2016	*aadA2b_1*	99.87	780/780	45	362..1141	aminoglycoside
i111	2016	*ant(2'')-Ia_1*	100	534/534	45	1211..1744	aminoglycoside
i111	2016	*blaCTX-M-137_1*	92.12	876/876	34	221..1096	beta-lactam
i111	2016	*blaCTX-M-9_1*	100	876/876	34	221..1096	beta-lactam
i111	2016	*catA1_1*	99.55	660/660	49	241..900	phenicol
i111	2016	*dfrA16_2*	100	474/474	55	222..695	trimethoprim
i111	2016	*qnrA1_1*	99.85	657/657	40	158..814	quinolone
i111	2016	*sul1_2*	100	840/840	50	271..1110	sulphonamide
i111	2016	*tet(A)_4*	94.92	1200/1200	37	1655..2854	tetracycline
i112	2016	*aac(6')-Iaa_1*	100	438/438	1	569630..570067	aminoglycoside
i112	2016	*aadA2b_1*	99.87	780/780	35	362..1141	aminoglycoside
i112	2016	*ant(2'')-Ia_1*	100	534/534	35	1211..1744	aminoglycoside
i112	2016	*blaCTX-M-137_1*	92.12	876/876	38	221..1096	beta-lactam
i112	2016	*blaCTX-M-9_1*	100	876/876	38	221..1096	beta-lactam
i112	2016	*blaTEM-1B_1*	100	861/861	12	73077..73937	beta-lactam
i112	2016	*catA1_1*	99.55	660/660	50	296..955	phenicol
i112	2016	*qnrA1_1*	99.85	657/657	43	158..814	quinolone
i112	2016	*sul1_2*	100	840/840	51	271..1110	sulphonamide
i112	2016	*tet(A)_4*	94.92	1200/1200	41	1180..2379	tetracycline
i113	2016	*aac(6')-Iaa_1*	100	438/438	3	309171..309608	aminoglycoside
i113	2016	*aadA2b_1*	99.87	780/780	58	145..924	aminoglycoside
i113	2016	*aadA5_1*	100	789/789	19	82142..82930	aminoglycoside

i113	2016	*blaCARB-2_1*	100	867/867	55	189..1055	beta-lactam
i113	2016	*blaCTX-M-1_1*	100	876/876	19	894..1769	beta-lactam
i113	2016	*dfrA17_1*	100	474/474	19	83061..83534	trimethoprim
i113	2016	*floR_2*	100	1215/1215	38	787..2001	phenicol
i113	2016	*sul1_39*	100	529/828	61	455..983	sulphonamide
i113	2016	*sul2_2*	100	816/816	19	78925..79740	sulphonamide
i113	2016	*tet(G)_2*	100	1176/1176	38	2938..4113	tetracycline
i114	2016	*aac(6')-Iaa_1*	100	438/438	1	118179..118616	aminoglycoside
i114	2016	*aph(3'')-Ib_3*	99.88	804/804	66	33..836	aminoglycoside
i114	2016	*aph(3'')-Ib_4*	99.88	804/804	66	33..836	aminoglycoside
i114	2016	*aph(3'')-Ib_5*	99.88	804/804	66	33..836	aminoglycoside
i114	2016	*aph(3')-Ia_3*	99.88	816/816	45	455..1270	aminoglycoside
i114	2016	*aph(6)-Id_1*	100	837/837	66	836..1672	aminoglycoside
i114	2016	*blaCTX-M-32_2*	100	876/876	64	207..1082	beta-lactam
i114	2016	*blaTEM-1B_1*	100	861/861	59	660..1520	beta-lactam
i114	2016	*sul2_2*	100	791/816	78	11..801	sulphonamide
i114	2016	*tet(B)_1*	99.92	1206/1206	43	4431..5636	tetracycline
i115	2017	*ARR-2_1*	100	453/453	32	957..1409	rifampicin
i115	2017	*aac(3)-IId_1*	99.88	861/861	29	392..1252	aminoglycoside
i115	2017	*aac(6')-Iaa_1*	99.31	438/438	1	825440..825877	aminoglycoside
i115	2017	*aadA22_1*	100	792/792	31	1072..1863	aminoglycoside
i115	2017	*aph(3')-Ia_3*	99.88	816/816	35	369..1184	aminoglycoside
i115	2017	*aph(6)-Id_1*	100	837/837	25	5048..5884	aminoglycoside
i115	2017	*blaCTX-M-55_1*	100	876/876	22	3357..4232	beta-lactam
i115	2017	*blaTEM-1B_1*	100	861/861	22	7054..7914	beta-lactam
i115	2017	*dfrA14_1*	100	474/474	32	373..846	trimethoprim
i115	2017	*floR_2*	98.11	1214/1215	24	756..1969	phenicol
i115	2017	*lnu(F)_1*	100	822/822	31	1995..2816	macrolide

i115	2017	*mph(A)_1*	100	906/906	30	2704..3609	macrolide
i115	2017	*qnrS1_1*	100	657/657	27	3727..4383	quinolone
i115	2017	*sul3_2*	100	792/792	21	13516..14307	sulphonamide
i115	2017	*tet(A)_4*	100	1200/1200	25	2513..3712	tetracycline
i116	2017	*ARR-2_1*	100	453/453	32	957..1409	rifampicin
i116	2017	*aac(3)-IId_1*	99.88	861/861	29	392..1252	aminoglycoside
i116	2017	*aac(6')-Iaa_1*	99.31	438/438	1	825347..825784	aminoglycoside
i116	2017	*aadA22_1*	100	792/792	31	1168..1959	aminoglycoside
i116	2017	*aph(3')-Ia_3*	99.88	816/816	37	250..1065	aminoglycoside
i116	2017	*aph(6)-Id_1*	100	837/837	24	276..1112	aminoglycoside
i116	2017	*blaCTX-M-55_1*	100	876/876	28	174..1049	beta-lactam
i116	2017	*blaTEM-1B_1*	100	861/861	25	3374..4234	beta-lactam
i116	2017	*dfrA14_1*	100	474/474	32	373..846	trimethoprim
i116	2017	*floR_2*	98.11	1214/1215	23	4501..5714	phenicol
i116	2017	*lnu(F)_1*	100	822/822	31	215..1036	macrolide
i116	2017	*mph(A)_1*	100	906/906	30	196..1101	macrolide
i116	2017	*qnrS1_1*	100	657/657	26	3727..4383	quinolone
i116	2017	*sul3_2*	100	792/792	21	13516..14307	sulphonamide
i116	2017	*tet(A)_4*	100	1200/1200	24	2448..3647	tetracycline
i117	2017	*aac(6')-Iaa_1*	97.47	435/438	11	130850..131284	aminoglycoside
i117	2017	*aph(3'')-Ib_5*	100	804/804	55	845..1648	aminoglycoside
i117	2017	*aph(6)-Id_1*	100	837/837	55	9..845	aminoglycoside
i117	2017	*blaCTX-M-15_1*	100	876/876	40	2513..3388	beta-lactam
i117	2017	*blaTEM-1B_1*	100	861/861	61	149..1009	beta-lactam
i117	2017	*catA1_1*	99.85	660/660	43	8216..8875	phenicol
i117	2017	*dfrA7_1*	100	474/474	43	1719..2192	trimethoprim
i117	2017	*qnrS1_1*	100	657/657	40	8029..8685	quinolone
i117	2017	*sul1_2*	100	840/840	43	309..1148	sulphonamide

i117	2017	*sul2_2*	100	816/816	55	1709..2524	sulphonamide
i118	2017	*aac(6')-Iaa_1*	100	438/438	2	263032..263469	aminoglycoside
i118	2017	*aadA2b_1*	99.87	780/780	42	737..1516	aminoglycoside
i118	2017	*ant(2'')-Ia_1*	100	534/534	42	134..667	aminoglycoside
i118	2017	*blaCTX-M-137_1*	92.12	876/876	32	221..1096	beta-lactam
i118	2017	*blaCTX-M-9_1*	100	876/876	32	221..1096	beta-lactam
i118	2017	*catA1_1*	99.55	660/660	47	241..900	phenicol
i118	2017	*dfrA16_2*	100	474/474	53	144..617	trimethoprim
i118	2017	*qnrA1_1*	99.85	657/657	37	158..814	quinolone
i118	2017	*sul1_2*	100	840/840	48	271..1110	sulphonamide
i118	2017	*tet(A)_4*	94.92	1200/1200	35	1180..2379	tetracycline
i119	2017	*aac(6')-Iaa_1*	100	438/438	7	31671..32108	aminoglycoside
i119	2017	*aph(3'')-Ib_5*	100	804/804	34	7838..8641	aminoglycoside
i119	2017	*aph(6)-Id_1*	100	837/837	34	8641..9477	aminoglycoside
i119	2017	*blaCTX-M-137_1*	92.58	876/876	22	70303..71178	beta-lactam
i119	2017	*blaCTX-M-14_1*	100	876/876	22	70303..71178	beta-lactam
i119	2017	*sul2_2*	100	816/816	34	6962..7777	sulphonamide
i119	2017	*tet(A)_4*	100	1023/1200	34	1..1023	tetracycline
i120	2017	*aac(6')-Iaa_1*	100	438/438	3	309171..309608	aminoglycoside
i120	2017	*aadA2b_1*	99.87	780/780	51	145..924	aminoglycoside
i120	2017	*blaCARB-2_1*	100	867/867	35	231..1097	beta-lactam
i120	2017	*blaCTX-M-3_1*	100	876/876	17	66258..67133	beta-lactam
i120	2017	*floR_2*	100	1215/1215	35	6731..7945	phenicol
i120	2017	*sul1_39*	100	529/828	55	455..983	sulphonamide
i120	2017	*tet(G)_2*	100	1176/1176	35	4619..5794	tetracycline
i121	2017	*aac(6')-Iaa_1*	100	438/438	1	255224..255661	aminoglycoside
i121	2017	*aadA2b_1*	99.87	780/780	32	12466..13245	aminoglycoside
i121	2017	*ant(2'')-Ia_1*	100	534/534	32	11863..12396	aminoglycoside

i121	2017	*blaCTX-M-137_1*	92.12	876/876	34	3359..4234	beta-lactam
i121	2017	*blaCTX-M-9_1*	100	876/876	34	3359..4234	beta-lactam
i121	2017	*blaTEM-1B_1*	100	861/861	13	67794..68654	beta-lactam
i121	2017	*catA1_1*	99.55	660/660	46	241..900	phenicol
i121	2017	*qnrA1_1*	99.85	657/657	39	1509..2165	quinolone
i121	2017	*sul1_2*	100	840/840	47	271..1110	sulphonamide
i121	2017	*tet(A)_4*	94.92	1200/1200	37	1180..2379	tetracycline
i122	2017	*aac(6')-Iaa_1*	97.72	438/438	1	635026..635463	aminoglycoside
i122	2017	*aadA2_2*	100	792/792	20	6996..7787	aminoglycoside
i122	2017	*aph(3'')-Ib_5*	100	804/804	14	48068..48871	aminoglycoside
i122	2017	*aph(6)-Id_1*	100	837/837	14	48871..49707	aminoglycoside
i122	2017	*blaCMY-2_1*	100	1146/1146	14	17251..18396	beta-lactam
i122	2017	*blaTEM-1B_1*	100	861/861	29	444..1304	beta-lactam
i122	2017	*dfrA12_8*	100	498/498	20	6091..6588	trimethoprim
i122	2017	*floR_2*	98.19	1214/1215	14	52843..54056	phenicol
i122	2017	*mph(A)_1*	100	906/906	20	196..1101	macrolide
i122	2017	*qnrS1_1*	100	657/657	22	7507..8163	quinolone
i122	2017	*sul1_2*	100	840/840	20	8292..9131	sulphonamide
i122	2017	*sul2_2*	100	816/816	14	47192..48007	sulphonamide
i122	2017	*tet(A)_6*	99.92	1275/1275	14	50968..52242	tetracycline
i123	2017	*aac(6')-Iaa_1*	100	438/438	1	380063..380500	aminoglycoside
i123	2017	*aadA2b_1*	99.87	780/780	61	737..1516	aminoglycoside
i123	2017	*ant(2'')-Ia_1*	100	534/534	61	134..667	aminoglycoside
i123	2017	*aph(3'')-Ib_5*	100	804/804	63	845..1648	aminoglycoside
i123	2017	*aph(6)-Id_1*	100	837/837	63	9..845	aminoglycoside
i123	2017	*blaCTX-M-137_1*	92.12	876/876	45	221..1096	beta-lactam
i123	2017	*blaCTX-M-9_1*	100	876/876	45	221..1096	beta-lactam
i123	2017	*blaTEM-1B_1*	100	861/861	65	522..1382	beta-lactam

i123	2017	*dfrA16_2*	100	474/474	74	222..695	trimethoprim
i123	2017	*floR_2*	98.19	1214/1215	44	1502..2715	phenicol
i123	2017	*qnrA1_1*	99.85	657/657	56	158..814	quinolone
i123	2017	*sul1_2*	100	840/840	70	25..864	sulphonamide
i123	2017	*sul2_3*	100	816/816	52	156..971	sulphonamide
i123	2017	*tet(A)_4*	94.92	1200/1200	48	1655..2854	tetracycline
i123	2017	*tet(B)_2*	100	1206/1206	36	679..1884	tetracycline
i123	2017	*mcr-9_1*	100	1618/1620	59	258..1875	colistin
i124	2017	*aac(6')-Iaa_1*	97.49	438/438	1	574093..574530	aminoglycoside
i124	2017	*aph(3')-Ia_7*	100	816/816	39	220..1035	aminoglycoside
i124	2017	*blaCTX-M-1_1*	100	876/876	30	186..1061	beta-lactam
i124	2017	*dfrA1_10*	99.79	474/474	17	2228..2701	trimethoprim
i124	2017	*dfrA1_8*	99.79	474/474	17	2228..2701	trimethoprim
i124	2017	*dfrA1_9*	99.79	474/474	17	2228..2701	trimethoprim
i124	2017	*sul1_2*	100	840/840	17	3234..4073	sulphonamide
i124	2017	*tet(A)_4*	100	1200/1200	17	13715..14914	tetracycline
i125	2017	*aac(6')-Iaa_1*	97.94	438/438	1	416545..416982	aminoglycoside
i125	2017	*aph(3'')-Ib_3*	99.88	804/804	28	4609..5412	aminoglycoside
i125	2017	*aph(3'')-Ib_4*	99.88	804/804	28	4609..5412	aminoglycoside
i125	2017	*aph(3'')-Ib_5*	99.88	804/804	28	4609..5412	aminoglycoside
i125	2017	*aph(6)-Id_1*	100	837/837	28	3773..4609	aminoglycoside
i125	2017	*blaCMY-2_1*	100	1146/1146	15	57975..59120	beta-lactam
i125	2017	*blaTEM-1B_1*	100	861/861	31	246..1106	beta-lactam
i125	2017	*fosA7_1*	97.16	423/423	5	18017..18439	fosfomycin
i125	2017	*qnrS1_1*	100	657/657	26	15931..16587	quinolone
i125	2017	*sul3_2*	100	792/792	31	1364..2155	sulphonamide
i125	2017	*tet(A)_4*	99.92	1200/1200	26	10077..11276	tetracycline
i126	2018	*aac(6')-Iaa_1*	97.49	438/438	1	620847..621284	aminoglycoside

i126	2018	*blaTEM-52C_2*	100	861/861	16	26487..27347	beta-lactam
i127	2018	*aac(3)-IV_1*	100	777/777	32	11487..12263	aminoglycoside
i127	2018	*aac(6')-Iaa_1*	97.49	438/438	1	620845..621282	aminoglycoside
i127	2018	*aadA1_3*	99.75	792/792	14	115763..116554	aminoglycoside
i127	2018	*aadA1_5*	99.75	792/792	14	115763..116554	aminoglycoside
i127	2018	*aph(3')-Ia_7*	100	816/816	45	221..1036	aminoglycoside
i127	2018	*aph(4)-Ia_1*	100	1026/1026	32	10233..11258	aminoglycoside
i127	2018	*blaCTX-M-137_1*	92.35	876/876	41	2825..3700	beta-lactam
i127	2018	*blaCTX-M-65_1*	100	876/876	41	2825..3700	beta-lactam
i127	2018	*dfrA14_4*	100	474/474	34	3860..4333	trimethoprim
i127	2018	*floR_2*	98.19	1214/1215	32	2421..3634	phenicol
i127	2018	*sul1_2*	100	840/840	14	114419..115258	sulphonamide
i127	2018	*tet(A)_4*	100	1200/1200	14	100703..101902	tetracycline
i128	2018	*aac(6')-Iaa_1*	97.49	438/438	1	620845..621282	aminoglycoside
i128	2018	*aadA1_3*	99.75	792/792	28	18709..19500	aminoglycoside
i128	2018	*aadA1_5*	99.75	792/792	28	18709..19500	aminoglycoside
i128	2018	*aph(3')-Ia_7*	100	816/816	72	214..1029	aminoglycoside
i128	2018	*blaCTX-M-55_1*	100	876/876	39	176..1051	beta-lactam
i128	2018	*dfrA14_4*	100	474/474	33	2371..2844	trimethoprim
i128	2018	*sul1_2*	100	840/840	28	17365..18204	sulphonamide
i128	2018	*tet(A)_4*	100	1200/1200	28	3649..4848	tetracycline
i129	2018	*aac(6')-Iaa_1*	100	438/438	1	569630..570067	aminoglycoside
i129	2018	*aadA2b_1*	99.87	780/780	40	362..1141	aminoglycoside
i129	2018	*ant(2'')-Ia_1*	100	534/534	40	1211..1744	aminoglycoside
i129	2018	*blaCTX-M-137_1*	92.12	876/876	31	221..1096	beta-lactam
i129	2018	*blaCTX-M-9_1*	100	876/876	31	221..1096	beta-lactam
i129	2018	*catA1_1*	99.55	660/660	44	296..955	phenicol
i129	2018	*dfrA16_2*	100	474/474	49	222..695	trimethoprim

i129	2018	*qnrA1_1*	99.85	657/657	35	1509..2165	quinolone
i129	2018	*sul1_2*	100	840/840	45	271..1110	sulphonamide
i129	2018	*tet(A)_4*	94.92	1200/1200	33	1180..2379	tetracycline
i130	2018	*aac(3)-IV_1*	100	777/777	33	177..953	aminoglycoside
i130	2018	*aac(6')-Iaa_1*	97.49	438/438	1	620845..621282	aminoglycoside
i130	2018	*aadA1_3*	99.75	792/792	26	33730..34521	aminoglycoside
i130	2018	*aadA1_5*	99.75	792/792	26	33730..34521	aminoglycoside
i130	2018	*aph(4)-Ia_1*	100	1026/1026	33	1182..2207	aminoglycoside
i130	2018	*blaCTX-M-137_1*	92.35	876/876	42	2825..3700	beta-lactam
i130	2018	*blaCTX-M-65_1*	100	876/876	42	2825..3700	beta-lactam
i130	2018	*dfrA14_4*	100	474/474	35	3860..4333	trimethoprim
i130	2018	*floR_2*	98.19	1214/1215	33	8806..10019	phenicol
i130	2018	*sul1_2*	100	840/840	26	32386..33225	sulphonamide
i130	2018	*tet(A)_4*	100	1200/1200	26	18670..19869	tetracycline
i131	2018	*aac(6')-Iaa_1*	100	438/438	1	569630..570067	aminoglycoside
i131	2018	*aadA2b_1*	99.87	780/780	41	362..1141	aminoglycoside
i131	2018	*ant(2'')-Ia_1*	100	534/534	41	1211..1744	aminoglycoside
i131	2018	*blaCTX-M-137_1*	92.12	876/876	30	221..1096	beta-lactam
i131	2018	*blaCTX-M-9_1*	100	876/876	30	221..1096	beta-lactam
i131	2018	*catA1_1*	99.55	660/660	45	296..955	phenicol
i131	2018	*dfrA16_2*	100	474/474	51	144..617	trimethoprim
i131	2018	*qnrA1_1*	99.85	657/657	36	1509..2165	quinolone
i131	2018	*sul1_2*	100	840/840	46	271..1110	sulphonamide
i131	2018	*tet(A)_4*	94.92	1200/1200	33	1655..2854	tetracycline
i131	2018	*mcr-9_1*	100.0	1618/1620	40	258..1875	colistin

Table S5: PlasmidFinder output of all *Shigella* isolates used in this study

Isolate	Plasmid replicon	Identity (%)	Query/Template Length	Contig	Position in contig	ESBL gene on same contig

i1	Col(BS512)_1	100	233/233	306	1971..2203	-
i1	Col156_1	98.7	154/154	337	913..1066	-
i1	IncI1_1	97.89	142/142	190	6852..6993	-
i2	Col(BS512)_1	100	233/233	285	1636..1868	-
i2	Col156_1	98.7	154/154	142	913..1066	-
i2	IncB/O/K/Z_4	100	149/149	1	70569..70717	blaCTX-M-15_1
i2	IncFII_1	96.17	261/261	274	1548..1807	-
i3	Col(BS512)_1	100	233/233	339	1284..1516	-
i3	Col(MG828)_1	95.77	260/262	380	989..1247	-
i3	Col156_1	98.7	154/154	300	913..1066	-
i3	IncFII_1	96.17	261/261	360	1010..1269	-
i3	IncI1_1	100	142/142	41	11947..12088	-
i4	IncFII_1	96.17	261/261	200	1770..2029	-
i4	IncI1_1	100	142/142	4	69762..69903	-
i5	Col(BS512)_1	100	233/233	281	191..423	-
i5	Col156_1	98.7	154/154	154	8450..8603	-
i5	IncB/O/K/Z_4	96.6	147/149	1	10517..10663	blaCTX-M-15_1
i5	IncFII_1	96.17	261/261	242	1012..1271	-
i6	Col(BS512)_1	100	233/233	327	922..1154	-
i6	Col156_1	98.7	154/154	153	913..1066	-
i6	IncFII_1	96.17	261/261	268	2542..2801	-
i6	IncI1_1	97.89	142/142	61	6489..6630	blaCTX-M-3_1
i7	Col(BS512)_1	100	233/233	323	1045..1277	-
i7	Col156_1	98.7	154/154	147	913..1066	-
i7	IncFII_1	96.17	261/261	259	1013..1272	-
i7	IncI1_1	97.89	142/142	1	16204..16345	blaCTX-M-3_1

i8	Col(BS512)_1	100	233/233	287	1045..1277	-
i8	Col156_1	98.7	154/154	154	8450..8603	-
i8	IncI1_1	97.89	142/142	52	16763..16904	blaCTX-M-3_1
i9	Col(BS512)_1	100	233/233	287	1624..1856	-
i9	Col156_1	98.05	154/154	151	913..1066	-
i9	IncFII_1	96.17	261/261	245	2542..2801	-
i9	IncI1_1	99.3	142/142	1	96562..96703	blaCTX-M-1_1
i10	Col(BS512)_1	100	233/233	307	550..782	-
i10	Col156_1	98.7	154/154	272	2240..2393	-
i10	IncFII_1	100	261/261	23	26638..26898	blaCTX-M-15_1
i11	Col(BS512)_1	100	233/233	333	1718..1950	-
i11	Col156_1	98.7	154/154	141	8962..9115	-
i11	IncFII_1	100	261/261	24	26629..26889	blaCTX-M-15_1
i12	Col(BS512)_1	100	233/233	271	189..421	-
i12	Col156_1	98.7	154/154	152	8450..8603	-
i12	IncI1_1	97.89	142/142	1	14615..14756	blaCTX-M-15_1
i13	Col(BS512)_1	100	233/233	300	1796..2028	-
i13	Col156_1	98.7	154/154	156	8450..8603	-
i13	IncFII_1	100	261/261	119	3925..4185	-
i14	Col(BS512)_1	100	233/233	293	1214..1446	-
i14	Col156_1	98.7	154/154	170	913..1066	-
i14	IncFII_1	96.17	261/261	248	1012..1271	-
i14	IncI1_1	100	142/142	3	71213..71354	blaCMY-2_1
i15	Col(BS512)_1	100	233/233	298	716..948	-
i15	Col156_1	98.7	154/154	265	2291..2444	-
i15	IncB/O/K/Z_4	96.6	147/149	1	56384..56530	blaCTX-M-15_1

i16	Col(BS512)_1	100	233/233	281	1806..2038	-
i16	Col156_1	98.7	154/154	139	9086..9239	-
i16	IncB/O/K/Z_4	96.6	147/149	1	36150..36296	blaCTX-M-15_1
i17	Col(BS512)_1	100	233/233	308	784..1016	-
i17	Col156_1	98.7	154/154	141	8962..9115	-
i17	IncFII_1	96.17	261/261	278	984..1243	-
i17	IncI1_1	100	142/142	188	6742..6883	blaCTX-M-15_1
i18	Col(BS512)_1	100	233/233	274	23..255	
i18	Col156_1	98.7	154/154	145	913..1066	-
i18	IncFII_1	96.17	261/261	355	640..899	-
i18	IncI1_1	100	142/142	1	76270..76411	blaCTX-M-15_1
i19	Col(BS512)_1	100	233/233	284	433..665	-
i19	Col156_1	98.7	154/154	153	913..1066	-
i19	IncI1_1	100	142/142	1	69392..69533	blaCTX-M-55_1
i20	Col(BS512)_1	100	233/233	273	1898..2130	-
i20	Col156_1	98.7	154/154	150	8450..8603	-
i20	IncI1_1	100	142/142	2	15900..16041	blaCTX-M-55_1
i21	Col(BS512)_1	100	208/233	272	2009..2216	-
i21	Col156_1	98.7	154/154	149	913..1066	-
i21	IncI1_1	100	142/142	2	68775..68916	blaCTX-M-55_1
i22	Col(BS512)_1	100	233/233	285	1156..1388	-
i22	Col156_1	98.7	154/154	151	8450..8603	-
i22	IncFII_1	96.17	261/261	344	317..576	-
i22	IncI1_1	100	142/142	1	17046..17187	blaCTX-M-15_1
i23	Col(BS512)_1	100	233/233	274	1022..1254	-
i23	Col156_1	98.7	154/154	153	913..1066	-

i23	IncB/O/K/Z_4	96.6	147/149	1	22478..22624	blaCTX-M-15_1
i24	Col(BS512)_1	100	233/233	289	1249..1481	-
i24	Col156_1	98.7	154/154	170	913..1066	-
i24	IncFII_1	96.17	261/261	247	2542..2801	-
i24	IncI1_1	97.89	142/142	1	15522..15663	blaCTX-M-3_1
i25	Col(BS512)_1	100	233/233	289	1889..2121	-
i25	Col156_1	98.7	154/154	148	8450..8603	-
i25	IncB/O/K/Z_4	96.6	147/149	1	82286..82432	blaCTX-M-15_1
i25	IncFII_1	96.17	261/261	247	1012..1271	-
i26	Col(BS512)_1	100	233/233	280	913..1145	-
i26	Col156_1	98.7	154/154	157	913..1066	-
i26	IncFII_1	100	261/261	121	3452..3712	-
i26	IncI1_1	97.89	142/142	11	10352..10493	-
i27	Col(BS512)_1	100	233/233	276	773..1005	-
i27	Col156_1	98.7	154/154	155	8450..8603	-
i27	IncI1_1	100	142/142	64	16008..16149	blaCTX-M-55_1
i27	IncX4_2	98.74	712/712	36	9604..10315	-
i28	Col(BS512)_1	100	233/233	291	1971..2203	-
i28	Col156_1	98.7	154/154	151	913..1066	-
i28	IncFII_1	96.17	261/261	248	1010..1269	-
i28	IncI1_1	100	142/142	2	56178..56319	blaCTX-M-55_1
i29	Col(BS512)_1	99.57	233/233	289	220..452	-
i29	Col156_1	98.7	154/154	174	6921..7074	-
i29	IncFII_1	96.17	261/261	272	1583..1842	-
i29	IncI1_1	100	142/142	13	12401..12542	blaCTX-M-15_1
i30	Col(BS512)_1	100	233/233	289	1800..2032	-

i30	Col156_1	98.7	154/154	172	6921..7074	-
i30	IncFII_1	96.17	261/261	248	2542..2801	-
i30	IncI1_1	100	142/142	1	77163..77304	blaCTX-M-15_1
i31	Col(BS512)_1	100	233/233	294	201..433	-
i31	Col156_1	98.7	154/154	152	8450..8603	-
i31	ColpVC_1	100	193/193	300	1811..2003	-
i31	IncFII_1	96.17	261/261	252	2542..2801	-
i31	IncI1_1	97.89	142/142	1	69196..69337	blaCTX-M-3_1
i32	IncFII_1	95.78	261/261	120	2480..2739	-
i33	Col(MG828)_1	96.92	260/262	282	1346..1605	-
i33	Col156_1	98.7	154/154	152	8450..8603	-
i33	IncFII_1	100	261/261	41	3925..4185	-
i33	IncI1_1	100	142/142	4	4487..4628	-
i34	Col(BS512)_1	100	233/233	273	1033..1265	-
i34	Col156_1	98.7	154/154	147	913..1066	-
i34	IncFII_1	100	261/261	11	30538..30798	blaCTX-M-15_1
i35	Col(BS512)_1	100	233/233	287	713..945	-
i35	Col156_1	98.7	154/154	151	8450..8603	-
i35	IncB/O/K/Z_4	96.6	147/149	1	10390..10536	blaCTX-M-15_1
i36	Col(BS512)_1	100	233/233	266	1191..1423	-
i36	Col156_1	98.7	154/154	171	6921..7074	-
i37	Col(BS512)_1	100	233/233	286	642..874	-
i37	Col156_1	98.7	154/154	146	913..1066	-
i37	IncFII_1	100	261/261	41	3925..4185	-
i38	Col(BS512)_1	100	233/233	295	1863..2095	-
i38	Col156_1	98.7	154/154	167	6921..7074	-

i38	IncFII_1	96.17	261/261	246	2542..2801	-
i39	Col(BS512)_1	100	233/233	268	312..544	-
i39	Col156_1	98.7	154/154	150	913..1066	-
i39	IncB/O/K/Z_4	100	149/149	2	76036..76184	blaCTX-M-15_1
i39	IncFIB(pLF82)_1	100	560/560	1	32552..33111	-
i40	Col(BS512)_1	100	233/233	297	1854..2086	-
i40	Col156_1	98.7	154/154	142	9087..9240	-
i40	IncB/O/K/Z_4	96.6	147/149	1	10479..10625	blaCTX-M-15_1
i40	IncFII_1	96.17	261/261	252	1012..1271	-
i41	Col(BS512)_1	100	233/233	287	898..1130	-
i41	Col156_1	98.7	154/154	154	913..1066	-
i41	IncFII_1	96.17	261/261	246	2542..2801	-
i42	Col(BS512)_1	100	233/233	290	836..1068	-
i42	Col156_1	98.7	154/154	172	6921..7074	-
i42	IncFII_1	100	261/261	41	26648..26908	-
i43	Col(BS512)_1	100	233/233	287	312..544	-
i43	Col156_1	98.7	154/154	154	913..1066	-
i43	IncFII_1	96.17	261/261	310	45..304	-
i43	IncI1_1	100	142/142	21	24387..24528	blaCTX-M-15_1
i44	Col(BS512)_1	100	233/233	296	399..631	-
i44	Col156_1	98.7	154/154	152	8450..8603	-
i44	IncFII_1	100	261/261	44	25976..26236	-
i44	IncI1_1	100	142/142	80	3822..3963	-
i45	Col(BS512)_1	100	233/233	273	59..291	-
i45	Col156_1	98.7	154/154	170	6921..7074	-
i45	IncI1_1	99.3	142/142	24	5051..5192	blaCTX-M-1_1

i46	Col(BS512)_1	100	233/233	290	1877..2109	-
i46	Col156_1	98.7	154/154	151	913..1066	-
i46	IncFII_1	100	261/261	41	3925..4185	-
i47	Col(BS512)_1	100	233/233	271	304..536	-
i47	Col156_1	98.7	154/154	140	9353..9506	-
i47	IncI1_1	99.3	142/142	1	5051..5192	blaCTX-M-1_1
i48	Col(BS512)_1	100	233/233	273	1680..1912	-
i48	Col156_1	98.7	154/154	149	913..1066	-
i48	IncI1_1	99.3	142/142	1	5051..5192	blaCTX-M-1_1
i49	Col(BS512)_1	100	233/233	276	1365..1597	-
i49	Col156_1	98.7	154/154	154	8450..8603	-
i49	IncFII_1	100	261/261	37	3925..4185	-
i50	Col(BS512)_1	100	233/233	289	1379..1611	-
i50	Col156_1	98.7	154/154	159	913..1066	-
i50	IncFII_1	100	261/261	245	3452..3712	-
i51	Col(BS512)_1	100	233/233	279	1135..1367	-
i51	Col156_1	98.7	154/154	145	8962..9115	-
i51	IncFII_1	100	261/261	198	2040..2300	-
i51	IncI1_1	100	142/142	4	65317..65458	-
i52	Col(BS512)_1	100	233/233	291	535..767	-
i52	Col156_1	98.7	154/154	151	8450..8603	-
i52	IncFII_1	96.17	261/261	247	1012..1271	-
i52	IncI1_1	99.3	142/142	71	15972..16113	-
i53	Col(BS512)_1	100	233/233	275	279..511	-
i53	Col156_1	98.7	154/154	171	6921..7074	-
i53	IncI1_1	99.3	142/142	69	5051..5192	-

i54	Col(BS512)_1	100	233/233	278	1017..1249	-
i54	Col156_1	98.7	154/154	153	913..1066	-
i54	IncFII_1	100	261/261	39	3925..4185	-
i55	Col(BS512)_1	100	233/233	272	399..631	-
i55	Col156_1	98.7	154/154	169	6921..7074	-
i55	IncI1_1	99.3	142/142	11	48552..48693	blaCTX-M-1_1
i56	Col(BS512)_1	100	233/233	272	1706..1938	-
i56	Col156_1	98.7	154/154	151	913..1066	-
i56	IncI1_1	99.3	142/142	68	16528..16669	-
i57	Col(BS512)_1	100	233/233	293	189..421	-
i57	Col156_1	98.7	154/154	155	913..1066	-
i57	IncFII_1	100	261/261	38	3925..4185	-
i58	Col(BS512)_1	100	233/233	271	436..668	-
i58	Col156_1	98.7	154/154	154	8450..8603	-
i58	IncFII_1	100	261/261	38	26630..26890	-
i59	Col(BS512)_1	100	233/233	315	1769..2001	-
i59	Col156_1	98.7	154/154	183	918..1071	-
i59	IncFII_1	100	261/261	264	395..655	-
i59	IncI1_1	100	142/142	58	15241..15382	-
i59	IncI2_1	98.42	316/316	10	27515..27830	-
i60	Col(BS512)_1	100	233/233	276	1477..1709	-
i60	Col156_1	98.7	154/154	157	8450..8603	-
i60	IncFII_1	100	261/261	39	26666..26926	-
i60	IncI1_1	100	142/142	62	8240..8381	-
i60	IncI2_1	98.42	316/316	10	27514..27829	-
i61	Col(BS512)_1	100	233/233	701	1625..1857	-

i61	Col156_1	96.1	154/154	158	954..1107	-
i61	IncFII_1	100	261/261	1	44806..45066	-
i62	Col(BS512)_1	100	233/233	289	163..395	-
i62	Col156_1	98.7	154/154	151	913..1066	-
i62	IncFII_1	96.17	261/261	249	2542..2801	-
i62	IncI1_1	97.89	142/142	1	15648..15789	blaCTX-M-3_1
i63	Col(BS512)_1	100	233/233	270	959..1191	-
i63	Col156_1	98.7	154/154	154	913..1066	-
i63	IncFII_1	100	261/261	200	1295..1555	-
i64	Col(BS512)_1	100	233/233	367	630..862	-
i64	Col156_1	98.7	154/154	154	8450..8603	-
i64	IncFII_1	100	261/261	38	4019..4279	-
i65	Col(BS512)_1	100	233/233	270	92..324	-
i65	Col156_1	98.7	154/154	156	913..1066	-
i65	IncFII_1	100	261/261	37	26630..26890	-
i66	IncFII_1	100	261/261	11	57980..58240	-
i67	Col(BS512)_1	100	233/233	286	1870..2102	-
i67	Col156_1	98.7	154/154	155	8449..8602	-
i67	IncB/O/K/Z_1	96.69	151/151	84	4457..4607	-
i67	IncFII_1	100	261/261	152	4517..4777	-
i68	Col(BS512)_1	100	233/233	302	650..882	-
i68	Col156_1	98.7	154/154	156	913..1066	-
i68	IncB/O/K/Z_1	96.69	151/151	84	14490..14640	-
i68	IncFII_1	100	261/261	153	4936..5196	-
i69	Col(BS512)_1	100	233/233	275	1018..1250	-
i69	Col156_1	98.7	154/154	148	8450..8603	-

i69	IncB/O/K/Z_1	100	151/151	1	15939..16089	blaCTX-M-14_1
i70	Col(BS512)_1	100	233/233	287	61..293	-
i70	Col156_1	98.7	154/154	155	8449..8602	-
i70	IncB/O/K/Z_1	96.69	151/151	84	14490..14640	-
i70	IncFII_1	100	261/261	152	4936..5196	-
i71	Col(BS512)_1	100	233/233	282	1208..1440	-
i71	Col156_1	98.7	154/154	156	8449..8602	-
i71	IncB/O/K/Z_1	96.69	151/151	83	14490..14640	-
i71	IncFII_1	100	261/261	153	4936..5196	-
i72	Col(BS512)_1	100	233/233	298	1622..1854	-
i72	Col156_1	98.7	154/154	155	8449..8602	-
i72	IncB/O/K/Z_1	96.69	151/151	81	4457..4607	-
i72	IncFII_1	100	261/261	152	4936..5196	-
i73	Col(BS512)_1	100	233/233	288	1284..1516	-
i73	Col(KPHS6)_1	100	178/178	326	1209..1386	-
i73	Col156_1	98.7	154/154	150	8450..8603	-
i73	IncB/O/K/Z_4	96.6	147/149	2	10517..10663	blaCTX-M-15_1
i73	IncFII_1	96.17	261/261	247	2542..2801	-
i74	Col(BS512)_1	100	233/233	283	713..945	-
i74	Col156_1	98.7	154/154	154	8449..8602	-
i74	IncB/O/K/Z_1	96.69	151/151	82	14490..14640	-
i74	IncFII_1	100	261/261	151	4517..4777	-
i75	Col(BS512)_1	100	233/233	304	180..412	-
i75	Col156_1	98.7	154/154	149	8961..9114	-
i75	IncB/O/K/Z_1	96.69	151/151	85	14490..14640	-
i75	IncFII_1	100	261/261	155	4936..5196	-

i76	Col(BS512)_1	100	233/233	283	1068..1300	-
i76	Col156_1	98.7	154/154	146	8961..9114	-
i76	IncB/O/K/Z_1	96.69	151/151	84	14490..14640	-
i76	IncFII_1	100	261/261	152	4936..5196	-
i77	Col(BS512)_1	100	233/233	307	179..411	-
i77	Col156_1	98.7	154/154	158	8449..8602	-
i77	IncB/O/K/Z_1	96.69	151/151	86	4457..4607	-
i77	IncFII_1	100	261/261	155	4936..5196	-
i78	Col(BS512)_1	100	233/233	306	83..315	-
i78	Col156_1	98.7	154/154	159	8449..8602	-
i78	IncB/O/K/Z_1	96.69	151/151	199	1712..1862	-
i78	IncFII_1	100	261/261	210	4517..4777	-
i79	Col(BS512)_1	100	233/233	306	1067..1299	-
i79	Col156_1	98.7	154/154	148	913..1066	-
i79	IncB/O/K/Z_1	96.69	151/151	85	4457..4607	-
i79	IncFII_1	100	261/261	153	4517..4777	-
i80	Col(BS512)_1	100	233/233	281	312..544	-
i80	Col156_1	98.7	154/154	154	913..1066	-
i80	IncB/O/K/Z_1	96.69	151/151	84	4457..4607	-
i80	IncFII_1	100	261/261	151	4936..5196	-
i81	Col(BS512)_1	100	233/233	277	1535..1767	-
i81	Col156_1	98.7	154/154	140	8962..9115	-
i81	IncFII_1	100	261/261	5	26639..26899	blaCTX-M-15_1
i82	Col(BS512)_1	100	233/233	288	520..752	-
i82	Col156_1	98.7	154/154	151	913..1066	-
i82	IncFII_1	96.17	261/261	246	1012..1271	-

i82	IncI1_1	97.89	142/142	1	15541..15682	blaCTX-M-3_1
i83	Col(BS512)_1	100	233/233	287	6..238	-
i83	Col156_1	98.7	154/154	147	913..1066	-
i83	IncFII_1	100	261/261	41	3451..3711	-
i84	IncFII_1	100	261/261	11	3452..3712	-
i85	Col(BS512)_1	100	233/233	282	1319..1551	-
i85	Col156_1	98.7	154/154	152	8450..8603	-
i85	IncB/O/K/Z_1	96.69	151/151	1	73851..74001	-
i85	IncFII_1	100	261/261	40	26630..26890	-
i86	Col(BS512)_1	100	233/233	297	501..733	-
i86	Col156_1	98.7	154/154	144	8962..9115	-
i86	IncB/O/K/Z_4	96.6	147/149	3	10390..10536	blaCTX-M-15_1
i86	IncFII_1	96.17	261/261	289	1012..1271	-
i87	Col(BS512)_1	100	233/233	274	951..1183	-
i87	Col156_1	98.7	154/154	146	8962..9115	-
i87	IncFII_1	100	261/261	43	25958..26218	-
i87	IncI2_1	98.14	322/316	9	29279..29600	-
i88	Col(BS512)_1	100	233/233	295	520..752	-
i88	Col156_1	98.7	154/154	155	913..1066	-
i88	IncFII_1	96.17	261/261	249	2542..2801	-
i88	IncI1_1	100	142/142	1	5172..5313	blaCTX-M-15_1
i88	IncI2_1	98.42	316/316	9	30687..31002	-
i89	IncFII_1	100	261/261	2	57221..57481	-
i90	Col(BS512)_1	100	233/233	300	1910..2142	-
i90	Col156_1	98.7	154/154	159	8450..8603	-
i90	IncFII_1	100	261/261	40	26630..26890	-

i90	IncI2_1	98.14	322/316	10	28125..28446	-
i91	Col(BS512)_1	100	233/233	284	520..752	-
i91	Col156_1	98.7	154/154	141	913..1066	-
i91	IncFII_1	96.17	261/261	379	560..819	-
i91	IncI1_1	97.89	142/142	1	68012..68153	blaCTX-M-3_1
i92	Col(BS512)_1	100	233/233	270	446..678	-
i92	Col156_1	98.7	154/154	150	8450..8603	-
i92	IncFIB(pLF82)_1	99.82	560/560	1	50062..50621	-
i92	IncI1_1	97.89	142/142	2	16732..16873	blaCTX-M-3_1
i93	Col(BS512)_1	100	233/233	292	288..520	-
i93	Col156_1	98.7	154/154	149	913..1066	-
i93	IncFII_1	100	261/261	10	26666..26926	blaCTX-M-15_1
i94	Col(BS512)_1	100	203/233	272	2026..2228	-
i94	Col156_1	98.7	154/154	152	8450..8603	-
i94	IncFII_1	100	261/261	40	4321..4581	-
i95	IncFIB(K)_1	98.93	560/560	28	37363..37922	-
i95	IncFII_1	100	261/261	148	4358..4618	-
i96	Col(BS512)_1	100	233/233	272	1021..1253	-
i96	Col156_1	98.7	154/154	152	8450..8603	-
i96	IncI1_1	97.89	142/142	2	70467..70608	blaCTX-M-3_1
i97	Col(BS512)_1	100	233/233	297	1110..1342	-
i97	Col156_1	98.7	154/154	156	8450..8603	-
i97	IncFII_1	96.17	261/261	249	2542..2801	-
i97	IncI1_1	97.89	142/142	57	15649..15790	blaCTX-M-3_1
i98	Col(BS512)_1	100	233/233	282	312..544	-
i98	Col156_1	98.7	154/154	153	8449..8602	-

i98	IncB/O/K/Z_1	96.69	151/151	83	4457..4607	-
i98	IncFII_1	100	261/261	150	4517..4777	-
i99	Col(BS512)_1	100	233/233	277	312..544	-
i99	Col156_1	98.7	154/154	151	8450..8603	-
i99	IncFII_1	100	261/261	21	13670..13930	-
i99	IncI1_1	100	142/142	2	77686..77827	-
i100	IncFIB(K)_1	98.93	560/560	7	42765..43324	-
i100	IncFII_1	95.78	261/261	123	10435..10694	-

Table S6: PlasmidFinder output of all *Salmonella* isolates used in this study

**Isolate**	**Plasmid replicon**	**Identity (%)**	**Query/Template Length**	**Contig**	**Position in contig**	**ESBL gene on same contig**
i101	IncHI2	100	327 / 327	14	15497..15823	-
i101	IncHI2A	99.52	630 / 630	14	30691..31320	-
i102	IncA/C2	100	417 / 417	23	30579..30995	-
i103	-	-	-	-	-	-
i104	Col(BS512)	100	233 / 233	47	312..544	-
i104	IncFIB(AP001918)	97.65	682 / 682	36	2185..2866	-
i104	IncI1	100	142 / 142	27	16626..16767	blaCTX-M-1_1
i105	IncFIB(S)	98.91	643 / 643	12	40548..41190	-
i105	IncFII	96.56	262 / 261	14	39967..40227	-
i105	IncFII(S)	100	262 / 262	19	1927..2188	-
i106	Col(MG828)	95	260 / 262	53	1080..1339	-
i106	Col156	96.71	152 / 154	39	3543..3694	-
i106	IncFIA	100	388 / 388	20	31612..31999	-
i106	IncFIB(AP001918)	97.36	682 / 682	20	16418..17099	-
i106	IncFII(pRSB107)	100	261 / 261	11	173236..173496	-

i107	-	-	-	-	-	-
i108	IncQ1	100	529 / 796	37	122..650	-
i109	IncQ1	100	529 / 796	37	3990..4518	-
i110	IncQ1	100	529 / 796	33	3990..4518	-
i111	IncHI2	100	327 / 327	19	37305..37631	-
i111	IncHI2A	100	630 / 630	19	21810..22439	-
i112	IncFIA	99.72	363 / 388	12	14452..14814	-
i112	IncFIB(AP001918)	98.39	682 / 682	12	139088..139769	-
i112	IncFIC(FII)	95.79	499 / 499	12	67801..68297	-
i112	IncHI2	100	327 / 327	10	118929..119255	-
i112	IncHI2A	100	630 / 630	10	134121..134750	-
i113	IncFIB(S)	100	643 / 643	15	44843..45485	-
i113	IncFII(S)	100	262 / 262	15	29497..29758	-
i113	IncI1	99.3	142 / 142	33	7340..7481	-
i114	Col156	95.33	107 / 154	49	3..109	-
i114	IncFIA	99.74	388 / 388	37	5631..6017	-
i114	IncFIB(AP001918)	98.09	682 / 682	57	642..1323	-
i114	IncFII(pCoo)	96.18	262 / 262	24	4512..4773	-
i114	IncI1	100	142 / 142	33	15241..15382	-
i114	IncQ1	100	529 / 796	61	122..650	-
i115	IncHI2	100	327 / 327	14	39918..40244	-
i115	IncHI2A	99.52	630 / 630	14	21305..21934	-
i116	IncHI2	100	327 / 327	12	5543..5869	-
i116	IncHI2A	99.52	630 / 630	12	23853..24482	-
i117	IncQ1	100	529 / 796	59	1164..1692	-
i117	IncY	98.82	765 / 765	35	24976..25740	-
i118	IncHI2	100	327 / 327	13	105295..105621	-

i118	IncHI2A	100	630 / 630	13	89800..90429	-
i119	IncFIB(S)	100	643 / 643	17	79905..80547	-
i119	IncFII(S)	100	262 / 262	17	1782..2043	-
i119	IncI1	100	142 / 142	29	15973..16114	-
i119	IncQ1	100	796 / 796	34	4827..5622	-
i120	IncFIB(S)	100	643 / 643	14	11229..11871	-
i120	IncFII(S)	100	262 / 262	14	89818..90079	-
i120	IncI1	97.89	142 / 142	17	70282..70423	-
i121	IncFIA	99.72	363 / 388	13	126921..127283	-
i121	IncFIB(AP001918)	98.39	682 / 682	13	1962..2643	-
i121	IncFIC(FII)	95.79	499 / 499	13	73434..73930	-
i121	IncHI2	100	327 / 327	11	118929..119255	-
i121	IncHI2A	100	630 / 630	11	134121..134750	-
i122	IncA/C2	100	417 / 417	16	17621..18037	-
i123	IncHI2	100	327 / 327	11	118680..119006	-
i123	IncHI2A	100	630 / 630	11	133872..134501	-
i123	IncQ1	100	529 / 796	52	2310..2838	-
i123	p0111	99.32	885 / 885	14	68166..69050	-
i124	-	-	-	-	-	-
i125	IncI1	100	142 / 142	15	71414..71555	-
i125	IncX1	98.66	374 / 374	26	2779..3152	-
i126	IncI1	99.3	142 / 142	16	16133..16274	-
i127	-	-	-	-	-	-
i128	IncI1	100	142 / 142	42	514..655	-
i128	IncX4	100	374 / 374	24	1144..1517	-
i129	IncHI2	100	327 / 327	7	105295..105621	-
i129	IncHI2A	100	630 / 630	7	89800..90429	-

i130	-	-	-	-	-	-
i131	IncHI2	100	327 / 327	10	118929..119255	-
i131	IncHI2A	100	630 / 630	10	134121..134750	-
i131	IncI2	100	324 / 324	19	25861..26184	-

Table S7: prediction with BLAST, mlplasmid, plasflow and mobsuite on location (chromosome or plasmid) of each ESBL containing short-read contig of *Shigella*

isolate	contig	size of contig (bp)	ESBL gene | plasmid replicon on same contig	blast	mlplasmid	plasflow	mobsuite
i1	contig3	64128	blaCTX-M-15_1|no	plasmid	Plasmid	plasmid	plasmid
i2	contig1	92572	blaCTX-M-15_1|IncB/O/K/Z_4	plasmid	Plasmid	plasmid	plasmid
**i3**	**contig6**	**48366**	**blaCTX-M-14_1|no**	**chromosome**	**Chromosome**	**plasmid**	**chromosome**
**i4**	**contig261**	**1760**	**blaCTX-M-15_1|no**	**unclassified**	**Chromosome**	**unclassified**	**plasmid**
**i5**	**contig1**	**93332**	**blaCTX-M-15_1|IncB/O/K/Z_4**	**plasmid**	**Plasmid**	**plasmid**	**plasmid**
i6	contig61	22277	blaCTX-M-3_1|IncI1_1	plasmid	Plasmid	plasmid	plasmid
i7	contig1	84865	blaCTX-M-3_1|IncI1_1	plasmid	Plasmid	plasmid	plasmid
i8	contig52	26547	blaCTX-M-3_1|IncI1_1	plasmid	Plasmid	plasmid	plasmid
**i9**	**contig1**	**112567**	**blaCTX-M-1_1|IncI1_1**	**plasmid**	**Plasmid**	**plasmid**	**plasmid**
i10	contig23	35881	blaCTX-M-15_1|IncFII_1	plasmid	Plasmid	plasmid	plasmid
i11	contig24	35872	blaCTX-M-15_1|IncFII_1	plasmid	Plasmid	plasmid	plasmid
**i12**	**contig1**	**89966**	**blaCTX-M-15_1|IncI1_1**	**plasmid**	**Plasmid**	**plasmid**	**plasmid**
i13	contig285	2873	blaCTX-M-15_1|no	unclassified	Plasmid	unclassified	plasmid
i14	contig3	87872	blaCMY-2_1|IncI1_1	plasmid	Plasmid	plasmid	plasmid
i15	contig1	95949	blaCTX-M-15_1|IncB/O/K/Z_4	plasmid	Plasmid	plasmid	plasmid
i16	contig1	95949	blaCTX-M-15_1|IncB/O/K/Z_4	plasmid	Plasmid	plasmid	plasmid
i17	contig188	7172	blaCTX-M-15_1|IncI1_1	plasmid	Plasmid	plasmid	plasmid
i18	contig1	92454	blaCTX-M-15_1|IncI1_1	plasmid	Plasmid	plasmid	plasmid
i19	contig1	85432	blaCTX-M-55_1|IncI1_1	plasmid	Plasmid	plasmid	plasmid

**i20**	**contig2**	**85149**	**blaCTX-M-55_1|IncI1_1**	**plasmid**	**Plasmid**	**plasmid**	**plasmid**
i21	contig2	85391	blaCTX-M-55_1|IncI1_1	plasmid	Plasmid	plasmid	plasmid
i22	contig1	94050	blaCTX-M-15_1|IncI1_1	plasmid	Plasmid	plasmid	plasmid
i23	contig1	91344	blaCTX-M-15_1|IncB/O/K/Z_4	plasmid	Plasmid	plasmid	plasmid
i24	contig1	84725	blaCTX-M-3_1|IncI1_1	plasmid	Plasmid	plasmid	plasmid
i25	contig1	9327	blaCTX-M-15_1|IncB/O/K/Z_4	plasmid	Plasmid	plasmid	plasmid
i26	contig268	2958	blaCTX-M-15_1|no	unclassified	Plasmid	unclassified	plasmid
i27	contig64	23136	blaCTX-M-55_1|IncI1_1	plasmid	Plasmid	plasmid	plasmid
i28	contig2	86521	blaCTX-M-55_1|IncI1_1	plasmid	Plasmid	plasmid	plasmid
i29	contig13	55029	blaCTX-M-15_1|IncI1_1	plasmid	Plasmid	plasmid	plasmid
i30	contig1	93903	blaCTX-M-15_1|IncI1_1	plasmid	Plasmid	plasmid	plasmid
**i31**	**contig1**	**83102**	**blaCTX-M-3_1|IncI1_1**	**plasmid**	**Plasmid**	**plasmid**	**plasmid**
i32	contig79	19206	blaCTX-M-15_1|no	chromosome	Chromosome	plasmid	chromosome
i33	contig326	1092	blaCTX-M-15_1|no	unclassified	Chromosome	unclassified	plasmid
**i34**	**contig11**	**56782**	**blaCTX-M-15_1|IncFII_1**	**plasmid**	**Plasmid**	**plasmid**	**plasmid**
i35	contig1	92747	blaCTX-M-15_1|IncB/O/K/Z_4	plasmid	Plasmid	plasmid	plasmid
**i36**	**contig88**	**17349**	**blaCTX-M-15_1|no**	**Chromosome**	**Chromosome**	**plasmid**	**chromosome**
i37	contig272	2958	blaCTX-M-15_1|no	unclassified	Chromosome	plasmid	plasmid
**i38**	**contig1**	**111076**	**blaCTX-M-15_1|no**	**plasmid**	**Plasmid**	**plasmid**	**plasmid**
**i39**	**contig2**	**92189**	**blaCTX-M-15_1|IncB/O/K/Z_4**	**plasmid**	**Plasmid**	**plasmid**	**plasmid**
i40	contig1	89429	blaCTX-M-15_1|IncB/O/K/Z_4	unclassified	Plasmid	plasmid	plasmid
**i41**	**contig39**	**32264**	**blaCTX-M-15_1|no**	**unclassified**	**Chromosome**	**plasmid**	**chromosome**
i42	contig274	2958	blaCTX-M-15_1|no	plasmid or chromosome	Chromosome	plasmid	plasmid
i43	contig21	40571	blaCTX-M-15_1|IncI1_1	plasmid	Plasmid	plasmid	plasmid
i44	contig282	2873	blaCTX-M-15_1|no	unclassified	Plasmid	unclassified	plasmid
i45	contig24	39784	blaCTX-M-1_1|IncI1_1	plasmid	Plasmid	plasmid	plasmid
i46	contig276	2958	blaCTX-M-15_1|no	unclassified	Chromosome	plasmid	plasmid
i47	contig1	100611	blaCTX-M-1_1|IncI1_1	plasmid	Plasmid	plasmid	plasmid
i48	contig1	90001	blaCTX-M-1_1|IncI1_1	plasmid	Plasmid	plasmid	plasmid
i49	contig238	3964	blaCTX-M-15_1|no	unclassified	Plasmid	plasmid	plasmid

i50	contig251	3964	blaCTX-M-15_1|no	unclassified	Chromosome	plasmid	plasmid
i51	contig91	16981	blaCTX-M-55_1|no	plasmid	Plasmid	plasmid	plasmid
i52	contig5	69011	blaCTX-M-1_1|no	plasmid	Plasmid	plasmid	plasmid
i53	contig237	68563	blaCTX-M-1_1|no	plasmid	plasmid	plasmid	plasmid
**i54**	**contig237**	**3964**	**blaCTX-M-15_1|no**	**unclassified**	**Chromosome**	**chromosome**	**plasmid**
**i55**	**contig11**	**53743**	**blaCTX-M-1_1|IncI1_1**	**plasmid**	**Plasmid**	**plasmid**	**plasmid**
i56	contig237	3776	blaCTX-M-1_1|no	plasmid	Chromosome	unclassfied	plasmid
i57	contig248	3964	blaCTX-M-15_1|no	unclassified	Plasmid	plasmid	plasmid
i58	contig236	3962	blaCTX-M-15_1|no	unclassified	Plasmid	plasmid	plasmid
**i59**	**contig270**	**3964**	**blaCTX-M-15_1|no**	**unclassified**	**Chromosome**	**plasmid**	**plasmid**
i60	contig243	3964	blaCTX-M-15_1|no	unclassified	Chromosome	plasmid	plasmid
i61	contig354	3962	blaCTX-M-15_1|no	unclassified	Plasmid	plasmid	plasmid
i62	contig1	86359	blaCTX-M-3_1|IncI1_1	plasmid	Plasmid	plasmid	plasmid
i63	contig146	10115	blaCTX-M-15_1|no	plasmid	Chromosome	plasmid	plasmid
i64	contig245	3964	blaCTX-M-15_1|no	unclassified	Chromosome	plasmid	plasmid
i65	contig238	3964	blaCTX-M-15_1|no	unclassified	Plasmid	plasmid	plasmid
i66	contig201	4047	blaCTX-M-15_1|no	unclassified	Chromosome	plasmid	plasmid
**i67**	**contig281**	**2456**	**blaCTX-M-27_1|no**	**unclassified**	**Chromosome**	**plasmid**	**chromosome**
i68	contig296	2456	blaCTX-M-27_1|no	unclassified	Chromosome	plasmid	chromosome
**i69**	**contig1**	**93616**	**blaCTX-M-14_1|IncB/O/K/Z_1**	**unclassified**	**Plasmid**	**plasmid**	**plasmid**
i70	contig281	2455	blaCTX-M-27_1|no	unclassified	Chromosome	plasmid	chromosome
i71	contig278	2456	blaCTX-M-27_1|no	unclassified	Chromosome	plasmid	chromosome
i72	contig291	2454	blaCTX-M-27_1|no	unclassified	Chromosome	plasmid	chromosome
**i73**	**contig2**	**98328**	**blaCTX-M-15_1|IncB/O/K/Z_4**	**plasmid**	**Plasmid**	**plasmid**	**plasmid**
i74	contig279	2454	blaCTX-M-27_1|no	unclassified	Chromosome	plasmid	chromosome
i75	contig299	2454	blaCTX-M-27_1|no	unclassified	Chromosome	plasmid	chromosome
i76	contig279	2454	blaCTX-M-27_1|no	unclassified	Chromosome	plasmid	chromosome
i77	contig303	2455	blaCTX-M-27_1|no	unclassified	Chromosome	plasmid	chromosome
i78	contig301	2454	blaCTX-M-27_1|no	unclassified	Chromosome	plasmid	chromosome
i79	contig301	2454	blaCTX-M-27_1|no	unclassified	Chromosome	plasmid	chromosome

i80	contig277	2455	blaCTX-M-27_1|no	unclassified	Chromosome	plasmid	chromosome
i81	contig5	66446	blaCTX-M-15_1|IncFII_1	plasmid	Plasmid	plasmid	plasmid
i82	contig1	84218	blaCTX-M-3_1|IncI1_1	plasmid	Plasmid	plasmid	plasmid
**i83**	**contig107**	**14441**	**blaCTX-M-15_1|no**	**plasmid**	**Chromosome**	**plasmid**	**plasmid**
i84	contig236	2367	blaCTX-M-15_1|no	unclassified	Chromosome	plasmid	plasmid
i85	contig268	2958	blaCTX-M-15_1|no	unclassified	Chromosome	plasmid	plasmid
i86	contig3	97734	blaCTX-M-15_1|IncB/O/K/Z_4	plasmid	Plasmid	plasmid	plasmid
i87	contig238	3962	blaCTX-M-15_1|no	unclassified	Plasmid	plasmid	plasmid
i88	contig1	81242	blaCTX-M-15_1|IncI1_1	plasmid	Plasmid	plasmid	plasmid
i89	contig278	3964	blaCTX-M-15_1|no	unclassified	Chromosome	plasmid	plasmid
i90	contig251	3964	blaCTX-M-15_1|no	unclassified	Plasmid	plasmid	plasmid
i91	contig1	86499	blaCTX-M-3_1|IncI1_1	plasmid	Plasmid	plasmid	plasmid
i92	contig2	86634	blaCTX-M-3_1|IncI1_1	plasmid	Plasmid	plasmid	plasmid
i93	contig10	58601	blaCTX-M-15_1|IncFII_1	plasmid	Plasmid	plasmid	plasmid
i94	contig260	2958	blaCTX-M-15_1|no	unclassified	Chromosome	plasmid	plasmid
i95	contig98	15215	blaDHA-1_1|no	unclassified	Chromosome	plasmid	plasmid
i96	contig2	86256	blaCTX-M-3_1|IncI1_1	plasmid	Plasmid	plasmid	plasmid
i97	contig57	24273	blaCTX-M-3_1|IncI1_1	plasmid	Plasmid	plasmid	plasmid
i98	contig279	2454	blaCTX-M-27_1|no	unclassified	Chromosome	plasmid	plasmid
i99	contig141	10162	blaCTX-M-15_1|no	plasmid	Chromosome	plasmid	chromosome
i100	contig64	21568	blaCTX-M-15_1|no	plasmid	Plasmid	plasmid	plasmid

In Bold are all isolates of which a hybrid assembly was also made to confirm the real location of the ESBL gene

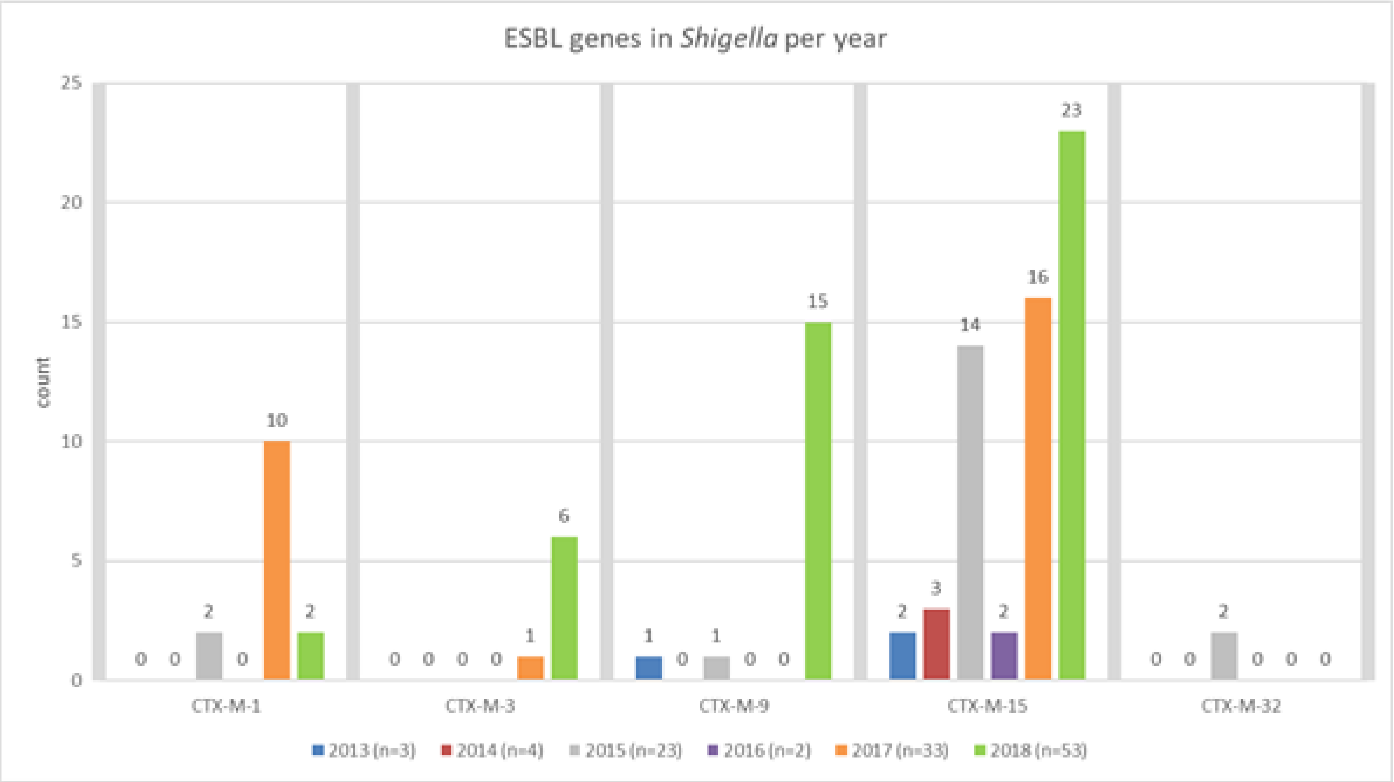

Figure S1: inhouse genotyping assays to determine CTX-M gene variants in *Shigella*.

A.

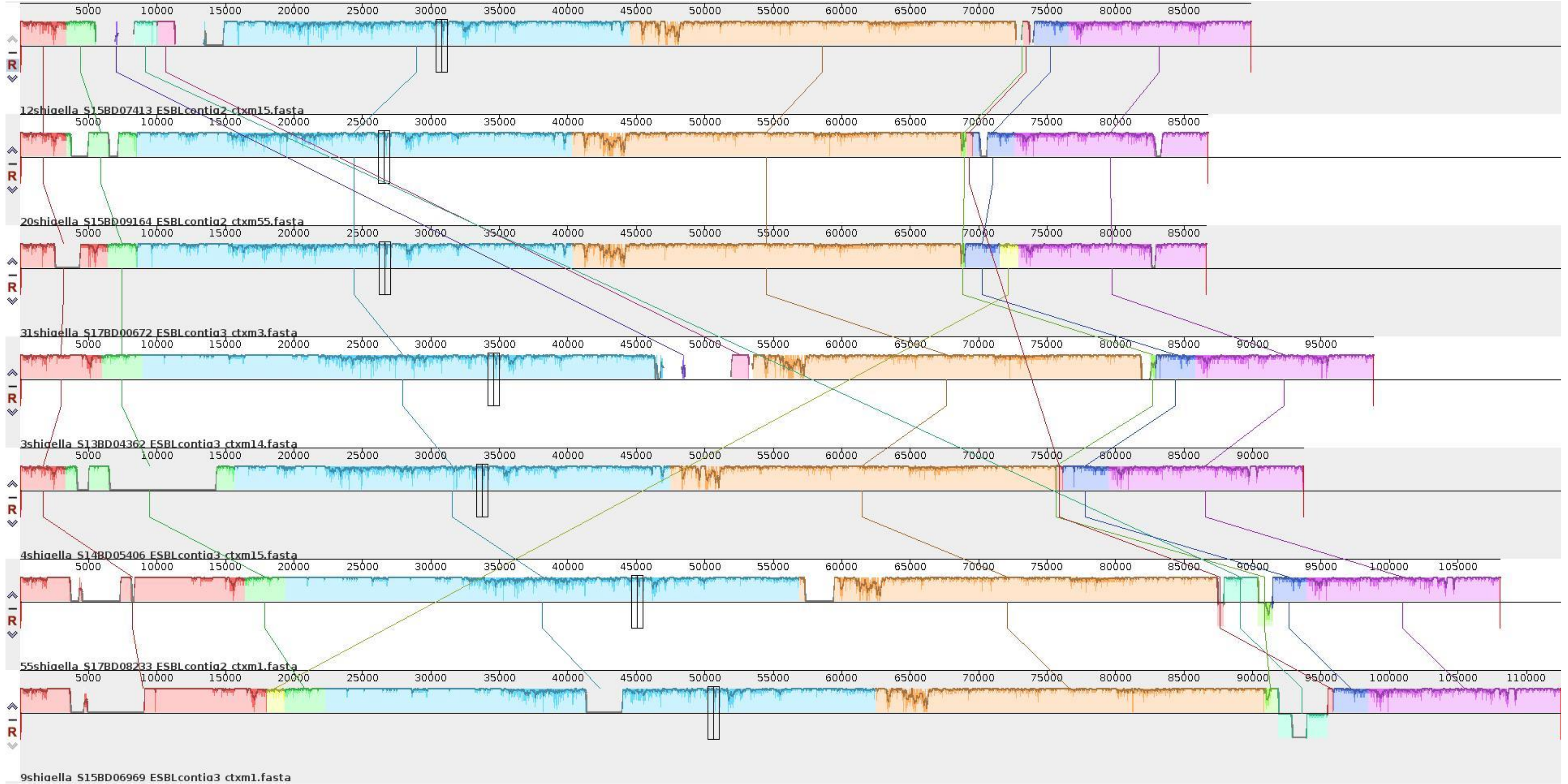

12shigella_S15BD07413_ESBLcontig2_ctxm15.fasta
20shigella_S15BD09164_ESBLcontig2_ctxm55.fasta
31shigella_S17BD00672_ESBLcontig3_ctxm3.fasta
3shigella_S13BD04362_ESBLcontig3_ctxm14.fasta
4shigella_S14BD05406_ESBLcontig3_ctxm15.fasta
55shigella_S17BD08233_ESBLcontig2_ctxm1.fasta
9shigella_S15BD06969_ESBLcontig3_ctxm1.fasta

B.

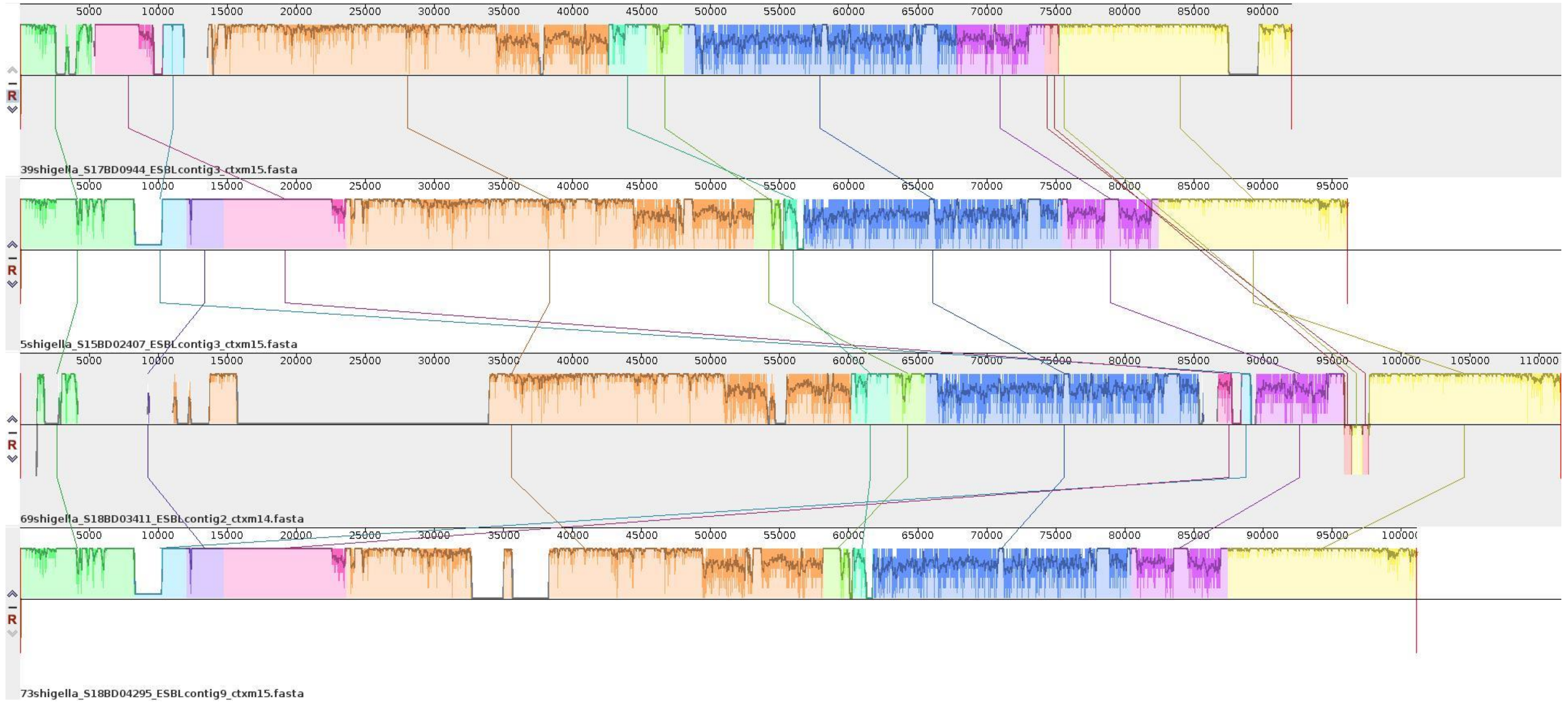
39shigella_S17BD0944_ESBLcontig3_ctxm15.fasta
5shigella_S15BD02407_ESBLcontig3_ctxm15.fasta
69shigella_S18BD03411_ESBLcontig2_ctxm14.fasta
73shigella_S18BD04295_ESBLcontig9_ctxm15.fasta

C.

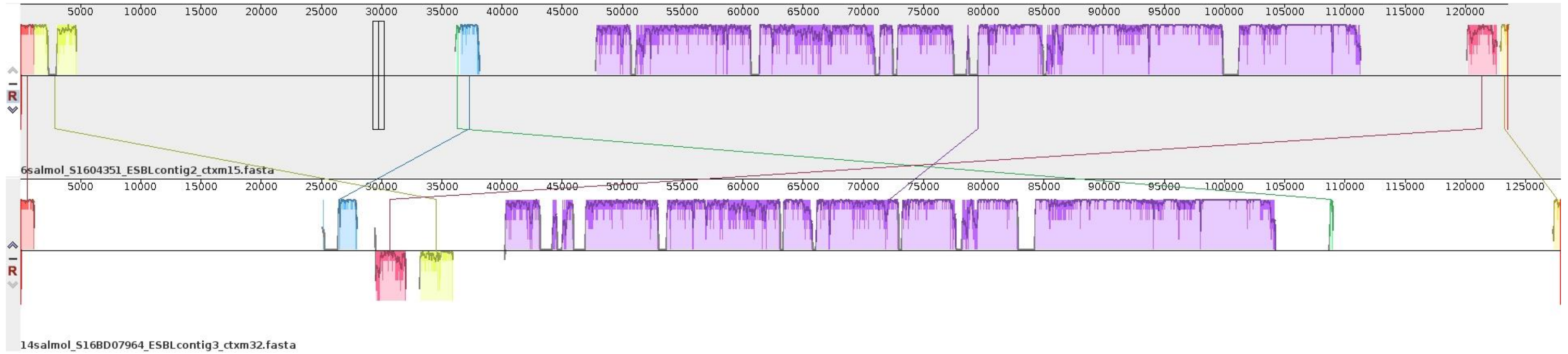

D.

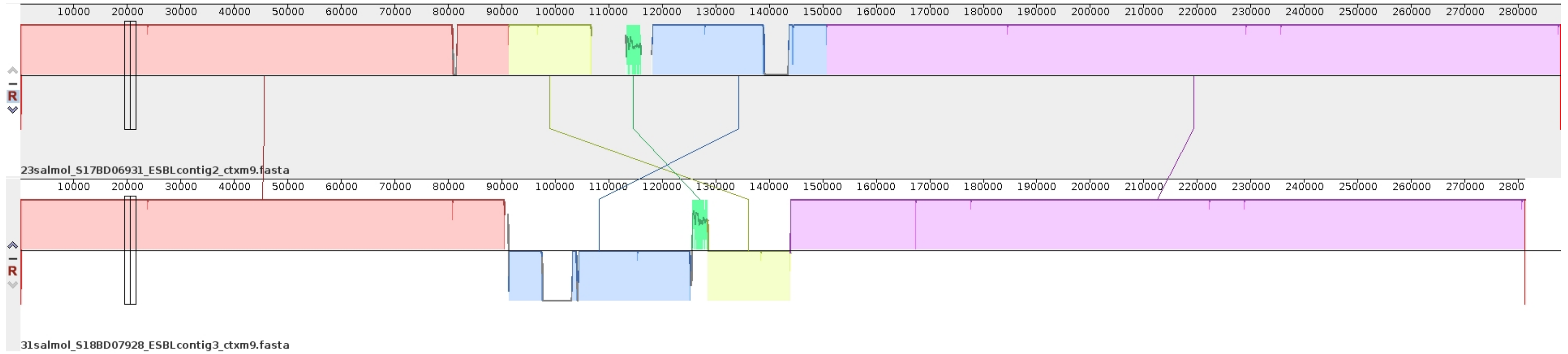

Figure S2: global alignment between all reconstructed IncI1-I(Gamma) (A.), IncB/O/K/Z (B.), IncFIA (C.) and IncHI2 (D.) plasmids. The colours indicate locally collinear blocks (LCBs), which are homologous regions without rearrangements between two or more sequences.

A.

Species: Shigella
Isolate: S13BD04362 (i3)
Structure: Plasmid (contig 3)

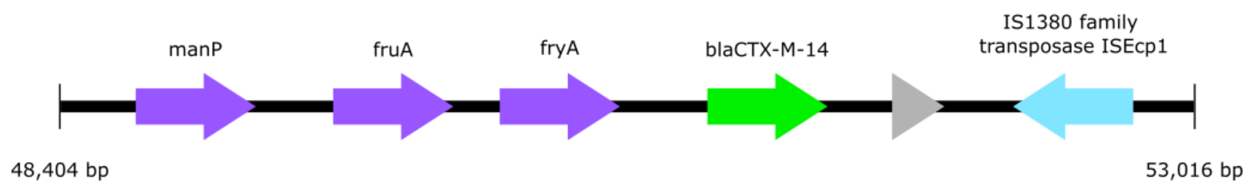

B.

Species: Shigella
Isolate: S14BD05406 (i4)
Structure: Plasmid (contig 3)

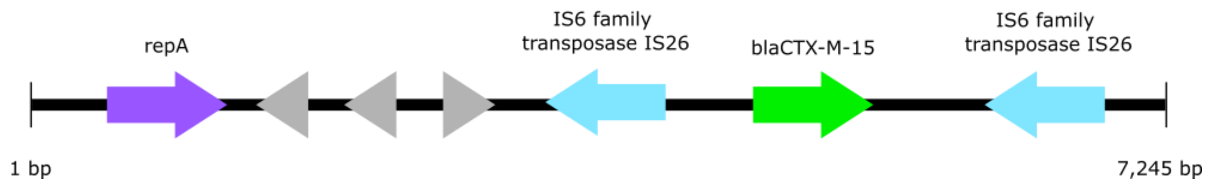

C.

Species: Shigella
Isolate: S15BD02407 (i5)
Structure: Plasmid (contig 3)

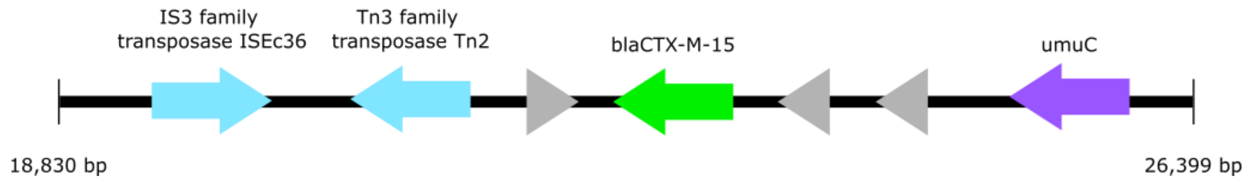

D.

Species: Shigella
Isolate: S15BD06969 (i9)
Structure: Plasmid (contig 3)

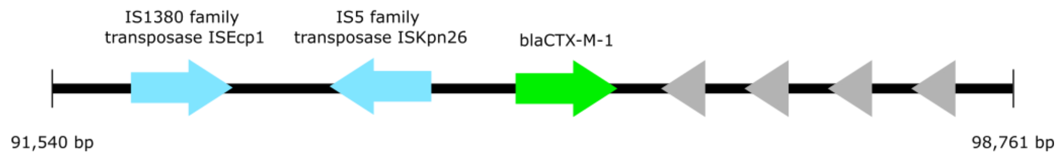

E.

Species: Shigella
Isolate: S15BD07413 (i12)
Structure: Plasmid (contig 3)

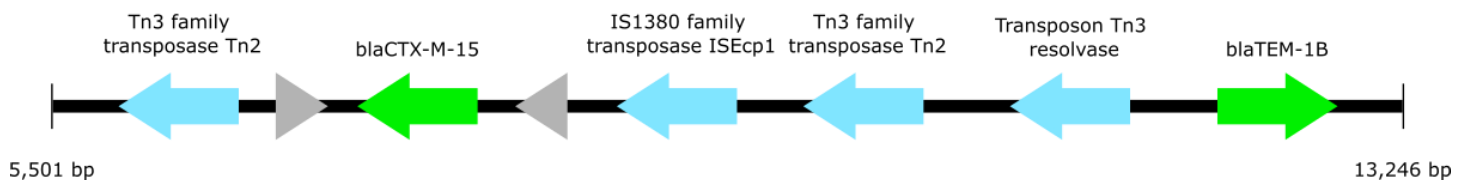

F.

Species: Shigella
Isolate: S15BD09164 (i20)
Structure: Plasmid (contig 2)

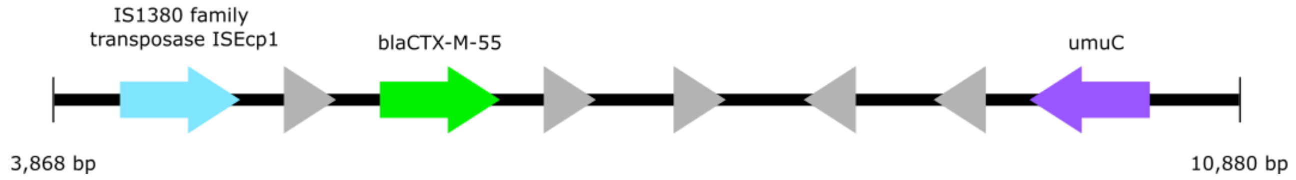

G.

Species: Shigella
Isolate: S17BD00672 (i31)
Structure: Plasmid (contig 3)

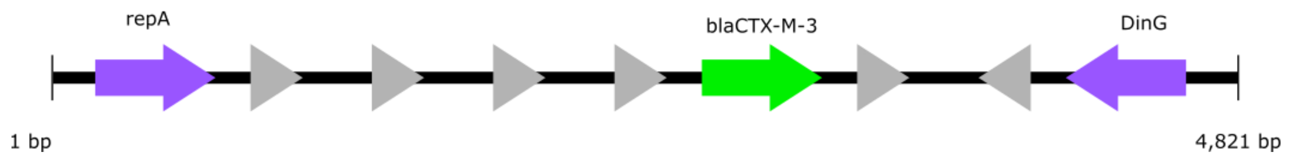

H.

Species: Shigella
Isolate: S17BD05916 (i38)
Structure: Plasmid (contig 3)

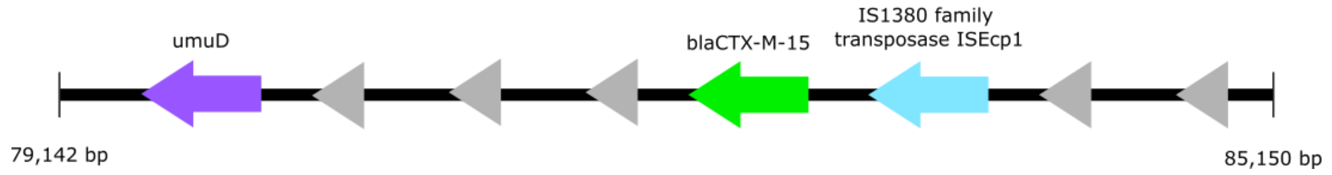

I.

Species: Shigella
Isolate: S17BD05944 (i39)
Structure: Plasmid (contig 3)

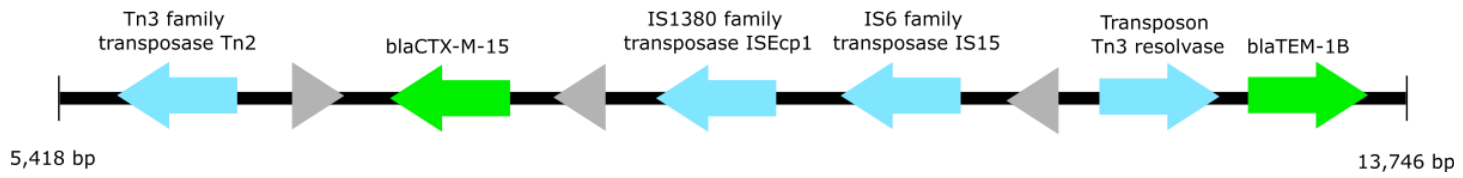

J.

Species: Shigella
Isolate: S17BD08179 (i54)
Structure: Plasmid (contig 2)

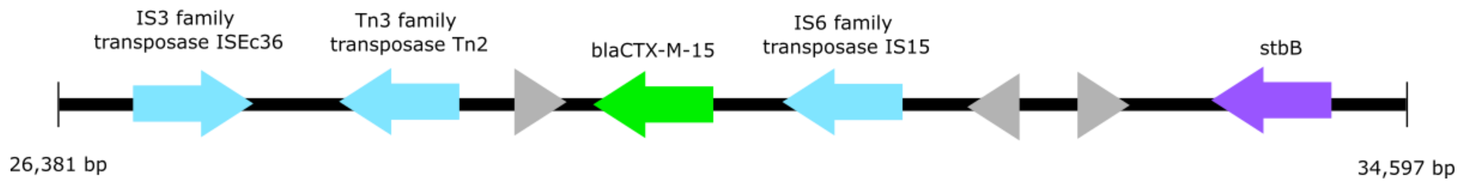

K.

Species: Shigella
Isolate: S17BD08233 (i55)
Structure: Plasmid (contig 2)

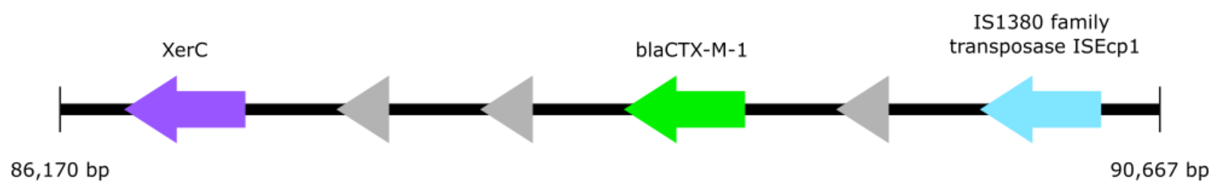

L.

Species: Shigella
Isolate: S18BD01106 (i59)
Structure: Plasmid (contig 3)

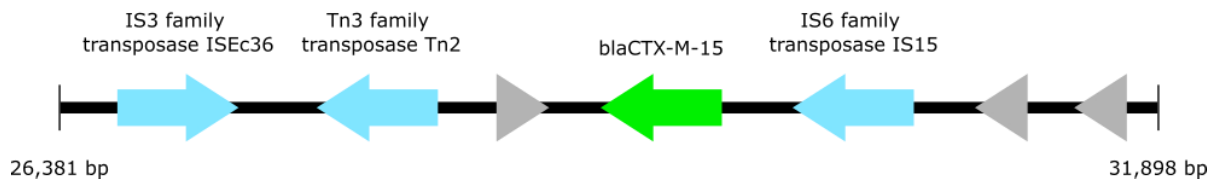

M.

Species: Shigella
Isolate: S18BD03178 (i67)
Structure: Plasmid (contig 3)

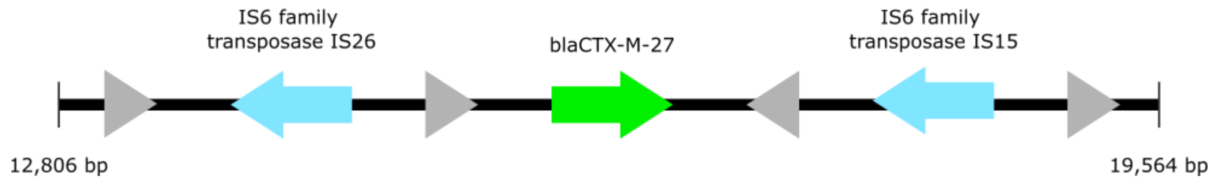

N.

Species: Shigella
Isolate: S18BD03411 (i69)
Structure: Plasmid (contig 2)

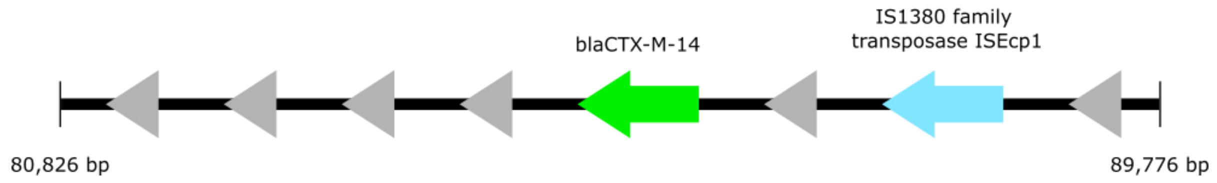

O.

Species: Shigella
Isolate: S18BD04295 (i73)
Structure: Plasmid (contig 9)

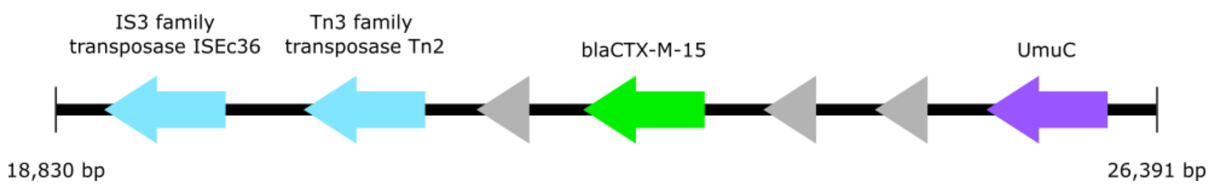

P.

Species: Shigella
Isolate: S18BD05612 (i83)
Structure: Plasmid (contig 3)

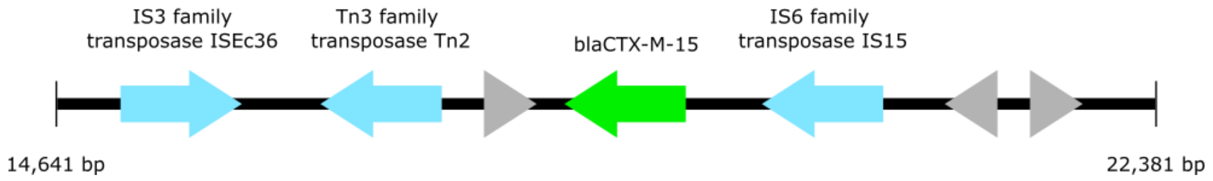

Figure S3: Context of the ESBL genes (green), transposases (blue), hypothetical genes (grey) and other genes (purple) around the ESBL gene of *Shigella* i3 (A), i4 (B), i5 (C), i9 (D), i12 (E), i20 (F), i31(G), i38 (H), i39 (I), i54 (J), i55 (K), i59 (L), i67 (M), i69 (N), i73 (O) and i83 (P) The direction of the arrow shows displays the orientation of the genes.

Table S8: transposases detected in short read assemblies

isolate	transposase count	transposase names

i1	0	-
i2	4	product ISNCY family transposase ISRor2<br>product Tn3 family transposase Tn2<br>product IS1380 family transposase ISEcp1<br>product IS6 family transposase IS15
i3	1	product IS1380 family transposase ISEcp1
i4	0	-
i5	5	product ISNCY family transposase ISRor2<br>product Tn3 family transposase Tn2<br>product IS3 family transposase ISEc36<br>product ISKra4 family transposase ISKpn19<br>product IS6 family transposase IS26
i6	0	-
i7	1	product ISNCY family transposase ISSen7
i8	0	-
i9	7	product IS5 family transposase ISKpn26<br>product IS1380 family transposase ISEcp1<br>product ISNCY family transposase ISSen7<br>product IS3 family transposase IS2<br>product IS110 family transposase IS5075<br>product IS6 family transposase IS26<br>product IS91 family transposase ISSbo1
i10	4	product IS1 family transposase IS1D<br>product Tn3 family transposase Tn3<br>product IS6 family transposase IS15<br>product Tn3 family transposase Tn2
i11	4	product IS1 family transposase IS1D<br>product Tn3 family transposase Tn3<br>product IS6 family transposase IS15<br>product Tn3 family transposase Tn2
i12	4	product Tn3 family transposase Tn2<br>product IS1380 family transposase ISEcp1

		product Tn3 family transposase Tn2<br>product ISNCY family transposase ISRor2
i13	0	-
i14	3	product IS1380 family transposase ISEcp1<br>product IS5 family transposase IS5<br>product ISNCY family transposase ISRor2
i15	5	product ISNCY family transposase ISRor2<br>product Tn3 family transposase Tn2<br>product IS3 family transposase ISEc36<br>product ISKra4 family transposase ISKpn19<br>product IS6 family transposase IS15
i16	5	product IS6 family transposase IS15<br>product ISKra4 family transposase ISKpn19<br>product IS3 family transposase ISEc36<br>product Tn3 family transposase Tn2<br>product ISNCY family transposase ISRor2
i17	1	product IS6 family transposase IS26
i18	4	product ISNCY family transposase ISRor2<br>product IS1182 family transposase ISCfr1<br>product IS6 family transposase IS26<br>product IS6 family transposase IS26
i19	2	product ISNCY family transposase ISRor2<br>product IS1380 family transposase ISEcp1
i20	2	product IS1380 family transposase ISEcp1<br>product ISNCY family transposase ISRor2
i21	2	product ISNCY family transposase ISRor2<br>product IS1380 family transposase ISEcp1
i22	4	product IS6 family transposase IS26<br>product IS6 family transposase IS26<br>product IS1182 family transposase ISCfr1<br>product ISNCY family transposase ISRor2
i23	5	product IS6 family transposase IS15<br>product ISKra4 family transposase ISKpn19

		product IS3 family transposase ISEc36<br>product Tn3 family transposase Tn2<br>product ISNCY family transposase ISRor2
i24	1	product ISNCY family transposase ISSen7
i25	5	product ISKra4 family transposase ISKpn19<br>product IS3 family transposase ISEc36<br>product Tn3 family transposase Tn2<br>product ISNCY family transposase ISRor2<br>product IS6 family transposase IS15
i26	0	-
i27	1	product IS1380 family transposase ISEcp1
i28	2	product IS1380 family transposase ISEcp1<br>product ISNCY family transposase ISRor2
i29	1	product IS1380 family transposase ISEcp1
i30	4	product ISNCY family transposase ISRor2<br>product IS1182 family transposase ISCfr1<br>product IS6 family transposase IS26<br>product IS6 family transposase IS26
i31	1	product ISNCY family transposase ISRor2
i32	1	product IS1380 family transposase ISEcp1
i33	0	-
i34	5	product IS6 family transposase IS26<br>product Tn3 family transposase Tn2<br>product IS6 family transposase IS26<br>product Tn3 family transposase Tn3<br>product IS1 family transposase IS1D
i35	4	product ISNCY family transposase ISRor2<br>product Tn3 family transposase Tn2<br>product IS3 family transposase ISEc36<br>product ISKra4 family transposase ISKpn19
i36	7	product IS110 family transposase ISEc45<br>product IS1380 family transposase ISEcp1

		product ISNCY family transposase ISSen7<br>product IS21 family transposase IS100kyp<br>product IS21 family transposase IS21<br>product IS66 family transposase ISCro1<br>product IS66 family transposase ISCro1
i37	0	-
i38	1	product IS1380 family transposase ISEcp1
i39	4	product ISNCY family transposase ISRor2<br>product IS6 family transposase IS15<br>product IS1380 family transposase ISEcp1<br>product Tn3 family transposase Tn2
i40	4	product ISNCY family transposase ISRor2<br>product Tn3 family transposase Tn2<br>product IS3 family transposase ISEc36<br>product ISKra4 family transposase ISKpn19
i41	4	product IS1380 family transposase ISEcp1<br>product Tn3 family transposase Tn2<br>product IS6 family transposase IS26<br>product IS3 family transposase IS3
i42	0	-
i43	3	product IS1182 family transposase ISCfr1<br>product IS6 family transposase IS26<br>product IS6 family transposase IS26
i44	0	-
i45	2	product IS91 family transposase ISSbo1<br>product IS1380 family transposase ISEcp1
i46	0	-
i47	3	product IS91 family transposase ISSbo1<br>product IS1380 family transposase ISEcp1<br>product ISNCY family transposase ISSen7
i48	3	product IS91 family transposase ISSbo1<br>product IS1380 family transposase ISEcp1

		product ISNCY family transposase ISSen7
i49	0	-
i50	0	-
i51	4	product IS6 family transposase IS6100<br>product IS6 family transposase IS15<br>product Tn3 family transposase Tn2<br>product IS6 family transposase IS15
i52	2	product IS1380 family transposase ISEcp1<br>product ISNCY family transposase ISSen7
i53	2	product IS1380 family transposase ISEcp1<br>product ISNCY family transposase ISSen7
i54	0	-
i55	2	product IS1380 family transposase ISEcp1<br>product IS91 family transposase ISSbo1
i56	1	product IS1380 family transposase ISEcp1
i57	0	-
i58	0	-
i59	0	-
i60	0	-
i61	0	-
i62	1	product ISNCY family transposase ISSen7
i63	3	product Tn3 family transposase Tn2<br>product IS3 family transposase ISEc36<br>product ISKra4 family transposase ISKpn19
i64	0	-
i65	0	-
i66	0	-
i67	0	-
i68	0	-
i69	2	product IS1380 family transposase ISEcp1<br>product ISNCY family transposase ISSen7

i70	0	-
i71	0	-
i72	0	-
i73	5	product ISNCY family transposase ISRor2<br>product Tn3 family transposase Tn2<br>product IS3 family transposase ISEc36<br>product ISKra4 family transposase ISKpn19<br>product IS6 family transposase IS15
i74	0	-
i75	0	-
i76	0	-
i77	0	-
i78	0	-
i79	0	-
i80	0	-
i81	7	product IS1 family transposase IS1D<br>product Tn3 family transposase Tn2<br>product IS91 family transposase IS91<br>product ISKra4 family transposase ISKpn19<br>product IS3 family transposase ISEc36<br>product Tn3 family transposase Tn2<br>product IS6 family transposase IS15
i82	1	product ISNCY family transposase ISSen7
i83	3	product Tn3 family transposase Tn2<br>product IS3 family transposase ISEc36<br>product ISKra4 family transposase ISKpn19
i84	0	-
i85	0	-
i86	4	product ISNCY family transposase ISRor2<br>product Tn3 family transposase Tn2<br>product IS3 family transposase ISEc36<br>product ISKra4 family transposase ISKpn19

i87	0	-
i88	4	product IS6 family transposase IS26<br>product IS6 family transposase IS26<br>product IS1182 family transposase ISCfr1<br>product ISNCY family transposase ISRor2
i89	0	-
i90	0	-
i91	1	product ISNCY family transposase ISRor2
i92	1	product ISNCY family transposase ISRor2
i93	5	product IS1 family transposase IS1D<br>product Tn3 family transposase Tn3<br>product IS6 family transposase IS26<br>product Tn3 family transposase Tn2<br>product IS6 family transposase IS26
i94	0	-
i95	1	product IS6 family transposase IS6100
i96	1	product ISNCY family transposase ISSen7
i97	0	-
i98	0	-
i99	3	product Tn3 family transposase Tn2<br>product IS3 family transposase ISEc36<br>product ISKra4 family transposase ISKpn19
i100	6	product IS110 family transposase IS5075<br>product ISKra4 family transposase ISKpn19<br>product IS3 family transposase ISEc36<br>product Tn3 family transposase Tn2<br>product IS1380 family transposase ISEcp1<br>product Tn3 family transposase Tn2
i101	2	product IS3 family transposase ISEc36<br>product Tn3 family transposase Tn2
i102	0	-
i103	0	-

i104	1	product IS1380 family transposase ISEcp1
i105	1	product IS1380 family transposase ISEcp1
i106	0	-
i107	0	-
i108	0	-
i109	0	-
i110	0	-
i111	1	product Tn3 family transposase IS3000
i112	13	product Tn3 family transposase Tn2<br>product Tn3 family transposase TnAs1<br>product IS91 family transposase IS91<br>product IS30 family transposase IS30<br>product IS66 family transposase ISEc8<br>product IS481 family transposase ISKpn28<br>product IS3 family transposase ISEc17<br>product IS3 family transposase ISEc17<br>product IS3 family transposase IS2<br>product ISNCY family transposase ISBcen27<br>product IS3 family transposase IS629<br>product IS3 family transposase IS629<br>product IS3 family transposase IS629
i113	4	product IS1380 family transposase ISEcp1<br>product ISNCY family transposase ISSen7<br>product IS110 family transposase IS5075<br>product IS6 family transposase IS26
i114	0	-
i115	3	product Tn3 family transposase Tn2<br>product IS1380 family transposase ISEcp1<br>product Tn3 family transposase Tn2
i116	1	product Tn3 family transposase Tn2
i117	5	product Tn3 family transposase Tn2<br>product IS1380 family transposase ISEcp1

		product Tn3 family transposase Tn2<br>product IS3 family transposase ISEc36<br>product ISKra4 family transposase ISKpn19
i118	1	product Tn3 family transposase IS3000
i119	4	product ISNCY family transposase ISRor2<br>product ISL3 family transposase ISKox3<br>product IS6 family transposase IS15<br>product IS5 family transposase IS903
i120	1	product ISNCY family transposase ISRor2
i121	1	product Tn3 family transposase IS3000
i122	1	product IS1380 family transposase ISEcp1
i123	1	product Tn3 family transposase IS3000
i124	0	-
i125	3	product ISNCY family transposase ISRor2<br>product IS200/IS605 family transposase ISEc46<br>product IS1380 family transposase ISEcp1
i126	3	product Tn3 family transposase Tn2<br>product IS4 family transposase IS421<br>product ISNCY family transposase ISRor2
i127	1	product IS5 family transposase IS903
i128	0	-
i129	1	product Tn3 family transposase IS3000
i130	1	product IS5 family transposase IS903
i131	1	product Tn3 family transposase IS3000

Table S9: *Shigella* pointfinder output

**isolate**	**chromosomal point mutations**
i1	parC p.S80I, gyrA p.S83L, gyrA p.D87G
i2	-
i3	-
i4	-

i5	gyrA p.S83L
i6	-
i7	-
i8	-
i9	-
i10	-
i11	-
i12	-
i13	gyrA p.D87Y
i14	gyrA p.S83L
i15	gyrA p.S83L, gyrA p.D87G, parC p.S80I
i16	gyrA p.S83L, gyrA p.D87G, parC p.S80I
i17	gyrA p.S83L
i18	gyrA p.S83L
i19	gyrA p.S83L, gyrA p.D87G, parC p.S80I
i20	gyrA p.S83L, gyrA p.D87G, parC p.S80I
i21	gyrA p.S83L, gyrA p.D87G, parC p.S80I
i22	gyrA p.S83L
i23	parC p.S80I, gyrA p.S83L, gyrA p.D87G
i24	-
i25	gyrA p.S83L
i26	gyrA p.D87Y
i27	gyrA p.S83L, gyrA p.D87G, parC p.S80I
i28	parC p.S80I, gyrA p.S83L, gyrA p.D87G
i29	gyrA p.S83L
i30	-
i31	-
i32	parC p.E84K, gyrA p.S83L, gyrA p.D87Y

i33	-
i34	-
i35	-
i36	gyrA p.S83L, gyrA p.D87G, parC p.S80I
i37	-
i38	-
i39	-
i40	gyrA p.S83L
i41	parC p.S80I, gyrA p.S83L, gyrA p.D87G
i42	-
i43	gyrA p.S83L
i44	-
i45	-
i46	-
i47	-
i48	-
i49	gyrA p.D87Y
i50	gyrA p.D87Y
i51	-
i52	-
i53	-
i54	gyrA p.D87Y
i55	-
i56	-
i57	gyrA p.D87Y
i58	gyrA p.D87Y
i59	gyrA p.D87Y
i60	gyrA p.D87Y

i61	-
i62	-
i63	parC p.S80I, gyrA p.S83L, gyrA p.D87G
i64	gyrA p.D87Y
i65	gyrA p.D87Y
i66	-
i67	gyrA p.S83L
i68	gyrA p.S83L
i69	gyrA p.S83L
i70	gyrA p.S83L
i71	gyrA p.S83L
i72	gyrA p.S83L
i73	-
i74	gyrA p.S83L
i75	gyrA p.S83L
i76	gyrA p.S83L
i77	gyrA p.S83L
i78	gyrA p.S83L
i79	gyrA p.S83L
i80	gyrA p.S83L
i81	gyrA p.S83L, gyrA p.D87G, parC p.S80I
i82	-
i83	-
i84	-
i85	-
i86	-
i87	-
i88	-

i89	gyrA p.S83L
i90	gyrA p.D87Y
i91	-
i92	-
i93	-
i94	-
i95	-
i96	-
i97	-
i98	gyrA p.S83L
i99	parC p.S80I, gyrA p.S83L, gyrA p.D87G
i100	-

A.

```
Query: NODE_1+_length_4836985_cov_1 Query ID: lcl|Query_29001 Length: 17001

>NODE_1+_length_4863168_cov_1
Sequence ID: Query_29003 Length: 4863168
Range 1: 4393427 to 4395942

Score:4636 bits(2510), Expect:0.0,
Identities:2514/2516(99%),  Gaps:0/2516(0%), Strand: Plus/Plus

Query  2090815  GAATAAAACCTGGGACCTACGTGCGCCCGCACCGACACCCTCACACCTTCGAGCTACTGT  2090874
                |||||  |||||||||||||||||||||||||||||||||||||||||||||||||||||
Sbjct  4393427  GAATATGACCTGGGACCTACGTGCGCCCGCACCGACACCCTCACACCTTCGAGCTACTGT  4393486

Query  2090875  TGCCATTAAGGGGTCGTTTCGTGGTGCTGAATTTTGACGATCGGGGTACCGTCACCCATC  2090934
                ||||||||||||||||||||||||||||||||||||||||||||||||||||||||||||
Sbjct  4393487  TGCCATTAAGGGGTCGTTTCGTGGTGCTGAATTTTGACGATCGGGGTACCGTCACCCATC  4393546

Query  2090935  GGGCGATATTGGGGGAAACCTGTACGGTGCTGGAGATGGCCGCAGGAACCTGGCATGCCG  2090994
```

```
                 ||||||||||||||||||||||||||||||||||||||||||||||||||||||||||||
Sbjct  4393547  GGGCGATATTGGGGGAAACCTGTACGGTGCTGGAGATGGCCGCAGGAACCTGGCATGCCG  4393606

Query  2090995  TGCTGTCGCTGGATACCGGTGGCATAATTTTTGAAGTAAAACACGGTGGCTATCAACCCG  2091054
                ||||||||||||||||||||||||||||||||||||||||||||||||||||||||||||
Sbjct  4393607  TGCTGTCGCTGGATACCGGTGGCATAATTTTTGAAGTAAAACACGGTGGCTATCAACCCG  4393666

Query  2091055  TGGCTGCCGATGACTATGCGCACTGGGCTCCAGCGGAAGGAGAACCAGGAACCACGGAGC  2091114
                ||||||||||||||||||||||||||||||||||||||||||||||||||||||||||||
Sbjct  4393667  TGGCTGCCGATGACTATGCGCACTGGGCTCCAGCGGAAGGAGAACCAGGAACCACGGAGC  4393726

Query  2091115  TTATGGCCTGGTATGCGCAAGCGCAGGTGGGCGACAGCACTTTTGCCGTCTAAGGCGATA  2091174
                ||||||||||||||||||||||||||||||||||||||||||||||||||||||||||||
Sbjct  4393727  TTATGGCCTGGTATGCGCAAGCGCAGGTGGGCGACAGCACTTTTGCCGTCTAAGGCGATA  4393786

Query  2091175  AACAAAAACGGAATGAGTTTCCCCATTCCGTTTCCGCTATTACAAACCGTCGGTGACGAT  2091234
                ||||||||||||||||||||||||||||||||||||||||||||||||||||||||||||
Sbjct  4393787  AACAAAAACGGAATGAGTTTCCCCATTCCGTTTCCGCTATTACAAACCGTCGGTGACGAT  4393846

Query  2091235  TTTAGCCGCCGACGCTAATACATCGCGACGGCTTTCTGCCTTAGGTTGAGGCTGGGTGAA  2091294
                ||||||||||||||||||||||||||||||||||||||||||||||||||||||||||||
Sbjct  4393847  TTTAGCCGCCGACGCTAATACATCGCGACGGCTTTCTGCCTTAGGTTGAGGCTGGGTGAA  4393906

Query  2091295  GTAAGTGACCAGAATCAGCGGCGCACGATCTTTTGGCCAGATCACCGCGATATCGTTGGT  2091354
                ||||||||||||||||||||||||||||||||||||||||||||||||||||||||||||
Sbjct  4393907  GTAAGTGACCAGAATCAGCGGCGCACGATCTTTTGGCCAGATCACCGCGATATCGTTGGT  4393966

Query  2091355  GGTGCCATAGCCACCGCTGCCGGTTTTATCCCCCACAACCCAGGAAGCAGGCAGTCCAGC  2091414
                ||||||||||||||||||||||||||||||||||||||||||||||||||||||||||||
Sbjct  4393967  GGTGCCATAGCCACCGCTGCCGGTTTTATCCCCCACAACCCAGGAAGCAGGCAGTCCAGC  4394026

Query  2091415  CTGAATGCTCGCTGCACCGGTGGTATTGCCTTTCATCCATGTCACCAGCTGCGCCCGTTG  2091474
                ||||||||||||||||||||||||||||||||||||||||||||||||||||||||||||
Sbjct  4394027  CTGAATGCTCGCTGCACCGGTGGTATTGCCTTTCATCCATGTCACCAGCTGCGCCCGTTG  4394086
```

```
Query  2091475  GCTGTCGCCCAATGCTTTACCCAGCGTCAGATTCCGCAGAGTTTGCGCCATTGCCCGAGG  2091534
                ||||||||||||||||||||||||||||||||||||||||||||||||||||||||||||
Sbjct  4394087  GCTGTCGCCCAATGCTTTACCCAGCGTCAGATTCCGCAGAGTTTGCGCCATTGCCCGAGG  4394146

Query  2091535  TGAAGTGGTATCACGCGGATCGCCCGGAATGGCGGTGTTTAACGTCGGCTCGGTACGGTC  2091594
                ||||||||||||||||||||||||||||||||||||||||||||||||||||||||||||
Sbjct  4394147  TGAAGTGGTATCACGCGGATCGCCCGGAATGGCGGTGTTTAACGTCGGCTCGGTACGGTC  4394206

Query  2091595  GAGACGGAACGTTTCGTCTCCCAGCTGTCGGGCGAACGCGGTGACGCTAGCCGGGCCGCC  2091654
                ||||||||||||||||||||||||||||||||||||||||||||||||||||||||||||
Sbjct  4394207  GAGACGGAACGTTTCGTCTCCCAGCTGTCGGGCGAACGCGGTGACGCTAGCCGGGCCGCC  4394266

Query  2091655  AACGTGAGCAATCAGCTTATTCATCGCCACGTTATCGCTGTACTGTAGCGCGGCCGCGCT  2091714
                ||||||||||||||||||||||||||||||||||||||||||||||||||||||||||||
Sbjct  4394267  AACGTGAGCAATCAGCTTATTCATCGCCACGTTATCGCTGTACTGTAGCGCGGCCGCGCT  4394326

Query  2091715  AAGCTCAGCCAGTGACATCGTCCCATTGACGTGCTTTTCCGCAATCGGATTATAGTTAAC  2091774
                ||||||||||||||||||||||||||||||||||||||||||||||||||||||||||||
Sbjct  4394327  AAGCTCAGCCAGTGACATCGTCCCATTGACGTGCTTTTCCGCAATCGGATTATAGTTAAC  4394386

Query  2091775  AAGGTCAGATTTTTTGATCTCAACTCGCTGATTTAACAGATTCGGTTCGCTTTCACTTTT  2091834
                ||||||||||||||||||||||||||||||||||||||||||||||||||||||||||||
Sbjct  4394387  AAGGTCAGATTTTTTGATCTCAACTCGCTGATTTAACAGATTCGGTTCGCTTTCACTTTT  4394446

Query  2091835  CTTCAGCACCGCGGCCGCGGCCATCACTTTACTGGTGCTGCACATCGCAAAGCGCTCATC  2091894
                ||||||||||||||||||||||||||||||||||||||||||||||||||||||||||||
Sbjct  4394447  CTTCAGCACCGCGGCCGCGGCCATCACTTTACTGGTGCTGCACATCGCAAAGCGCTCATC  4394506

Query  2091895  AGCACGATAAAGTATTTGCGAATTATCTGCTGTGTTAATCAATGCCACACCCAGTCTGCC  2091954
                ||||||||||||||||||||||||||||||||||||||||||||||||||||||||||||
Sbjct  4394507  AGCACGATAAAGTATTTGCGAATTATCTGCTGTGTTAATCAATGCCACACCCAGTCTGCC  4394566

Query  2091955  TCCCGACTGCCGCTCTAATTCGGCAAGTTTTTGCTGTACGTCCGCCGTTTGCGCATACAG  2092014
```

```
                 ||||||||||||||||||||||||||||||||||||||||||||||||||||||||||||
Sbjct  4394567  TCCCGACTGCCGCTCTAATTCGGCAAGTTTTTGCTGTACGTCCGCCGTTTGCGCATACAG  4394626

Query  2092015  CGGCACACTTCCTAACAACAGCGTGACGGTTGCCGTCGCCATCAGCGTGAACTGGCGCAG  2092074
                ||||||||||||||||||||||||||||||||||||||||||||||||||||||||||||
Sbjct  4394627  CGGCACACTTCCTAACAACAGCGTGACGGTTGCCGTCGCCATCAGCGTGAACTGGCGCAG  4394686

Query  2092075  TGATTTTTTAACCATGGGATTCCTTATTCTGGAAGATACGAAATAACAACAACATGAATA  2092134
                ||||||||||||||||||||||||||||||||||||||||||||||||||||||||||||
Sbjct  4394687  TGATTTTTTAACCATGGGATTCCTTATTCTGGAAGATACGAAATAACAACAACATGAATA  4394746

Query  2092135  GTCCCTAAATTCCACGTGTGTTTTTTATTAGCTTCAAAAATCACTATTTCACGAAGAATT  2092194
                ||||||||||||||||||||||||||||||||||||||||||||||||||||||||||||
Sbjct  4394747  GTCCCTAAATTCCACGTGTGTTTTTTATTAGCTTCAAAAATCACTATTTCACGAAGAATT  4394806

Query  2092195  TAGACTGCTTCTCACACATTGTAACATTATTTACAACCACCTTTCAATCATTTTTGATAA  2092254
                ||||||||||||||||||||||||||||||||||||||||||||||||||||||||||||
Sbjct  4394807  TAGACTGCTTCTCACACATTGTAACATTATTTACAACCACCTTTCAATCATTTTTGATAA  4394866

Query  2092255  ATCATTGATTTCATCTTTGCTGCAATGATACTTAATAAACTCTGCAAGTTATCCACAGAG  2092314
                ||||||||||||||||||||||||||||||||||||||||||||||||||||||||||||
Sbjct  4394867  ATCATTGATTTCATCTTTGCTGCAATGATACTTAATAAACTCTGCAAGTTATCCACAGAG  4394926

Query  2092315  CAACACTCAATTTTATTGATGATATTCTTATTATACCAGACATTTTTCATACACTCCCTT  2092374
                ||||||||||||||||||||||||||||||||||||||||||||||||||||||||||||
Sbjct  4394927  CAACACTCAATTTTATTGATGATATTCTTATTATACCAGACATTTTTCATACACTCCCTT  4394986

Query  2092375  GTACGGATAGTTTTCCGACAACTTCATGATTACATATCTTGCGGTTTTGATTATTTTTGC  2092434
                ||||||||||||||||||||||||||||||||||||||||||||||||||||||||||||
Sbjct  4394987  GTACGGATAGTTTTCCGACAACTTCATGATTACATATCTTGCGGTTTTGATTATTTTTGC  4395046

Query  2092435  TGCAAGAAATACATACTTCAAACGAAAGGTCTTTATTTGCTGTCTGTATTCTGAAGAGTC  2092494
                ||||||||||||||||||||||||||||||||||||||||||||||||||||||||||||
Sbjct  4395047  TGCAAGAAATACATACTTCAAACGAAAGGTCTTTATTTGCTGTCTGTATTCTGAAGAGTC  4395106
```

```
Query  2092495  CAAGGAATCAAACTTGAACAACAAAAATAGGTTATATGAAAGCATCATCATTTGAAACAC  2092554
                ||||||||||||||||||||||||||||||||||||||||||||||||||||||||||||
Sbjct  4395107  CAAGGAATCAAACTTGAACAACAAAAATAGGTTATATGAAAGCATCATCATTTGAAACAC  4395166

Query  2092555  GGCTTCATTCGCCCAAAATGACTTTAGCAAGAGATGACCCACCGCCATGTCGTATTTGGC  2092614
                ||||||||||||||||||||||||||||||||||||||||||||||||||||||||||||
Sbjct  4395167  GGCTTCATTCGCCCAAAATGACTTTAGCAAGAGATGACCCACCGCCATGTCGTATTTGGC  4395226

Query  2092615  TTCTTTGATATAGTTTTCAGCATTACCACGCTTTTCATAGTATATAACTACTTTTTCAGA  2092674
                ||||||||||||||||||||||||||||||||||||||||||||||||||||||||||||
Sbjct  4395227  TTCTTTGATATAGTTTTCAGCATTACCACGCTTTTCATAGTATATAACTACTTTTTCAGA  4395286

Query  2092675  AAGCAAGGTAGTATTTGTTACAAAGAAAAAGTAGTCGTATTCGGAACCTTCTAAAAGTGA  2092734
                ||||||||||||||||||||||||||||||||||||||||||||||||||||||||||||
Sbjct  4395287  AAGCAAGGTAGTATTTGTTACAAAGAAAAAGTAGTCGTATTCGGAACCTTCTAAAAGTGA  4395346

Query  2092735  TAATTGTGCTCTTTCTTTTTCTGGTTTCAGTACGCGAGATACGACAAATCTTCTGTCTTT  2092794
                ||||||||||||||||||||||||||||||||||||||||||||||||||||||||||||
Sbjct  4395347  TAATTGTGCTCTTTCTTTTTCTGGTTTCAGTACGCGAGATACGACAAATCTTCTGTCTTT  4395406

Query  2092795  TTCCCATTTAACTAATTTTGTATACAGTTCTGTAGTTTCTCTACCTTCTTCTCCTTTAAC  2092854
                ||||||||||||||||||||||||||||||||||||||||||||||||||||||||||||
Sbjct  4395407  TTCCCATTTAACTAATTTTGTATACAGTTCTGTAGTTTCTCTACCTTCTTCTCCTTTAAC  4395466

Query  2092855  GAATACAATTGATGAATTCGTTGCTTGTGAGGTGAGTGTAGAATAACTTTTGGCTTTAAT  2092914
                ||||||||||||||||||||||||||||||||||||||||||||||||||||||||||||
Sbjct  4395467  GAATACAATTGATGAATTCGTTGCTTGTGAGGTGAGTGTAGAATAACTTTTGGCTTTAAT  4395526

Query  2092915  TAAATATTTGCATCCAAGAGATTCTATCGTTTCGATAATTTTTTCATCAAAGTAGCCACT  2092974
                ||||||||||||||||||||||||||||||||||||||||||||||||||||||||||||
Sbjct  4395527  TAAATATTTGCATCCAAGAGATTCTATCGTTTCGATAATTTTTTCATCAAAGTAGCCACT  4395586

Query  2092975  ATCCATTCGAAATAAAATTTCTAAATCGTCTGATTTGATGTTAGCAACAATTTCTTTGAT  2093034
```

```
                  ||||||||||||||||||||||||||||||||||||||||||||||||||||||||||||
Sbjct  4395587  ATCCATTCGAAATAAAATTTCTAAATCGTCTGATTTGATGTTAGCAACAATTTCTTTGAT  4395646

Query  2093035  CATTTCCGCAGCACCGTTTGCAGTGTAAGTATTGCCACTTCTTACAAATCCGGTAACATA  2093094
                ||||||||||||||||||||||||||||||||||||||||||||||||||||||||||||
Sbjct  4395647  CATTTCCGCAGCACCGTTTGCAGTGTAAGTATTGCCACTTCTTACAAATCCGGTAACATA  4395706

Query  2093095  TGCTTTTAATTCGTCGCAAAATGCAAATTGGATATTGTAGCATCGGTTTCCCAGTTTCTT  2093154
                ||||||||||||||||||||||||||||||||||||||||||||||||||||||||||||
Sbjct  4395707  TGCTTTTAATTCGTCGCAAAATGCAAATTGGATATTGTAGCATCGGTTTCCCAGTTTCTT  4395766

Query  2093155  AGGATTATATCCTTTTGACGCACCTTCTTGATGACCTTCTACGTTAATTACACTACTATC  2093214
                ||||||||||||||||||||||||||||||||||||||||||||||||||||||||||||
Sbjct  4395767  AGGATTATATCCTTTTGACGCACCTTCTTGATGACCTTCTACGTTAATTACACTACTATC  4395826

Query  2093215  AATATCAATCGTAATGGATGTCAATTTACTTTTAGTGAGCAGTTTTTTAAAGACTTTAAA  2093274
                ||||||||||||||||||||||||||||||||||||||||||||||||||||||||||||
Sbjct  4395827  AATATCAATCGTAATGGATGTCAATTTACTTTTAGTGAGCAGTTTTTTAAAGACTTTAAA  4395886

Query  2093275  ATTAATGTCTCTAAACATTTGGGTTGTCTTGAAGTTGAAGTTTCCTAGAAACCGTG  2093330
                ||||||||||||||||||||||||||||||||||||||||||||||||||||||||
Sbjct  4395887  ATTAATGTCTCTAAACATTTGGGTTGTCTTGAAGTTGAAGTTTCCTAGAAACCGTG  4395942
```

B.

```
Query: NODE_1+_length_4836985_cov_1 Query ID: lcl|Query_34321 Length: 4836985

>ISEcp1
Sequence ID: Query_34323 Length: 1656
Range 1: 1 to 1656

Score:3059 bits(1656), Expect:0.0,
Identities:1656/1656(100%),  Gaps:0/1656(0%), Strand: Plus/Minus
```

```
Query  2092138  CCTAAATTCCACGTGTGTTTTTTATTAGCTTCAAAAATCACTATTTCACGAAGAATTTAG  2092197
                ||||||||||||||||||||||||||||||||||||||||||||||||||||||||||||
Sbjct  1656     CCTAAATTCCACGTGTGTTTTTTATTAGCTTCAAAAATCACTATTTCACGAAGAATTTAG  1597

Query  2092198  ACTGCTTCTCACACATTGTAACATTATTTACAACCACCTTTCAATCATTTTTGATAAATC  2092257
                ||||||||||||||||||||||||||||||||||||||||||||||||||||||||||||
Sbjct  1596     ACTGCTTCTCACACATTGTAACATTATTTACAACCACCTTTCAATCATTTTTGATAAATC  1537

Query  2092258  ATTGATTTCATCTTTGCTGCAATGATACTTAATAAACTCTGCAAGTTATCCACAGAGCAA  2092317
                ||||||||||||||||||||||||||||||||||||||||||||||||||||||||||||
Sbjct  1536     ATTGATTTCATCTTTGCTGCAATGATACTTAATAAACTCTGCAAGTTATCCACAGAGCAA  1477

Query  2092318  CACTCAATTTTATTGATGATATTCTTATTATACCAGACATTTTTCATACACTCCCTTGTA  2092377
                ||||||||||||||||||||||||||||||||||||||||||||||||||||||||||||
Sbjct  1476     CACTCAATTTTATTGATGATATTCTTATTATACCAGACATTTTTCATACACTCCCTTGTA  1417

Query  2092378  CGGATAGTTTTCCGACAACTTCATGATTACATATCTTGCGGTTTTGATTATTTTTGCTGC  2092437
                ||||||||||||||||||||||||||||||||||||||||||||||||||||||||||||
Sbjct  1416     CGGATAGTTTTCCGACAACTTCATGATTACATATCTTGCGGTTTTGATTATTTTTGCTGC  1357

Query  2092438  AAGAAATACATACTTCAAACGAAAGGTCTTTATTTGCTGTCTGTATTCTGAAGAGTCCAA  2092497
                ||||||||||||||||||||||||||||||||||||||||||||||||||||||||||||
Sbjct  1356     AAGAAATACATACTTCAAACGAAAGGTCTTTATTTGCTGTCTGTATTCTGAAGAGTCCAA  1297

Query  2092498  GGAATCAAACTTGAACAACAAAAATAGGTTATATGAAAGCATCATCATTTGAAACACGGC  2092557
                ||||||||||||||||||||||||||||||||||||||||||||||||||||||||||||
Sbjct  1296     GGAATCAAACTTGAACAACAAAAATAGGTTATATGAAAGCATCATCATTTGAAACACGGC  1237

Query  2092558  TTCATTCGCCCAAAATGACTTTAGCAAGAGATGACCCACCGCCATGTCGTATTTGGCTTC  2092617
                ||||||||||||||||||||||||||||||||||||||||||||||||||||||||||||
Sbjct  1236     TTCATTCGCCCAAAATGACTTTAGCAAGAGATGACCCACCGCCATGTCGTATTTGGCTTC  1177

Query  2092618  TTTGATATAGTTTTCAGCATTACCACGCTTTTCATAGTATATAACTACTTTTTCAGAAAG  2092677
                ||||||||||||||||||||||||||||||||||||||||||||||||||||||||||||
```

```
Sbjct  1176     TTTGATATAGTTTTCAGCATTACCACGCTTTTCATAGTATATAACTACTTTTTCAGAAAG  1117

Query  2092678  CAAGGTAGTATTTGTTACAAAGAAAAAGTAGTCGTATTCGGAACCTTCTAAAAGTGATAA  2092737
                ||||||||||||||||||||||||||||||||||||||||||||||||||||||||||||
Sbjct  1116     CAAGGTAGTATTTGTTACAAAGAAAAAGTAGTCGTATTCGGAACCTTCTAAAAGTGATAA  1057

Query  2092738  TTGTGCTCTTTCTTTTTCTGGTTTCAGTACGCGAGATACGACAAATCTTCTGTCTTTTTC  2092797
                ||||||||||||||||||||||||||||||||||||||||||||||||||||||||||||
Sbjct  1056     TTGTGCTCTTTCTTTTTCTGGTTTCAGTACGCGAGATACGACAAATCTTCTGTCTTTTTC  997

Query  2092798  CCATTTAACTAATTTTGTATACAGTTCTGTAGTTTCTCTACCTTCTTCTCCTTTAACGAA  2092857
                ||||||||||||||||||||||||||||||||||||||||||||||||||||||||||||
Sbjct  996      CCATTTAACTAATTTTGTATACAGTTCTGTAGTTTCTCTACCTTCTTCTCCTTTAACGAA  937

Query  2092858  TACAATTGATGAATTCGTTGCTTGTGAGGTGAGTGTAGAATAACTTTTGGCTTTAATTAA  2092917
                ||||||||||||||||||||||||||||||||||||||||||||||||||||||||||||
Sbjct  936      TACAATTGATGAATTCGTTGCTTGTGAGGTGAGTGTAGAATAACTTTTGGCTTTAATTAA  877

Query  2092918  ATATTTGCATCCAAGAGATTCTATCGTTTCGATAATTTTTTCATCAAAGTAGCCACTATC  2092977
                ||||||||||||||||||||||||||||||||||||||||||||||||||||||||||||
Sbjct  876      ATATTTGCATCCAAGAGATTCTATCGTTTCGATAATTTTTTCATCAAAGTAGCCACTATC  817

Query  2092978  CATTCGAAATAAAATTTCTAAATCGTCTGATTTGATGTTAGCAACAATTTCTTTGATCAT  2093037
                ||||||||||||||||||||||||||||||||||||||||||||||||||||||||||||
Sbjct  816      CATTCGAAATAAAATTTCTAAATCGTCTGATTTGATGTTAGCAACAATTTCTTTGATCAT  757

Query  2093038  TTCCGCAGCACCGTTTGCAGTGTAAGTATTGCCACTTCTTACAAATCCGGTAACATATGC  2093097
                ||||||||||||||||||||||||||||||||||||||||||||||||||||||||||||
Sbjct  756      TTCCGCAGCACCGTTTGCAGTGTAAGTATTGCCACTTCTTACAAATCCGGTAACATATGC  697

Query  2093098  TTTTAATTCGTCGCAAAATGCAAATTGGATATTGTAGCATCGGTTTCCCAGTTTCTTAGG  2093157
                ||||||||||||||||||||||||||||||||||||||||||||||||||||||||||||
Sbjct  696      TTTTAATTCGTCGCAAAATGCAAATTGGATATTGTAGCATCGGTTTCCCAGTTTCTTAGG  637
```

```
Query  2093158  ATTATATCCTTTTGACGCACCTTCTTGATGACCTTCTACGTTAATTACACTACTATCAAT  2093217
                ||||||||||||||||||||||||||||||||||||||||||||||||||||||||||||
Sbjct  636      ATTATATCCTTTTGACGCACCTTCTTGATGACCTTCTACGTTAATTACACTACTATCAAT  577

Query  2093218  ATCAATCGTAATGGATGTCAATTTACTTTTAGTGAGCAGTTTTTTAAAGACTTTAAAATT  2093277
                ||||||||||||||||||||||||||||||||||||||||||||||||||||||||||||
Sbjct  576      ATCAATCGTAATGGATGTCAATTTACTTTTAGTGAGCAGTTTTTTAAAGACTTTAAAATT  517

Query  2093278  AATGTCTCTAAACATTTGGGTTGTCTTGAAGTTGAAGTTTCCTAGAAACCGTGACACTGT  2093337
                ||||||||||||||||||||||||||||||||||||||||||||||||||||||||||||
Sbjct  516      AATGTCTCTAAACATTTGGGTTGTCTTGAAGTTGAAGTTTCCTAGAAACCGTGACACTGT  457

Query  2093338  TTCAGGTTCTTTTACGGAAATATCAAACTCGTTGACGAGGGGATCATTTTGAAGTAGCTT  2093397
                ||||||||||||||||||||||||||||||||||||||||||||||||||||||||||||
Sbjct  456      TTCAGGTTCTTTTACGGAAATATCAAACTCGTTGACGAGGGGATCATTTTGAAGTAGCTT  397

Query  2093398  TAGACGTTCTAACTTATCAATGCCAATGAAGTGACCGCAGAGCATGGTCTTTATATGATT  2093457
                ||||||||||||||||||||||||||||||||||||||||||||||||||||||||||||
Sbjct  396      TAGACGTTCTAACTTATCAATGCCAATGAAGTGACCGCAGAGCATGGTCTTTATATGATT  337

Query  2093458  CATCTTGATTTTATTTGTTGAGTCATTATCAAATACGAGGTCATTTTCAATAAAATCAAA  2093517
                ||||||||||||||||||||||||||||||||||||||||||||||||||||||||||||
Sbjct  336      CATCTTGATTTTATTTGTTGAGTCATTATCAAATACGAGGTCATTTTCAATAAAATCAAA  277

Query  2093518  AATCCCATTGCTTTTTGCATTCTCAAGGAGCAGAAAAAGACCTGCATTTGATGTTAGATT  2093577
                ||||||||||||||||||||||||||||||||||||||||||||||||||||||||||||
Sbjct  276      AATCCCATTGCTTTTTGCATTCTCAAGGAGCAGAAAAAGACCTGCATTTGATGTTAGATT  217

Query  2093578  CTTAGCTTTGAAATCAATTTTATTAATCATAATTAGAACCCCTTTTTACTACTTTTCTTA  2093637
                ||||||||||||||||||||||||||||||||||||||||||||||||||||||||||||
Sbjct  216      CTTAGCTTTGAAATCAATTTTATTAATCATAATTAGAACCCCTTTTTACTACTTTTCTTA  157

Query  2093638  CTATTATTTTACCATATATCGAGTCATAAAAGCTGATAATTTAACATATTTTTGAGCACT  2093697
                ||||||||||||||||||||||||||||||||||||||||||||||||||||||||||||
```

```
Sbjct  156      CTATTATTTTACCATATATCGAGTCATAAAAGCTGATAATTTAACATATTTTTGAGCACT  97

Query  2093698  TTTCTTTCACCCAATGGGTGAAAGCTGAATTTCGAAGGAATGCATATTTATCAAGGCTTT  2093757
                ||||||||||||||||||||||||||||||||||||||||||||||||||||||||||||
Sbjct  96       TTTCTTTCACCCAATGGGTGAAAGCTGAATTTCGAAGGAATGCATATTTATCAAGGCTTT  37

Query  2093758  GATTATGCTTTTTGAAGTACTGACGTAGAATCTAGG  2093793
                ||||||||||||||||||||||||||||||||||||
Sbjct  36       GATTATGCTTTTTGAAGTACTGACGTAGAATCTAGG  1
```

C.

```
Query: NODE_1+_length_4863168_cov_1 Query ID: lcl|Query_83353 Length: 4863168

>ISEcp1
Sequence ID: Query_83355 Length: 1656
Range 1: 464 to 1656

Score:2204 bits(1193), Expect:0.0,
Identities:1193/1193(100%),  Gaps:0/1193(0%), Strand: Plus/Minus

Query  4394750  CCTAAATTCCACGTGTGTTTTTTATTAGCTTCAAAAATCACTATTTCACGAAGAATTTAG  4394809
                ||||||||||||||||||||||||||||||||||||||||||||||||||||||||||||
Sbjct  1656     CCTAAATTCCACGTGTGTTTTTTATTAGCTTCAAAAATCACTATTTCACGAAGAATTTAG  1597

Query  4394810  ACTGCTTCTCACACATTGTAACATTATTTACAACCACCTTTCAATCATTTTTGATAAATC  4394869
                ||||||||||||||||||||||||||||||||||||||||||||||||||||||||||||
Sbjct  1596     ACTGCTTCTCACACATTGTAACATTATTTACAACCACCTTTCAATCATTTTTGATAAATC  1537

Query  4394870  ATTGATTTCATCTTTGCTGCAATGATACTTAATAAACTCTGCAAGTTATCCACAGAGCAA  4394929
                ||||||||||||||||||||||||||||||||||||||||||||||||||||||||||||
Sbjct  1536     ATTGATTTCATCTTTGCTGCAATGATACTTAATAAACTCTGCAAGTTATCCACAGAGCAA  1477

Query  4394930  CACTCAATTTTATTGATGATATTCTTATTATACCAGACATTTTTCATACACTCCCTTGTA  4394989
```

```
               ||||||||||||||||||||||||||||||||||||||||||||||||||||||||||||
Sbjct  1476     CACTCAATTTTATTGATGATATTCTTATTATACCAGACATTTTTCATACACTCCCTTGTA  1417

Query  4394990  CGGATAGTTTTCCGACAACTTCATGATTACATATCTTGCGGTTTTGATTATTTTTGCTGC  4395049
               ||||||||||||||||||||||||||||||||||||||||||||||||||||||||||||
Sbjct  1416     CGGATAGTTTTCCGACAACTTCATGATTACATATCTTGCGGTTTTGATTATTTTTGCTGC  1357

Query  4395050  AAGAAATACATACTTCAAACGAAAGGTCTTTATTTGCTGTCTGTATTCTGAAGAGTCCAA  4395109
               ||||||||||||||||||||||||||||||||||||||||||||||||||||||||||||
Sbjct  1356     AAGAAATACATACTTCAAACGAAAGGTCTTTATTTGCTGTCTGTATTCTGAAGAGTCCAA  1297

Query  4395110  GGAATCAAACTTGAACAACAAAAATAGGTTATATGAAAGCATCATCATTTGAAACACGGC  4395169
               ||||||||||||||||||||||||||||||||||||||||||||||||||||||||||||
Sbjct  1296     GGAATCAAACTTGAACAACAAAAATAGGTTATATGAAAGCATCATCATTTGAAACACGGC  1237

Query  4395170  TTCATTCGCCCAAAATGACTTTAGCAAGAGATGACCCACCGCCATGTCGTATTTGGCTTC  4395229
               ||||||||||||||||||||||||||||||||||||||||||||||||||||||||||||
Sbjct  1236     TTCATTCGCCCAAAATGACTTTAGCAAGAGATGACCCACCGCCATGTCGTATTTGGCTTC  1177

Query  4395230  TTTGATATAGTTTTCAGCATTACCACGCTTTTCATAGTATATAACTACTTTTTCAGAAAG  4395289
               ||||||||||||||||||||||||||||||||||||||||||||||||||||||||||||
Sbjct  1176     TTTGATATAGTTTTCAGCATTACCACGCTTTTCATAGTATATAACTACTTTTTCAGAAAG  1117

Query  4395290  CAAGGTAGTATTTGTTACAAAGAAAAAGTAGTCGTATTCGGAACCTTCTAAAAGTGATAA  4395349
               ||||||||||||||||||||||||||||||||||||||||||||||||||||||||||||
Sbjct  1116     CAAGGTAGTATTTGTTACAAAGAAAAAGTAGTCGTATTCGGAACCTTCTAAAAGTGATAA  1057

Query  4395350  TTGTGCTCTTTCTTTTTCTGGTTTCAGTACGCGAGATACGACAAATCTTCTGTCTTTTTC  4395409
               ||||||||||||||||||||||||||||||||||||||||||||||||||||||||||||
Sbjct  1056     TTGTGCTCTTTCTTTTTCTGGTTTCAGTACGCGAGATACGACAAATCTTCTGTCTTTTTC  997

Query  4395410  CCATTTAACTAATTTTGTATACAGTTCTGTAGTTTCTCTACCTTCTTCTCCTTTAACGAA  4395469
               ||||||||||||||||||||||||||||||||||||||||||||||||||||||||||||
Sbjct  996      CCATTTAACTAATTTTGTATACAGTTCTGTAGTTTCTCTACCTTCTTCTCCTTTAACGAA  937
```

```
Query  4395470  TACAATTGATGAATTCGTTGCTTGTGAGGTGAGTGTAGAATAACTTTTGGCTTTAATTAA  4395529
                ||||||||||||||||||||||||||||||||||||||||||||||||||||||||||||
Sbjct  936      TACAATTGATGAATTCGTTGCTTGTGAGGTGAGTGTAGAATAACTTTTGGCTTTAATTAA  877

Query  4395530  ATATTTGCATCCAAGAGATTCTATCGTTTCGATAATTTTTTCATCAAAGTAGCCACTATC  4395589
                ||||||||||||||||||||||||||||||||||||||||||||||||||||||||||||
Sbjct  876      ATATTTGCATCCAAGAGATTCTATCGTTTCGATAATTTTTTCATCAAAGTAGCCACTATC  817

Query  4395590  CATTCGAAATAAAATTTCTAAATCGTCTGATTTGATGTTAGCAACAATTTCTTTGATCAT  4395649
                ||||||||||||||||||||||||||||||||||||||||||||||||||||||||||||
Sbjct  816      CATTCGAAATAAAATTTCTAAATCGTCTGATTTGATGTTAGCAACAATTTCTTTGATCAT  757

Query  4395650  TTCCGCAGCACCGTTTGCAGTGTAAGTATTGCCACTTCTTACAAATCCGGTAACATATGC  4395709
                ||||||||||||||||||||||||||||||||||||||||||||||||||||||||||||
Sbjct  756      TTCCGCAGCACCGTTTGCAGTGTAAGTATTGCCACTTCTTACAAATCCGGTAACATATGC  697

Query  4395710  TTTTAATTCGTCGCAAAATGCAAATTGGATATTGTAGCATCGGTTTCCCAGTTTCTTAGG  4395769
                ||||||||||||||||||||||||||||||||||||||||||||||||||||||||||||
Sbjct  696      TTTTAATTCGTCGCAAAATGCAAATTGGATATTGTAGCATCGGTTTCCCAGTTTCTTAGG  637

Query  4395770  ATTATATCCTTTTGACGCACCTTCTTGATGACCTTCTACGTTAATTACACTACTATCAAT  4395829
                ||||||||||||||||||||||||||||||||||||||||||||||||||||||||||||
Sbjct  636      ATTATATCCTTTTGACGCACCTTCTTGATGACCTTCTACGTTAATTACACTACTATCAAT  577

Query  4395830  ATCAATCGTAATGGATGTCAATTTACTTTTAGTGAGCAGTTTTTTAAAGACTTTAAAATT  4395889
                ||||||||||||||||||||||||||||||||||||||||||||||||||||||||||||
Sbjct  576      ATCAATCGTAATGGATGTCAATTTACTTTTAGTGAGCAGTTTTTTAAAGACTTTAAAATT  517

Query  4395890  AATGTCTCTAAACATTTGGGTTGTCTTGAAGTTGAAGTTTCCTAGAAACCGTG  4395942
                |||||||||||||||||||||||||||||||||||||||||||||||||||||
Sbjct  516      AATGTCTCTAAACATTTGGGTTGTCTTGAAGTTGAAGTTTCCTAGAAACCGTG  464
```

Figure S4: alignments between chromosomal ESBL integrations in i36 and i41 (A.), between i36 and the ISEcp1 sequence (B.) and between i41 and the ISEcp1 sequence (C.)

Table S10: mapping of *Shigella* miseq reads to reconstructed *Shigella* plasmids

		year	2013	2014	2015	2015	2015	2015	2016	2017	2017	2017	2017	2017	2017	2018	2018	2018	2018
		isolate	S13BD04362	S14BD05406	S15BD02407	S15BD06969	S15BD07413	S15BD09164	S17BD00672	S17BD04134	S17BD05916	S17BD05944	S17BD08179	S17BD08233	S18BD01106	S18BD03178	S18BD03411	S18BD04295	S18BD05612
year	ST	code	p3	p4	p5	p9	p12	p20	p31	p34	p38	p39	p54	p55	p59	p67	p69	p73	p83
2013	152	i1	82.3	87.4	66.3	74.9	91.5	96.4	99.1	28.8	5.9	35.2	21.9	80.8	21.9	26.4	24.9	63.0	25.2
2013	152	i2	33.0	35.8	49.9	31.3	41.9	36.6	37.2	44.5	6.0	99.8	34.7	33.3	34.7	38.4	72.0	47.4	39.3
2013	152	i3	[redacted]	79.8	68.5	83.8	86.3	89.1	89.1	34.3	5.7	31.5	25.9	88.0	25.9	34.2	30.6	65.1	29.8
2014	245	i4	78.4	[redacted]	66.6	73.1	93.6	95.5	95.8	82.7	4.3	36.8	63.2	76.1	63.2	73.5	32.0	63.3	71.7
2015	152	i5	70.4	67.8	[redacted]	63.6	73.6	73.2	74.4	44.6	4.7	52.3	40.6	69.0	40.6	38.3	39.8	95.1	45.6
2015	152	i6	81.0	87.8	65.8	73.8	90.4	95.0	99.6	38.0	4.8	34.0	29.0	77.6	29.0	35.1	26.1	62.6	33.1
2015	152	i7	81.0	88.0	66.3	74.6	90.7	95.1	99.8	38.0	4.9	34.5	29.0	80.4	29.1	34.9	26.3	63.0	33.1
2015	152	i8	80.8	88.0	65.7	73.2	90.7	95.1	99.7	23.5	4.9	34.1	17.9	78.9	17.9	22.7	24.9	62.5	20.4
2015	152	i9	94.4	83.8	70.3	[redacted]	87.2	89.9	91.2	40.3	6.1	34.0	33.7	96.8	33.8	38.1	35.5	69.5	34.9
2015	152	i10	15.0	13.6	16.0	16.3	17.1	13.4	15.3	100.0	4.6	16.2	76.7	19.7	76.7	86.3	13.6	15.2	87.2
2015	152	i11	43.8	39.3	37.0	42.3	43.8	41.5	43.6	100.0	4.6	21.8	76.4	46.8	76.4	86.0	19.6	35.1	86.9
2015	152	i12	80.5	88.7	67.5	72.0	[redacted]	93.9	93.5	29.6	6.6	40.7	22.6	77.7	22.6	24.3	26.2	64.1	26.1
2015	152	i13	13.9	12.6	21.6	15.4	16.3	12.2	14.2	100.0	4.8	16.7	89.0	18.8	89.0	86.4	13.9	20.5	100.0
2015	152	i14	82.0	87.8	65.4	74.7	90.3	97.5	94.8	35.7	4.8	33.9	27.1	79.6	27.1	33.9	26.7	62.1	31.2
2015	152	i15	68.0	67.6	97.3	61.1	73.4	73.0	74.3	42.5	4.7	52.1	38.6	64.4	38.6	35.8	38.2	94.8	43.6
2015	152	i16	67.9	67.6	97.3	59.7	73.4	72.9	74.2	29.5	4.6	52.1	29.0	63.3	28.9	24.8	37.6	94.8	32.5
2015	152	i17	79.1	99.9	66.0	73.8	93.3	95.3	95.6	38.6	4.9	36.2	30.0	79.5	30.0	37.0	31.0	62.8	33.5
2015	152	i18	79.3	100.0	66.5	73.8	93.4	95.5	95.8	38.2	4.9	36.2	29.7	79.6	29.7	36.5	30.9	63.2	33.3
2015	152	i19	81.6	88.4	65.9	72.4	91.8	100.0	95.8	23.3	6.0	35.1	17.5	78.2	17.5	22.0	25.7	62.7	20.1
2015	152	i20	81.6	88.7	66.3	72.7	92.0	[redacted]	95.8	23.8	6.0	35.5	17.9	78.4	17.8	22.2	25.9	63.0	20.5
2015	152	i21	81.9	88.6	67.7	73.4	91.9	100.0	95.9	25.9	6.0	36.3	19.6	79.2	19.6	24.0	26.5	64.3	22.6
2015	152	i22	79.3	100.0	66.1	73.9	93.3	95.4	95.8	39.5	5.0	36.3	30.6	79.7	30.6	37.7	31.1	62.9	34.3
2015	152	i23	69.5	69.1	94.9	61.8	75.1	74.5	75.8	29.4	5.0	52.0	26.4	67.1	26.4	24.5	37.8	90.2	29.7
2016	152	i24	80.9	88.1	66.1	74.6	90.8	95.1	99.8	37.9	4.8	34.5	28.9	80.4	29.0	35.0	26.2	62.8	33.0
2016	152	i25	70.8	68.2	99.9	64.3	74.0	73.5	75.0	44.8	4.9	52.5	40.6	69.7	40.6	38.3	39.9	95.0	45.9
2016	152	i26	78.7	88.1	74.4	70.6	95.3	91.5	93.4	100.0	4.8	37.1	89.0	76.2	89.0	86.3	28.2	70.7	100.0

2016	152	i27	82.0	88.4	80.4	72.8	91.9	100.0	95.9	26.0	6.1	85.9	19.6	78.6	19.6	24.2	69.8	76.4	22.5
2016	152	i28	81.8	88.4	66.9	74.7	91.4	98.4	96.1	37.8	5.4	35.8	28.6	80.4	28.7	34.2	26.9	63.6	33.0
2016	1503	i29	81.7	88.4	66.9	74.6	91.3	98.5	96.0	37.7	6.2	35.7	28.6	80.4	28.6	34.1	26.7	65.9	32.9
2017	152	i30	79.3	100.0	67.0	73.9	93.4	95.7	95.9	39.6	4.7	36.9	30.9	79.7	30.9	37.5	31.5	63.6	34.4
2017	152	i31	81.1	88.1	66.2	74.6	90.9	95.3	[redacted]	38.4	5.3	34.4	29.3	80.5	29.3	35.2	25.9	62.9	33.4
2017	145	i32	10.9	8.0	7.3	11.4	11.7	10.3	10.5	21.5	6.1	8.0	20.6	11.8	20.6	18.8	6.4	7.0	23.7
2017	152	i33	79.1	100.0	74.6	71.6	96.9	95.6	95.4	100.0	4.9	39.1	89.0	75.5	89.0	86.7	31.8	70.9	100.0
2017	152	i34	12.9	11.9	14.5	13.0	15.4	11.5	12.9	[redacted]	4.7	15.4	75.4	16.3	75.4	84.9	13.2	13.8	85.7
2017	152	i35	70.3	67.6	100.0	62.5	73.4	73.0	74.2	29.9	4.7	52.1	29.7	67.8	29.7	25.8	38.2	95.0	33.3
2017	152	i36	6.7	11.6	4.0	8.2	7.0	4.3	4.5	14.6	6.0	6.7	20.9	10.8	20.9	21.4	15.5	3.8	12.7
2017	152	i37	13.9	12.3	21.3	15.5	16.0	11.7	13.8	100.0	4.6	16.3	88.8	18.9	88.8	86.3	13.9	20.2	100.0
2017	152	i38	8.2	3.6	5.0	9.8	7.1	5.7	6.2	28.0	[redacted]	6.1	21.9	12.9	21.9	25.7	5.3	4.8	25.2
2017	152	i39	32.5	35.4	49.5	30.9	41.6	36.0	36.8	35.8	85.9	[redacted]	28.3	33.0	28.3	30.7	71.0	47.0	31.8
2017	152	i40	68.0	66.9	94.1	64.6	72.8	71.6	73.0	44.2	5.2	52.6	39.6	68.2	39.5	37.6	40.7	89.4	43.0
2017	152	i41	7.6	5.2	8.2	10.0	9.0	5.0	6.3	33.6	5.6	9.3	26.3	13.1	26.3	29.7	7.8	7.8	29.2
2017	152	i42	13.4	12.0	21.1	15.0	15.7	11.5	13.6	100.0	4.6	16.1	89.0	18.4	89.0	86.2	13.3	20.0	100.0
2017	152	i43	79.2	100.0	66.2	73.9	93.2	95.4	95.8	39.0	4.7	36.5	30.4	79.7	30.4	37.2	31.4	62.9	33.9
2017	152	i44	81.0	79.2	70.7	75.6	84.7	86.4	85.8	100.0	5.3	34.5	91.4	81.2	91.4	86.3	30.6	67.2	100.0
2017	152	i45	94.1	83.7	69.9	91.9	87.0	89.9	91.2	26.3	6.1	33.7	20.6	100.0	20.5	26.1	31.2	66.5	22.8
2017	152	i46	13.6	12.5	21.2	15.3	16.1	12.0	14.0	100.0	4.6	16.1	88.8	18.7	88.8	86.2	13.5	20.1	100.0
2017	152	i47	94.1	83.7	70.1	91.9	87.2	89.9	91.2	25.8	6.0	34.0	20.1	100.0	20.2	25.9	31.5	66.6	22.2
2017	152	i48	94.2	83.7	70.2	91.9	87.2	89.9	91.2	26.6	6.2	34.0	21.1	100.0	21.1	26.4	31.3	66.8	23.5
2017	152	i49	12.8	11.9	20.2	15.1	15.3	11.5	13.0	100.0	4.6	15.7	100.0	16.3	100.0	91.5	21.3	19.2	99.9
2017	152	i50	13.3	11.9	20.8	16.9	15.5	11.5	13.5	100.0	4.6	16.1	100.0	18.2	100.0	91.6	21.4	19.8	99.9
2017	152	i51	86.9	84.0	68.6	78.1	90.5	91.1	90.5	99.2	4.8	35.1	80.3	84.1	80.4	90.9	37.2	65.2	86.1
2017	152	i52	94.1	83.7	70.3	91.9	87.2	89.8	91.2	40.1	6.0	33.8	31.0	100.0	31.1	37.9	32.3	66.8	34.8
2017	152	i53	94.1	83.6	69.9	91.9	87.1	89.9	91.2	25.6	6.1	34.0	19.9	100.0	19.9	25.7	31.5	66.5	22.0
2017	152	i54	14.4	13.0	21.3	18.0	16.4	12.7	14.5	100.0	4.7	16.1	[redacted]	19.4	100.0	91.6	21.4	20.3	99.9
2017	152	i55	94.0	83.6	69.8	91.9	86.9	89.8	91.1	26.2	6.0	33.5	20.4	[redacted]	20.4	26.2	31.2	66.4	22.6
2017	152	i56	94.1	83.6	69.9	91.9	87.0	89.8	91.1	33.5	6.1	33.8	25.8	100.0	25.7	32.3	31.1	66.5	28.8

2017	152	i57	14.5	12.7	20.9	18.0	16.5	12.3	14.3	100.0	4.7	16.2	100.0	19.3	100.0	91.7	21.8	19.9	99.9
2018	152	i58	16.9	15.7	24.1	19.7	19.1	15.6	17.0	100.0	4.6	16.5	100.0	21.1	100.0	91.6	22.1	22.9	99.9
2018	152	i59	86.2	82.0	74.3	80.2	88.8	88.7	88.8	100.0	4.6	39.5	100.0	84.1	[redacted]	91.5	41.4	70.7	99.9
2018	152	i60	86.1	81.7	73.7	78.5	88.6	88.5	88.6	100.0	4.7	39.6	100.0	82.3	100.0	91.9	41.3	70.1	99.9
2018	-	i61	12.6	11.8	20.3	15.3	15.3	11.1	12.8	100.0	4.4	15.2	100.0	16.6	100.0	91.5	20.8	19.3	99.9
2018	152	i62	81.0	88.1	66.3	74.7	90.9	95.1	99.8	38.4	4.9	35.5	29.8	80.5	29.8	35.4	27.0	63.0	33.9
2018	152	i63	12.8	13.1	20.3	12.1	16.6	11.4	12.9	98.8	4.6	16.8	86.3	13.4	86.3	90.6	17.6	19.3	92.9
2018	152	i64	15.5	14.4	23.0	17.0	18.2	14.2	15.9	100.0	4.6	16.4	92.5	20.4	92.5	90.3	16.4	21.9	100.0
2018	152	i65	12.9	11.9	20.1	15.3	15.3	11.5	13.0	100.0	4.7	15.6	100.0	16.6	100.0	91.6	21.3	19.1	99.9
2018	245	i66	13.3	13.2	22.1	14.0	16.8	13.0	14.9	100.0	4.1	17.4	89.0	14.3	89.0	84.4	14.2	21.0	100.0
2018	152	i67	31.2	30.6	44.3	31.2	32.4	31.3	34.0	94.6	4.1	87.6	76.6	33.3	76.7	[redacted]	77.6	42.1	81.9
2018	152	i68	31.0	30.8	44.7	31.3	32.5	31.4	34.0	94.7	4.2	87.6	77.6	33.3	77.6	100.0	77.6	42.5	83.0
2018	152	i69	36.3	33.2	41.8	33.9	31.5	31.3	31.4	26.4	5.0	83.5	29.7	32.7	29.7	34.9	[redacted]	39.7	22.7
2018	152	i70	31.2	30.7	44.3	31.2	32.4	31.3	34.0	94.6	4.2	87.5	76.9	33.3	76.9	100.0	77.6	42.1	82.2
2018	152	i71	31.5	31.0	44.6	31.7	32.7	31.6	34.3	94.6	4.1	87.7	77.6	33.4	77.6	100.0	78.0	42.4	82.4
2018	152	i72	31.1	30.8	44.7	31.2	32.5	31.3	34.1	94.6	4.1	87.5	77.1	33.2	77.1	100.0	77.5	42.5	82.4
2018	152	i73	70.2	67.8	100.0	66.3	73.6	73.0	74.3	44.3	4.7	52.5	40.4	69.3	40.4	37.9	39.8	[redacted]	45.6
2018	152	i74	31.1	30.6	44.3	31.2	32.3	31.2	33.9	94.5	4.1	87.4	77.0	33.2	77.0	100.0	77.5	42.1	82.2
2018	152	i75	31.3	31.0	45.1	31.4	32.6	31.5	34.1	94.6	4.1	87.5	77.1	33.4	77.2	100.0	77.6	42.9	82.4
2018	152	i76	31.2	30.8	44.7	31.3	32.5	31.4	34.0	94.6	4.1	87.6	77.1	33.4	77.1	100.0	77.5	42.5	82.4
2018	152	i77	30.9	30.9	45.2	31.1	33.1	31.0	33.7	95.1	4.1	88.5	77.7	33.1	77.7	100.0	77.6	43.0	83.1
2018	152	i78	31.2	30.7	45.0	31.3	32.5	31.4	34.0	94.5	4.1	87.5	77.4	33.3	77.5	100.0	77.6	42.7	82.8
2018	152	i79	31.4	31.1	45.1	31.4	32.8	31.6	34.3	94.6	4.1	87.5	77.2	33.5	77.2	100.0	77.6	42.9	82.5
2018	152	i80	31.7	30.7	44.4	31.7	32.4	31.3	34.0	94.6	4.1	87.6	76.7	33.8	76.7	100.0	78.3	42.2	81.9
2018	152	i81	13.3	11.9	20.4	14.9	15.5	11.5	13.5	99.5	4.7	16.1	82.9	18.2	82.9	85.0	12.6	19.4	94.9
2018	152	i82	80.9	88.4	66.4	74.8	90.9	95.2	99.8	39.6	4.9	34.8	30.2	80.6	30.3	36.7	26.5	63.2	34.5
2018	152	i83	13.6	12.6	21.4	15.1	16.1	12.3	14.3	100.0	4.6	16.7	87.7	18.5	87.7	86.2	13.6	20.3	100.0
2018	245	i84	13.4	13.2	21.8	17.3	16.5	13.0	14.9	100.0	4.5	16.7	100.0	14.0	100.0	89.7	23.1	20.7	100.0
2018	152	i85	34.1	30.1	52.6	32.9	34.4	30.8	32.6	100.0	4.6	86.7	89.0	37.0	89.0	86.3	78.7	50.0	100.0
2018	152	i86	70.1	67.7	100.0	66.3	73.5	73.0	74.3	45.2	4.7	52.8	40.9	69.3	40.9	38.7	40.1	100.0	46.1

2018	152	i87	14.8	13.9	21.5	16.2	17.4	13.6	15.2	100.0	4.7	18.9	87.7	19.5	87.7	85.9	15.6	20.5	100.0
2018	152	i88	79.1	100.0	66.6	73.5	93.4	95.5	95.8	40.9	4.8	37.8	32.0	77.3	32.0	38.9	32.4	63.4	35.8
2018	148	i89	13.0	12.3	20.9	17.5	15.6	11.9	13.9	100.0	5.2	16.9	100.0	14.9	100.0	91.7	23.7	20.0	100.0
2018	152	i90	14.2	13.2	21.5	18.0	16.8	12.8	14.4	100.0	4.7	18.7	100.0	19.2	100.0	91.6	23.3	20.4	99.9
2018	152	i91	81.1	88.3	66.1	74.7	90.9	95.3	100.0	38.9	4.8	34.3	29.7	80.5	29.7	35.8	26.1	62.8	33.8
2018	152	i92	81.2	88.3	66.3	73.6	91.0	95.4	100.0	26.2	85.7	34.6	21.0	79.1	21.0	25.1	26.7	63.0	23.1
2018	152	i93	14.4	12.8	15.6	15.9	16.2	12.4	14.5	100.0	4.7	16.9	77.1	19.3	77.1	86.2	14.0	14.8	87.7
2018	152	i94	13.7	12.7	21.2	14.9	16.1	12.0	14.0	100.0	4.6	16.8	89.0	18.3	89.0	86.2	14.2	20.2	100.0
2018	8878	i95	13.4	13.8	13.5	16.5	15.4	11.8	14.1	94.7	2.9	15.5	84.0	17.7	84.0	89.5	21.0	12.8	83.7
2018	152	i96	80.8	88.0	65.6	73.2	90.6	95.1	99.8	26.7	4.9	34.0	20.2	77.0	20.2	25.3	24.7	62.4	23.0
2018	152	i97	80.8	88.1	66.2	73.8	90.8	95.1	99.8	38.2	5.0	34.3	29.2	77.6	29.3	35.2	26.0	62.9	33.3
2018	152	i98	31.4	30.8	44.5	31.3	32.5	31.5	34.2	94.5	4.1	87.4	76.9	33.4	76.9	100.0	77.5	42.3	82.2
2018	152	i99	85.3	86.5	73.9	79.2	92.5	90.1	91.4	99.9	4.6	38.2	87.0	85.2	87.0	96.6	36.9	70.3	93.8
2018	145	i100	6.1	5.9	13.5	11.6	11.4	4.6	5.3	27.0	5.4	12.1	33.8	12.5	33.8	22.3	8.1	12.8	35.6

Yellow = mapping percentage > 75%, Red = mapping percentage of 100%, black = same isolate as reference and subject

Table S11: mapping of *Salmonella* miseq reads to reconstructed *Shigella* plasmids

isolate	S13BD04362	S14BD05406	S15BD02407	S15BD06969	S15BD07413	S15BD09164	S17BD00672	S17BD04134	S17BD05916	S17BD05944	S17BD08179	S17BD08233	S18BD01106	S18BD03178	S18BD03411	S18BD04295	S18BD05612
code	p3	p4	p5	p9	p12	p20	p31	p34	p38	p39	p54	p55	p59	p67	p69	p73	p83
i101	4.4	7.3	8.7	6.2	7.2	3.6	2.2	10.1	5.8	7.7	13.8	6.6	13.7	8.9	12.3	8.3	12.4
i102	1.9	6.6	10.2	6.3	9.2	2.3	3.8	25.5	5.0	8.4	23.7	8.6	23.8	20.5	5.3	9.7	26.0
i103	74.4	74.0	57.0	69.8	78.6	80.3	82.1	15.9	5.0	29.2	17.3	74.3	17.3	18.0	33.2	54.2	13.7
i104	86.4	85.4	67.4	82.6	91.7	90.5	89.7	77.5	5.2	37.6	59.6	81.4	59.5	63.9	33.4	64.0	66.3
i105	5.3	2.9	2.7	3.9	4.4	4.5	3.1	57.7	5.0	4.3	42.7	4.0	42.7	48.3	4.1	2.6	49.2
i106	14.4	14.8	13.2	12.2	14.4	13.3	14.0	84.6	4.2	14.3	66.8	12.5	66.8	77.4	17.1	12.5	72.1
i107	74.4	74.0	57.0	69.4	78.2	80.4	81.6	15.9	5.1	29.0	17.4	74.4	17.4	17.9	33.4	54.2	13.6
i108	3.0	5.0	9.1	3.8	7.1	2.3	2.3	10.1	4.0	8.0	12.3	4.0	12.3	6.8	4.8	8.6	12.8
i109	2.9	4.9	9.0	3.8	7.0	2.2	2.2	10.0	4.1	7.9	12.0	4.0	11.9	6.6	4.7	8.6	12.4
i110	3.0	5.0	9.5	3.8	7.0	2.3	2.2	10.1	4.1	8.4	12.1	4.0	12.1	6.8	5.2	9.1	12.6
i111	4.4	2.1	2.0	5.2	0.3	0.3	0.7	4.1	2.5	1.2	8.6	4.6	8.6	8.8	9.6	1.9	4.3

i112	15.8	13.3	15.0	17.5	17.4	11.1	13.3	72.4	2.6	16.9	59.6	15.3	59.5	64.7	19.4	14.2	62.9
i113	93.6	83.5	70.0	96.2	85.7	89.9	89.8	49.9	5.3	33.9	43.2	94.7	43.2	47.1	38.4	66.5	42.5
i114	89.1	82.2	67.7	81.8	87.8	89.2	88.6	76.1	5.0	35.8	57.3	83.6	57.2	66.4	32.5	64.3	64.8
i115	6.6	10.1	13.6	9.8	10.8	5.3	4.1	10.0	5.2	9.4	19.9	11.6	19.9	13.0	13.1	12.9	16.2
i116	4.8	8.9	12.4	8.1	9.5	4.0	2.6	10.7	5.2	9.6	20.3	9.9	20.3	13.6	12.9	11.8	16.7
i117	3.6	5.7	13.8	10.6	9.9	4.5	3.8	10.4	5.1	10.9	24.9	9.3	24.8	7.8	8.8	13.1	22.3
i118	4.2	2.4	2.2	5.1	0.6	0.7	1.0	3.6	2.6	2.3	8.3	4.8	8.3	8.9	10.3	2.1	3.9
i119	78.0	82.7	58.3	68.7	84.4	86.4	83.4	48.2	4.1	29.9	36.3	73.6	36.2	44.5	28.6	55.4	41.0
i120	79.7	88.2	66.0	71.4	89.6	95.4	98.4	47.9	4.2	34.0	39.5	73.2	39.5	44.2	29.7	62.7	40.8
i121	16.3	13.8	15.5	17.9	18.0	11.9	13.8	72.5	2.5	17.0	59.5	15.8	59.4	64.8	19.5	14.7	62.8
i122	4.3	4.0	8.8	9.1	6.3	2.5	1.5	5.5	4.3	6.6	19.9	8.1	19.9	11.0	12.4	8.3	11.1
i123	4.6	4.4	5.4	7.6	6.7	1.1	1.1	8.7	3.3	7.5	12.8	7.1	12.8	9.8	11.1	5.2	9.0
i124	74.4	74.1	57.1	69.4	78.1	80.4	81.6	16.0	5.2	29.0	17.3	74.4	17.3	17.9	33.3	54.2	13.7
i125	84.2	74.0	67.4	74.1	75.7	80.7	77.3	7.2	4.7	23.5	12.6	79.9	12.6	9.8	24.6	64.0	13.4
i126	80.1	81.2	63.0	72.5	88.2	86.0	86.6	17.4	3.2	33.6	13.3	76.4	13.3	12.9	27.5	59.9	15.3
i127	74.1	77.7	59.3	66.4	79.3	84.0	83.0	14.6	2.8	27.1	14.8	71.7	14.8	18.0	30.0	56.3	12.6
i128	83.5	90.4	67.7	77.6	90.8	98.7	97.1	16.7	5.2	35.0	16.3	83.2	16.3	16.8	35.0	64.3	14.4
i129	3.6	1.8	1.6	4.5	0	0	0.4	3.3	2.6	1.1	8.1	3.9	8.1	8.2	9.2	1.5	3.7
i130	74.0	77.7	59.3	66.4	79.3	84.1	83.1	14.4	2.9	27.3	14.5	71.7	14.4	17.6	30.1	56.3	12.3
i131	4.7	3.4	2.5	5.7	2.0	1.7	1.5	4.3	2.6	4.1	8.9	5.1	8.8	8.9	11.5	2.4	4.5

Yellow = mapping percentage > 75%, Red =mapping percentage of 100%

Table S12: mapping of *Salmonella* miseq reads to reconstructed *Salmonella* plasmids

		S16BD03602 i105	S16BD04351 i106	S16BD07964 i114	S17BD06931 i123	S18BD02037 i127	S18BD07928 i131
isolate	serovar	p105	p106	p114	p123	p127	p131
i101	MONOPHASIC TM 4,12:I:-	5.72	6.48	13.8191	59.5752	9.11725	59.7997
i102	RISSEN	23.24	15.09	17.8329	6.93316	7.78178	4.85727
i103	INFANTIS	5.65	16.18	16.4079	6.22717	85.7452	6.28158
i104	TYPHIMURIUM	66.76	49.56	50.3861	6.81196	31.3497	5.32315

i105	ENTERITIDIS	100.00	32.90	29.8737	0.130225	4.06167	0.133363
i106	ENTERITIDIS	67.98	100.00	52.7593	3.78902	19.3631	3.57982
i107	INFANTIS	5.65	16.10	15.8021	6.34663	85.6052	6.4089
i108	MONOPHASIC TM 4,5,12:I:-	3.46	7.37	14.2982	6.18688	7.14167	4.22566
i109	TYPHIMURIUM	3.46	7.25	14.2513	6.14452	6.91124	4.18263
i110	MONOPHASIC TM 4,5,12:I:-	3.47	7.35	14.2365	6.20112	7.10808	4.2598
i111	TYPHIMURIUM	1.22	5.35	5.80699	96.0915	8.30919	100
i112	TYPHIMURIUM	62.06	49.90	66.9528	97.5848	12.9165	99.7873
i113	TYPHIMURIUM	47.51	32.12	38.257	7.02136	30.3705	6.13328
i114	MONOPHASIC TM 4,5,12:I:-	67.65	55.98	100	5.25135	29.3594	4.18654
i115	NEWPORT	5.69	10.83	9.82319	71.7551	9.77243	70.0281
i116	NEWPORT	6.24	11.10	10.2015	71.7767	9.50559	70.0292
i117	TYPHI	6.24	7.66	11.0199	9.38208	8.18707	7.57716
i118	TYPHIMURIUM VAR. O:5-	44.74	4.94	5.38177	96.103	8.27277	100
i119	DUBLIN	44.73	30.92	33.7038	4.37589	30.4352	3.97351
i120	MONOPHASIC TM 4,5,12:I:-	62.07	30.00	28.9967	4.58148	28.6448	3.97529
i121	INFANTIS	3.56	50.10	67.0364	97.5768	13.0719	99.7777
i122	AGONA	0.26	7.43	5.65926	10.7149	9.6478	7.76885
i123	INFANTIS	5.65	6.79	13.5525	100	11.6384	98.5647
i124	INFANTIS	4.38	16.27	15.9851	6.38621	83.7511	6.47007
i125	INFANTIS	0.83	8.67	12.5559	5.52291	26.7961	4.67803
i126	TYPHIMURIUM	1.16	12.54	9.57071	2.3562	25.0252	0.453435
i127	INFANTIS	10.69	14.84	15.0408	9.31783	100	8.14903
i128	TYPHIMURIUM	0.00	15.48	16.3188	6.44629	88.8139	6.71439
i129	TYPHIMURIUM VAR. O:5-	0.68	5.17	5.36457	96.0933	8.1337	100
i130	TYPHIMURIUM	2.11	14.65	14.0153	9.23622	99.676	8.08145
i131	TYPHIMURIUM	0.00	5.43	5.77729	96.1186	8.20967	100

Yellow = mapping percentage > 75%, Red =mapping percentage of 100%

Table S13: mapping of *Shigella* miseq reads to reconstructed *Salmonella* plasmids

		year	2016	2016	2016	2017	2018	2018
		isolate	S16BD03602	S16BD04351	S16BD07964	S17BD06931	S18BD02037	S18BD07928
year	ST	code	p105	p106	p114	p123	p127	p131
2013	152	i1	24.3033	20.6332	33.9751	2.60588	26.2809	1.87206
2013	152	i2	36.0294	28.3067	40.1307	4.44639	12.12	3.02503
2013	152	i3	31.6805	24.2664	39.392	2.30063	25.8901	2.32336
2014	245	i4	73.3545	51.7028	62.6708	4.1408	27.3097	3.10932
2015	152	i5	33.5914	27.7878	42.3514	4.79713	22.9328	3.15235
2015	152	i6	33.7869	25.7948	38.0139	1.92281	26.237	1.96702
2015	152	i7	33.6892	25.7332	40.0377	3.47196	27.1581	2.74942
2015	152	i8	15.3678	17.417	29.8995	3.30701	26.8821	2.68398
2015	152	i9	35.7975	28.3869	44.186	4.56481	27.8437	4.33306
2015	152	i10	71.7925	50.8609	58.8227	5.52083	8.75246	4.04713
2015	152	i11	71.7867	51.0536	59.4863	5.21628	15.8006	3.71532
2015	152	i12	20.0655	19.4011	31.609	4.21824	26.0769	1.8678
2015	152	i13	71.9305	51.4252	59.3612	5.20447	8.32331	3.74982
2015	152	i14	33.8004	24.2647	40.4731	2.92745	26.6112	2.23019
2015	152	i15	31.2052	26.4829	37.4644	3.19623	21.295	2.2942
2015	152	i16	14.8081	19.5598	28.5628	3.30423	21.2439	2.31803
2015	152	i17	32.2421	27.919	40.4129	4.13003	27.3684	3.1143
2015	152	i18	31.738	27.1645	39.5648	4.04912	27.3128	3.07162
2015	152	i19	17.7866	17.1871	30.3239	2.55518	26.5632	1.84539
2015	152	i20	17.8211	17.4882	30.61	2.73159	26.8576	2.02819
2015	152	i21	21.1676	18.6936	32.1741	2.56247	26.5553	1.81445
2015	152	i22	32.9992	27.936	40.6278	4.16024	27.5423	3.14524
2015	152	i23	14.9001	19.5614	30.8859	4.68809	22.5436	3.067
2016	152	i24	33.762	25.7697	40.1487	3.46641	27.1719	2.76151

2016	152	i25	34.0955	28.1165	43.0064	4.76101	23.0785	3.13279
2016	152	i26	72.0704	50.9654	56.4042	4.96069	27.7106	3.45713
2016	152	i27	28.9934	19.1251	31.3057	2.5215	26.6407	1.81374
2016	152	i28	36.1023	25.3681	40.7654	2.63332	26.819	1.92897
2016	1503	i29	36.1119	25.2556	41.8629	2.63922	26.7926	1.9343
2017	152	i30	33.5339	28.2306	40.7763	4.70545	27.9526	3.7676
2017	152	i31	34.2565	25.785	40.2448	3.60809	27.418	2.91656
2017	145	i32	27.2377	15.9137	33.7679	1.83148	5.53025	1.67042
2017	152	i33	72.6339	51.749	54.5149	4.41097	26.9126	3.22099
2017	152	i34	71.6065	49.6353	55.1504	5.21662	8.00342	3.70536
2017	152	i35	14.7966	19.5193	32.4164	5.13293	22.3003	3.47136
2017	152	i36	17.4704	15.7097	24.531	6.75466	7.03305	5.84842
2017	152	i37	71.848	51.1167	59.0915	5.49721	8.32488	4.03646
2017	152	i38	35.6499	18.7357	34.294	3.207	4.72124	2.53355
2017	152	i39	25.3306	23.2399	35.726	4.63773	11.7596	2.966
2017	152	i40	33.2426	28.038	40.9999	7.40023	23.8564	5.53724
2017	152	i41	34.8622	21.0647	35.1304	4.51793	5.94025	3.20676
2017	152	i42	71.8595	51.1046	58.8641	5.54896	8.23133	4.11506
2017	152	i43	48.0814	27.9481	40.4277	4.11822	27.4667	3.07447
2017	152	i44	71.848	53.2183	60.7354	8.85388	30.8794	7.42211
2017	152	i45	18.2754	20.3782	37.3706	4.43215	28.0616	3.40485
2017	152	i46	72.287	51.2317	59.122	5.53576	8.41938	4.09727
2017	152	i47	17.5164	20.1054	37.2307	4.44951	28.1912	3.43472
2017	152	i48	17.572	20.0965	37.0845	4.48459	28.1102	3.40699
2017	152	i49	71.7158	52.0493	54.9964	7.1818	8.50194	5.76307
2017	152	i50	71.7024	53.385	58.7562	7.19916	8.85449	5.80183
2017	152	i51	72.4	55.8347	59.899	5.33817	28.0681	4.07914
2017	152	i52	35.7707	28.3545	45.8275	4.57904	28.3246	3.49945
2017	152	i53	17.5662	20.2479	37.251	4.43389	28.0823	3.44006
2017	152	i54	71.8097	52.96	58.6874	7.29813	8.98854	5.87083

2017	152	i55	17.7828	20.5264	37.3183	4.43354	28.1039	3.39489
2017	152	i56	27.715	24.2761	41.3204	4.51272	28.0973	3.43472
2017	152	i57	71.8059	53.5113	59.022	7.30959	9.11882	5.88505
2018	152	i58	71.7426	52.6872	56.1165	7.19639	9.71341	5.79685
2018	152	i59	73	55.0146	61.0934	7.26237	28.805	5.82068
2018	152	i60	73.2376	54.1314	57.7955	7.20993	28.669	5.77587
2018	-	i61	71.2807	51.7555	54.9354	6.89947	8.12679	5.33631
2018	152	i62	33.8828	25.9291	40.273	3.50009	27.1819	2.81592
2018	152	i63	70.146	54.7434	54.2913	4.08801	6.88613	2.68789
2018	152	i64	71.8691	53.4587	58.9508	4.98569	8.78605	3.50585
2018	152	i65	71.597	52.0914	55.4021	7.14534	8.48781	5.72999
2018	245	i66	71.804	53.0329	58.4959	4.0446	7.64208	3.30563
2018	152	i67	71.9975	52.3464	59.9006	6.58381	14.5904	5.93875
2018	152	i68	72.4518	53.8295	60.9613	6.72237	14.7856	6.04687
2018	152	i69	17.848	23.4771	31.1455	6.2046	13.6979	6.46723
2018	152	i70	72.1432	52.2468	59.8896	6.60534	14.5072	5.91741
2018	152	i71	71.4417	52.4622	60.1015	6.7564	14.8076	6.10803
2018	152	i72	71.4379	53.5801	60.7549	6.72063	14.7436	6.06785
2018	152	i73	33.2254	27.8315	42.6672	5.46179	23.1523	3.86291
2018	152	i74	71.2079	52.4719	59.7888	6.5845	14.6048	5.92168
2018	152	i75	71.3919	53.6149	60.9136	6.79286	14.8811	6.12724
2018	152	i76	71.712	52.4225	60.1038	6.49387	14.6145	5.82104
2018	152	i77	72.0033	53.9436	60.8605	7.81625	14.8738	6.88652
2018	152	i78	72.172	53.7153	61.0574	6.95122	15.023	6.10412
2018	152	i79	72.2946	53.7825	61.0066	6.67271	14.9906	5.99992
2018	152	i80	71.1734	54.1703	61.6062	6.90017	14.5759	6.23002
2018	152	i81	71.6621	50.848	58.7007	4.87526	7.28639	3.33088
2018	152	i82	35.0769	26.5646	40.7756	3.56329	27.2871	2.88207
2018	152	i83	72.0895	50.7185	58.9618	5.47082	8.38547	3.99912
2018	245	i84	71.8423	55.9933	61.9009	8.08122	9.18129	7.7781

2018	152	i85	87.6989	53.0402	60.9777	5.47603	13.2439	4.04108
2018	152	i86	34.015	28.1157	42.8579	5.31837	23.0157	3.65166
2018	152	i87	72.952	50.7727	58.954	5.49235	8.62469	4.0137
2018	152	i88	35.5924	28.8531	39.3811	3.21637	26.8168	2.95355
2018	148	i89	71.9937	55.1968	64.3506	9.26088	9.31628	8.14938
2018	152	i90	73.1073	53.4433	59.0361	7.28563	9.14613	5.87154
2018	152	i91	34.8756	26.2068	40.3972	3.47613	27.4268	2.77324
2018	152	i92	18.193	18.9688	31.6559	3.6074	27.1414	2.90696
2018	152	i93	71.7752	50.7937	58.9532	5.30275	8.44763	3.82841
2018	152	i94	71.7196	50.763	58.9555	5.5441	8.38798	4.06989
2018	8878	i95	85.2724	56.8207	61.0965	9.52827	10.0751	8.54022
2018	152	i96	19.4101	19.9071	30.9907	1.86621	26.0389	1.85713
2018	152	i97	33.8981	25.8579	38.2367	2.0072	26.1362	2.03174
2018	152	i98	71.2366	52.2978	59.8756	6.59944	14.629	5.93235
2018	152	i99	72.0167	57.5776	58.2497	5.66286	27.2752	3.50407
2018	145	i100	27.4198	21.3432	34.9655	8.37292	8.96656	6.2069

Yellow = mapping percentage > 75%, Red =mapping percentage of 100%

A.

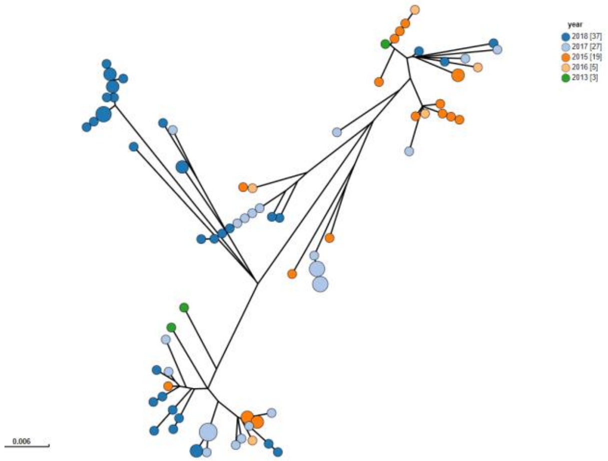

B.

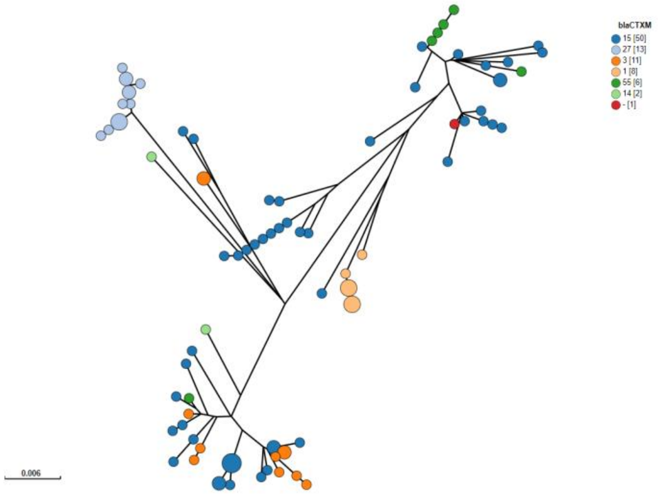

Figure S5: cgMLST tree of all *Shigella* isolates and coloured by year of isolation (A.) and *bla*$_{CTX-M}$ variant (B.)

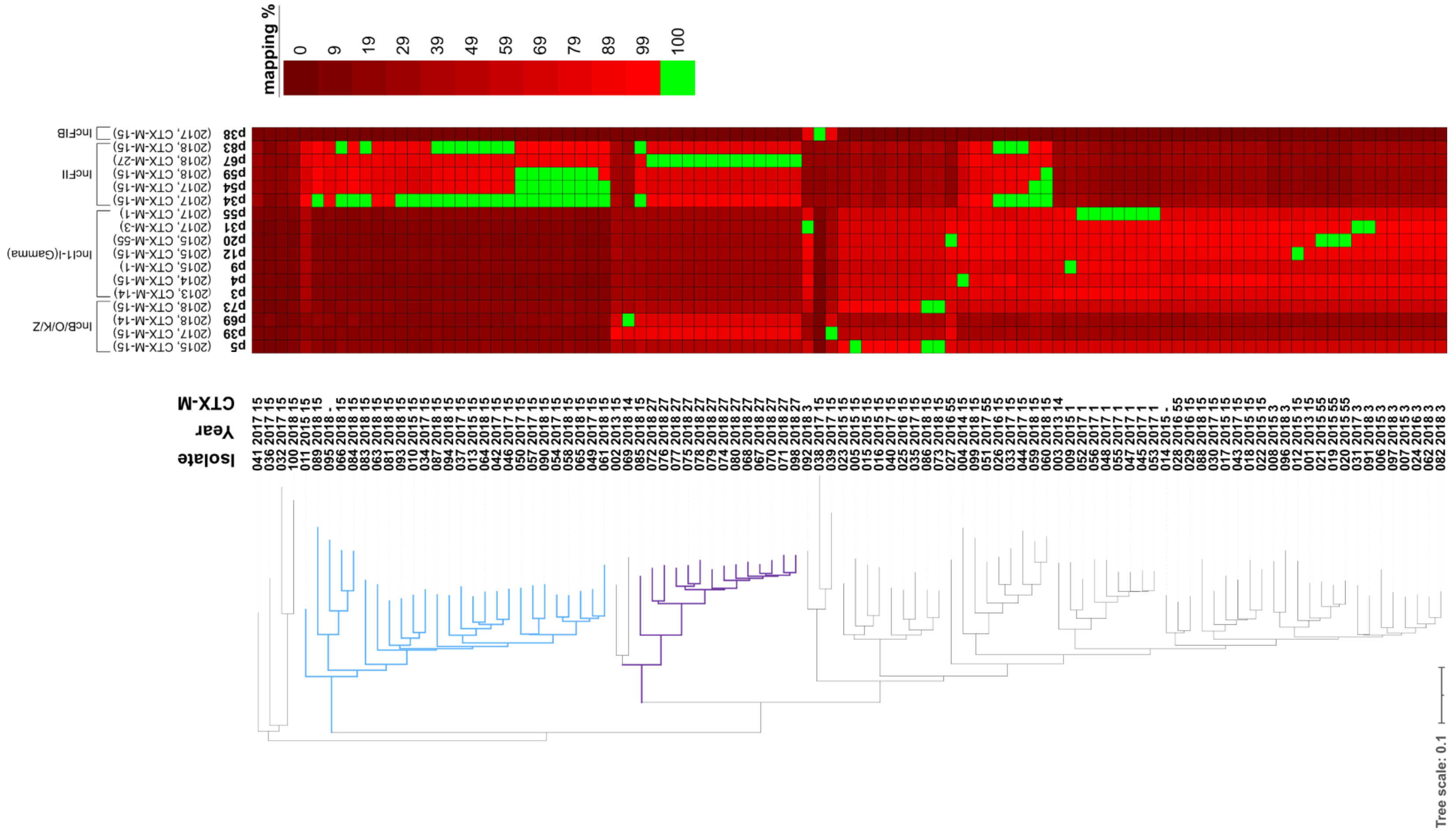

Figure S6: High similarity of ESBL plasmids in *bla*$_{CTX\text{-}M\text{-}15}$ and *bla*$_{CTX\text{-}M\text{-}27}$ clusters in *Shigella*. Neighbourjoining tree based on plasmid alignment with filtered short read assemblies between the 100 *Shigella* isolates with annotations of the isolate, year, *bla*$_{CTX\text{-}M}$ variant and mapping percentage to the reconstructed *Shigella* ESBL plasmids. The distance between nodes is based on the average nucleotides aligned between genome pairs and then normalized to a value between 0 to 1.The mapping percentage is displayed as a heatmap where light red is a high and dark red is a low mapping percentage to the reference (breadth of coverage). Light green colours in the heatmap indicate 100% alignment to the reference plasmids. The branches of the clusters are indicated with bold blue lines for *bla*$_{CTX\text{-}M\text{-}15}$ and bold purple lines for *bla*$_{CTX\text{-}M\text{-}27}$.

A.

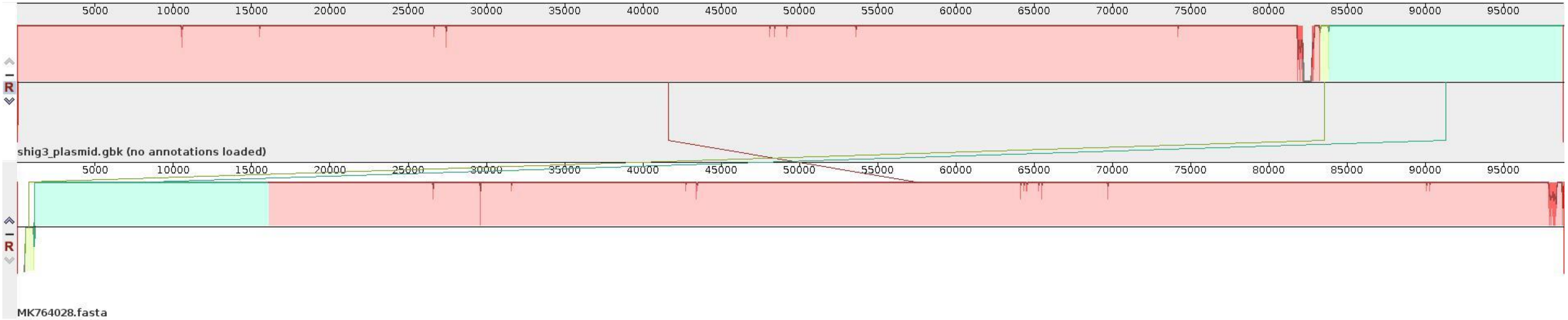
5000 10000 15000 20000 25000 30000 35000 40000 45000 50000 55000 60000 65000 70000 75000 80000 85000 90000 95000
shig3_plasmid.gbk (no annotations loaded)
5000 10000 15000 20000 25000 30000 35000 40000 45000 50000 55000 60000 65000 70000 75000 80000 85000 90000 95000
MK764028.fasta

B.

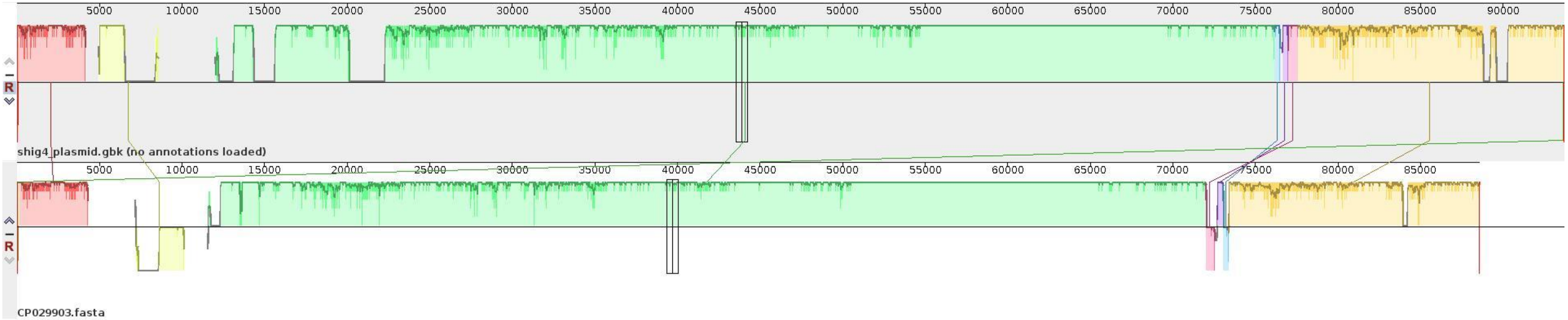
5000 10000 15000 20000 25000 30000 35000 40000 45000 50000 55000 60000 65000 70000 75000 80000 85000 90000
shig4_plasmid.gbk (no annotations loaded)
5000 10000 15000 20000 25000 30000 35000 40000 45000 50000 55000 60000 65000 70000 75000 80000 85000
CP029903.fasta

C.

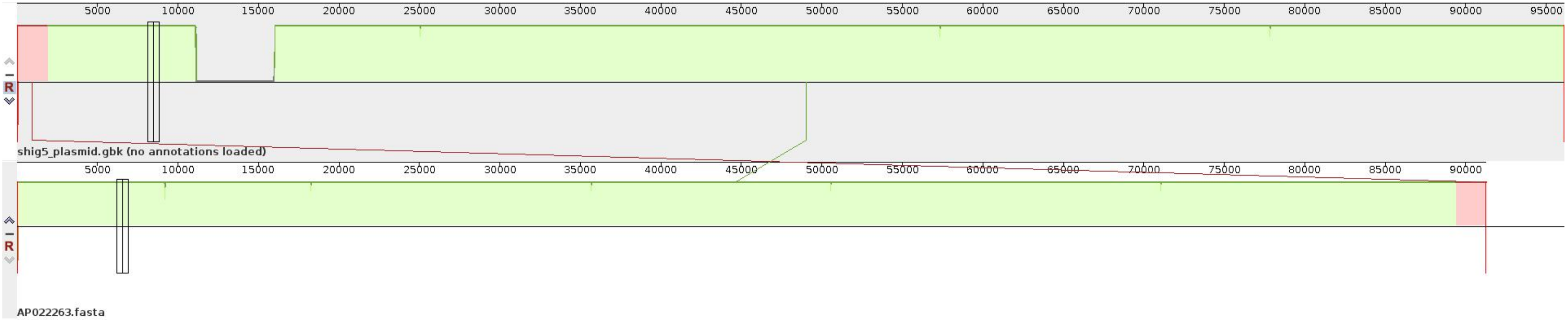

5000 10000 15000 20000 25000 30000 35000 40000 45000 50000 55000 60000 65000 70000 75000 80000 85000 90000 95000
R
shig5_plasmid.gbk (no annotations loaded)
5000 10000 15000 20000 25000 30000 35000 40000 45000 50000 55000 60000 65000 70000 75000 80000 85000 90000
R
AP022263.fasta

D.

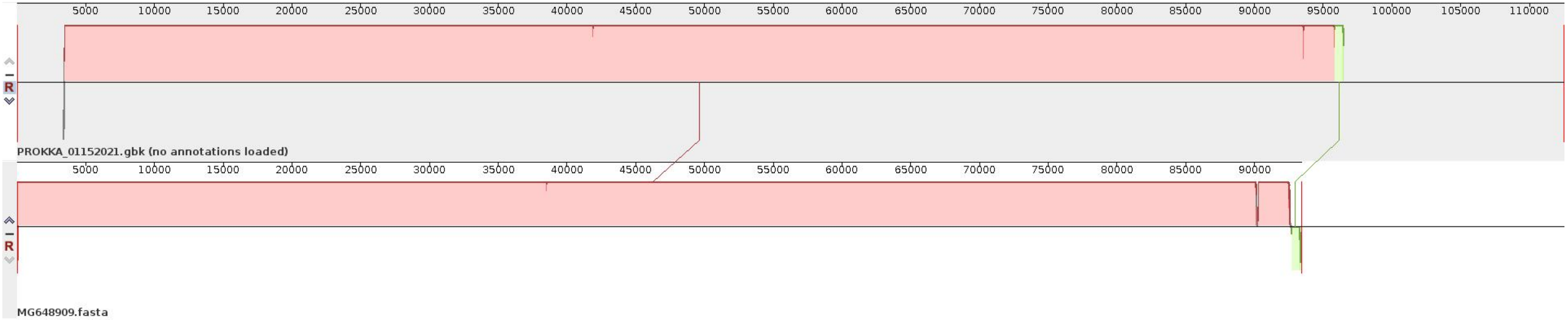

5000 10000 15000 20000 25000 30000 35000 40000 45000 50000 55000 60000 65000 70000 75000 80000 85000 90000 95000 100000 105000 110000
R
PROKKA_01152021.gbk (no annotations loaded)
5000 10000 15000 20000 25000 30000 35000 40000 45000 50000 55000 60000 65000 70000 75000 80000 85000 90000
R
MG648909.fasta

E.

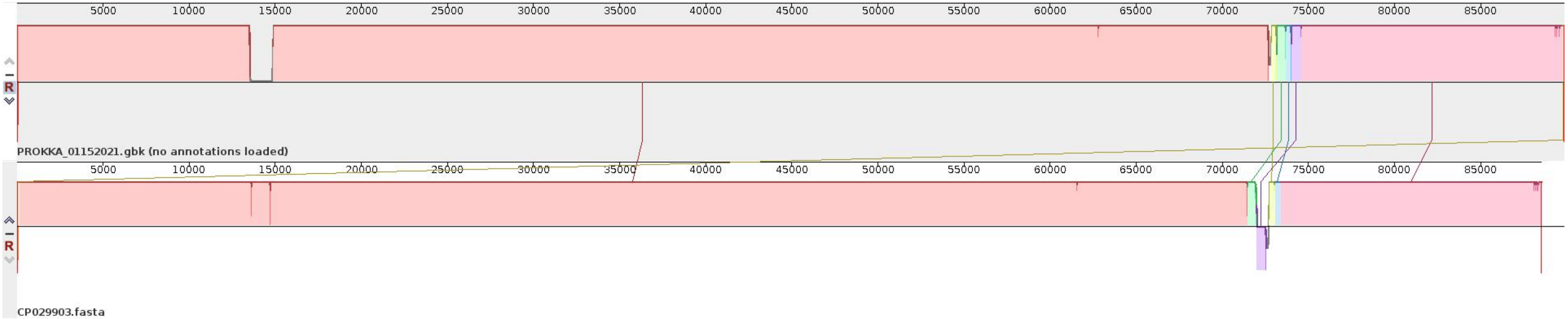
5000 10000 15000 20000 25000 30000 35000 40000 45000 50000 55000 60000 65000 70000 75000 80000 85000
PROKKA_01152021.gbk (no annotations loaded)
5000 10000 15000 20000 25000 30000 35000 40000 45000 50000 55000 60000 65000 70000 75000 80000 85000
CP029903.fasta

F.

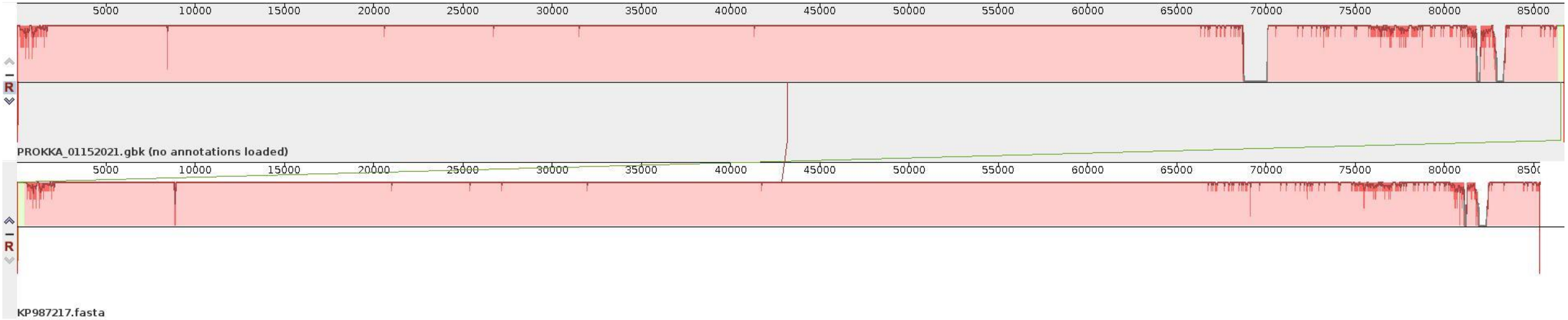
5000 10000 15000 20000 25000 30000 35000 40000 45000 50000 55000 60000 65000 70000 75000 80000 85000
PROKKA_01152021.gbk (no annotations loaded)
5000 10000 15000 20000 25000 30000 35000 40000 45000 50000 55000 60000 65000 70000 75000 80000
KP987217.fasta

G.

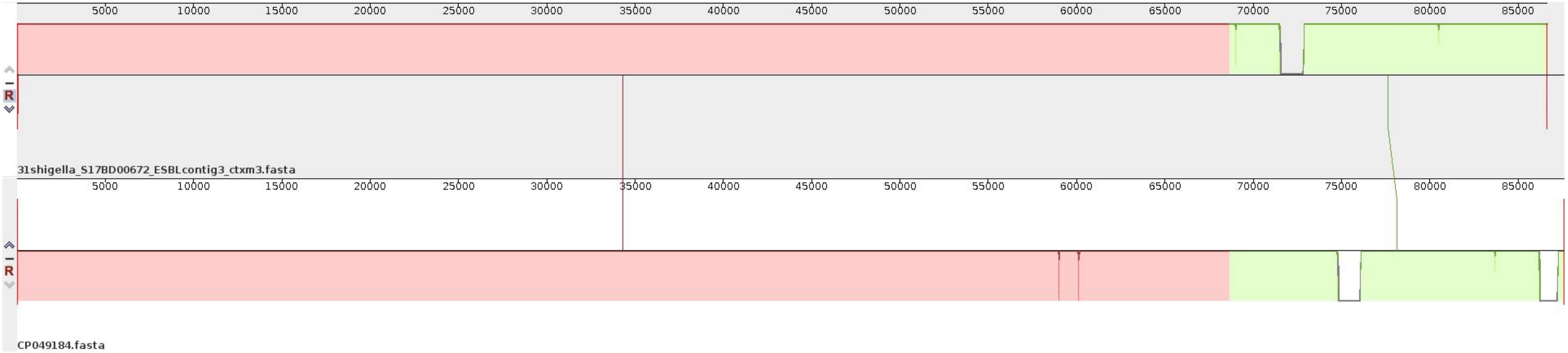

31shigella_S17BD00672_ESBLcontig3_ctxm3.fasta
CP049184.fasta

H.

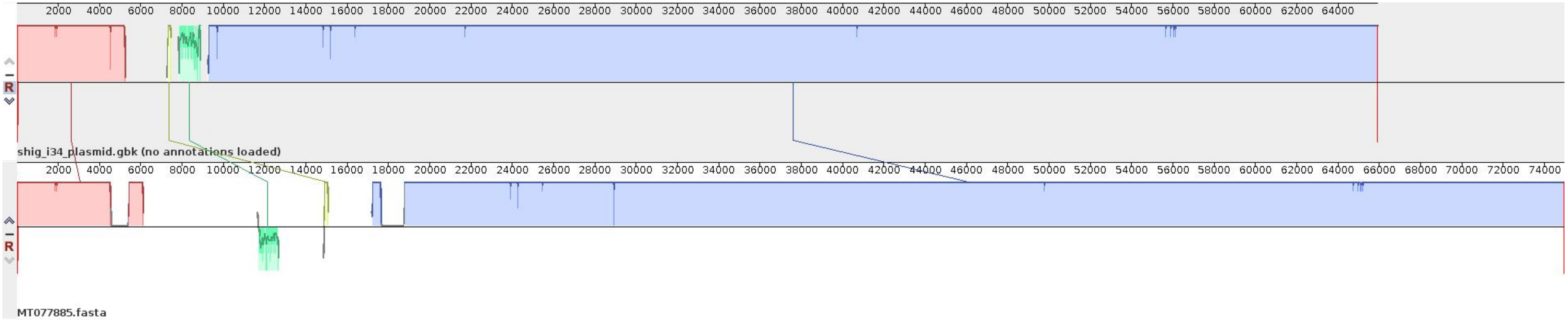

shig_i34_plasmid.gbk (no annotations loaded)
MT077885.fasta

I.

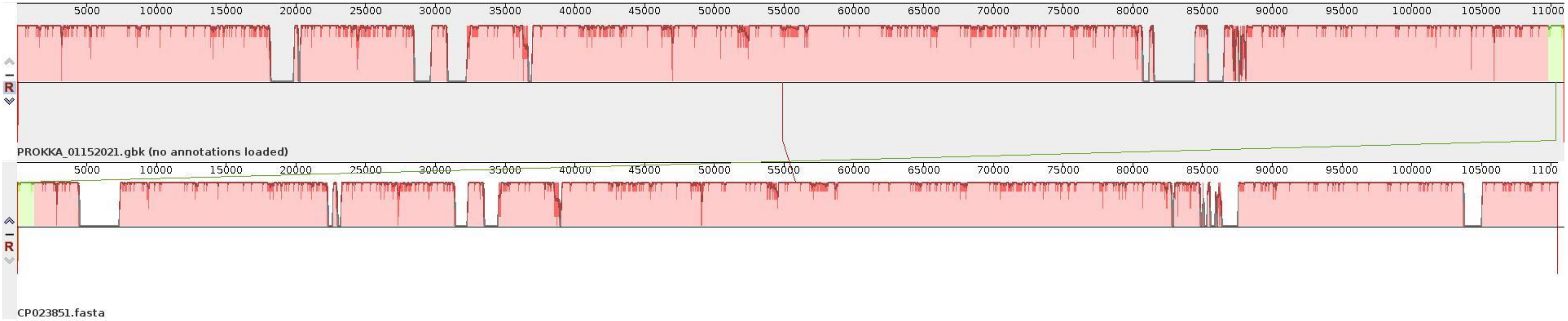

5000 10000 15000 20000 25000 30000 35000 40000 45000 50000 55000 60000 65000 70000 75000 80000 85000 90000 95000 100000 105000 11000
PROKKA_01152021.gbk (no annotations loaded)
5000 10000 15000 20000 25000 30000 35000 40000 45000 50000 55000 60000 65000 70000 75000 80000 85000 90000 95000 100000 105000 1100
CP023851.fasta

J.

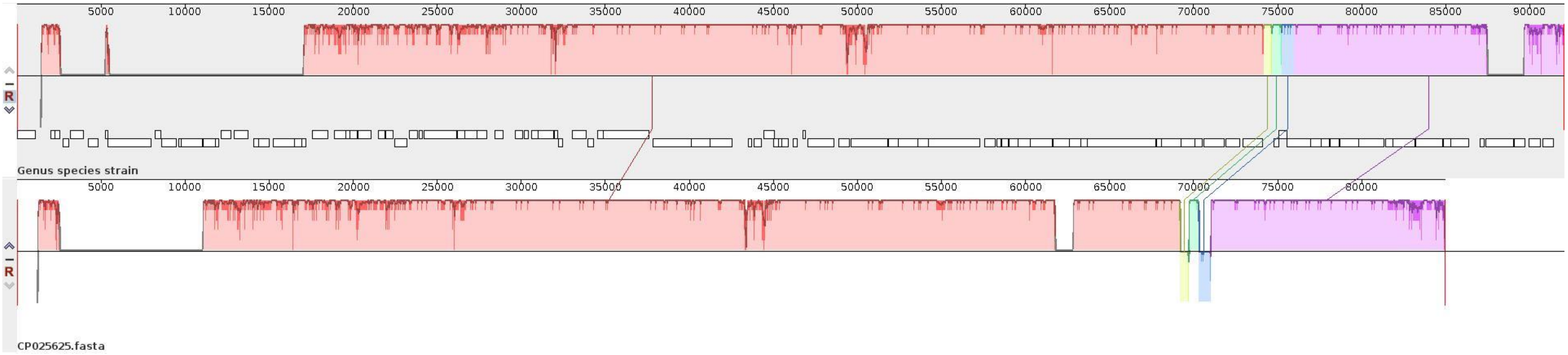
5000 10000 15000 20000 25000 30000 35000 40000 45000 50000 55000 60000 65000 70000 75000 80000 85000 90000
R
Genus species strain
5000 10000 15000 20000 25000 30000 35000 40000 45000 50000 55000 60000 65000 70000 75000 80000
R
CP025625.fasta

K.

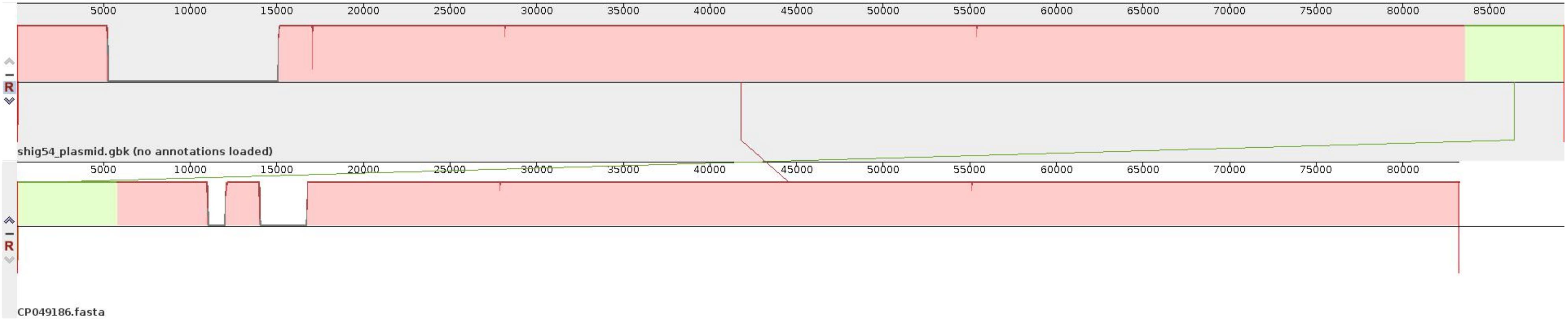
5000 10000 15000 20000 25000 30000 35000 40000 45000 50000 55000 60000 65000 70000 75000 80000 85000
R
shig54_plasmid.gbk (no annotations loaded)
5000 10000 15000 20000 25000 30000 35000 40000 45000 50000 55000 60000 65000 70000 75000 80000
R
CP049186.fasta

L.

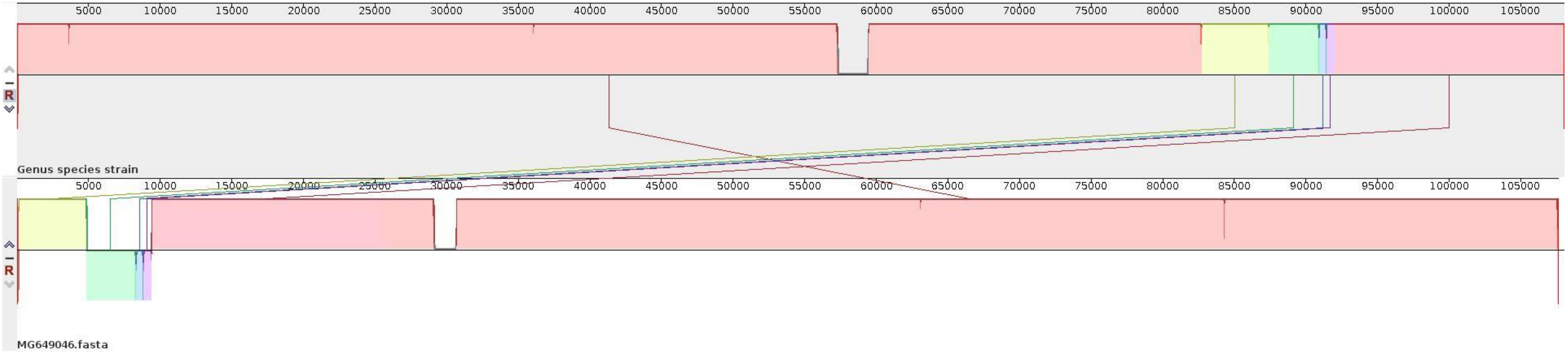
5000 10000 15000 20000 25000 30000 35000 40000 45000 50000 55000 60000 65000 70000 75000 80000 85000 90000 95000 100000 105000
Genus species strain
5000 10000 15000 20000 25000 30000 35000 40000 45000 50000 55000 60000 65000 70000 75000 80000 85000 90000 95000 100000 105000
MG649046.fasta

M.

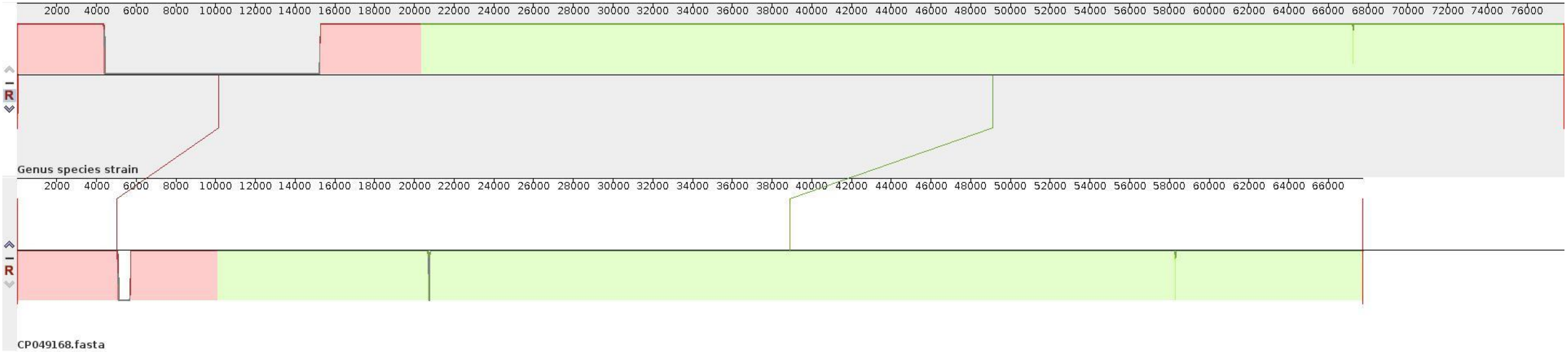
2000 4000 6000 8000 10000 12000 14000 16000 18000 20000 22000 24000 26000 28000 30000 32000 34000 36000 38000 40000 42000 44000 46000 48000 50000 52000 54000 56000 58000 60000 62000 64000 66000 68000 70000 72000 74000 76000
Genus species strain
2000 4000 6000 8000 10000 12000 14000 16000 18000 20000 22000 24000 26000 28000 30000 32000 34000 36000 38000 40000 42000 44000 46000 48000 50000 52000 54000 56000 58000 60000 62000 64000 66000
CP049168.fasta

N.

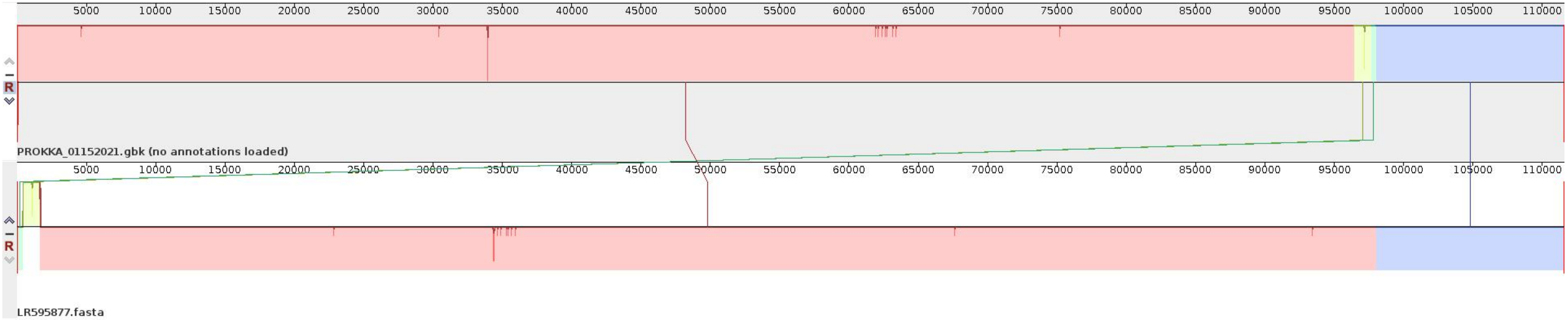
PROKKA_01152021.gbk (no annotations loaded)
LR595877.fasta

O.

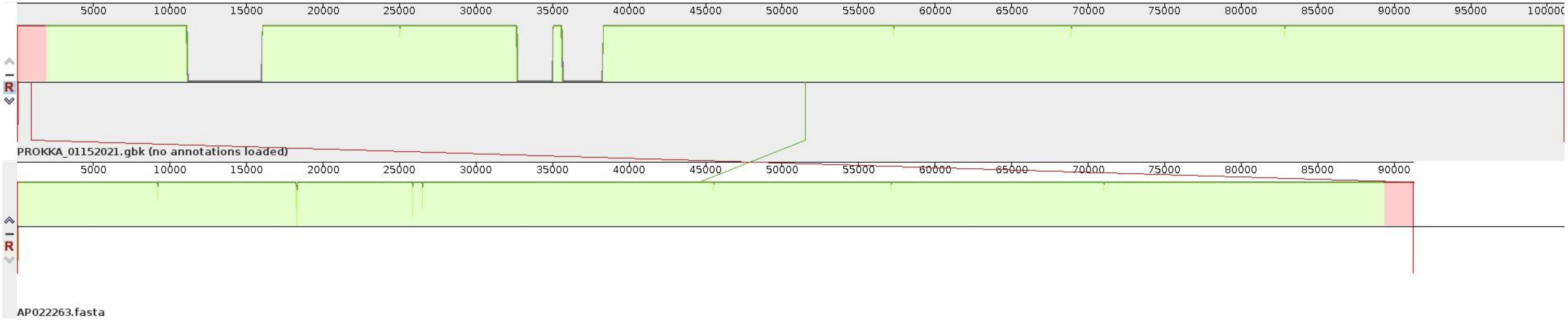
PROKKA_01152021.gbk (no annotations loaded)
AP022263.fasta

P.

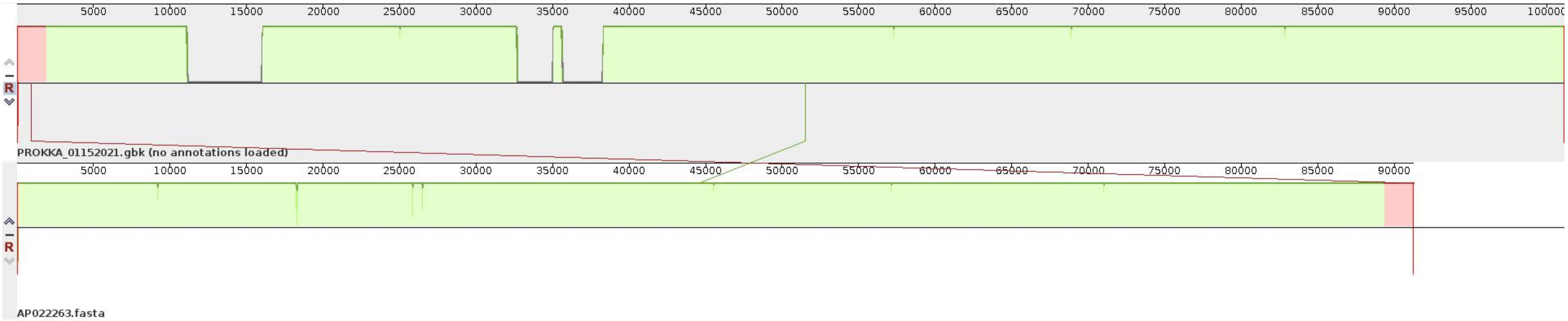
5000 10000 15000 20000 25000 30000 35000 40000 45000 50000 55000 60000 65000 70000 75000 80000 85000 90000 95000 100000
PROKKA_01152021.gbk (no annotations loaded)
5000 10000 15000 20000 25000 30000 35000 40000 45000 50000 55000 60000 65000 70000 75000 80000 85000 90000
AP022263.fasta

Q.

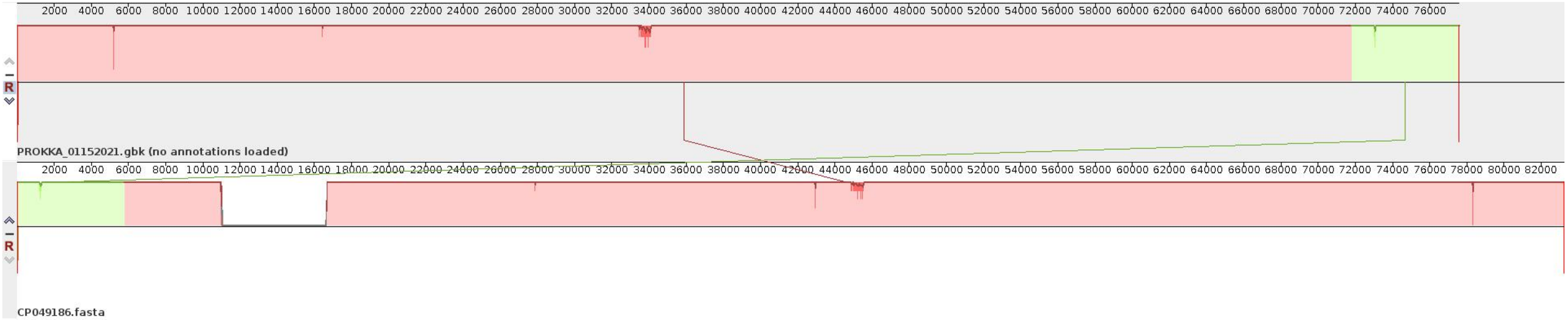
2000 4000 6000 8000 10000 12000 14000 16000 18000 20000 22000 24000 26000 28000 30000 32000 34000 36000 38000 40000 42000 44000 46000 48000 50000 52000 54000 56000 58000 60000 62000 64000 66000 68000 70000 72000 74000 76000
PROKKA_01152021.gbk (no annotations loaded)
2000 4000 6000 8000 10000 12000 14000 16000 18000 20000 22000 24000 26000 28000 30000 32000 34000 36000 38000 40000 42000 44000 46000 48000 50000 52000 54000 56000 58000 60000 62000 64000 66000 68000 70000 72000 74000 76000 78000 80000 82000
CP049186.fasta

R.

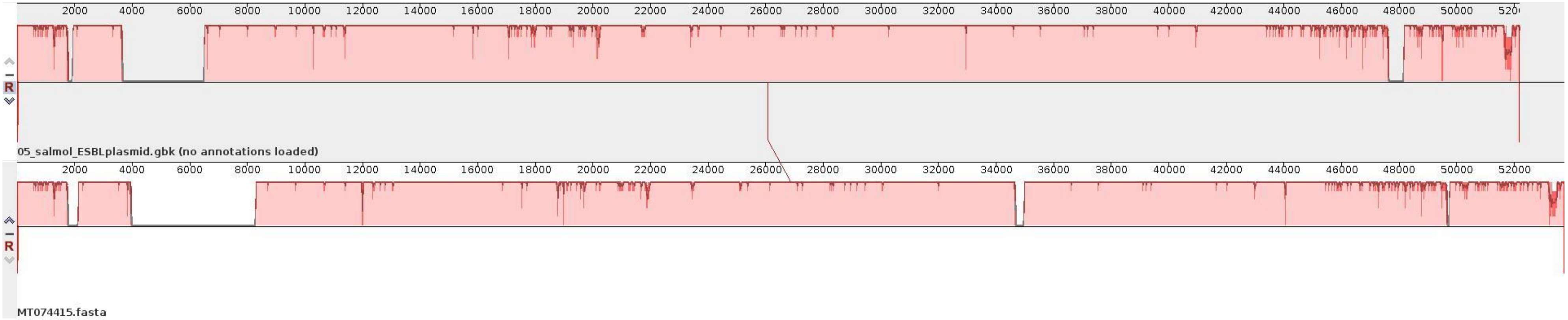
2000 4000 6000 8000 10000 12000 14000 16000 18000 20000 22000 24000 26000 28000 30000 32000 34000 36000 38000 40000 42000 44000 46000 48000 50000 52000
R
05_salmol_ESBLplasmid.gbk (no annotations loaded)
R
MT074415.fasta

S.

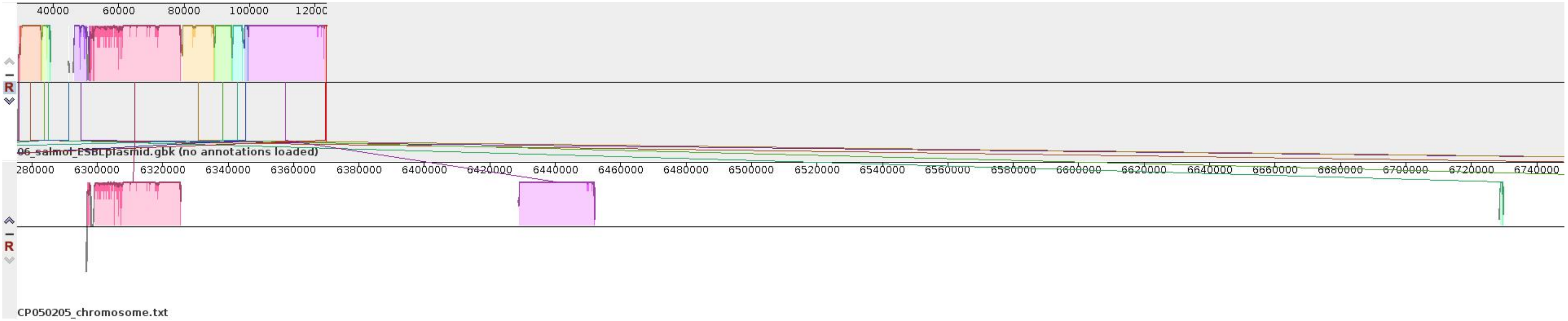
40000 60000 80000 100000
R
06_salmol_ESBLplasmid.gbk (no annotations loaded)
6300000 6320000 6340000 6360000 6380000 6400000 6420000 6440000 6460000 6480000 6500000 6520000 6540000 6560000 6580000 6600000 6620000 6640000 6660000 6680000 6700000 6720000 6740000
R
CP050205_chromosome.txt

T.

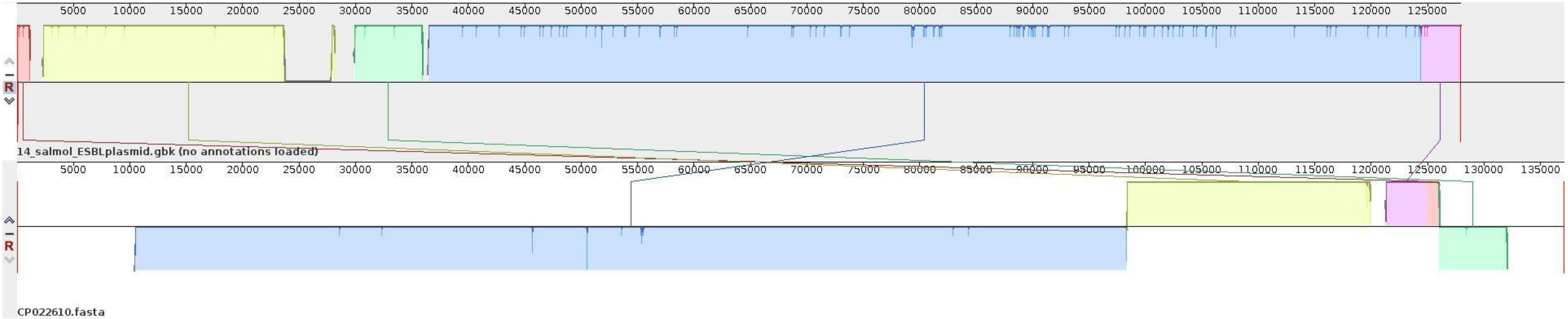
14_salmol_ESBLplasmid.gbk (no annotations loaded)
CP022610.fasta

U.

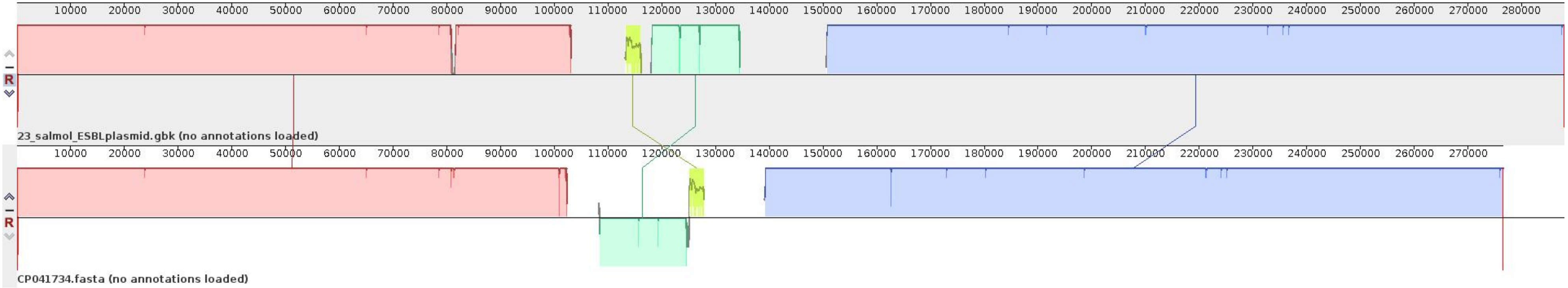
23_salmol_ESBLplasmid.gbk (no annotations loaded)
CP041734.fasta (no annotations loaded)

V.

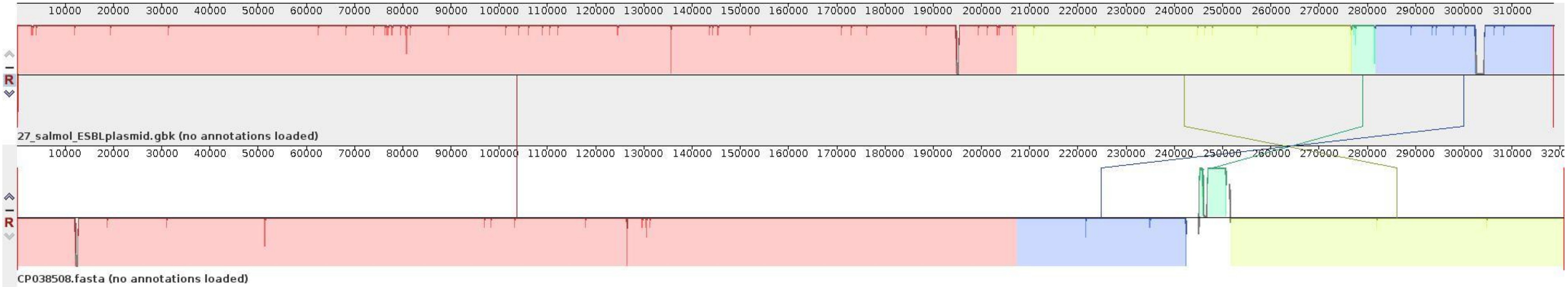

W.

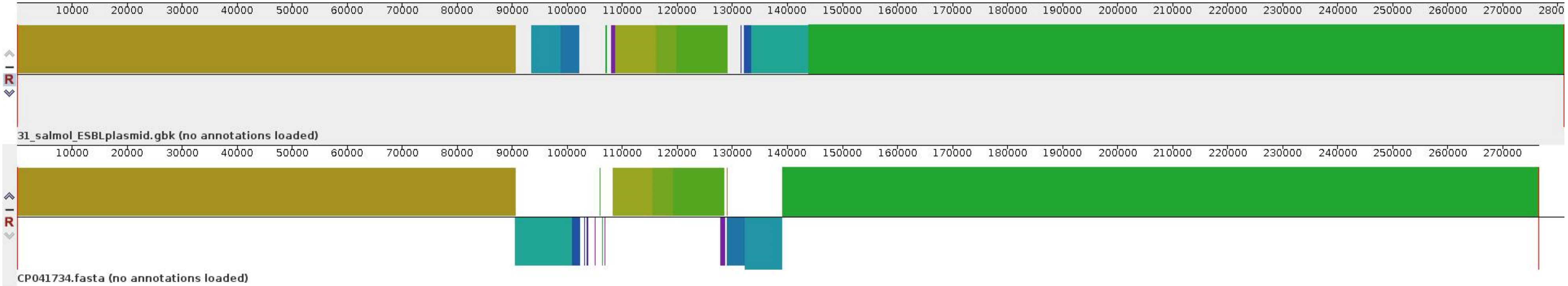

Figure S7:Global alignments between p3 (A.), p4 (B.), p5 (C.) p9 (D.), p12 (E.), p20 (F.), p31 (G.), p34 (H.), p38 (I.), p39 (J.), p54 (K.), p55 (L.), p59 (M.), p67 (N.), p69 (O.), p73 (P.), p83 (Q.), p105 (R.), p106 (S.), p114 (T.), p123 (U.), p127 (V.), p131 (W.) and their top blast hits The colours indicate locally collinear blocks (LCBs), which are homologous regions without rearrangements between two or more sequences.

Table S14: blast hits 2nd to 10th for p3

Blast hits	Description	Max Score	Total Score	Query Cover	Identity (%)	Accession
2	Escherichia coli strain 130SW plasmid p130SW, complete sequence	1.51E+05	1.84E+05	99%	99.95	MK764027.1
3	Escherichia coli strain 105CF plasmid p105CF, complete sequence	1.51E+05	1.84E.+05	99%	99.95	MK764025.1
4	Escherichia coli strain 116DF2 plasmid p116DF2, complete sequence	92067	1.84E+05	99%	99.93	MK764029.1
5	Escherichia coli strain 74DF1 plasmid p74DF1, complete sequence	88019	1.84E+05	99%	99.95	MK764026.1
6	Escherichia coli strain B3804 plasmid pIFM3804, complete sequence	81102	1.65E+05	92%	99.63	KF787110.1

7	Salmonella enterica subsp. enterica serovar Heidelberg str. SL476 plasmid pSL476_91, complete sequence	63078	1.59E+05	90%	98.57	CP001118.1
8	Salmonella enterica subsp. enterica strain 08-00436 plasmid pSE08-00436-2, complete sequence	62794	92561	51%	98.43	CP020494.1
9	Escherichia coli strain 123DF2 plasmid p123DF2, complete sequence	61808	1.84E+05	99%	99.93	MK764024.1
10	Escherichia coli plasmid pCOV29 clone COV29_c1	59337	1.61E+05	92%	98.39	MG649040.1

Table S15: blast hits 2nd to 10th for p4

Blast hits	Description	Max Score	Total Score	Query Cover	Identity (%)	Accession
2	Escherichia coli HUSEC2011 plasmid pHUSEC2011-1 complete sequence	95053	1.47E+05	88%	98.49	HE610900.2
3	Escherichia coli plasmid pV150-a DNA, contig: V150-a_scaffold_1, strain: V150	94106	1.15E+05	67%	98.49	LC056403.1
4	Escherichia coli strain B4-75 genome assembly, plasmid: RCS80_p	94097	1.45E+05	87%	98.48	LT985289.1
5	Escherichia coli strain FDAARGOS_403 plasmid unnamed1, complete sequence	94095	1.81E+05	88%	98.48	CP023534.1
6	Escherichia coli genome assembly FHI102, scaffold scaffold-17_contig-12.0_1_88561_[organism:Escherichia	94089	1.46E+05	86%	98.49	LM995498.1
7	Escherichia coli strain RHBSTW-00321 plasmid pRHBSTW-00321_3, complete sequence	93940	1.56E+05	89%	98.43	CP056606.1
8	Escherichia coli strain MVAST0167 plasmid pMVAST0167_2, complete sequence	93570	1.36E+05	82%	98.32	CP014494.1
9	Salmonella enterica subsp. enterica serovar Enteritidis strain 69-3861 plasmid pSE69-3861-1, complete sequence	93482	1.41E+05	85%	98.29	CP018638.1
10	Escherichia coli O10:H32 strain NMBU-W12E19 plasmid pNMBU-W12E19_02, complete sequence	89855	1.41E+05	85%	98.44	CP042886.1

Table S16: blast hits 2nd to 10th for p5

Blast hits	Description	Max Score	Total Score	Query Cover	Identity (%)	Accession
2	Escherichia coli strain ET20160881 plasmid pET20160881, complete sequence	1.36E+05	1.75E+05	96%	99.98	MF078004.1
3	Escherichia coli strain TW10590 plasmid pTW10590_71, complete sequence	1.01E+05	1.28E+05	73%	99.82	CP035854.1
4	Escherichia coli O169:H41 strain 2014EL-1345-2 plasmid unnamed3	95164	1.62E+05	90%	99.98	CP024226.1
5	Escherichia coli O25:NM strain 2014EL-1343-2 plasmid unnamed2	95157	1.39E+05	76%	99.97	CP024230.1
6	Escherichia coli strain RHBSTW-00321 plasmid pRHBSTW-00321_3, complete sequence	67558	1.52E+05	82%	99.44	CP056606.1
7	Escherichia coli strain RM-065-CS chromosome	60131	2.08E+05	98%	99.99	CP050211.1

8	Escherichia coli strain 266917_2 plasmid p266917_2_03, complete sequence	58959	1.11E+05	64%	98.52	CP026726.1
9	Escherichia coli strain Ec42 plasmid pEc42_2, complete sequence	58783	1.11E+05	65%	98.43	CP059121.1
10	Escherichia coli strain EC2341 plasmid pEC2341_3, complete sequence	58743	1.14E+05	65%	98.41	CP072978.1

Table S17: blast hits 2nd to 10th for p9

Blast hits	Description	Max Score	Total Score	Query Cover	Identity (%)	Accession
2	Escherichia coli DH5alpha plasmid p386-1 contig Covetlab39_c1 genomic sequence	1.61E+05	1.75E+05	82%	100	MG692614.1
3	Escherichia coli plasmid pCOV15 clone COV15_c1	1.52E+05	2.03E+05	95%	100	MG648932.1
4	Escherichia coli strain AR_0081 plasmid unnamed1, complete sequence	1.23E+05	1.79E+05	84%	99.49	CP027535.1
5	Escherichia coli isolate H5 genome assembly, plasmid: pZPK-H5	1.13E+05	1.78E+05	83%	99.79	LR999862.1
6	Escherichia coli plasmid pCOV24 clone COV24	1.12E+05	2.46E+05	100%	99.97	MG648999.1
7	Escherichia coli strain 2016-40-22638 plasmid p22638, complete sequence	1.06E+05	1.97E+05	93%	99.97	MN419437.1
8	Escherichia coli strain J53 plasmid pCTX-M-1.A, complete sequence	1.02E+05	2.32E+05	94%	99.86	MW978788.1
9	Escherichia coli strain RHBSTW-00177 plasmid pRHBSTW-00177_4, complete sequence	95162	1.55E+05	74%	99.42	CP056797.1
10	Escherichia coli strain Res13-Lact-ER02-39 plasmid unnamed476, complete sequence	91916	1.92E+05	90%	100	CP062899.1

Table S18: blast hits 2nd to 10th for p12

Blast hits	Description	Max Score	Total Score	Query Cover	Identity (%)	Accession
2	Escherichia coli strain B4-75 genome assembly, plasmid: RCS80_p	1.07E+05	1.65E+05	97%	99.98	LT985289.1
3	Escherichia coli HUSEC2011 plasmid pHUSEC2011-1 complete sequence	1.07E+05	1.67E+05	98%	99.98	HE610900.2
4	Escherichia coli strain FDAARGOS_403 plasmid unnamed1, complete sequence	1.07E+05	2.05E+05	98%	99.98	CP023534.1
5	Escherichia coli plasmid pV150-a DNA, contig: V150-a_scaffold_1, strain: V150	1.07E+05	1.23E+05	71%	99.97	LC056403.1
6	Escherichia coli genome assembly FHI102, scaffold scaffold-17_contig-12.0_1_88561_[organism:Escherichia	1.07E+05	1.66E+05	96%	99.97	LM995498.1
7	Escherichia coli strain RHBSTW-00321 plasmid pRHBSTW-00321_3, complete sequence	1.06E+05	1.61E+05	94%	99.89	CP056606.1
8	Escherichia coli strain MVAST0167 plasmid pMVAST0167_2, complete sequence	1.06E+05	1.44E+05	86%	99.61	CP014494.1

9	Escherichia coli O10:H32 strain NMBU-W12E19 plasmid pNMBU-W12E19_02, complete sequence	1.02E+05	1.54E+05	91%	99.97	CP042886.1
10	Shigella sonnei strain AR_0030 plasmid unnamed1, complete sequence	92621	1.59E+05	95%	99.07	CP032524.1

Table S19: blast hits 2nd to 10th for p20

Blast hits	Description	Max Score	Total Score	Query Cover	Identity (%)	Accession
2	Escherichia coli strain RD174 plasmid pHNRD174, complete sequence	1.26E+05	1.58E+05	98%	99.78	KX246268.1
3	Enterobacter hormaechei strain RHBSTW-00218 plasmid pRHBSTW-00218_2, complete sequence	1.26E+05	1.58E+05	98%	99.8	CP056650.1
4	Escherichia coli strain L3Cip3 plasmid unnamed2, complete sequence	1.14E+05	1.62E+05	99%	99.96	CP062213.1
5	Escherichia coli strain 266917_2 plasmid p266917_2_03, complete sequence	1.12E+05	1.48E+05	94%	99.19	CP026726.1
6	Escherichia coli strain C plasmid pEK204, complete sequence	1.11E+05	1.58E+05	98%	99.91	EU935740.1
7	Shigella sonnei strain SS084469 plasmid pSH4469, complete sequence	1.11E+05	1.58E+05	98%	99.9	KJ406378.1
8	Escherichia coli strain 1500 plasmid pEc1500_CTX, complete sequence	1.11E+05	1.58E+05	98%	99.9	CP040270.1
9	Klebsiella pneumoniae strain D16KP0008 plasmid pD16KP0008-1, complete sequence	1.11E+05	1.58E+05	98%	99.84	CP052382.1
10	Escherichia coli strain strain Z247 plasmid p2474-3, complete sequence	1.09E+05	1.63E+05	100%	99.95	CP021208.1

Table S20: blast hits 2nd to 10th for p31

Blast hits	Description	Max Score	Total Score	Query Cover	Identity (%)	Accession
2	Shigella sonnei strain LC1477/18 plasmid pLC1477_18-1, complete sequence	1.27E+05	1.59E+05	97%	99.94	CP035009.1
3	Shigella sonnei strain 1205.3131 plasmid p1205-3131, complete sequence	73791	1.57E+05	97%	99.86	CP049178.1
4	Shigella sonnei strain 509.1022 plasmid p509-1022, complete sequence	71963	1.61E+05	98%	100	CP049182.1
5	Shigella sonnei strain 7111.69 plasmid p7111-69, complete sequence	60979	1.60E+05	98%	99.83	CP049176.1
6	Shigella sonnei strain L4094 plasmid pL4094, complete sequence	52211	1.60E+05	98%	99.86	CP049180.1
7	Escherichia coli O6:H16 strain 2014EL-1346-6 plasmid unnamed2	51456	68384	43%	99.51	CP024234.1
8	Escherichia coli strain Ecol_224 plasmid pEC224_2, complete sequence	51313	1.53E+05	95%	99.3	CP018946.1
9	Escherichia coli strain RHB13-C03 plasmid pRHB13-C03_4, complete sequence	51168	1.33E+05	88%	98.82	CP057846.1
10	Escherichia coli strain RHB13-C01 plasmid pRHB13-C01_4, complete sequence	50891	1.34E+05	88%	98.65	CP057857.1

Table S21: blast hits 2nd to 10th for p34

Blast hits	Description	Max Score	Total Score	Query Cover	Identity (%)	Accession
2	Salmonella enterica subsp. enterica serovar Typhimurium strain 81741 plasmid unnamed2, complete sequence	1.04E+05	1.26E+05	94%	99.95	CP019444.1
3	Shigella sonnei strain 893916 plasmid p893916, complete sequence	1.04E+05	1.29E+05	94%	99.95	MW396858.1
4	Escherichia coli O10:H32 strain NMBU-W12E19 plasmid pNMBU-W12E19_03, complete sequence	1.04E+05	1.22E+05	94%	99.93	CP042887.1
5	Escherichia coli strain CJ5423 plasmid pDW36-14 genomic sequence	1.04E+05	1.28E+05	97%	99.93	MK436212.1
6	Shigella sonnei strain 19.0821.3486 plasmid p19-0821-3486, complete sequence	93059	1.35E+05	100%	99.96	CP049186.1
7	Klebsiella pneumoniae plasmid pABC143C-NDM, complete sequence	90996	1.24E+05	92%	99.97	KY130431.1
8	Escherichia coli strain 3347558 plasmid p3347558_4, complete sequence	90976	1.19E+05	94%	99.96	CP071077.1
9	Escherichia coli strain AR202.2 plasmid pMPCMY-2, complete sequence	84585	1.12E+05	90%	99.97	CP043949.1
10	Escherichia coli isolate strain_Ecoli_008 genome assembly, plasmid: pMOL008_IncFII	84585	1.20E+05	94%	99.97	LR880737.1

Table S22: blast hits 2nd to 10th for p38

Blast hits	Description	Max Score	Total Score	Query Cover	Identity (%)	Accession
2	Escherichia coli O18ac:H14 strain 873.10 plasmid unnamed3, complete sequence	76417	1.63E+05	84%	98.25	CP061757.1
3	Escherichia coli O25b:H4-ST131 plasmid p7.2.1, complete sequence	74435	1.81E+05	93%	98.34	MK125034.1
4	Escherichia coli H89 plasmid pECOH89, complete sequence	74428	1.81E+05	93%	98.34	HG530657.1
5	Escherichia coli O25b:H4-ST131 plasmid pB20 clone ST131-Rx_O25b:fimH30, complete sequence	74422	1.81E+05	93%	98.34	MK125035.1
6	Escherichia coli strain RHBSTW-00849 plasmid pRHBSTW-00849_2, complete sequence	74147	1.67E+05	88%	97.37	CP056300.1
7	Escherichia coli strain 503829 plasmid p503829_100, complete sequence	68024	1.40E+05	75%	98.06	CP025870.1
8	Escherichia coli strain 503025 plasmid p503025_100, complete sequence	68024	1.40E+05	75%	98.06	CP025894.1
9	Escherichia coli SP15 plasmid SP15_P2 DNA, complete sequence	66096	1.75E+05	90%	98.51	AP024133.2
10	Escherichia coli strain 3f3 plasmid pERB3f3, complete sequence	65743	1.94E+05	96%	99.29	MW590712.1

Table S23: blast hits 2nd to 10th for p39

Blast hits	Description	Max Score	Total Score	Query Cover	Identity (%)	Accession
2	Escherichia coli strain 0126:B16 plasmid R16, complete sequence	74458	1.42E+05	85%	99.55	MK758104.1
3	Salmonella enterica subsp. enterica strain H-185 plasmid R805a, complete sequence	74458	1.42E+05	85%	99.55	MK088173.1
4	Escherichia coli strain MS7163 plasmid pMS7163B, complete sequence	63970	1.32E+05	80%	99.69	CP026855.1
5	Escherichia coli NCCP15648 plasmid p15648-2, complete sequence	61673	1.47E+05	87%	98.12	CP009052.1
6	Escherichia coli strain 3-S1R plasmid pCERC10, complete sequence	61324	1.44E+05	86%	97.92	MF156268.1
7	Escherichia coli strain TCBK21G178 plasmid unnamed, complete sequence	60859	1.57E+05	85%	98.89	CP048936.1
8	Shigella sonnei strain 893916 plasmid unnamed, complete sequence	60270	1.50E+05	90%	98.06	MW396864.1
9	Escherichia coli isolate MINF_8D-sc-2280460 genome assembly, plasmid: 4	58584	1.36E+05	82%	97.71	LR890609.1
10	Escherichia coli strain FDAARGOS_1300 plasmid unnamed3, complete sequence	57891	1.42E+05	85%	99.74	CP069999.1

Table S24: blast hits 2nd to 10th for p54

Blast hits	Description	Max Score	Total Score	Query Cover	Identity (%)	Accession
2	Escherichia coli strain NMBU-W13E19 plasmid pNMBU-W13E19_02, complete sequence	1.13E+05	1.34E+05	76%	99.7	CP043408.1
3	Shigella sonnei strain 6904.27 plasmid p6904-27	1.09E+05	1.64E+05	88%	99.99	CP045525.2
4	Shigella sonnei strain 893916 plasmid p893916, complete sequence	1.07E+05	1.56E+05	83%	99.98	MW396858.1
5	Escherichia coli isolate BB1426 genome assembly, plasmid: pBB1426_IncFII	1.07E+05	1.39E+05	75%	99.95	LR701157.1
6	Salmonella enterica subsp. enterica serovar Derby strain 75 plasmid, partial sequence	1.07E+05	1.27E+05	71%	99.94	MK191836.1
7	Klebsiella pneumoniae strain FDAARGOS_1325 plasmid unnamed2, complete sequence	1.07E+05	1.33E+05	71%	99.94	CP069948.1
8	Escherichia coli strain CJ5423 plasmid pDW36-14 genomic sequence	1.07E+05	1.36E+05	74%	99.95	MK436212.1
9	Escherichia coli O10:H32 strain NMBU-W12E19 plasmid pNMBU-W12E19_03, complete sequence	1.07E+05	1.36E+05	77%	99.93	CP042887.1
10	Escherichia coli plasmid p37, complete sequence	1.07E+05	1.43E+05	72%	99.93	MT077885.1

Table S25: blast hits 2nd to 10th for p55

Blast hits	Description	Max Score	Total Score	Query Cover	Identity (%)	Accession
2	Escherichia coli strain DH5alpha plasmid pESBL112, complete sequence	99048	2.00E+05	98%	99.99	MT230105.1
3	Escherichia coli plasmid pCOV32 clone COV32_c1	99042	2.00E+05	98%	99.99	MG649045.1
4	Escherichia coli strain DH5alpha plasmid pESBL243, complete sequence	99038	1.93E+05	94%	99.99	MT230229.1
5	Escherichia coli strain DH5alpha plasmid pESBL26, complete sequence	99036	1.99E+05	98%	99.99	MT230257.1
6	Escherichia coli plasmid pCOV6 clone COV6_c1	99036	1.24E+05	60%	99.99	MG648861.1
7	Escherichia coli plasmid pCOV7 clone COV7_c1	99031	1.97E+05	97%	99.99	MG648892.1
8	Escherichia coli plasmid pCOV2 clone COV2_c1	99031	1.69E+05	82%	99.98	MG648842.1
9	Escherichia coli DH5alpha plasmid p369-6 contig COV38TF1_c1 genomic sequence	99029	2.00E+05	98%	99.99	MG692636.1
10	Escherichia coli strain 369 plasmid p369, complete sequence	99029	2.00E+05	98%	99.99	KT779550.1

Table S26: blast hits 2nd to 10th for p59

Blast hits	Description	Max Score	Total Score	Query Cover	Identity (%)	Accession
2	Shigella sonnei strain 0401930105 plasmid p0401930105	1.12E+05	1.64E+05	88%	100	CP049172.1
3	Shigella sonnei strain 6904.27 plasmid p6904-27	1.09E+05	1.65E+05	88%	99.99	CP045525.2
4	Klebsiella pneumoniae strain KP1814 plasmid pKP1814-3, complete sequence	97827	1.40E+05	79%	99.56	KX839209.1
5	Shigella sonnei strain 19.0820.1561 plasmid p19-0820-1561, complete sequence	94529	1.81E+05	100%	99.99	CP049174.1
6	Escherichia coli strain NMBU-W13E19 plasmid pNMBU-W13E19_02, complete sequence	93631	1.35E+05	76%	99.67	CP043408.1
7	Escherichia coli strain 637 genome assembly, plasmid: RCS61_p	87851	1.49E+05	76%	99.97	LT985267.1
8	Klebsiella pneumoniae strain 4743 plasmid unnamed4, complete sequence	87846	1.37E+05	75%	99.98	CP033629.1
9	Klebsiella pneumoniae MH15-269M plasmid pMH15-269M_4 DNA, complete sequence	87840	1.42E+05	75%	99.99	AP023341.1
10	Salmonella enterica subsp. enterica serovar Derby strain 75 plasmid, partial sequence	87814	1.27E+05	71%	99.98	MK191836.1

Table S27: blast hits 2nd to 10th for p67

Blast hits	Description	Max Score	Total Score	Query Cover	Identity (%)	Accession
2	Klebsiella pneumoniae plasmid pABC143C-NDM, complete sequence	59731	1.52E+05	90%	99.99	KY130431.1
3	Salmonella enterica subsp. enterica serovar Typhimurium strain 81741 plasmid unnamed2, complete sequence	59731	1.45E+05	89%	99.99	CP019444.1
4	Escherichia coli plasmid p37, complete sequence	59725	1.60E+05	91%	99.99	MT077885.1
5	Escherichia coli plasmid pV300-b DNA, contig: V300-b_scaffold_1, strain: V300	59690	77242	54%	99.97	LC056580.1
6	Escherichia coli strain 3347558 plasmid p3347558_4, complete sequence	59690	1.24E+05	83%	99.97	CP071077.1
7	Escherichia coli plasmid pV318-b DNA, contig: V318-b_scaffold_1, strain: V318	59681	78426	54%	99.95	LC056626.1
8	Escherichia coli O10:H32 strain NMBU-W12E19 plasmid pNMBU-W12E19_03, complete sequence	59673	1.38E+05	88%	99.96	CP042887.1
9	Klebsiella pneumoniae strain MRK9 plasmid unnamed4	59673	1.23E+05	84%	99.97	CP043863.1
10	Escherichia coli strain AR202.2 plasmid pMPCMY-2, complete sequence	59668	1.20E+05	82%	99.96	CP043949.1

Table S28: blast hits 2nd to 10th for p69

Blast hits	Description	Max Score	Total Score	Query Cover	Identity (%)	Accession
2	Klebsiella pneumoniae strain JX-CR-hvKP-1 plasmid pJX1-2, complete sequence	1.29E+05	2.08E+05	93%	100	CP064254.1
3	Shigella sonnei strain SH287-2 plasmid pSH287-2, complete sequence	1.17E+05	2.21E+05	99%	99.99	MG299151.1
4	Shigella sonnei strain SH284-2 plasmid pSH284-2, complete sequence	1.17E+05	2.21E+05	99%	99.98	MG299147.1
5	Shigella sonnei strain SH272-2 plasmid pSH272-2, complete sequence	1.17E+05	2.21E+05	99%	99.98	MG299133.1
6	Shigella sonnei strain SH262-2 plasmid pSH262-2, complete sequence	1.17E+05	2.21E+05	99%	99.98	MG299128.1
7	Shigella sonnei strain SH15sh99 plasmid pSH15sh99, complete sequence	1.17E+05	2.11E+05	94%	99.98	KY471628.1
8	Shigella sonnei strain SH271-2 plasmid pSH271-2, complete sequence	1.16E+05	2.21E+05	99%	100	MG299131.1
9	Shigella sonnei strain SH15sh105 plasmid pSH15sh104, complete sequence	1.16E+05	2.11E+05	94%	99.99	KY471629.1
10	Escherichia coli strain VRES-hospital6495171 genome assembly, plasmid: 1	1.03E+05	1.66E+05	80%	99.42	LR595872.1

Table S29: blast hits 2nd to 10th for p73

Blast hits	Description	Max Score	Total Score	Query Cover	Identity (%)	Accession
2	Escherichia coli strain ET20160881 plasmid pET20160881, complete sequence	1.16E+05	1.75E+05	91%	99.99	MF078004.1
3	Escherichia coli strain TW10590 plasmid pTW10590_71, complete sequence	1.01E+05	1.28E+05	69%	99.82	CP035854.1
4	Escherichia coli O169:H41 strain 2014EL-1345-2 plasmid unnamed3	95158	1.62E+05	85%	99.98	CP024226.1

5	Escherichia coli O25:NM strain 2014EL-1343-2 plasmid unnamed2	95151	1.39E+05	73%	99.97	CP024230.1
6	Escherichia coli strain RM-065-CS chromosome	60126	2.08E+05	93%	99.99	CP050211.1
7	Escherichia coli strain 266917_2 plasmid p266917_2_03, complete sequence	58953	1.11E+05	61%	98.52	CP026726.1
8	Escherichia coli strain SCAID WND2-2021 (3/145) plasmid unnamed2, complete sequence	58796	1.31E+05	61%	98.44	CP082829.1
9	Escherichia coli strain Ec42 plasmid pEc42_2, complete sequence	58778	1.11E+05	62%	98.43	CP059121.1
10	Escherichia coli strain EC2341 plasmid pEC2341_3, complete sequence	58737	1.14E+05	61%	98.41	CP072978.1

Table S30: blast hits 2nd to 10th for p83

Blast hits	Description	Max Score	Total Score	Query Cover	Identity (%)	Accession
2	Escherichia coli strain NMBU-W13E19 plasmid pNMBU-W13E19_02, complete sequence	1.13E+05	1.31E+05	87%	99.64	CP043408.1
3	Shigella sonnei strain 6904.27 plasmid p6904-27	1.09E+05	1.58E+05	100%	99.94	CP045525.2
4	Shigella sonnei strain 893916 plasmid p893916, complete sequence	1.07E+05	1.32E+05	82%	99.9	MW396858.1
5	Escherichia coli strain CJ5423 plasmid pDW36-14 genomic sequence	1.07E+05	1.31E+05	84%	99.89	MK436212.1
6	Escherichia coli O10:H32 strain NMBU-W12E19 plasmid pNMBU-W12E19_03, complete sequence	1.07E+05	1.25E+05	82%	99.87	CP042887.1
7	Escherichia coli plasmid p37, complete sequence	1.07E+05	1.35E+05	82%	99.87	MT077885.1
8	Escherichia coli isolate BB1426 genome assembly, plasmid: pBB1426_IncFII	1.07E+05	1.27E+05	81%	99.87	LR701157.1
9	Salmonella enterica subsp. enterica serovar Typhimurium strain 81741 plasmid unnamed2, complete sequence	1.04E+05	1.27E+05	81%	99.87	CP019444.1
10	Shigella sonnei strain 0401930105 plasmid p0401930105	92448	1.58E+05	100%	99.93	CP049172.1

Table S31: blast hits 2nd to 10th for p105

Blast hits	Description	Max Score	Total Score	Query Cover	Identity (%)	Accession
2	Escherichia coli O157:H7 strain Z1834 plasmid pZ1834-2, complete sequence	39659	85719	88%	98.23	CP062704.1
3	Escherichia coli strain SJ7 plasmid pSJ7-2	36721	85777	88%	98.15	CP051658.1
4	Escherichia coli isolate L5_E1779_ETEC genome assembly, plasmid: 6	30854	1.06E+05	90%	98.09	LR883011.1
5	Escherichia coli strain TA321 / Oneg:H31 / fimH31 / 6968 (ST Warwick) genome assembly, chromosome: 1	24432	59711	74%	95.96	OU349846.1
6	Escherichia coli plasmid pV085-c DNA, complete sequence, strain: V085	22107	65303	73%	97.8	AP014877.1

7	Escherichia coli strain FDAARGOS_497 plasmid unnamed1, complete sequence	21416	56018	69%	93.06	CP033847.1
8	Escherichia coli strain Ecol_881 plasmid pEC881_1, complete sequence	21416	56018	69%	93.06	CP019028.1
9	Escherichia coli strain 127 plasmid p123, complete sequence	21403	56937	70%	93.03	CP023378.1
10	Klebsiella pneumoniae strain ST11 plasmid pKP12226, complete sequence	21403	60263	72%	93.03	KP453775.1

Table S32: blast hits 2nd to 10th for p106

Blast hits	Description	Max Score	Total Score	Query Cover	Identity (%)	Accession
2	Escherichia coli isolate Escherichia coli str. 3426 genome assembly, plasmid: 2	34856	1.56E+05	57%	100	LS992186.1
3	Escherichia coli strain FDAARGOS_1252 plasmid unnamed3	33216	1.10E+05	43%	96.56	CP069531.1
4	Escherichia coli 042 plasmid pAA complete sequence	32860	1.05E+05	45%	98.24	FN554767.1
5	Escherichia coli strain E-1246 plasmid pE1246_1, complete sequence	30782	1.34E+05	61%	99.49	CP025574.1
6	Escherichia coli plasmid pBEC1-S17-ESBL-09_1 DNA, complete genome, strain: BEC1-S17-ESBL-09	30782	1.22E+05	55%	99.47	AP022299.1
7	Escherichia coli strain pAA-ST131 plasmid ESBL20150001, complete sequence	30758	1.31E+05	57%	99.45	KY706108.1
8	Escherichia coli plasmid pV158-a DNA, contig: V158-a_scaffold_1, strain: V158	30269	79069	32%	99.63	LC056409.1
9	Escherichia coli O8:H28 strain BMH-17-0027 plasmid unnamed, complete sequence	29591	85448	44%	95.5	CP076705.1
10	Escherichia coli strain 14EC047 plasmid p14EC047b, complete sequence	29375	1.07E+05	48%	95.56	CP024157.1

Table S33: blast hits 2nd to 10th for p114

Blast hits	Description	Max Score	Total Score	Query Cover	Identity (%)	Accession
2	Escherichia coli strain FAM21845 plasmid pFAM21845_1, complete sequence	1.62E+05	2.33E+05	89%	99.94	CP017221.1
3	Salmonella enterica subsp. enterica serovar Albert strain AR-0401 plasmid pAR-0401-2, complete sequence	1.59E+05	2.38E+05	95%	99.96	CP044190.1
4	Salmonella enterica subsp. enterica serovar Typhimurium strain CVM 24350 plasmid p24350-2, complete sequence	1.38E+05	2.39E+05	95%	99.96	CP051388.1
5	Salmonella enterica subsp. enterica serovar Worthington strain CVM 33836 plasmid p33836-2, complete sequence	1.13E+05	2.60E+05	93%	99.95	CP051335.1
6	Escherichia coli strain CFS3273 plasmid pCFS3273-2, complete sequence	1.11E+05	2.50E+05	96%	99.95	CP026934.1
7	Escherichia coli O39:H21 strain Res13-Lact-PEB08-01 plasmid unnamed972, complete sequence	97179	1.95E+05	71%	99.84	CP062867.1

8	Escherichia coli strain Res13-Lact-PEB09-33 plasmid unnamed972, complete sequence	97147	1.98E+05	71%	99.83	CP066202.1
9	Salmonella enterica subsp. enterica serovar Heidelberg plasmid pSH163_120, complete sequence	86448	2.39E+05	95%	99.94	JN983046.1
10	Salmonella enterica subsp. enterica serovar Heidelberg plasmid pSH696_117, complete sequence	86429	2.31E+05	92%	99.65	JN983047.1

Table S34: blast hits 2nd to 10th for p123

Blast hits	Description	Bit Score	Query Cover (%)	Identity (%)	Accession
2	Citrobacter freundii strain 680 plasmid p680_1, complete sequence	2.650e+05	93	99.982	CP038659
3	Enterobacter hormaechei strain EGYMCRVIM plasmid pMS-37a, complete sequence	2.536e+05	92	99.991	CP053191
4	Enterobacter hormaechei subsp. steigerwaltii strain ME-1 plasmid pME-1a, complete sequence	2.536e+05	94	99.991	CP041734
5	Enterobacter hormaechei strain WCHEH020038 plasmid pCTXM9_020038, complete sequence	2.536e+05	95	99.988	CP031724
6	Enterobacter hormaechei strain ZHH plasmid pZHH-1, complete sequence	2.535e+05	95	99.983	CP059712
7	Mixta calida strain 5098PV plasmid p5098PV-IncHI2, complete sequence	2.535e+05	96	99.980	CP061512
8	Enterobacter cloacae complex bacterium isolate C45 genome assembly, plasmid: pC45-VIM4	2.534e+05	95	99.972	LT991958
9	Enterobacter hormaechei strain ECC59 plasmid pECC59-1, complete sequence	2.468e+05	94	99.984	CP080471
10	Citrobacter freundii strain 565 plasmid p565_1, complete sequence	2.438e+05	86	99.981	CP038657

Table S35: blast hits 2nd to 10th for p127

Blast hits	Description	Bit Score	Query Cover (%)	Identity (%)	Accession
2	Salmonella enterica subsp. enterica serovar Infantis strain SPE100 plasmid pSPE100_vir	3.627e+05	99	99.978	CP040064
3	Salmonella enterica subsp. enterica serovar Infantis strain VNSEC002 plasmid pCFSAN086834, complete sequence	3.604e+05	100	99.971	CP039444
4	Salmonella enterica subsp. enterica serovar Infantis strain VNSEC003 plasmid pCFSAN086835, complete sequence	3.593e+05	100	99.972	CP039442

5	Salmonella enterica subsp. enterica serovar Infantis strain VNSEC001 plasmid pCFSAN086833, complete sequence	3.593e+05	100	99.971	CP039446
6	Salmonella enterica subsp. enterica serovar Infantis strain FARPER-219 plasmid p-F219, complete sequence	3.591e+05	99	99.955	CP038508
7	Salmonella enterica subsp. enterica serovar Infantis strain CVM N17S1592 plasmid pN17S1592, complete sequence	3.498e+05	93	99.982	CP082618
8	Salmonella enterica subsp. enterica serovar Infantis strain CVM N17S637 plasmid pN17S0637, complete sequence	3.494e+05	98	99.994	CP052808
9	Salmonella enterica subsp. enterica serovar Infantis strain CVM N19S0552 plasmid pN17S0637, complete sequence	3.362e+05	92	99.970	CP052791
10	Salmonella enterica subsp. enterica serovar Infantis strain CVM N17S1245 plasmid pN17S0637, complete sequence	3.356e+05	95	99.973	CP052826

Table S36: blast hits 2nd to 10th for p131

Blast hits	Description	Bit Score	Query Cover (%)	Identity (%)	Accession
2	Enterobacter hormaechei subsp. steigerwaltii strain ME-1 plasmid pME-1a, complete sequence	2.536e+05	97	99.996	CP041734
3	Enterobacter hormaechei strain WCHEH020038 plasmid pCTXM9_020038, complete sequence	2.536e+05	99	99.993	CP031724
4	Citrobacter freundii strain 154 plasmid p154_1, complete sequence	2.536e+05	99	99.993	CP038654
5	Enterobacter hormaechei strain ZHH plasmid pZHH-1, complete sequence	2.536e+05	98	99.989	CP059712
6	Mixta calida strain 5098PV plasmid p5098PV-IncHI2, complete sequence	2.536e+05	99	99.986	CP061512
7	Citrobacter freundii strain 680 plasmid p680_1, complete sequence	2.536e+05	96	99.987	CP038659
8	Enterobacter cloacae complex bacterium isolate C45 genome assembly, plasmid: pC45-VIM4	2.535e+05	98	99.977	LT991958
9	Enterobacter hormaechei strain ECC59 plasmid pECC59-1, complete sequence	2.468e+05	96	99.990	CP080471
10	Enterobacter hormaechei subsp. steigerwaltii isolate C309 genome assembly, plasmid: pC309-VIM4	2.415e+05	92	99.937	LT991955
